# Supplementary material for: Collective synthesis of acetylenic pharmaceuticals via enantioselective Nickel/Lewis acid-catalyzed propargylic alkylation
Source: Nat Commun. 2021 Jan 12;12:299. doi: 10.1038/s41467-020-20644-9 (PMC7803749; doi:10.1038/s41467-020-20644-9)
Supplement: Supplementary file 1 — Supplementary Information [file 41467_2020_20644_MOESM1_ESM.pdf]

Collective synthesis of acetylenic pharmaceuticals via enantioselective Nickel/Lewis acid-catalyzed propargylic alkylation

Xihao Chang, Jiayin Zhang, Lingzi Peng, Chang Guo*

Hefei National Laboratory for Physical Sciences at the Microscale, University of Science and Technology of China, Hefei 230026, China

*e-mail: guochang@ustc.edu.cn

CONTENTS

1. General information S2

2. Procedures for the synthesis of substrates S3

3. Characterization of substrates S6

4. Influence of base on the asymmetric propargylic alkylation reaction S11

5. Lewis acid effect for APS reaction S11

6. Stereochemical models for the asymmetric propargylic alkylation S12

7. General procedure for the synthesis of products S12

8. Derivatization reaction synthesis procedure S14

9. Characterization of products S18

10. X-ray Crystallography data S32

11. NMR spectra S33

12. HPLC traces S113

13. Supplementary References S158

1. General Information

Unless otherwise noted, all reagents were purchased from commercial suppliers and used without further purification. All reactions were carried out in flame-dried glassware under a dry nitrogen atmosphere. ^{1}H NMR and ^{13}C NMR spectra were recorded at 25 ºC on a Bruker Advance 400M or 500M NMR spectrometers ($CDCl_3$ as solvent). Chemical shifts for ^{1}H NMR spectra are reported as δ in units of parts per million (ppm) downfield from $SiMe_4$ (δ 0.00) and relative to the signal of chloroform-*d* (δ 7.26, singlet). Multiplicities were given as: s (singlet); d (doublet); t (triplet); q (quartet); dd (doublet of doublets); dt (doublet of triplets); m (multiplets), etc. Coupling constants are reported as a *J* value in Hz. ^{13}C NMR spectra are reported as δ in units of parts per million (ppm) downfield from $SiMe_4$ (δ 0.00) and relative to the signal of chloroform-*d* (δ 77.16, triplet). Melting points were measured on a digital melting point apparatus. Infrared (IR) spectra were recorded on a Nicolet MX-1E FT-IR spectrometer. Optical rotations were measured at 589 nm (sodium D line) by using a Perkin-Elmer-343 polarimeter (1 dm path length cell). High resolution mass spectral analysis (HRMS) was performed on Waters XEVO G2 Q-TOF. The enantiomericratio (*ee*) was determined by HPLC analysis on Chiral Daicel Chiralpak OD-H, AD-H, OJ-H, IB, IF, IG columns. The absolute configuration of **3e** was assigned by the X-ray analysis. The catalysts or reagents used in this article are commercial reagents if no corresponding synthetic route is given, and they have not undergone subsequent treatment.

2. Procedures for the synthesis of substrates

Substrates 1.

Propargylic carbonates **1** were prepared according to literature procedure.[1-5] Among them, the precursor propargyl alcohol compound for synthesis of **1x** was purchased commercially.

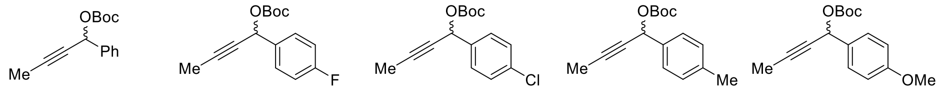

1a **1b** **1c** **1d** **1e**

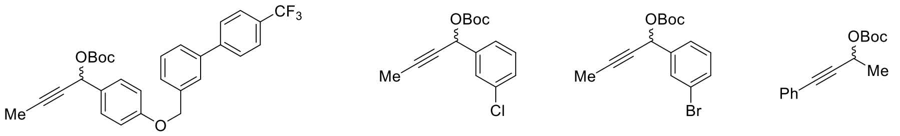

1f **1g** **1h** **1i**

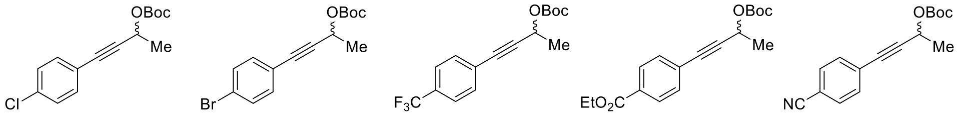

1j **1k** **1l** **1m** **1n**

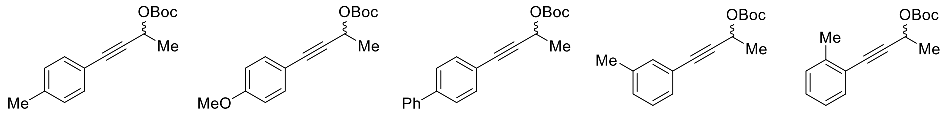

1o **1p** **1q** **1r** **1s**

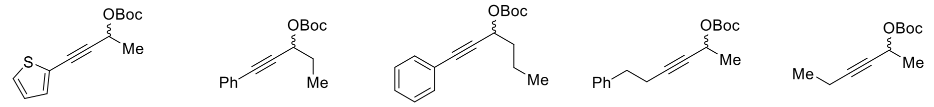

1t **1u** **1v** **1w** **1x**

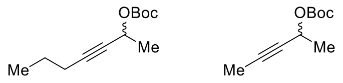

1y **1z**

General procedure A for the synthesis of 1i, 1j, 1k, 1l, 1m, 1n, 1o, 1p, 1q, 1r, 1s, 1t [1, 2]

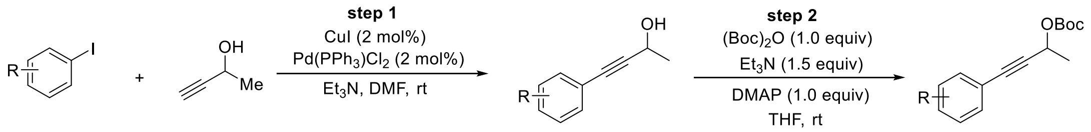

S1 **1-1**

Step 1: CuI (38.1 mg, 0.2 mmol) and $PdCl_2(PPh_3)_2$ (140.4 mg, 0.2 mmol) were charged into a two necked round flask and the flask was refilled with N_2. DMF (5 mL) was added to the flask. Aryl iodides (10 mmol), 3-butyn-2-ol (701 mg, 10 mmol) and triethylamine (10 mL, 72 mmol) were added to the mixture at room temperature. The reaction mixture was stirred at room temperature overnight. Saturated NH_4Cl solution (40 mL) was added to the mixture and the resulting aqueous phase was extracted three times with EtOAc. The combined organic phase was dried over anhydrous $MgSO_4$ and concentrated under reduced pressure. The residue was purified by silica gel column chromatography to obtain the desired aryl propargyl alcohol compounds **S1**.

Step 2: In a reaction flask, a mixture of $(Boc)_2O$ (1.0 equiv), Et_3N (1.5 equiv) and DMAP (1.0

equiv) in THF (0.3 M) were stirred at room temperature, followed by dropwise addition of the propargyl alcohol derivative **S1** (1.0 equiv). The reaction was monitored by TLC and then quenched with aqueous NH_4Cl solution and extracted with EtOAc (x3). The organic phase was washed with brine after which it was dried over anhydrous $MgSO_4$, filtered and evaporated under reduced pressure. The crude product was purified by silica gel column chromatography to give the desired *tert*-butyl propargyl carbonates **1-1**.

General procedure B for the synthesis of 1u, 1v, 1w, 1y [2, 3]

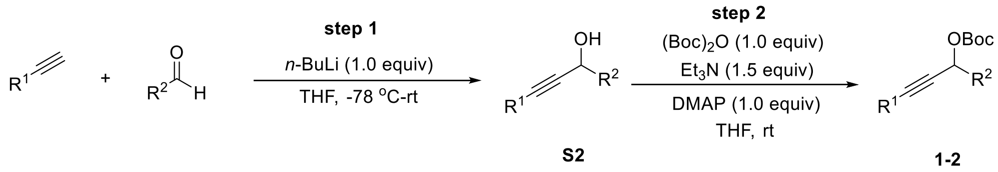

Step 1: *n*-BuLi (8 mL, 2.5 M in hexane, 20 mmol) was added to a stirred solution of alkyne compound (20 mmol, 1.0 equiv) in THF (20 mL) at -78 °C, and the mixture was stirred at -78 °C for 30 min. To the resulting solution was added aldehyde (26 mmol, 1.3 equiv) at -78 °C, and the mixture was gradually warmed to rt. The reaction was monitored by TLC and then quenched by the addition of water and extracted with EtOAc (x3). The organic phase was washed with brine after which it was dried over anhydrous $MgSO_4$, filtered and evaporated under reduced pressure. The residue was purified by silica gel column chromatography to obtain the desired propargyl alcohol compounds **S2**.
Step 2: The preparation of the subsequent *tert*-butyl propargyl carbonates **1-2** are the same as that of the step 2 in the previous general procedure A.

General procedure C for the synthesis of 1a, 1b, 1c, 1d, 1e, 1g, 1h, 1z [2, 4]

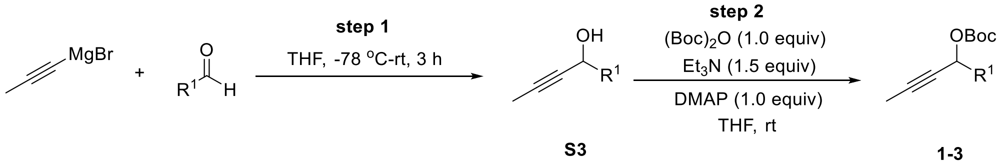

Step 1: To a dry 100 mL round-bottom flask containing a stir bar was added aldehyde (10 mmol). The flask was placed under an N_2 atmosphere, and cooled to -78 °C. A solution of 1-propynyl magnesium bromide (22 mL, 0.5 M in THF, 1.1 equiv) was added slowly while stirring. The mixture was stirred at -78 °C for 1 h, prior to allowing it to warm to room temperature, and stirring for an additional 2 h. Saturated NH_4Cl solution (20 mL) was added to the mixture slowly and the resulting aqueous phase was extracted three times with EtOAc. The combined organic phase was washed with water, then brine, and then dried over anhydrous $MgSO_4$. The organic mixture was concentrated under reduced pressure, and the concentrate was purified by silica gel flash chromatography to obtain the desired propargyl alcohol compounds **S3**.
Step 2: The preparation of the subsequent *tert*-butyl propargyl carbonates **1-3** are the same as that of the step 2 in the previous general procedure A.

Procedure for the synthesis of 1f [2, 4, 5]

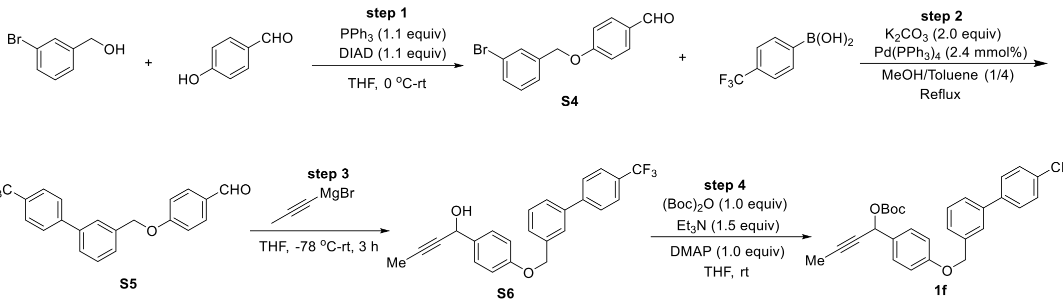

Step 1: To a stirred and ice-bath cooled mixture of the phenol (1.0 equiv) in anhydrous THF (9 ml), was added the 4-hydroxybenzaldehyde (1.0 equiv). Triphenylphosphine (PPh_3) (1.1 equiv) was added portionwise to this mixture and the system was kept under nitrogen. Subsequently, diisopropyl azodicarboxylate (DIAD) (1.1 equiv) was added in a strictly dropwise fashion. The reaction mixture was first stirred for 1 h at 0 ^oC and then overnight at room temperature. After completion of the reaction as indicated by TLC, the reaction mixture was concentrated under reduced pressure. The residue was diluted with EtOAc and the organic phase was washed successively with saturated aqueous $NaHCO_3$ and sodium chloride solutions, dried over anhydrous $MgSO_4$ and concentrated in vacuo affording an oily residue. The crude oil was purified by silica gel flash chromatography to obtain the desired ether derivative **S4**.

Step 2: A solvent mixture of MeOH (0.5 M), toluene (0.125 M) and potassium carbonate aqueous solution (2.0 equiv) were sonicated at room temperature for 30 min and then flushed with nitrogen for 30 min. Bromobenzene derivative (1.0 equiv), (4-(trifluoromethyl)phenyl)boronic acid (1.25 equiv), and tetrakis(triphenylphosphine) palladium(0) [$Pd(PPh_3)_4$] (0.024 equiv) were added sequentially at room temperature. The reaction mixture was refluxed with stirring under nitrogen atmosphere overnight. The reaction was ceased through stirring in open air for 30 min at room temperature forming a black emulsion. The insoluble material was filtered off through celite pad and the filtrate was diluted with EtOAc. The organic phase was washed successively with water, saturated aqueous sodium bicarbonate, and brine. After drying over anhydrous $MgSO_4$, the solvent was evaporated to dryness in-vacuo affording a brownish yellow sticky mass. The residue was purified by chromatography on silica gel to give the desired biaryl derivative **S5**.

Step 3: The preparation of the propargyl alcohol compound **S6** is the same as that of the step 1 in the previous general procedure C.

Step 4: The preparation of the subsequent *tert*-butyl propargyl carbonate **1f** is the same as that of the step 2 in the previous general procedure A.

Substrates 2.

Substituted malonate esters and methanetricarboxylates **2** were purchased from commercial suppliers and used without further purification.

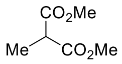

2a

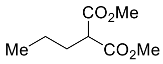

2b

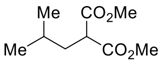

2c

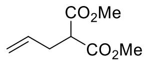

2d

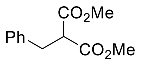

2e

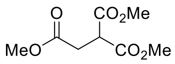

2f

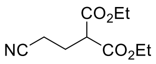

2g

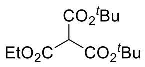

2h

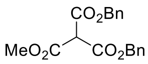

2i

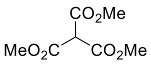

2j

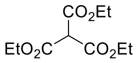

2k

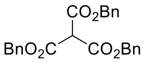

2l

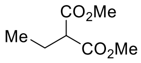

2m

3. Characterization of substrates

***tert*-butyl (1-phenylbut-2-yn-1-yl) carbonate (1a)**

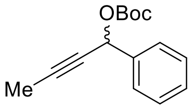

Yellow oil. **^{1}H NMR (400 MHz, $CDCl_3$)** δ 7.55 – 7.51 (m, 2H), 7.40 – 7.32 (m, 3H), 6.22 (q, *J* = 2.1 Hz, 1H), 1.90 (d, *J* = 2.2 Hz, 3H), 1.48 (s, 9H). **^{13}C NMR (100 MHz, $CDCl_3$)** δ 152.76, 137.51, 128.94, 128.68, 127.76, 84.60, 82.88, 75.85, 69.09, 27.89, 3.96. **ATR-FTIR (cm^{-1}):** 2983, 2933, 2921, 2300, 2240, 1801, 1739, 1479, 1456, 1267, 950, 871, 759. **ESI-MS:** calculated $[C_{15}H_{18}O_3 + Na]^+$: 269.1148, found: 269.1155.

***tert*-butyl (1-(4-fluorophenyl)but-2-yn-1-yl) carbonate (1b)**

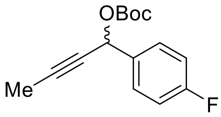

Yellow oil. **^{1}H NMR (400 MHz, $CDCl_3$)** δ 7.56 – 7.48 (m, 2H), 7.10 – 7.01 (m, 2H), 6.18 (q, *J* = 2.1 Hz, 1H), 1.90 (d, *J* = 2.2 Hz, 3H), 1.48 (s, 9H). **^{19}F NMR (375 MHz, $CDCl_3$)** δ -112.85 (s). **^{13}C NMR (100 MHz, $CDCl_3$)** δ 163.08 (d, *J* = 246.1 Hz), 152.68, 133.46 (d, *J* = 3.2 Hz), 129.80 (d, *J* = 8.5 Hz), 115.59 (d, *J* = 21.6 Hz), 84.85, 83.06, 75.65, 68.38, 27.88, 3.94. **ATR-FTIR (cm^{-1}):** 2981, 2933, 2237, 1743, 1606, 1510, 1274, 1145, 875, 840. **ESI-MS:** calculated $[C_{15}H_{17}FO_3 + Na]^+$: 287.1054, found: 287.1056.

***tert*-butyl (1-(4-chlorophenyl)but-2-yn-1-yl) carbonate (1c)**

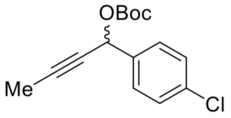

Yellow oil. **^{1}H NMR (400 MHz, $CDCl_3$)** δ 7.49 – 7.44 (m, 2H), 7.36 – 7.31 (m, 2H), 6.17 (q, *J* = 2.1 Hz, 1H), 1.90 (d, *J* = 2.2 Hz, 3H), 1.48 (s, 9H). **^{13}C NMR (100 MHz, $CDCl_3$)** δ 152.63, 136.09, 134.87, 129.20, 128.87, 85.00, 83.15, 75.45, 68.31, 27.88, 3.95. **ATR-FTIR (cm^{-1}):** 2981, 2933, 2238, 1743, 1596, 1492, 1369, 1274, 1145, 873, 848. **ESI-MS:** calculated $[C_{15}H_{17}ClO_3 + Na]^+$: 303.0758, found: 303.0754.

***tert*-butyl (1-(p-tolyl)but-2-yn-1-yl) carbonate (1d)**

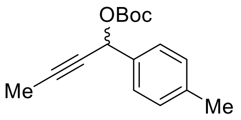

Yellow oil. **^{1}H NMR (400 MHz, $CDCl_3$)** δ 7.45 – 7.39 (m, 2H), 7.18 (d, *J* = 7.9 Hz, 2H), 6.19 (q, *J* = 2.1 Hz, 1H), 2.35 (s, 3H), 1.89 (d, *J* = 2.2 Hz, 3H), 1.48 (s, 9H). **^{13}C NMR (100 MHz, $CDCl_3$)** δ 152.79, 138.83, 134.65, 129.34, 127.72, 84.33, 82.74, 76.00, 68.98, 27.88, 21.33, 3.94. **ATR-FTIR (cm^{-1}):** 2981, 2923, 2237, 1741, 1513, 1457, 1369, 1272, 1270, 1145, 873. **ESI-MS:** calculated $[C_{16}H_{20}O_3 + Na]^+$: 283.1305, found: 283.1306.

***tert*-butyl (1-(4-methoxyphenyl)but-2-yn-1-yl) carbonate (1e)**

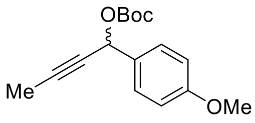

Yellow oil. **^{1}H NMR (500 MHz, $CDCl_3$)** δ 7.49 – 7.44 (m, 2H), 6.91 – 6.86 (m, 2H), 6.17 (q, *J* = 2.0 Hz, 1H), 3.80 (s, 3H), 1.90 (d, *J* = 2.2 Hz, 3H), 1.47 (s, 9H). **^{13}C NMR (125 MHz, $CDCl_3$)** δ 160.15, 152.83, 129.79, 129.40, 114.02, 84.36, 82.78, 76.03, 68.87, 55.45, 27.92, 3.99. **ATR-FTIR (cm^{-1}):** 2979, 2921, 2850, 2237, 1739, 1513, 1369, 1249, 1145, 871, 833. **ESI-MS:** calculated $[C_{16}H_{20}O_4 + Na]^+$: 299.1254, found: 299.1257.

***tert*-butyl (1-(4-((4'-(trifluoromethyl)-[1,1'-biphenyl]-3-yl)methoxy)phenyl)but-2-yn-1-yl) carbonate (1f)**

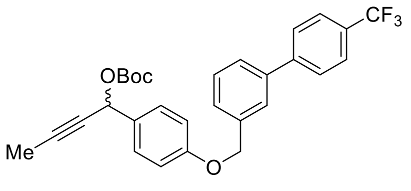

Yellow oil. **^{1}H NMR (500 MHz, $CDCl_3$)** δ 7.73 – 7.68 (m, 4H), 7.66 (s, 1H), 7.58 – 7.54 (m, 1H), 7.51 – 7.41 (m, 4H), 6.98 – 6.94 (m, 2H), 5.18 (q, *J* = 2.2, 1H), 5.13 (s, 2H), 1.86 (d, *J* = 2.1 Hz, 3H), 1.31 (s, 9H). **^{19}F NMR (470 MHz, $CDCl_3$)** δ -62.40 (s). **^{13}C NMR (125 MHz, $CDCl_3$)** δ 158.26, 144.56, 140.27, 138.08, 134.68, 129.59 (q, *J* = 32.5 Hz), 129.39, 128.26, 127.63, 127.38, 127.02, 126.47, 125.86 (q, *J* = 3.7 Hz), 124.40 (q, *J* = 270.3 Hz), 114.74, 81.91, 80.67, 75.40, 69.99, 64.01, 28.60, 4.01. **ATR-FTIR (cm^{-1}):** 2971, 2921, 2852, 2221, 1754, 1510, 1469, 1367, 1326, 1240, 1126, 1072, 842, 790. **ESI-MS:** calculated $[C_{29}H_{27}F_3O_4 + Na]^+$: 519.1754, found: 519.1760.

***tert*-butyl (1-(3-chlorophenyl)but-2-yn-1-yl) carbonate (1g)**

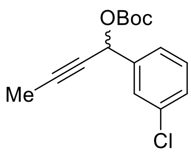

Yellow oil. **^{1}H NMR (400 MHz, $CDCl_3$)** δ 7.54 – 7.52 (m, 1H), 7.41 – 7.37 (m, 1H), 7.32 – 7.26 (m, 2H), 6.18 (q, *J* = 2.1 Hz, 1H), 1.89 (d, *J* = 2.3 Hz, 3H), 1.48 (s, 9H). **^{13}C NMR (100 MHz, $CDCl_3$)** δ 152.44, 139.35, 134.40, 129.84, 128.95, 127.73, 125.74, 84.96, 82.97, 75.20, 68.07, 27.71, 3.77. **ATR-FTIR (cm^{-1}):** 2981, 2923, 2242, 1743, 1577, 1475, 1255, 1147, 854, 788, 765, 690. **ESI-MS:** calculated $[C_{15}H_{17}ClO_3 + Na]^+$: 303.0758, found: 303.0764.

1-(3-bromophenyl)but-2-yn-1-yl *tert*-butyl carbonate (1h)

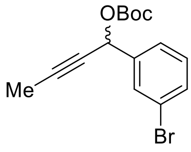

Yellow oil. **^{1}H NMR (400 MHz, $CDCl_3$)** δ 7.68 (t, *J* = 1.8 Hz, 1H), 7.48 – 7.42 (m, 2H), 7.26 – 7.20 (m, 1H), 6.16 (q, *J* = 2.1 Hz, 1H), 1.90 (d, *J* = 2.2 Hz, 3H), 1.48 (s, 9H). **^{13}C NMR (100 MHz, $CDCl_3$)** δ 152.55, 139.66, 132.02, 130.77, 130.22, 126.34, 122.65, 85.18, 83.22, 75.25, 68.16, 27.86, 3.96. **ATR-FTIR (cm^{-1}):** 2981, 2921, 2852, 2242, 1743, 1573, 1475, 1253, 1145, 852, 788, 765, 526. **ESI-MS:** calculated $[C_{15}H_{17}BrO_3 + Na]^+$: 347.0253, found: 347.0255.

***tert*-butyl (4-phenylbut-3-yn-2-yl) carbonate (1i)**

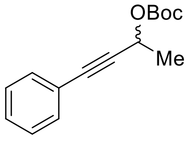

Yellow oil. **^{1}H NMR (400 MHz, $CDCl_3$)** δ 7.45 – 7.40 (m, 2H), 7.34 – 7.27 (m, 3H), 5.50 (q, *J* = 6.7 Hz, 1H), 1.62 (d, *J* = 6.7 Hz, 3H), 1.51 (s, 9H). **^{13}C NMR (100 MHz, $CDCl_3$)** δ 152.65, 131.94, 128.69, 128.35, 122.43, 87.20, 85.15, 82.77, 63.85, 27.90, 21.68. **ATR-FTIR (cm^{-1}):** 2983, 2937, 2233,

1743, 1490, 1369, 1276, 1162, 837, 757. **ESI-MS:** calculated [$C_{15}H_{18}O_3$ + Na]$^+$: 269.1148, found: 269.1153.

***tert*-butyl (4-(4-chlorophenyl)but-3-yn-2-yl) carbonate (1j)**

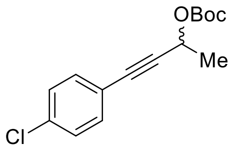

Yellow oil. **^{1}H NMR (400 MHz, $CDCl_3$)** δ 7.38 – 7.34 (m, 2H), 7.30 – 7.26 (m, 2H), 5.48 (q, *J* = 6.7 Hz, 1H), 1.61 (d, *J* = 6.7 Hz, 3H), 1.51 (s, 9H). **^{13}C NMR (100 MHz, $CDCl_3$)** δ 152.61, 134.79, 133.19, 128.73, 120.92, 88.19, 84.04, 82.90, 63.72, 27.90, 21.58. **ATR-FTIR (cm^{-1}):** 2983, 2937, 2237, 1743, 1592, 1490, 1276, 1162, 1089, 831, 792. **ESI-MS:** calculated [$C_{15}H_{17}ClO_3$ + Na]$^+$: 303.0758, found: 303.0773.

4-(4-bromophenyl)but-3-yn-2-yl *tert*-butyl carbonate (1k)

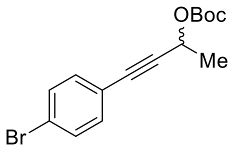

Yellow oil. **^{1}H NMR (400 MHz, $CDCl_3$)** δ 7.46 – 7.41 (m, 2H), 7.31 – 7.27 (m, 2H), 5.47 (q, *J* = 6.7 Hz, 1H), 1.60 (d, *J* = 6.7 Hz, 3H), 1.51 (s, 9H). **^{13}C NMR (100 MHz, $CDCl_3$)** δ 152.61, 133.40, 131.66, 123.03, 121.40, 88.38, 84.11, 82.92, 63.72, 27.90, 21.56. **ATR-FTIR (cm^{-1}):** 2983, 2935, 2237, 1743, 1486, 1276, 1162, 1093, 836, 524. **ESI-MS:** calculated [$C_{15}H_{17}BrO_3$ + Na]$^+$: 347.0253, found: 347.0253.

***tert*-butyl (4-(4-(trifluoromethyl)phenyl)but-3-yn-2-yl) carbonate (1l)**

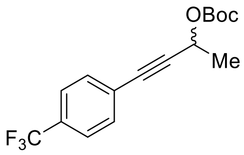

Yellow oil. **^{1}H NMR (400 MHz, $CDCl_3$)** δ 7.58 – 7.50 (m, 4H), 5.50 (q, *J* = 6.7 Hz, 1H), 1.62 (d, *J* = 6.7 Hz, 3H), 1.51 (s, 9H). **^{19}F NMR (375 MHz, $CDCl_3$)** δ -62.89 (s). **^{13}C NMR (100 MHz, $CDCl_3$)** δ 152.59, 132.20, 130.47 (q, *J* = 32.5 Hz), 126.26 (q, *J* = 1.3 Hz), 125.33 (q, *J* = 3.8 Hz), 123.98 (q, *J* = 270.6 Hz), 89.67, 83.78, 83.05, 63.58, 27.90, 21.49. **ATR-FTIR (cm^{-1}):** 2985, 2935, 2244, 1745, 1479, 1405, 1324, 1276, 1168, 842. **ESI-MS:** calculated [$C_{16}H_{17}F_3O_3$ + Na]$^+$: 337.1022, found: 337.1025.

ethyl 4-(3-((*tert*-butoxycarbonyl)oxy)but-1-yn-1-yl)benzoate (1m)

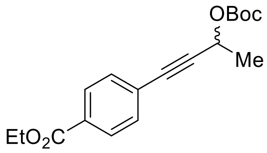

Yellow solid. **M.p.** 41-42 °C. **^{1}H NMR (400 MHz, $CDCl_3$)** δ 8.00 – 7.94 (m, 2H), 7.51 – 7.45 (m, 2H), 5.50 (q, *J* = 6.7 Hz, 1H), 4.36 (q, *J* = 7.1 Hz, 2H), 1.62 (d, *J* = 6.7 Hz, 3H), 1.51 (s, 9H), 1.38 (t, *J* = 7.1 Hz, 3H). **^{13}C NMR (100 MHz, $CDCl_3$)** δ 166.11, 152.59, 131.82, 130.35, 129.49, 126.97, 90.04, 84.42, 82.97, 63.65, 61.29, 27.89, 21.52, 14.42. **ATR-FTIR (cm^{-1}):** 2989, 2917, 2850, 2231, 1745, 1720, 1375, 1276, 1162, 836, 771. **ESI-MS:** calculated [$C_{18}H_{22}O_5$ + H]$^+$: 319.1540, found: 319.1559.

***tert*-butyl (4-(4-cyanophenyl)but-3-yn-2-yl) carbonate (1n)**

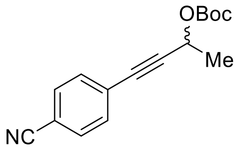

Yellow oil. **^{1}H NMR (500 MHz, $CDCl_3$)** δ 7.59 (d, *J* = 8.3 Hz, 2H), 7.51 (d, *J* = 8.3 Hz, 2H), 5.49 (q, *J* = 6.7 Hz, 1H), 1.62 (d, *J* = 6.7 Hz, 3H), 1.51 (s, 9H). **^{13}C NMR (125 MHz, $CDCl_3$)** δ 152.52, 132.45, 132.10, 127.33, 118.47, 112.14, 91.62, 83.46, 83.14, 63.44, 27.88, 21.37. **ATR-FTIR (cm^{-1}):** 2287, 2937, 2229, 1747, 1604, 1502, 1369, 1274, 840, 792. **ESI-MS:** calculated [$C_{16}H_{17}NO_3$ + Na]$^+$: 294.1101, found: 294.1105.

tert-butyl (4-(p-tolyl)but-3-yn-2-yl) carbonate (1o)

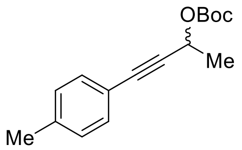

Yellow oil. **^{1}H NMR (400 MHz, $CDCl_3$)** δ 7.35 – 7.30 (m, 2H), 7.10 (d, *J* = 7.9 Hz, 2H), 5.49 (q, *J* = 6.7 Hz, 1H), 2.34 (s, 3H), 1.61 (d, *J* = 6.7 Hz, 3H), 1.51 (s, 9H). **^{13}C NMR (100 MHz, $CDCl_3$)** δ 152.68, 138.83, 131.86, 129.11, 119.36, 86.51, 85.32, 82.73, 63.96, 27.91, 21.75, 21.61. **ATR-FTIR (cm^{-1}):** 2983, 2937, 2871, 2233, 1743, 1510, 1456, 1369, 1274, 1164, 1093, 817, 750. **ESI-MS:** calculated $[C_{16}H_{20}O_3 + Na]^+$: 283.1305, found: 283.1314.

tert-butyl (4-(4-methoxyphenyl)but-3-yn-2-yl) carbonate (1p)

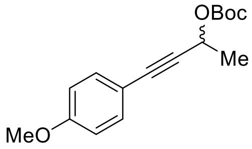

Yellow oil. **^{1}H NMR (400 MHz, $CDCl_3$)** δ 7.40 – 7.33 (m, 2H), 6.84 – 6.79 (m, 2H), 5.48 (q, *J* = 6.7 Hz, 1H), 3.79 (s, 3H), 1.60 (d, *J* = 6.7 Hz, 3H), 1.50 (s, 9H). **^{13}C NMR (100 MHz, $CDCl_3$)** δ 159.90, 152.66, 133.43, 114.48, 113.95, 85.83, 85.11, 82.65, 63.99, 55.35, 27.88, 21.76. **ATR-FTIR (cm^{-1}):** 2983, 2937, 2838, 2229, 1743, 1510, 1458, 1276, 1172, 1093, 835, 792. **ESI-MS:** calculated $[C_{16}H_{20}O_4 + Na]^+$: 299.1254, found: 299.1257.

4-([1,1'-biphenyl]-4-yl)but-3-yn-2-yl _tert_-butyl carbonate (1q)

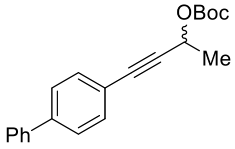

Yellow solid. **M.p.** 58-60 °C. **^{1}H NMR (500 MHz, $CDCl_3$)** δ 7.60 – 7.50 (m, 6H), 7.47 – 7.42 (m, 2H), 7.38 – 7.33 (m, 1H), 5.53 (q, *J* = 6.7 Hz, 1H), 1.64 (d, *J* = 6.7 Hz, 3H), 1.53 (s, 9H). **^{13}C NMR (125 MHz, $CDCl_3$)** δ 152.66, 141.43, 140.39, 132.39, 128.98, 127.80, 127.15, 127.04, 121.31, 87.83, 85.03, 82.83, 63.91, 27.91, 21.71. **ATR-FTIR (cm^{-1}):** 2983, 2921, 2850, 2233, 1743, 1486, 1369, 1276, 1162, 838, 763, 698. **ESI-MS:** calculated $[C_{21}H_{22}O_3 + Na]^+$: 345.1461, found: 345.1457.

tert-butyl (4-(m-tolyl)but-3-yn-2-yl) carbonate (1r)

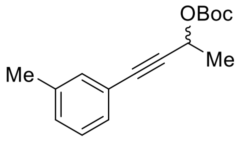

Yellow oil. **^{1}H NMR (400 MHz, $CDCl_3$)** δ 7.27 – 7.21 (m, 2H), 7.20 – 7.15 (m, 1H), 7.13 – 7.09 (m, 1H), 5.49 (q, *J* = 6.7 Hz, 1H), 2.30 (s, 3H), 1.60 (d, *J* = 6.7 Hz, 3H), 1.51 (s, 9H). **^{13}C NMR (100 MHz, $CDCl_3$)** δ 152.59, 137.93, 132.45, 129.51, 128.94, 128.19, 122.16, 86.78, 85.26, 82.59, 63.78, 27.81, 21.63, 21.20. **ATR-FTIR (cm^{-1}):** 2983, 2921, 2850, 2238, 1743, 1484, 1369, 1277, 1162, 852, 786, 690. **ESI-MS:** calculated $[C_{16}H_{20}O_3 + Na]^+$: 283.1305, found: 283.1311.

tert-butyl (4-(o-tolyl)but-3-yn-2-yl) carbonate (1s)

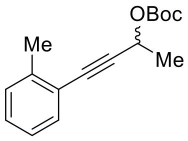

Yellow oil. **^{1}H NMR (400 MHz, $CDCl_3$)** δ 7.41 – 7.36 (m, 1H), 7.24 – 7.16 (m, 2H), 7.15 – 7.09 (m, 1H), 5.52 (q, *J* = 6.7 Hz, 1H), 2.41 (s, 3H), 1.63 (d, *J* = 6.7 Hz, 3H), 1.51 (s, 9H). **^{13}C NMR (100 MHz, $CDCl_3$)** δ 152.70, 140.66, 132.18, 129.51, 128.71, 125.59, 122.21, 91.24, 84.08, 82.75, 63.99, 27.92, 21.74, 20.70. **ATR-FTIR (cm^{-1}):** 2983, 2921, 2850, 2229, 1745, 1484, 1456, 1274, 923, 890, 839, 757. **ESI-MS:** calculated $[C_{16}H_{20}O_3 + Na]^+$: 283.1305, found: 283.1315.

***tert*-butyl (4-(thiophen-2-yl)but-3-yn-2-yl) carbonate (1t)**

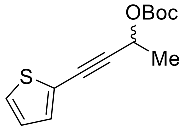

Yellow oil. **^{1}H NMR (500 MHz, $CDCl_3$)** δ 7.28 – 7.23 (m, 1H), 7.23 – 7.20 (m, 1H), 6.99 – 6.93 (m, 1H), 5.50 (q, *J* = 6.7 Hz, 1H), 1.61 (d, *J* = 6.7 Hz, 3H), 1.51 (s, 9H). **^{13}C NMR (125 MHz, $CDCl_3$)** δ 152.58, 132.81, 127.70, 127.02, 122.27, 91.00, 82.88, 78.55, 63.85, 27.88, 21.50. **ATR-FTIR (cm^{-1}):** 2983, 2935, 2873, 2225, 1743, 1519, 1456, 1369, 1274, 1160, 1093, 829, 703. **ESI-MS:** calculated $[C_{13}H_{16}O_3S + Na]^+$: 275.0712, found: 275.0725.

***tert*-butyl (1-phenylpent-1-yn-3-yl) carbonate (1u)**

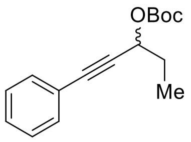

Yellow oil. **^{1}H NMR (400 MHz, $CDCl_3$)** δ 7.47 – 7.41 (m, 2H), 7.34 – 7.27 (m, 3H), 5.36 (t, *J* = 6.5 Hz, 1H), 1.97 – 1.86 (m, 2H), 1.51 (s, 9H), 1.09 (t, *J* = 7.4 Hz, 3H). **^{13}C NMR (100 MHz, $CDCl_3$)** δ 152.89, 131.98, 128.66, 128.35, 122.54, 86.21, 85.92, 82.72, 68.83, 28.44, 27.91, 9.59. **ATR-FTIR (cm^{-1}):** 2977, 2937, 2881, 2238, 1743, 1598, 1490, 1457, 1251, 898, 759. **ESI-MS:** calculated $[C_{16}H_{20}O_3 + Na]^+$: 283.1305, found: 283.1315.

***tert*-butyl (1-phenylhex-1-yn-3-yl) carbonate (1v)**

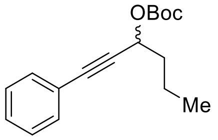

Yellow oil. **^{1}H NMR (400 MHz, $CDCl_3$)** δ 7.46 – 7.41 (m, 2H), 7.33 – 7.26 (m, 3H), 5.41 (t, *J* = 6.7 Hz, 1H), 1.92 – 1.80 (m, 2H), 1.59 – 1.52 (m, 2H), 1.51 (s, 9H), 0.98 (t, *J* = 7.4 Hz, 3H). **^{13}C NMR (100 MHz, $CDCl_3$)** δ 152.90, 131.97, 128.65, 128.35, 122.55, 86.46, 85.83, 82.72, 67.55, 37.18, 27.92, 18.55, 13.78. **ATR-FTIR (cm^{-1}):** 2964, 2933, 2875, 2237, 1743, 1598, 1490, 1459, 1272, 754, 692. **ESI-MS:** calculated $[C_{17}H_{22}O_3 + Na]^+$: 297.1461, found: 297.1467.

***tert*-butyl (6-phenylhex-3-yn-2-yl) carbonate (1w)**

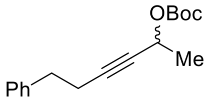

Yellow oil. **^{1}H NMR (400 MHz, $CDCl_3$)** δ 7.32 – 7.26 (m, 2H), 7.24 – 7.18 (m, 3H), 5.32 – 5.15 (m, 1H), 2.82 (t, *J* = 7.6 Hz, 2H), 2.54 – 2.43 (m, 2H), 1.50 (s, 9H), 1.48 (d, *J* = 6.7 Hz, 3H). **^{13}C NMR (100 MHz, $CDCl_3$)** δ 152.71, 140.65, 128.58, 128.47, 126.41, 85.33, 82.53, 79.19, 63.74, 34.97, 27.90, 21.86, 21.09. **ATR-FTIR (cm^{-1}):** 2985, 2935, 2246, 1741, 1496, 1454, 1272, 1159, 840, 750, 700. **ESI-MS:** calculated $[C_{17}H_{22}O_3 + Na]^+$: 297.1461, found: 297.1462.

***tert*-butyl hex-3-yn-2-yl carbonate (1x)**

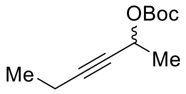

Colorless oil. **^{1}H NMR (500 MHz, $CDCl_3$)** δ 5.27 – 5.20 (m, 1H), 2.23 – 2.17 (m, 2H), 1.50 – 1.46 (m, 12H), 1.11 (t, *J* = 7.5 Hz, 3H). **^{13}C NMR (125 MHz, $CDCl_3$)** δ 152.72, 87.44, 82.50, 77.70, 63.84, 27.89, 21.95, 13.72, 12.50. **ATR-FTIR (cm^{-1}):** 2981, 2937, 2244, 1745, 1456, 1369, 1272, 1160, 840. **ESI-MS:** calculated $[C_{11}H_{18}O_3 + Na]^+$: 221.1148, found: 221.1146.

***tert*-butyl hex-3-yn-2-yl carbonate (1y)**

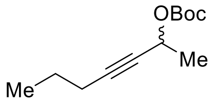

Yellow oil. **^{1}H NMR (500 MHz, $CDCl_3$)** δ 5.31 – 5.20 (m, 1H), 2.18 – 2.14 (m, 2H), 1.54 – 1.44 (m, 14H), 0.95 (t, *J* = 7.4 Hz, 3H). **^{13}C NMR (125 MHz, $CDCl_3$)** δ 152.72, 86.01, 82.47, 78.59, 63.87, 27.88, 21.98,

21.95, 20.75, 13.50. **ATR-FTIR (cm^{-1}):** 2967, 2937, 2875, 2246, 1745, 1456, 1369, 1257, 1162, 845. **ESI-MS:** calculated $[C_{12}H_{20}O_3 + Na]^+$: 235.1305, found: 235.1298.

***tert*-butyl pent-3-yn-2-yl carbonate (1z)**

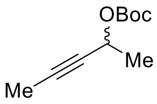

Colorless oil. **^1H NMR (500 MHz, $CDCl_3$)** δ 5.23 – 5.17 (m, 1H), 1.81 (d, *J* = 2.1 Hz, 3H), 1.48 – 1.44 (m, 12H). **^{13}C NMR (125 MHz, $CDCl_3$)** δ 152.71, 82.47, 81.67, 77.53, 63.76, 27.86, 21.83, 3.71. **ATR-FTIR (cm^{-1}):** 2983, 2937, 2252, 1745, 1456, 1369, 1270, 1159, 846. **ESI-MS:** calculated $[C_{10}H_{16}O_3 + Na]^+$: 207.0992, found: 207.0996.

4. Influence of base on the asymmetric propargylic alkylation reaction

Supplementary Table 1 | Influence of base on the asymmetric propargylic alkylation reaction

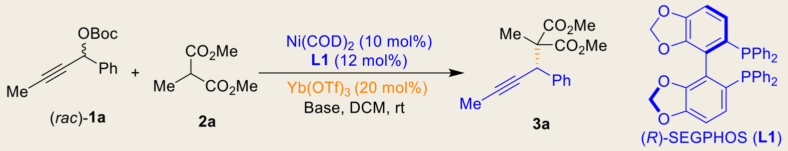

Entry	Base	Yield (%)	e.e. (%)
1	-	nr	-
2	K_2CO_3	nr	-
3	Na_2CO_3	nr	-
4	KO^{t}Bu	39	68
5	NaOMe	nr	-
6	NaHMDS	54	79
7	LiHMDS	81	82
8	DIPEA	nr	-
9	Pyridine	nr	-
10	**Cs_2CO_3**	**76**	**95**

Reactions were conducted with **1a** (0.225 mmol), **2a** (0.15 mmol), base (0.3 mmol), $Ni(cod)_2$ (10 mol%) , **L1** (12 mol%) and $Yb(OTf)_3$ (0.03mmol) in DCM (2.0 mL) at room temperature for 72 h. e.e. values were determined by high-performance liquid chromatography analysis. nr = no reaction. rt = room temperature.

5. Lewis acid effect for APS reaction

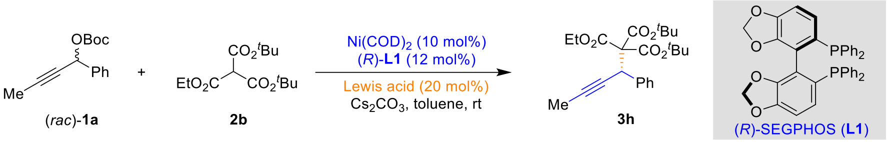

Entry	Lewis acid	Yield (%)	e.e. (%)
1	No lewis acid	32	96
2	$Cu(OTf)_2$	14	94
3	$Yb(OTf)_3$	76	95
4	$Zn(OTf)_2$	40	96
5	$Sc(OTf)_2$	4	94
6	$Sn(OTf)_2$	9	94
7	$Fe(OTf)_2$	43	96

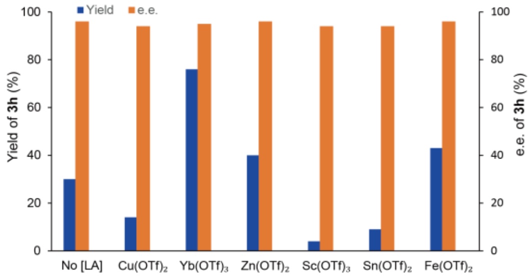

Supplementary Figure 1 Lewis acid effect for APS reaction

6. Stereochemical models for the asymmetric propargylic alkylation

We propose the possible reaction pathways and stereochemical models as shown in Supplementary Figure 2. Our transformation initiates with the generation of the electrophilic allenylnickel species **I** via decarboxylation of the propargylic carbonates **1a**. Meanwhile, the challenging asymmetric propargylation might be enhanced through the introduction of a Lewis acid cocatalyst via in situ deprotonation of acidic hydrocarbons providing access to the generation of nucleophilic enolate species. During the subsequent reaction of allenylnickel species **I** and enolate species, direct nucleophilic substitution may occurs selectively at the propargylic carbon affords the corresponding alkylation adduct (*R*)-**3a** as the major stereoisomer through the favored **TS I**.

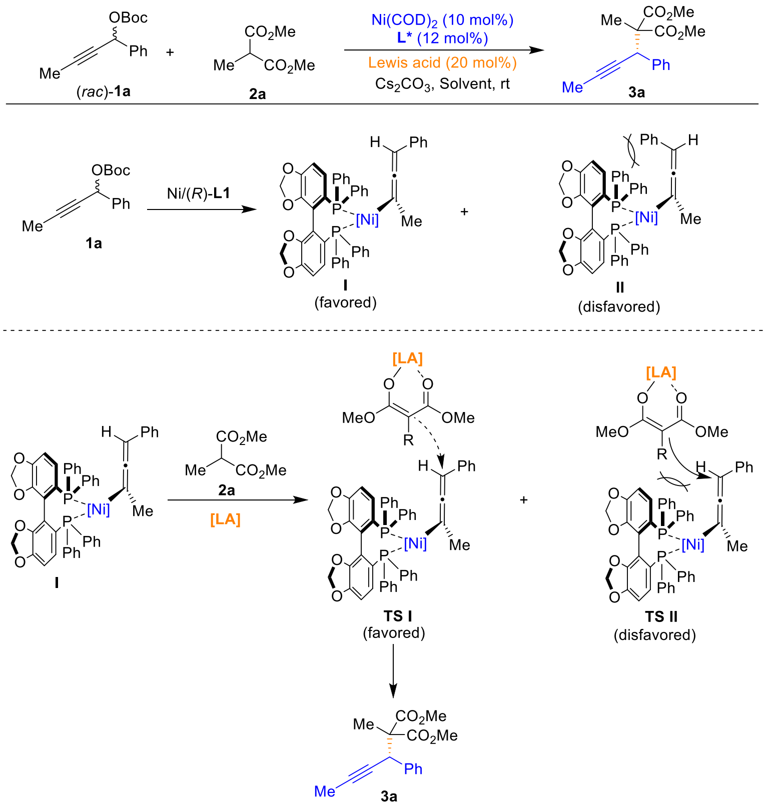

Supplementary Figure 2 Stereochemical models for the asymmetric propargylic alkylation

7. General procedure for the synthesis of products

7.1. Synthesis of racemic products

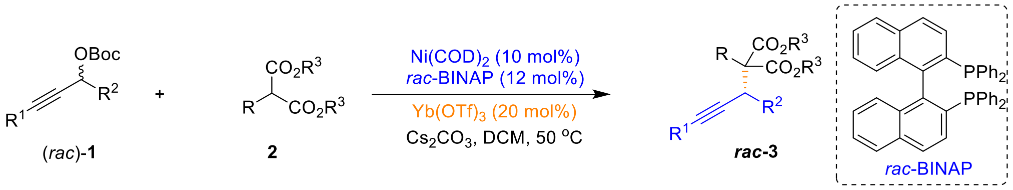

In a nitrogen-filled glove box, an oven-dried 10 mL Schlenk tube equipped with a stir bar was charged with $Ni(COD)_2$ (4.1 mg, 0.015 mmol, 10 mol%) and *rac*-BINAP (11.2 mg, 0.018 mmol, 12 mol%), and stirred in DCM (2 mL) for about 15 minutes at 50 °C. Then 0.225 mmol **1** (1.5 equiv), 0.15 mmol **2** (1.0 equiv), $Yb(OTf)_3$ (18.6 mg, 0.03 mmol, 20 mol%) and

Cs_2CO_3 (97.7 mg, 0.3 mmol, 2.0 equiv) were added to the tube subsequently under nitrogen atmosphere. The final solution was stirred for about 72 h at 50 °C until complete consumption of the substrate **2** (monitored by TLC). The solution was diluted with DCM and washed with water. The aqueous phase was then extracted three times with DCM. The combined organic phase was dried over anhydrous $MgSO_4$ and concentrated under reduced pressure. The residue was purified by silica gel chromatography to afford the desired racemic products **3**.

7.2. Synthesis of asymmetric products

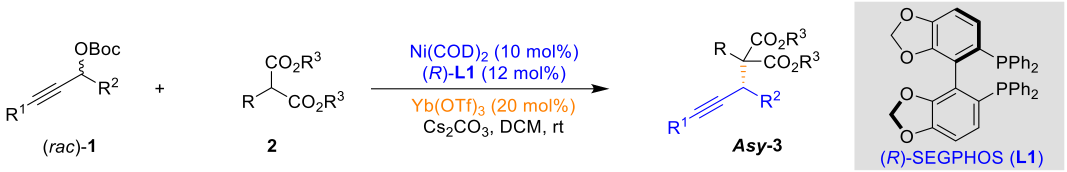

In a nitrogen-filled glove box, an oven-dried 10 mL Schlenk tube equipped with a stir bar was charged with $Ni(COD)_2$ (4.1 mg, 0.015 mmol, 10 mol%) and *R*-segphos (11.0 mg, 0.018 mmol, 12 mol%), and stirred in DCM (2 mL) for about 15 minutes at room temperature. Then 0.225 mmol **1** (1.5 equiv), 0.15 mmol **2** (1.0 equiv), $Yb(OTf)_3$ (18.6 mg, 0.03 mmol, 20 mol%) and Cs_2CO_3 (97.7 mg, 0.3 mmol, 2.0 equiv) were added to the tube subsequently under nitrogen atmosphere. The final solution was stirred for about 72 h at room temperature until complete consumption of the substrate **2** (monitored by TLC). The solution was diluted with DCM and washed with water. The aqueous phase was then extracted three times with DCM. The combined organic phase was dried over anhydrous $MgSO_4$ and concentrated under reduced pressure. The residue was purified by silica gel chromatography to afford the desired asymmetric products **3**.

Caution: **It is worth noting that the polarity of the substrates 2 and the products 3 in the reactions are very close.** It could lead to better separation of products **3** with dichloromethane as the eluent (Supplementary Figure 3, **TLC visualization reagents: aqueous alkaline $KMnO_4$ solution**).

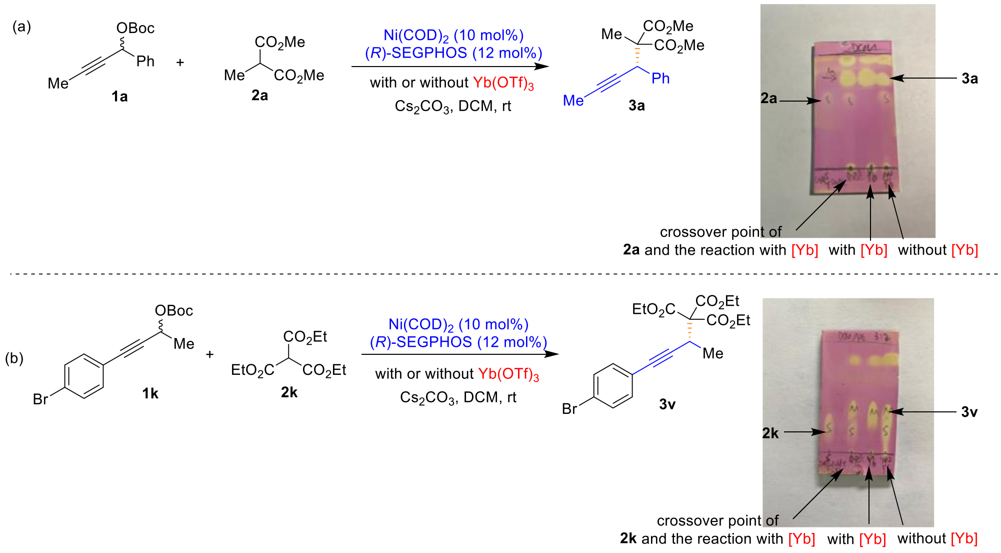

Supplementary Figure 3 Photographic guide for TLC plates of the APS reaction

8. Derivatization reaction synthesis procedure

8.1 Procedure for synthesis of (-)-Thiohexital (6) [6]

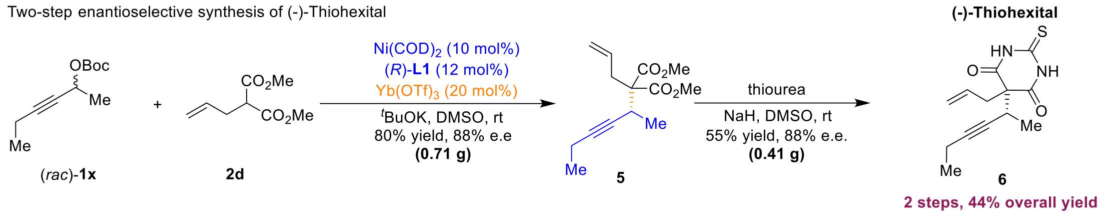

In a nitrogen-filled glove box, an oven-dried 100 mL Schlenk tube equipped with a stir bar was charged with Ni(COD)$_2$ (96.3 mg, 0.35 mmol, 10 mol%) and *R*-segphos (256 mg, 0.42 mmol, 12 mol%), and stirred in toluene (40 mL) for about 15 minutes at rt. Then 7.0 mmol **1x** (1.4 g, 2.0 equiv), 3.5 mmol **2d** (603 mg, 1.0 equiv), Yb(OTf)$_3$ (434.2 mg, 0.7 mmol, 20 mol%) and ^{t}BuOK (785.5 mg, 7.0 mmol, 2.0 equiv) were added to the tube subsequently under nitrogen atmosphere. The final solution was stirred for about 72 h at room temperature until complete consumption of the substrate **2d** (monitored by TLC). The solution was diluted with ethyl acetate and washed with water. The aqueous phase was then extracted three times with ethyl acetate. The combined organic phase was dried over anhydrous MgSO$_4$ and concentrated under reduced pressure. The residue was purified by silica gel chromatography to afford the desired products **5** (0.71 g, 80% yield).

Dry dimethylsulfoxide (30 mL), malonic ester **5** (707 mg, 2.8 mmol, 1.0 equiv) and thiourea (426 mg, 5.6 mmol, 2.0 equiv) were stirred and treated with a 60% dispersion of sodium hydride in mineral oil (336 mg, 8.4 mmol, 3.0 equiv). The solution was stirred for about 10 h at room temperature until complete consumption of the substrate **5** (monitored by TLC). Then the solution was diluted with ethyl acetate (50 ml) and acidified with HCl (6 M) at 0 °C. The aqueous phase was then extracted three times with ethyl acetate. The combined organic phase was dried over anhydrous MgSO$_4$ and concentrated under reduced pressure. The residue was purified by recrystallization to obtain the desired product **6** (0.41 g, 55% yield).

8.2 Procedure for synthesis of (+)-Thiopental (9) [6, 7]

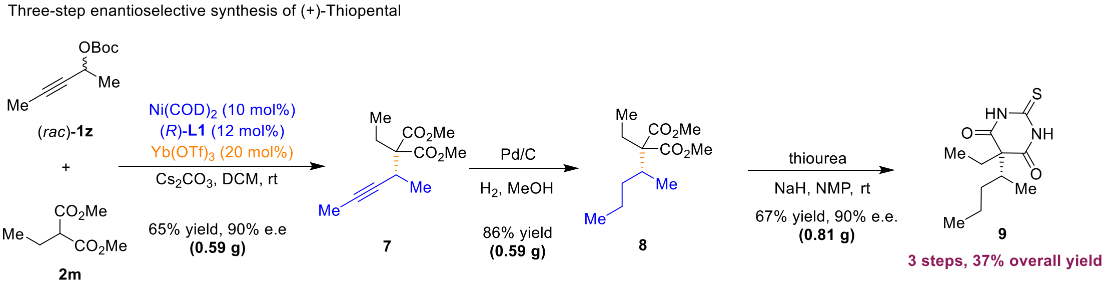

In a nitrogen-filled glove box, an oven-dried 100 mL Schlenk tube equipped with a stir bar was charged with Ni(COD)$_2$ (110.0 mg, 0.4 mmol, 10 mol%) and *R*-segphos (293 mg, 0.48 mmol, 12 mol%), and stirred in DCM (50 mL) for about 15 minutes at rt. Then 6.0 mmol **1z** (1.1 g, 1.5 equiv), 4.0 mmol **2m** (640 mg, 1.0 equiv), Yb(OTf)$_3$ (496.2 mg, 0.8 mmol, 20 mol%) and Cs$_2$CO$_3$ (2.6 g, 8.0 mmol, 2.0 equiv) were added to the tube subsequently under nitrogen atmosphere. The final solution was stirred for about 4 d at room temperature until complete consumption of the substrate **2m** (monitored by TLC). The solution was diluted with DCM

and washed with water. The aqueous phase was then extracted three times with DCM. The combined organic phase was dried over anhydrous $MgSO_4$ and concentrated under reduced pressure. The residue was purified by silica gel chromatography to afford the desired products **7** (0.59 g, 65% yield).

A solution of **7** (678.9 mg, 3.0 mmol) in MeOH (30 mL) was stirred under hydrogen atmoasphere in the presence of 10% Pd/C (68 mg) at room temperature for 12 h. The reaction mixture was then filtered through a short pad of silica gel eluting with EtOAc. The solvent was removed under reduced pressure. The residue was purified by silica gel chromatography to afford the desired products **8** (0.59 g, 86% yield).

NMP (50 ml), malonic ester **8** (1150 mg, 5.0 mmol, 1.0 equiv) and thiourea (761 mg, 10 mmol, 2.0 equiv) were stirred and treated with a 60% dispersion of sodium hydride in mineral oil (600 mg, 15 mmol, 3.0 equiv). The solution was stirred for about 10 h at room temperature until complete consumption of the substrate **8** (monitored by TLC). Then the solution was diluted with ethyl acetate (80 ml) and acidified with HCl (6 M) at 0 °C. The aqueous phase was then extracted three times with ethyl acetate. The combined organic phase was dried over anhydrous $MgSO_4$ and concentrated under reduced pressure. The residue was purified by recrystallization to obtain the desired product **9** (0.81 g, 67% yield).

8.3 Procedure for synthesis of (+)-Pentobarbital (10) [6, 7]

Three-step enantioselective synthesis of (+)-Pentobarbital

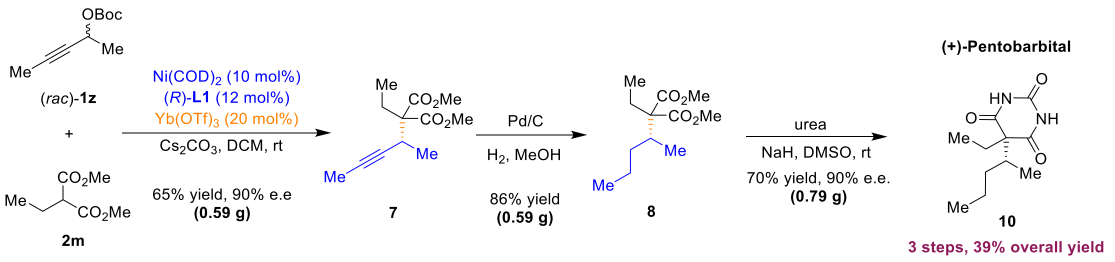

Dry dimethylsulfoxide (50 ml), malonic ester **8** (1150 mg, 5.0 mmol, 1.0 equiv) and urea (601 mg, 10 mmol, 2.0 equiv) were stirred and treated with a 60% dispersion of sodium hydride in mineral oil (600 mg, 15 mmol, 3.0 equiv). The solution was stirred for about 10 h at room temperature until complete consumption of the substrate **8** (monitored by TLC). Then the solution was diluted with ethyl acetate (80 ml) and acidified with HCl (6 M) at 0 °C. The aqueous phase was then extracted three times with ethyl acetate. The combined organic phase was dried over anhydrous $MgSO_4$ and concentrated under reduced pressure. The residue was purified by recrystallization to obtain the desired product **10** (0.79 g, 70% yield).

8.4 Procedure for synthesis of (-)-AMG 837 (11) [7]

Two-step enantioselective synthesis of (-)-AMG 837

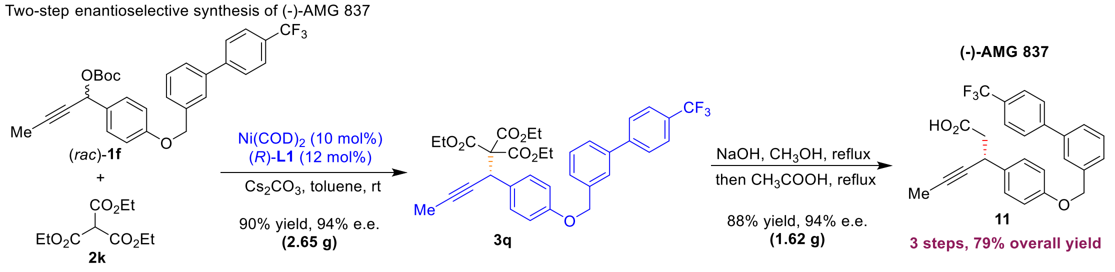

In a nitrogen-filled glove box, an oven-dried 100 mL Schlenk tube equipped with a stir bar was charged with $Ni(COD)_2$ (132 mg, 0.48 mmol, 10 mol%) and *R*-segphos (354 mg, 0.58 mmol, 12 mol%), and stirred in toluene (60 mL) for about 15 minutes at rt. Then 7.2 mmol **1f** (3.58 g, 1.5 equiv), 4.8 mmol **2k** (1115 mg, 1.0 equiv) and Cs_2CO_3 (3.1 g, 9.6 mmol, 2.0 equiv) were added to the tube subsequently under nitrogen atmosphere. The final solution was stirred for about 4 d at room temperature until complete consumption of the substrate **2k** (monitored by TLC). The solution was diluted with ethyl acetate and washed with water. The aqueous phase was then extracted three times with ethyl acetate. The combined organic phase was dried over anhydrous $MgSO_4$ and concentrated under reduced pressure. The residue was purified by silica gel chromatography to afford the desired products **3q** (2.65 g, 90% yield).

A solution of the **3q** (2550 mg, 4.18 mmol) in MeOH (40 mL) was treated with a solution 3 N NaOH (10 mL) in water, the resulting mixture was stirred at 80 °C and monitored by TLC. When no starting material remained, volatiles were removed in vacuo and the crude tris-acid was taken up in AcOH (40 mL) and heated to reflux. The reaction was monitored by TLC analysis, and when no starting material remained (12 h), solvent was concentrated in vacuo. The resultant crude acid was dissolved in water (50 mL) and extracted with EtOAc (60 mL × 3). The combined organic phase was dried over anhydrous $MgSO_4$ and concentrated under reduced pressure. The residue was purified by silica gel chromatography to afford the desired products **11** (1.62 g, 88% yield).

8.5 Procedure for synthesis of (+)-Phenoxanol (13), (+)-Citralis (14), (-)-Citralis Nitrlle (15) [7, 8 ,9]

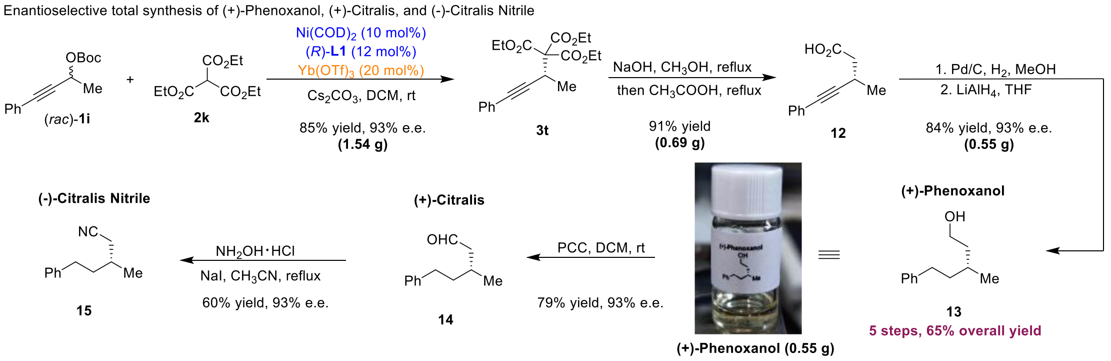

In a nitrogen-filled glove box, an oven-dried 100 mL Schlenk tube equipped with a stir bar was charged with $Ni(COD)_2$ (137.5 mg, 0.5 mmol, 10 mol%) and *R*-segphos (366.4 mg, 0.6 mmol, 12 mol%), and stirred in DCM (60 mL) for about 15 minutes at rt. Then 7.5 mmol **1i** (1.85 g, 1.5 equiv), 5.0 mmol **2k** (1160 mg, 1.0 equiv), $Yb(OTf)_3$ (620.2 mg, 1.0 mmol, 20 mol%) and Cs_2CO_3 (3.3 g, 10.0 mmol, 2.0 equiv) were added to the tube subsequently under nitrogen atmosphere. The final solution was stirred for about 4 d at room temperature until complete consumption of the substrate **2k** (monitored by TLC). The solution was diluted with DCM and washed with water. The aqueous phase was then extracted three times with DCM. The combined organic phase was dried over anhydrous $MgSO_4$ and concentrated under reduced pressure. The residue was purified by silica gel chromatography to afford the desired products **3t** (1.54 g, 85% yield).

A solution of the **3t** (1449 mg, 4.0 mmol) in MeOH (40 mL) was treated with a solution 3 N NaOH (10 mL) in water, the resulting mixture was stirred at 80 °C and monitored by TLC. When no starting material remained, volatiles were removed in vacuo and the crude tris-acid

was taken up in AcOH (40 mL) and heated to reflux. The reaction was monitored by TLC analysis, and when no starting material remained (12 h), solvent was concentrated in vacuo. The resultant crude acid was dissolved in water (50 mL) and extracted with EtOAc (60 mL × 3). The combined organic phase was dried over anhydrous $MgSO_4$ and concentrated under reduced pressure. The residue was purified by silica gel chromatography to afford the desired products **12** (0.69 g, 91% yield).

A solution of **12** (685.7 mg, 3.64 mmol) in MeOH (30 mL) was stirred under hydrogen atmoasphere in the presence of 10% Pd/C (69 mg) at room temperature for 12 h. The reaction mixture was then filtered through a short pad of silica gel eluting with EtOAc. The solvent was removed under reduced pressure. The residue was utilized in the next step without purification. To a mixture of $LiAlH_4$ (690.7 mg, 5.0 mmol) in anhydrous THF (20 mL) was added a solution of the crude product obtained from the previous step in anhydrous THF (10 mL) at 0 °C. The mixture was allowed to warm to room temperature. After stirring for 12 h, the reaction was quenched with $Na_2SO_4.10H_2O$ at 0 °C and then filtered. The filter cake was washed with ethyl acetate (10 mL × 3). The combined organic phase was dried over anhydrous $MgSO_4$ and concentrated under reduced pressure. The residue was purified by silica gel chromatography to afford the desired products **13** (0.55 g, 84% yield).

To a solution of pyridinium chlorochromate (145.3 mg, 0.674 mmol, 2.0 equiv) in DCM (2.0 ml), 3-methyl-5-phenylpentan-1-ol (60 mg, 0.337 mmol, 1.0 equiv) in DCM (0.5 ml) was added, and the solution was stirred at room temperature for 3 h. The reaction was purified by silica gel chromatography to afford the desired products **14** (47.0 mg, 79% yield).

To a solution of $NH_2OH.HCl$ (13.9 mg, 0.20 mmol, 1.3 equiv), NaI (22.5 mg, 0.15 mmol, 1.0 equiv) in MeCN (1.0 ml) was added a solution of 3-methyl-5-phenylpen-tanal (26.5 mg, 0.15 mmol) in MeCN (1.5 ml), and the reaction mixture was heated at reflux for 1 h. The reaction was monitored by TLC. The red mixture was cooled to room temperature, quenched with 5% aqueous $Na_2S_2O_3$ and stirred until complete disappearance of the red color. The solution was extracted three times with DCM. The combined organic phase was dried over anhydrous $MgSO_4$ and concentrated under reduced pressure. The residue was purified by silica gel chromatography to afford the desired products **15** (15.7 mg, 60% yield).

9. Characterization of products

Dimethyl (*R*)-2-methyl-2-(1-phenylbut-2-yn-1-yl)malonate (3a)

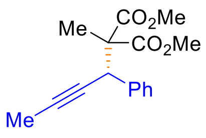

Yellow solid (31.3 mg, 76% yield). It was prepared according to the general procedure described above. **M.p.** 64-66 °C. **^{1}H NMR (500 MHz, $CDCl_3$)** δ 7.38 – 7.33 (m, 2H), 7.31 – 7.27 (m, 2H), 7.26 – 7.22 (m, 1H), 4.68 (q, *J* = 2.3 Hz, 1H), 3.78 (s, 3H), 3.59 (s, 3H), 1.85 (d, *J* = 2.5 Hz, 3H), 1.48 (s, 3H). **^{13}C NMR (125 MHz, $CDCl_3$)** δ 171.01, 170.30, 137.23, 129.60, 128.13, 127.73, 80.39, 77.47, 59.31, 53.00, 52.58, 42.79, 16.06, 3.83. **ATR-FTIR (cm^{-1}):** 2958, 2921, 2852, 2235, 1739, 1454, 1263, 1101, 1031, 800, 703. **ESI-MS:** calculated $[C_{16}H_{18}O_4 + H]^+$: 275.1278, found: 275.1280. $[\alpha]^{20}_D$ = -58.9 (c = 1.00, CH_2Cl_2). The product was analyzed by HPLC to determine the enantiomeric excess: 95% *ee* (IC, hexane/*i*-PrOH = 98/2, detector: 211 nm, flow rate: 1.0 mL/min), t_1(minor) = 13.1 min, t_2(major) = 14.3 min.

Dimethyl (*R*)-2-(1-phenylbut-2-yn-1-yl)-2-propylmalonate (3b)

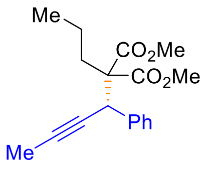

Colorless oil (24.1 mg, 53% yield). It was prepared according to the general procedure described above. **^{1}H NMR (500 MHz, $CDCl_3$)** δ 7.37 – 7.34 (m, 2H), 7.30 – 7.26 (m, 2H), 7.25 – 7.22 (m, 1H), 4.48 – 4.41 (m, 1H), 3.71 (s, 3H), 3.59 (s, 3H), 2.12 – 2.02 (m, 1H), 1.87 – 1.78 (m, 4H), 1.43 – 1.35 (m, 1H), 1.19 – 1.12 (m, 1H), 0.90 (t, *J* = 7.3 Hz, 3H). **^{13}C NMR (125 MHz, $CDCl_3$)** δ 170.59, 170.49, 137.91, 129.61, 127.99, 127.64, 80.17, 77.73, 63.01, 52.32, 52.15, 42.57, 36.11, 18.43, 14.61, 3.92. **ATR-FTIR (cm^{-1}):** 2962, 2921, 2854, 2239, 1732, 1496, 1454, 1261, 1099, 802, 701. **ESI-MS:** calculated $[C_{18}H_{22}O_4 + H]^+$: 303.1591, found: 303.1595. $[\alpha]^{20}_D$ = -48.8 (c = 0.91, CH_2Cl_2). The product was analyzed by HPLC to determine the enantiomeric excess: 96% *ee* (IC, hexane/*i*-PrOH = 99/1, detector: 211 nm, flow rate: 1.0 mL/min), t_1(minor) = 11.6 min, t_2(major) = 12.9 min.

Dimethyl (*R*)-2-isobutyl-2-(1-phenylbut-2-yn-1-yl)malonate (3c)

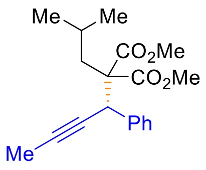

Colorless oil (24.2 mg, 51% yield). It was prepared according to the general procedure described above. **^{1}H NMR (500 MHz, $CDCl_3$)** δ 7.36 – 7.32 (m, 2H), 7.29 – 7.27 (m, 1H), 7.26 – 7.21 (m, 2H), 4.48 – 4.42 (m, 1H), 3.69 (s, 3H), 3.56 (s, 3H), 2.16 – 2.07 (m, 1H), 1.90 – 1.80 (m, 5H), 0.92 (d, *J* = 6.3 Hz, 3H), 0.84 (d, *J* = 6.2 Hz, 3H). **^{13}C NMR (125 MHz, $CDCl_3$)** δ 170.85, 170.51, 138.07, 129.67, 127.96, 127.62, 80.36, 77.89, 62.35, 52.11, 52.06, 43.22, 42.96, 24.89, 24.41, 23.35, 3.94. **ATR-FTIR (cm^{-1}):** 2956, 2923, 2854, 2238, 1732, 1454, 1230, 1128, 809, 746, 701. **ESI-MS:** calculated $[C_{19}H_{24}O_4 + H]^+$: 317.1747, found: 317.1759. $[\alpha]^{20}_D$ = -44.8 (c = 0.86, CH_2Cl_2). The product was analyzed by HPLC to determine the enantiomeric excess: 95% *ee* (AD-H, hexane/*i*-PrOH = 99/1, detector: 211 nm, flow rate: 1.0 mL/min), t_1(minor) = 9.3 min, t_2(major) = 13.4 min.

Dimethyl (*R*)-2-allyl-2-(1-phenylbut-2-yn-1-yl)malonate (3d)

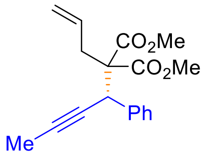

Colorless oil (24.9 mg, 55% yield). It was prepared according to the general procedure described above. **^{1}H NMR (500 MHz, $CDCl_3$)** δ 7.39 – 7.34 (m, 2H), 7.31 – 7.26 (m, 2H), 7.26 – 7.21 (m, 1H), 5.86 – 5.73 (m, 1H), 5.14 – 5.02 (m, 2H), 4.44 (q, *J* = 2.4 Hz, 1H), 3.71 (s, 3H), 3.58 (s,

3H), 2.92 – 2.60 (m, 2H), 1.85 (d, J = 2.5 Hz, 3H). **^{13}C NMR (125 MHz, $CDCl_3$)** δ 169.99, 169.86, 137.62, 133.44, 129.69, 128.01, 127.71, 118.60, 80.64, 77.56, 63.21, 52.33, 52.17, 42.38, 38.37, 3.89. **ATR-FTIR (cm^{-1}):** 3077, 2958, 2921, 2852, 2241, 1731, 1494, 1454, 1261, 1091, 917, 800, 700. **ESI-MS:** calculated [$C_{18}H_{20}O_4$ + H]$^+$: 301.1434, found: 301.1424. $[\alpha]^{20}_D$ = -41.0 (c = 0.88, CH_2Cl_2). The product was analyzed by HPLC to determine the enantiomeric excess: 96% *ee* (IC, hexane/*i*-PrOH = 99/1, detector: 211 nm, flow rate: 1.0 mL/min), t_1(minor) = 14.3 min, t_2(major) = 15.4 min.

Dimethyl (*R*)-2-benzyl-2-(1-phenylbut-2-yn-1-yl)malonate (3e)

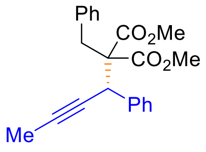

Yellow solid (38.5 mg, 73% yield). It was prepared according to the general procedure described above. **M.p.** 99-101 °C. **^{1}H NMR (500 MHz, $CDCl_3$)** δ 7.39 – 7.33 (m, 2H), 7.29 – 7.21 (m, 8H), 4.46 – 4.36 (m, 1H), 3.63 (d, J = 13.7 Hz, 1H), 3.54 (s, 3H), 3.50 (s, 3H), 3.32 (d, J = 13.7 Hz, 1H), 1.91 (d, J = 2.5 Hz, 3H). **^{13}C NMR (125 MHz, $CDCl_3$)** δ 169.78, 169.67, 137.91, 136.40, 130.21, 129.91, 128.20, 127.80, 127.59, 127.11, 81.65, 77.88, 64.48, 52.01, 51.96, 42.88, 40.47, 3.97. **ATR-FTIR (cm^{-1}):** 2950, 2921, 2854, 2241, 1732, 1496, 1454, 1265, 1085, 813, 746, 701. **ESI-MS:** calculated [$C_{22}H_{22}O_4$ + H]$^+$: 351.1591, found: 351.1592. $[\alpha]^{20}_D$ = -19.7 (c = 0.94, CH_2Cl_2). The product was analyzed by HPLC to determine the enantiomeric excess: 95% *ee* (AD-H, hexane/*i*-PrOH = 95/5, detector: 222 nm, flow rate: 1.0 mL/min), t_1(minor) = 8.1 min, t_2(major) = 9.2 min.

Trimethyl (*R*)-3-phenylhex-4-yne-1,2,2-tricarboxylate (3f)

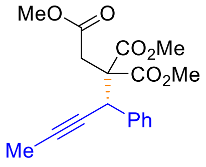

Colorless oil (48.3 mg, 97% yield). It was prepared according to the general procedure described above. **^{1}H NMR (500 MHz, $CDCl_3$)** δ 7.41 – 7.34 (m, 2H), 7.31 – 7.25 (m, 3H), 4.67 – 4.58 (m, 1H), 3.80 (s, 3H), 3.59 (s, 3H), 3.56 (s, 3H), 3.19 (d, J = 17.0 Hz, 1H), 2.96 (d, J = 17.0 Hz, 1H), 1.84 (d, J = 2.4 Hz, 3H). **^{13}C NMR (125 MHz, $CDCl_3$)** δ 171.11, 169.58, 169.06, 136.61, 129.67, 128.12, 127.97, 81.46, 76.54, 60.58, 53.09, 52.57, 51.83, 42.70, 36.42, 3.78. **ATR-FTIR (cm^{-1}):** 2958, 2923, 2854, 2240, 1741, 1456, 1261, 1095, 1020, 879, 800, 701. **ESI-MS:** calculated [$C_{18}H_{20}O_6$ + H]$^+$: 333.1333, found: 333.1331. $[\alpha]^{20}_D$ = -38.4 (c = 0.97, CH_2Cl_2). The product was analyzed by HPLC to determine the enantiomeric excess: 95% *ee* (OD-H, hexane/*i*-PrOH = 97/3, detector: 211 nm, flow rate: 1.0 mL/min), t_1(minor) = 13.7 min, t_2(major) = 20.4 min.

Diethyl (*R*)-2-(2-cyanoethyl)-2-(1-phenylbut-2-yn-1-yl)malonate (3g)

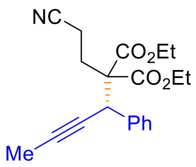

Colorless oil (47.5 mg, 93% yield). It was prepared according to the general procedure described above. **^{1}H NMR (500 MHz, $CDCl_3$)** δ 7.38 – 7.33 (m, 2H), 7.32 – 7.27 (m, 3H), 4.58 (q, J = 2.4 Hz, 1H), 4.34 – 4.19 (m, 2H), 4.14 – 4.00 (m, 2H), 2.74 – 2.62 (m, 1H), 2.53 – 2.39 (m, 2H), 2.31 – 2.21 (m, 1H), 1.86 (d, J = 2.6 Hz, 3H), 1.30 (t, J = 7.1 Hz, 3H), 1.16 (t, J = 7.1 Hz, 3H). **^{13}C NMR (125 MHz, $CDCl_3$)** δ 168.90, 168.46, 136.54, 129.56, 128.33, 128.09, 119.85, 81.53, 76.80, 62.11, 61.93, 61.52, 43.04, 27.96, 14.07, 13.89, 3.81. **ATR-FTIR (cm^{-1}):** 2960, 2923, 2854, 2248, 1728, 1454, 1367, 1261, 1093, 863, 800, 702. **ESI-MS:** calculated [$C_{20}H_{23}NO_4$ + H]$^+$: 342.1700, found: 342.1700. $[\alpha]^{20}_D$ = -28.8 (c = 1.05, CH_2Cl_2). The product

was analyzed by HPLC to determine the enantiomeric excess: 94% *ee* (OD-H, hexane/*i*-PrOH = 97/3, detector: 211 nm, flow rate: 1.0 mL/min), t_1(major) = 12.2 min, t_2(minor) = 13.6 min.

1,1-di-tert-butyl 1-ethyl (*R*)-2-phenylpent-3-yne-1,1,1-tricarboxylate (3h)

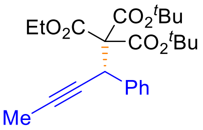

Colorless oil (47.5 mg, 76% yield). Prepared according to the above general procedure, but toluene is used as the reaction solvent. **^{1}H NMR (500 MHz, CDCl$_3$)** δ 7.65 – 7.59 (m, 2H), 7.26 – 7.19 (m, 3H), 4.65 (q, *J* = 2.4 Hz, 1H), 4.19 – 4.05 (m, 2H), 1.82 (d, *J* = 2.5 Hz, 3H), 1.41 (s, 9H), 1.39 (s, 9H), 1.20 (t, *J* = 7.2 Hz, 3H). **^{13}C NMR (125 MHz, CDCl$_3$)** δ 166.02, 164.51, 164.40, 137.44, 130.65, 127.77, 127.57, 82.79, 80.01, 77.80, 70.90, 61.68, 41.01, 27.78, 27.77, 14.00, 3.99. **ATR-FTIR (cm^{-1}):** 2979, 2929, 2856, 2244, 1743, 1496, 1454, 1369, 1257, 1151, 1062, 846, 700. **ESI-MS:** calculated [$C_{24}H_{32}O_6$ + Na]$^+$: 439.2091, found: 439.2095. $[\alpha]^{20}_D$ = -16.2 (c = 0.91, CH_2Cl_2). The product was analyzed by HPLC to determine the enantiomeric excess: 95% *ee* (IC, hexane/*i*-PrOH = 99/1, detector: 211 nm, flow rate: 1.0 mL/min), t_1(minor) = 14.4 min, t_2(major) = 18.7 min.

1,1-dibenzyl 1-methyl (*R*)-2-phenylpent-3-yne-1,1,1-tricarboxylate (3i)

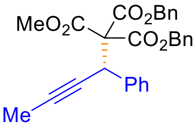

Colorless oil (56.9 mg, 81% yield). Prepared according to the above general procedure, but toluene is used as the reaction solvent. **^{1}H NMR (500 MHz, CDCl$_3$)** δ 7.53 – 7.48 (m, 2H), 7.34 – 7.28 (m, 6H), 7.25 – 7.17 (m, 7H), 5.17 – 5.04 (m, 4H), 4.81 (q, *J* = 2.3 Hz, 1H), 3.61 (s, 3H), 1.74 (d, *J* = 2.5 Hz, 3H). **^{13}C NMR (125 MHz, CDCl$_3$)** δ 165.57, 165.04, 164.97, 136.41, 134.96, 130.32, 128.56, 128.42, 128.40, 128.32, 128.29, 128.00, 127.91, 80.92, 77.87, 70.60, 67.82, 67.80, 52.91, 41.37, 3.77. **ATR-FTIR (cm^{-1}):** 2958, 2920, 2852, 2242, 1743, 1498, 1454, 1375, 1261, 1054, 800, 738, 696. **ESI-MS:** calculated [$C_{29}H_{26}O_6$ + H]$^+$: 471.1802, found: 471.1800. $[\alpha]^{20}_D$ = -14.8 (c = 1.09, CH_2Cl_2). The product was analyzed by HPLC to determine the enantiomeric excess: 95% *ee* (AD-H, hexane/*i*-PrOH = 80/20, detector: 211 nm, flow rate: 1.0 mL/min), t_1(major) = 9.5 min, t_2(minor) = 11.0 min.

Trimethyl (*R*)-2-phenylpent-3-yne-1,1,1-tricarboxylate (3j)

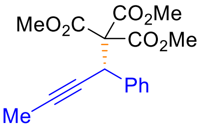

White soild (46.2 mg, 97% yield). Prepared according to the above general procedure, but toluene is used as the reaction solvent. **M.p.** 78-80 °C. **^{1}H NMR (500 MHz, CDCl$_3$)** δ 7.56 – 7.50 (m, 2H), 7.31 – 7.27 (m, 2H), 7.26 – 7.23 (m, 1H), 4.77 (q, *J* = 2.4 Hz, 1H), 3.71 (s, 9H), 1.84 (d, *J* = 2.5 Hz, 3H). **^{13}C NMR (125 MHz, CDCl$_3$)** δ 165.76, 136.43, 130.21, 128.04, 127.91, 80.71, 76.76, 70.37, 53.11, 41.37, 3.86. **ATR-FTIR (cm^{-1}):** 2960, 2921, 2852, 2244, 1747, 1496, 1454, 1261, 1080, 800, 700. **ESI-MS:** calculated [$C_{17}H_{18}O_6$ + H]$^+$: 319.1176, found: 319.1185. $[\alpha]^{20}_D$ = -45.0 (c = 1.02, CH_2Cl_2). The product was analyzed by HPLC to determine the enantiomeric excess: 93% *ee* (IC, hexane/*i*-PrOH = 95/5, detector: 211 nm, flow rate: 1.0 mL/min), t_1(minor) = 16.9 min, t_2(major) = 18.4 min.

Triethyl (*R*)-2-phenylpent-3-yne-1,1,1-tricarboxylate (3k)

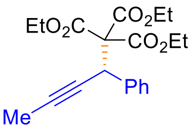

Colorless oil (51.0 mg, 94% yield). Prepared according to the above general procedure, but toluene is used as the reaction solvent. **^{1}H NMR**

(500 MHz, CDCl$_3$) δ 7.59 – 7.53 (m, 2H), 7.27 – 7.21 (m, 3H), 4.79 – 4.73 (m, 1H), 4.23 – 4.11 (m, 6H), 1.83 (d, *J* = 2.5 Hz, 3H), 1.20 (t, *J* = 7.1 Hz, 9H). **^{13}C NMR (125 MHz, CDCl$_3$)** δ 165.35, 136.77, 130.37, 127.86, 127.80, 80.44, 77.09, 70.18, 62.08, 41.17, 13.90, 3.86. **ATR-FTIR (cm^{-1}):** 2962, 2923, 2852, 2244, 1745, 1496, 1454, 1367, 1261, 1093, 863, 700. **ESI-MS:** calculated [$C_{20}H_{24}O_6$ + H]$^+$: 361.1646, found: 361.1651. $[\alpha]^{20}_D$ = -33.9 (c = 0.97, CH_2Cl_2). The product was analyzed by HPLC to determine the enantiomeric excess: 94% *ee* (IC, hexane/*i*-PrOH = 97/3, detector: 211 nm, flow rate: 1.0 mL/min), t_1(minor) = 17.0 min, t_2(major) = 18.6 min.

Tribenzyl (*R*)-2-phenylpent-3-yne-1,1,1-tricarboxylate (3l)

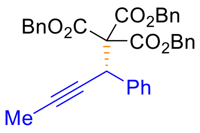

Colorless oil (74.0 mg, 90% yield). Prepared according to the above general procedure, but toluene is used as the reaction solvent. **^{1}H NMR (500 MHz, CDCl$_3$)** δ 7.53 – 7.46 (m, 2H), 7.33 – 7.25 (m, 8H), 7.24 – 7.22 (m, 1H), 7.22 – 7.17 (m, 3H), 7.17 – 7.10 (m, 6H), 5.11 – 4.97 (m, 6H), 4.82 (q, *J* = 2.3 Hz, 1H), 1.69 (d, *J* = 2.5 Hz, 3H). **^{13}C NMR (125 MHz, CDCl$_3$)** δ 164.94, 136.36, 134.87, 130.39, 128.55, 128.39, 128.34, 128.01, 127.93, 80.97, 76.88, 70.54, 67.84, 41.39, 3.78. **ATR-FTIR (cm^{-1}):** 3066, 3033, 2962, 2919, 2852, 2243, 1743, 1498, 1454, 1375, 1261, 1051, 800, 696. **ESI-MS:** calculated [$C_{35}H_{30}O_6$ + H]$^+$: 547.2115, found: 547.2127. $[\alpha]^{20}_D$ = -10.8 (c = 1.02, CH_2Cl_2). The product was analyzed by HPLC to determine the enantiomeric excess: 95% *ee* (AD-H, hexane/*i*-PrOH = 80/20, detector: 211 nm, flow rate: 1.0 mL/min), t_1(major) = 13.1 min, t_2(minor) = 16.6 min.

Triethyl (*R*)-2-(4-fluorophenyl)pent-3-yne-1,1,1-tricarboxylate (3m)

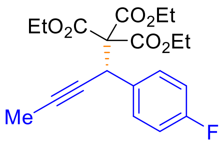

Colorless oil (54.0 mg, 95% yield). Prepared according to the above general procedure, but toluene is used as the reaction solvent. **^{1}H NMR (500 MHz, CDCl$_3$)** δ 7.59 – 7.51 (m, 2H), 7.01 – 6.90 (m, 2H), 4.74 (q, *J* = 2.3 Hz, 1H), 4.26 – 4.09 (m, 6H), 1.83 (d, *J* = 2.5 Hz, 3H), 1.21 (t, *J* = 7.1 Hz, 9H). **^{19}F NMR (470 MHz, CDCl$_3$)** δ -114.8 (s). **^{13}C NMR (125 MHz, CDCl$_3$)** δ 165.32, 162.45 (d, *J* = 246.5 Hz), 132.52 (d, *J* = 3.0 Hz), 132.21 (d, *J* = 8.2 Hz), 114.62 (d, *J* = 21.2 Hz), 80.74, 76.94, 70.18, 62.20, 40.43, 13.95, 3.86. **ATR-FTIR (cm^{-1}):** 2962, 2923, 2854, 2242, 1745, 1508, 1463, 1259, 1222, 1066, 800. **ESI-MS:** calculated [$C_{20}H_{23}FO_6$ + H]$^+$: 379.1551, found: 379.1558. $[\alpha]^{20}_D$ = -31.6 (c = 1.00, CH_2Cl_2). The product was analyzed by HPLC to determine the enantiomeric excess: 95% *ee* (IC, hexane/*i*-PrOH = 97/3, detector: 211 nm, flow rate: 1.0 mL/min), t_1(minor) = 11.8 min, t_2(major) = 13.1 min.

Triethyl (*R*)-2-(4-chlorophenyl)pent-3-yne-1,1,1-tricarboxylate (3n)

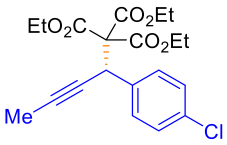

Colorless oil (51.1 mg, 87% yield). Prepared according to the above general procedure, but toluene is used as the reaction solvent. **^{1}H NMR (500 MHz, CDCl$_3$)** δ 7.54 – 7.49 (m, 2H), 7.26 – 7.21 (m, 2H), 4.72 (q, *J* = 2.4 Hz, 1H), 4.25 – 4.11 (m, 6H), 1.83 (d, *J* = 2.5 Hz, 3H), 1.21 (t, *J* = 7.1 Hz, 9H). **^{13}C NMR (125 MHz, CDCl$_3$)** δ 165.27, 135.35, 133.81, 131.89, 127.95, 80.86, 76.69, 70.08, 62.26, 40.55, 13.95, 3.86. **ATR-FTIR (cm^{-1}):** 2962, 2923, 2854, 2242, 1743, 1490, 1444, 1261, 1062, 863, 800, 703. **ESI-MS:** calculated [$C_{20}H_{23}ClO_6$ + H]$^+$: 395.1256, found: 395.1267. $[\alpha]^{20}_D$ = -25.0 (c = 0.96, CH_2Cl_2). The product was analyzed by HPLC to

determine the enantiomeric excess: 95% *ee* (IC, hexane/*i*-PrOH = 97/3, detector: 211 nm, flow rate: 1.0 mL/min), t_1(minor) = 11.1 min, t_2(major) = 12.9 min.

Triethyl (*R*)-2-(p-tolyl)pent-3-yne-1,1,1-tricarboxylate (3o)

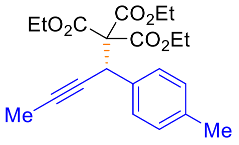

Colorless oil (49.0 mg, 87% yield). Prepared according to the above general procedure, but toluene is used as the reaction solvent. **^{1}H NMR (500 MHz, $CDCl_3$)** δ 7.43 (d, *J* = 8.1 Hz, 2H), 7.07 (d, *J* = 8.0 Hz, 2H), 4.71 (q, *J* = 2.3 Hz, 1H), 4.28 – 4.09 (m, 6H), 2.30 (s, 3H), 1.82 (d, *J* = 2.5 Hz, 3H), 1.21 (t, *J* = 7.1 Hz, 9H). **^{13}C NMR (125 MHz, $CDCl_3$)** δ 165.42, 137.53, 133.72, 130.21, 128.54, 80.23, 77.25, 70.18, 62.06, 40.87, 21.18, 13.93, 3.88. **ATR-FTIR (cm^{-1}):** 2960, 2923, 2854, 2242, 1745, 1513, 1463, 1259, 1024, 862, 800. **ESI-MS:** calculated $[C_{21}H_{26}O_6 + H]^+$: 375.1802, found: 375.1807. $[\alpha]^{20}_D$ = -24.4 (c = 0.94, CH_2Cl_2). The product was analyzed by HPLC to determine the enantiomeric excess: 95% *ee* (IC, hexane/*i*-PrOH = 97/3, detector: 220 nm, flow rate: 1.0 mL/min), t_1(minor) = 17.8 min, t_2(major) = 23.5 min.

Triethyl (*R*)-2-(4-methoxyphenyl)pent-3-yne-1,1,1-tricarboxylate (3p)

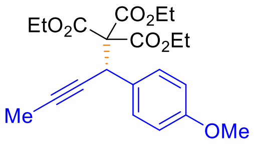

Colorless oil (56.0 mg, 96% yield). It was prepared according to the general procedure above without Lewis acid, and toluene was used as the reaction solvent. **^{1}H NMR (500 MHz, $CDCl_3$)** δ 7.54 – 7.43 (m, 2H), 6.85 – 6.74 (m, 2H), 4.74 – 4.66 (m, 1H), 4.24 – 4.12 (m, 6H), 3.77 (s, 3H), 1.82 (d, *J* = 2.4 Hz, 3H), 1.21 (t, *J* = 7.1 Hz, 9H). **^{13}C NMR (125 MHz, $CDCl_3$)** δ 165.42, 159.16, 131.51, 128.71, 113.11, 80.22, 77.32, 70.25, 62.04, 55.28, 40.47, 13.93, 3.84. **ATR-FTIR (cm^{-1}):** 2962, 2921, 2852, 2240, 1743, 1511, 1463, 1365, 1261, 1054, 862, 800. **ESI-MS:** calculated $[C_{21}H_{26}O_7 + H]^+$: 391.1751, found: 391.1741. $[\alpha]^{20}_D$ = -26.3 (c = 1.14, CH_2Cl_2). The product was analyzed by HPLC to determine the enantiomeric excess: 95% *ee* (IC, hexane/*i*-PrOH = 90/10, detector: 230 nm, flow rate: 1.0 mL/min), t_1(minor) = 12.4 min, t_2(major) = 14.8 min.

Triethyl (*R*)-2- (4-((4'-(trifluoromethyl)-[1,1'-biphenyl]-3-yl)methoxy)phenyl)pent-3-yne -1,1,1-tricarboxylate (3q)

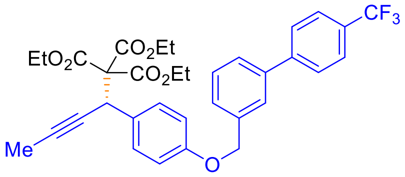

Colorless oil (86.0 mg, 94% yield). It was prepared according to the general procedure above without Lewis acid, and toluene was used as the reaction solvent. **^{1}H NMR (400 MHz, $CDCl_3$)** δ 7.73 – 7.67 (m, 4H), 7.66 – 7.62 (m, 1H), 7.57 – 7.42 (m, 5H), 6.93 – 6.86 (m, 2H), 5.11 (s, 2H), 4.71 (q, *J* = 2.3 Hz, 1H), 4.24 – 4.09 (m, 6H), 1.82 (d, *J* = 2.5 Hz, 3H), 1.20 (t, *J* = 7.1 Hz, 9H). **^{19}F NMR (375 MHz, $CDCl_3$)** δ -62.41 (s). **^{13}C NMR (100 MHz, $CDCl_3$)** δ 165.45, 158.34, 144.55, 140.27, 138.05, 131.68, 129.62 (q, *J* = 32.0 Hz), 129.38, 129.30, 127.62, 127.34, 127.00, 126.44, 125.86 (q, *J* = 4.0 Hz), 124.41 (q, *J* = 271.0 Hz), 114.13, 80.35, 77.35, 70.34, 69.88, 62.08, 40.55, 13.96, 3.87. **ATR-FTIR (cm^{-1}):** 2983, 2923, 2243, 1747, 1058, 1465, 1367, 1243, 1126, 1072, 844, 790, 700. **ESI-MS:** calculated $[C_{34}H_{33}F_3O_7 + H]^+$: 611.2251, found: 611.2234. $[\alpha]^{20}_D$ = -17.3 (c = 1.00, CH_2Cl_2). The product was analyzed by HPLC to determine the enantiomeric excess: 94% *ee* (IC, hexane/*i*-PrOH = 95/5, detector: 254 nm, flow rate: 1.0 mL/min), t_1(minor) = 19.8 min, t_2(major) = 23.8 min.

Diethyl (*R*)-2-(1-(3-chlorophenyl)but-2-yn-1-yl)-2-methylmalonate (3r)

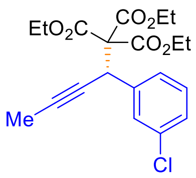

Colorless oil (42.2 mg, 72% yield). Prepared according to the above general procedure, but toluene is used as the reaction solvent. **^{1}H NMR (400 MHz, CDCl$_3$)** δ 7.57 (s, 1H), 7.47 (d, *J* = 6.9 Hz, 1H), 7.28 – 7.18 (m, 2H), 4.75 – 4.67 (m, 1H), 4.27 – 4.12 (m, 6H), 1.83 (s, 3H), 1.22 (t, *J* = 7.1 Hz, 9H). **^{13}C NMR (100 MHz, CDCl$_3$)** δ 165.20, 138.80, 133.52, 130.58, 128.98, 128.76, 127.99, 81.02, 76.44, 70.16, 62.26, 40.73, 13.89, 3.81. **ATR-FTIR (cm^{-1}):** 2964, 2923, 2852, 2246, 1745, 1469, 1367, 1259, 1051, 862, 798, 692. **ESI-MS:** calculated [$C_{20}H_{23}ClO_6$ + H]$^+$: 395.1256, found: 395.1267. $[\alpha]^{20}_D$ = -26.9 (c = 0.96, CH_2Cl_2). The product was analyzed by HPLC to determine the enantiomeric excess: 93% *ee* (IC, hexane/*i*-PrOH = 97/3, detector: 211 nm, flow rate: 1.0 mL/min), t_1(minor) = 11.8 min, t_2(major) = 17.8 min.

Triethyl (*R*)-2-(3-bromophenyl)pent-3-yne-1,1,1-tricarboxylate (3s)

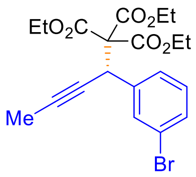

Colorless oil (34.6 mg, 52% yield). Prepared according to the above general procedure, but toluene is used as the reaction solvent. **^{1}H NMR (400 MHz, CDCl$_3$)** δ 7.70 (s, 1H), 7.52 (d, *J* = 7.8 Hz, 1H), 7.37 (d, *J* = 7.9 Hz, 1H), 7.14 (t, *J* = 7.8 Hz, 1H), 4.70 (s, 1H), 4.27 – 4.08 (m, 6H), 1.83 (s, 3H), 1.22 (t, *J* = 7.1 Hz, 9H). **^{13}C NMR (101 MHz, CDCl$_3$)** δ 165.24, 139.12, 133.47, 130.96, 129.34, 129.29, 121.74, 81.10, 76.46 70.22, 62.31, 40.74, 13.95, 3.87. **ATR-FTIR (cm^{-1}):** 2960, 2923, 2854, 2246, 1743, 1567, 1465, 1259, 1068, 863, 798, 692, 603. **ESI-MS:** calculated [$C_{20}H_{23}BrO_6$ + H]$^+$: 439.0751, found: 439.0755. $[\alpha]^{20}_D$ = -22.8 (c = 1.02, CH_2Cl_2). The product was analyzed by HPLC to determine the enantiomeric excess: 95% *ee* (IC, hexane/*i*-PrOH = 97/3, detector: 211 nm, flow rate: 1.0 mL/min), t_1(minor) = 12.3 min, t_2(major) = 19.7 min.

Triethyl (*R*)-2-methyl-4-phenylbut-3-yne-1,1,1-tricarboxylate (3t)

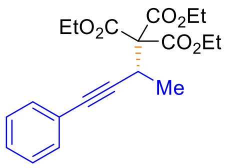

Colorless oil (52.7 mg, 97% yield). It was prepared according to the general procedure described above. **^{1}H NMR (500 MHz, CDCl$_3$)** δ 7.39 – 7.33 (m, 2H), 7.29 – 7.24 (m, 3H), 4.29 (q, *J* = 7.1 Hz, 6H), 3.68 (q, *J* = 7.0 Hz, 1H), 1.50 (d, *J* = 7.0 Hz, 3H), 1.29 (t, *J* = 7.1 Hz, 9H). **^{13}C NMR (125 MHz, CDCl$_3$)** δ 165.85, 131.67, 128.22, 127.93, 123.53, 89.84, 82.41, 69.08, 62.26, 30.88, 17.68, 14.09. **ATR-FTIR (cm^{-1}):** 2981, 2923, 2854, 2242, 1743, 1598, 1463, 1367, 1261, 1095, 862, 757, 692. **ESI-MS:** calculated [$C_{20}H_{24}O_6$ + H]$^+$: 361.1646, found: 361.1650. $[\alpha]^{20}_D$ = -31.2 (c = 1.11, CH_2Cl_2). The product was analyzed by HPLC to determine the enantiomeric excess: 93% *ee* (OD-H, hexane/*i*-PrOH = 99/1, detector: 254 nm, flow rate: 1.0 mL/min), t_1(minor) = 11.1 min, t_2(major) = 12.8 min.

Triethyl (*R*)-4-(4-chlorophenyl)-2-methylbut-3-yne-1,1,1-tricarboxylate (3u)

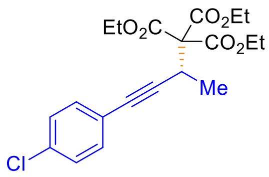

Colorless oil (56.9 mg, 96% yield). It was prepared according to the general procedure described above. **^{1}H NMR (500 MHz, CDCl$_3$)** δ 7.30 – 7.27 (m, 2H), 7.25 – 7.21 (m, 2H), 4.29 (q, *J* = 7.1 Hz, 6H), 3.66 (q, *J* = 7.0 Hz, 1H), 1.49 (d, *J* = 7.0 Hz, 3H), 1.29 (t, *J* = 7.1 Hz, 9H). **^{13}C NMR (125 MHz, CDCl$_3$)** δ 165.80, 133.93, 132.93, 128.59,

122.02, 90.97, 81.38, 68.99, 62.33, 30.91, 17.59, 14.11. **ATR-FTIR (cm^{-1}):** 2962, 2923, 2852, 2244, 1743, 1488, 1367, 1261, 1091, 862, 800, 696. **ESI-MS:** calculated $[C_{20}H_{23}ClO_6 + H]^+$: 395.1256, found: 395.1267. $[\alpha]^{20}_D$ = -31.3 (c = 0.98, CH_2Cl_2). The product was analyzed by HPLC to determine the enantiomeric excess: 92% *ee* (OJ-H, hexane/*i*-PrOH = 98/2, detector: 254 nm, flow rate: 1.0 mL/min), t_1(minor) = 13.5 min, t_2(major) = 20.1 min.

Triethyl (*R*)-4-(4-bromophenyl)-2-methylbut-3-yne-1,1,1-tricarboxylate (3v)

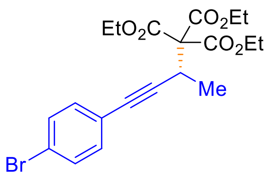

Colorless oil (62.6 mg, 95% yield). It was prepared according to the general procedure described above. **^{1}H NMR (500 MHz, $CDCl_3$)** δ 7.43 – 7.37 (m, 2H), 7.24 – 7.19 (m, 2H), 4.29 (q, *J* = 7.1 Hz, 6H), 3.65 (q, *J* = 7.0 Hz, 1H), 1.49 (d, *J* = 7.0 Hz, 3H), 1.29 (t, *J* = 7.1 Hz, 9H). **^{13}C NMR (125 MHz, $CDCl_3$)** δ 165.79, 133.15, 131.51, 122.48, 122.12, 91.18, 81.43, 68.96, 62.32, 30.92, 17.56, 14.11. **ATR-FTIR (cm^{-1}):** 2983, 2937, 2854, 2242, 1743, 1486, 1367, 1261, 1078, 863, 825, 522. **ESI-MS:** calculated $[C_{20}H_{23}BrO_6 + H]^+$: 439.0751, found: 439.0755. $[\alpha]^{20}_D$ = -31.1 (c = 1.01, CH_2Cl_2). The product was analyzed by HPLC to determine the enantiomeric excess: 93% *ee* (OJ-H, hexane/*i*-PrOH = 98/2, detector: 254 nm, flow rate: 1.0 mL/min), t_1(minor) = 12.6 min, t_2(major) = 17.9 min.

Triethyl (*R*)-2-methyl-4-(4-(trifluoromethyl)phenyl)but-3-yne-1,1,1-tricarboxylate (3w)

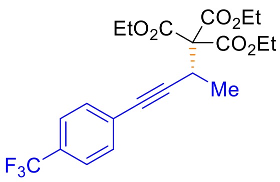

Colorless oil (51.4 mg, 80% yield). Prepared according to the above general procedure, but toluene is used as the reaction solvent. **^{1}H NMR (500 MHz, $CDCl_3$)** δ 7.52 (d, *J* = 8.3 Hz, 2H), 7.45 (d, *J* = 8.2 Hz, 2H), 4.29 (q, *J* = 7.1 Hz, 6H), 3.68 (q, *J* = 7.0 Hz, 1H), 1.50 (d, *J* = 7.0 Hz, 3H), 1.29 (t, *J* = 7.1 Hz, 9H). **^{19}F NMR (470 MHz, $CDCl_3$)** δ -62.80 (s). **^{13}C NMR (125 MHz, $CDCl_3$)** δ 165.75, 131.92, 129.75 (q, *J* = 31.3 Hz), 127.36, 125.22 (q, *J* = 3.9 Hz), 124.08 (q, *J* = 271.3 Hz), 92.73, 81.28, 68.95, 62.38, 30.91, 17.50, 14.09. **ATR-FTIR (cm^{-1}):** 2985, 2940, 2244, 1745, 1467, 1371, 1324, 1228, 1128, 844. **ESI-MS:** calculated $[C_{21}H_{23}F_3O_6 + Na]^+$: 451.1339, found: 451.1352. $[\alpha]^{20}_D$ = -26.2 (c = 1.13, CH_2Cl_2). The product was analyzed by HPLC to determine the enantiomeric excess: 94% *ee* (IC, hexane/*i*-PrOH = 98/2, detector: 254 nm, flow rate: 1.0 mL/min), t_1(major) = 11.0 min, t_2(minor) = 12.7 min.

Triethyl (*R*)-4-(4-(ethoxycarbonyl)phenyl)-2-methylbut-3-yne-1,1,1-tricarboxylate (3x)

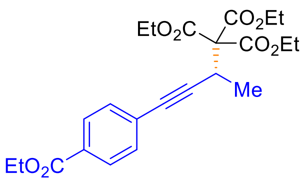

Colorless oil (62.2 mg, 96% yield). Prepared according to the above general procedure, but acetonitrile is used as the reaction solvent. **^{1}H NMR (500 MHz, $CDCl_3$)** δ 7.93 (d, *J* = 8.3 Hz, 2H), 7.40 (d, *J* = 8.3 Hz, 2H), 4.35 (q, *J* = 7.1 Hz, 2H), 4.28 (q, *J* = 7.1 Hz, 6H), 3.68 (q, *J* = 7.0 Hz, 1H), 1.49 (d, *J* = 7.0 Hz, 3H), 1.37 (t, *J* = 7.1 Hz, 3H), 1.28 (t, *J* = 7.1 Hz, 9H). **^{13}C NMR (125 MHz, $CDCl_3$)** δ 166.24, 165.76, 131.56, 129.65, 129.42, 128.16, 93.15, 81.90, 68.97, 62.35, 61.19, 30.96, 17.53, 14.41, 14.08. **ATR-FTIR (cm^{-1}):** 2983, 2939, 2242, 1743, 1606, 1465, 1367, 1272, 1097, 860, 771, 698. **ESI-MS:** calculated $[C_{23}H_{28}O_8 + Na]^+$: 455.1676, found: 455.1659. $[\alpha]^{20}_D$ = -34.5 (c = 1.05, CH_2Cl_2). The product was analyzed by HPLC to determine the enantiomeric excess: 96% *ee*

(IC, hexane/*i*-PrOH = 95/5, detector: 254 nm, flow rate: 1.0 mL/min), t_1(major) = 19.3 min, t_2(minor) = 21.6 min.

Triethyl (*R*)-4-(4-cyanophenyl)-2-methylbut-3-yne-1,1,1-tricarboxylate (3y)

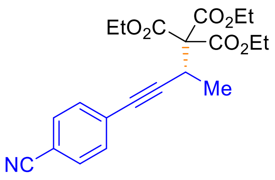

Colorless oil (56.1 mg, 97% yield). Prepared according to the above general procedure, but acetonitrile is used as the reaction solvent. **^{1}H NMR (500 MHz, $CDCl_3$)** δ 7.55 (d, *J* = 8.3 Hz, 2H), 7.42 (d, *J* = 8.3 Hz, 2H), 4.28 (q, *J* = 7.1 Hz, 6H), 3.67 (q, *J* = 7.0 Hz, 1H), 1.49 (d, *J* = 7.0 Hz, 3H), 1.28 (t, *J* = 7.1 Hz, 9H). **^{13}C NMR (125 MHz, $CDCl_3$)** δ 165.64, 132.20, 132.01, 128.46, 118.63, 111.33, 94.93, 81.10, 68.82, 62.39, 30.94, 17.38, 14.07. **ATR-FTIR (cm^{-1}):** 2985, 2917, 2850, 2227, 1743, 1604, 1502, 1465, 1230, 1097, 842. **ESI-MS:** calculated $[C_{21}H_{23}NO_6 + H]^+$: 386.1598, found: 386.1611. $[\alpha]^{20}_D$ = -37.2 (c = 1.09, CH_2Cl_2). The product was analyzed by HPLC to determine the enantiomeric excess: 96% *ee* (OD-H, hexane/*i*-PrOH = 95/5, detector: 254 nm, flow rate: 1.0 mL/min), t_1(minor) = 9.4 min, t_2(major) = 10.9 min.

Triethyl (*R*)-2-methyl-4-(p-tolyl)but-3-yne-1,1,1-tricarboxylate (3z)

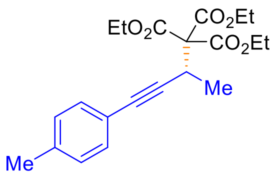

Colorless oil (54.7 mg, 97% yield). It was prepared according to the general procedure described above. **^{1}H NMR (500 MHz, $CDCl_3$)** δ 7.25 (d, *J* = 8.1 Hz, 2H), 7.07 (d, *J* = 7.9 Hz, 2H), 4.29 (q, *J* = 7.1 Hz, 6H), 3.67 (q, *J* = 7.0 Hz, 1H), 2.32 (s, 3H), 1.49 (d, *J* = 7.0 Hz, 3H), 1.29 (t, *J* = 7.1 Hz, 9H). **^{13}C NMR (125 MHz, $CDCl_3$)** δ 165.89, 137.92, 131.56, 128.97, 120.47, 89.03, 82.47, 69.13, 62.24, 30.90, 21.54, 17.73, 14.09. **ATR-FTIR (cm^{-1}):** 2962, 2923, 2856, 2238, 1743, 1510, 1446, 1367, 1259, 1095, 862, 815. **ESI-MS:** calculated $[C_{21}H_{26}O_6 + H]^+$: 375.1802, found: 375.1809. $[\alpha]^{20}_D$ = -33.3 (c = 1.11, CH_2Cl_2). The product was analyzed by HPLC to determine the enantiomeric excess: 95% *ee* (OD-H, hexane/*i*-PrOH = 99/1, detector: 254 nm, flow rate: 1.0 mL/min), t_1(minor) = 11.3 min, t_2(major) = 12.7 min.

Triethyl (*R*)-4-(4-methoxyphenyl)-2-methylbut-3-yne-1,1,1-tricarboxylate (3aa)

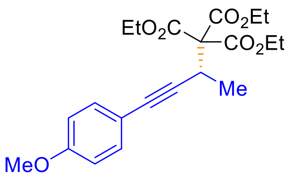

Colorless oil (57.3 mg, 98% yield). It was prepared according to the general procedure described above. **^{1}H NMR (500 MHz, $CDCl_3$)** δ 7.33 – 7.27 (m, 2H), 6.84 – 6.75 (m, 2H), 4.29 (q, *J* = 7.1 Hz, 6H), 3.79 (s, 3H), 3.66 (q, *J* = 7.0 Hz, 1H), 1.48 (d, *J* = 7.0 Hz, 3H), 1.29 (t, *J* = 7.1 Hz, 9H). **^{13}C NMR (125 MHz, $CDCl_3$)** δ 165.91, 159.33, 133.07, 115.72, 113.83, 88.27, 82.22, 69.16, 62.23, 55.36, 30.93, 17.78, 14.11. **ATR-FTIR (cm^{-1}):** 2964, 2923, 2852, 2240, 1743, 1511, 1465, 1367, 1259, 1095, 862, 798. **ESI-MS:** calculated $[C_{21}H_{26}O_7 + H]^+$: 391.1751, found: 391.1741. $[\alpha]^{20}_D$ = -35.3 (c = 1.00, CH_2Cl_2). The product was analyzed by HPLC to determine the enantiomeric excess: 97% *ee* (OD-H, hexane/*i*-PrOH = 99/1, detector: 254 nm, flow rate: 1.0 mL/min), t_1(minor) = 19.5 min, t_2(major) = 20.8 min.

Triethyl (*R*)-4-([1,1'-biphenyl]-4-yl)-2-methylbut-3-yne-1,1,1-tricarboxylate (3ab)

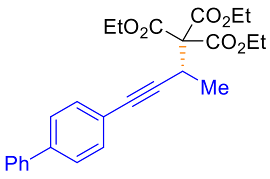

Colorless oil (63.0 mg, 96% yield). It was prepared according to the

general procedure described above. **^{1}H NMR (500 MHz, CDCl$_3$)** δ 7.60 – 7.55 (m, 2H), 7.53 – 7.48 (m, 2H), 7.46 – 7.41 (m, 4H), 7.37 – 7.32 (m, 1H), 4.31 (q, *J* = 7.1 Hz, 6H), 3.70 (q, *J* = 7.0 Hz, 1H), 1.51 (d, *J* = 7.0 Hz, 3H), 1.31 (t, *J* = 7.1 Hz, 9H). **^{13}C NMR (125 MHz, CDCl$_3$)** δ 165.88, 140.66, 140.54, 132.11, 128.94, 127.64, 127.10, 126.94, 122.48, 90.57, 82.29, 69.10, 62.30, 30.98, 17.71, 14.13. **ATR-FTIR (cm^{-1}):** 2960, 2923, 2854, 2239, 1739, 1486, 1463, 1367, 1261, 1095, 840, 800, 696. **ESI-MS:** calculated [$C_{26}H_{28}O_6$ + H]$^+$: 437.1959, found: 437.1948. $[\alpha]^{20}_D$ = -37.4 (c = 1.06, CH_2Cl_2). The product was analyzed by HPLC to determine the enantiomeric excess: 93% *ee* (IC, hexane/*i*-PrOH = 95/5, detector: 254 nm, flow rate: 1.0 mL/min), t_1(major) = 13.6 min, t_2(minor) = 15.5 min.

Triethyl (*R*)-2-methyl-4-(m-tolyl)but-3-yne-1,1,1-tricarboxylate (3ac)

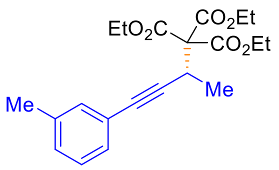

Colorless oil (53.3 mg, 95% yield). It was prepared according to the general procedure described above. **^{1}H NMR (500 MHz, CDCl$_3$)** δ 7.21 – 7.12 (m, 3H), 7.10 – 7.04 (m, 1H), 4.29 (q, *J* = 7.1 Hz, 6H), 3.67 (q, *J* = 6.9 Hz, 1H), 2.30 (s, 3H), 1.49 (d, *J* = 7.0 Hz, 3H), 1.30 (t, *J* = 7.1 Hz, 9H). **^{13}C NMR (125 MHz, CDCl$_3$)** δ 165.88, 137.86, 132.28, 128.82, 128.75, 128.12, 123.33, 89.41, 82.56, 69.09, 62.26, 30.89, 21.31, 17.72, 14.10. **ATR-FTIR (cm^{-1}):** 2983, 2939, 2229, 1743, 1463, 1367, 1261, 1095, 863, 786, 692. **ESI-MS:** calculated [$C_{21}H_{26}O_6$ + H]$^+$: 375.1802, found: 375.1807. $[\alpha]^{20}_D$ = -35.0 (c = 1.01, CH_2Cl_2). The product was analyzed by HPLC to determine the enantiomeric excess: 95% *ee* (IC, hexane/*i*-PrOH = 98/2, detector: 254 nm, flow rate: 1.0 mL/min), t_1(major) = 19.2 min, t_2(minor) = 21.4 min.

Triethyl (*R*)-2-methyl-4-(o-tolyl)but-3-yne-1,1,1-tricarboxylate (3ad)

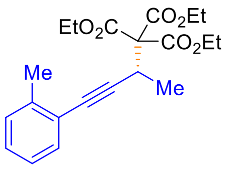

Colorless oil (53.6 mg, 95% yield). It was prepared according to the general procedure described above. **^{1}H NMR (500 MHz, CDCl$_3$)** δ 7.35 – 7.29 (m, 1H), 7.20 – 7.13 (m, 2H), 7.11 – 7.05 (m, 1H), 4.29 (q, *J* = 7.1 Hz, 6H), 3.73 (q, *J* = 7.0 Hz, 1H), 2.39 (s, 3H), 1.52 (d, *J* = 7.0 Hz, 3H), 1.29 (t, *J* = 7.1 Hz, 9H). **^{13}C NMR (125 MHz, CDCl$_3$)** δ 165.90, 140.34, 132.02, 129.35, 127.93, 125.43, 123.28, 93.71, 81.32, 69.11, 62.27, 31.04, 20.70, 17.94, 14.09. **ATR-FTIR (cm^{-1}):** 2981, 2925, 2239, 1743, 1456, 1367, 1261, 1097, 862, 757. **ESI-MS:** calculated [$C_{21}H_{26}O_6$ + H]$^+$: 375.1802, found: 375.1812. $[\alpha]^{20}_D$ = -30.6 (c = 1.04, CH_2Cl_2). The product was analyzed by HPLC to determine the enantiomeric excess: 92% *ee* (IC, hexane/*i*-PrOH = 98/2, detector: 254 nm, flow rate: 1.0 mL/min), t_1(major) = 14.5 min, t_2(minor) = 16.2 min.

Triethyl (*R*)-2-methyl-4-(thiophen-2-yl)but-3-yne-1,1,1-tricarboxylate (3ae)

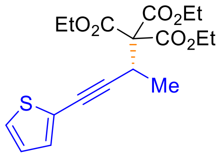

Colorless oil (50.0 mg, 91% yield). It was prepared according to the general procedure described above. **^{1}H NMR (500 MHz, CDCl$_3$)** δ 7.20 – 7.14 (m, 1H), 7.12 – 7.08 (m, 1H), 6.94 – 6.89 (m, 1H), 4.33 – 4.24 (m, 6H), 3.68 (q, *J* = 7.0 Hz, 1H), 1.48 (d, *J* = 7.0 Hz, 3H), 1.29 (t, *J* = 7.1 Hz, 9H). **^{13}C NMR (125 MHz, CDCl$_3$)** δ 165.78, 131.54, 126.87, 126.53, 123.56, 93.79, 75.59, 68.98, 62.36, 31.14, 17.52, 14.10. **ATR-FTIR (cm^{-1}):** 2983, 2940, 2902, 2254, 1743, 1519, 1463, 1367, 1263, 1097, 862, 703. **ESI-MS:** calculated [$C_{18}H_{22}O_6S$ + Na]$^+$:

389.1029, found: 389.1037. $[\alpha]^{20}_{D}$ = -32.3 (c = 1.00, CH_2Cl_2). The product was analyzed by HPLC to determine the enantiomeric excess: 97% *ee* (OD-H, hexane/*i*-PrOH = 99/1, detector: 254 nm, flow rate: 1.0 mL/min), t_1(minor) = 11.2 min, t_2(major) = 12.9 min.

Triethyl (*R*)-2-ethyl-4-phenylbut-3-yne-1,1,1-tricarboxylate (3af)

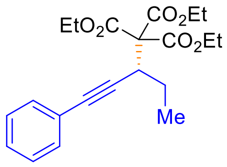

Colorless oil (53.8 mg, 96% yield). It was prepared according to the general procedure described above. **^{1}H NMR (500 MHz, CDCl$_3$)** δ 7.41 – 7.34 (m, 2H), 7.27 – 7.24 (m, 3H), 4.29 (q, *J* = 7.1 Hz, 6H), 3.40 (dd, *J* = 11.5, 2.9 Hz, 1H), 1.96 – 1.87 (m, 1H), 1.68 – 1.57 (m, 1H), 1.29 (t, *J* = 7.1 Hz, 9H), 1.17 (t, *J* = 7.3 Hz, 3H). **^{13}C NMR (125 MHz, CDCl$_3$)** δ 165.89, 131.71, 128.22, 127.90, 123.62, 88.56, 83.35, 69.06, 62.24, 38.46, 24.79, 14.08, 12.93. **ATR-FTIR (cm^{-1}):** 2981, 2935, 2875, 2242, 1743, 1598, 1490, 1444, 1367, 1263, 1058, 862, 757. **ESI-MS:** calculated $[C_{21}H_{26}O_6 + H]^+$: 375.1802, found: 375.1805. $[\alpha]^{20}_{D}$ = -60.7 (c = 1.12, CH_2Cl_2). The product was analyzed by HPLC to determine the enantiomeric excess: 97% *ee* (IC, hexane/*i*-PrOH = 98/2, detector: 254 nm, flow rate: 1.0 mL/min), t_1(minor) = 14.3 min, t_2(major) = 17.3 min.

Triethyl (*R*)-2-(phenylethynyl)pentane-1,1,1-tricarboxylate (3ag)

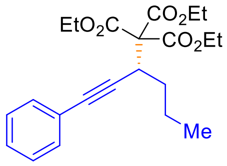

Colorless oil (55.0 mg, 94% yield). It was prepared according to the general procedure described above. **^{1}H NMR (500 MHz, CDCl$_3$)** δ 7.39 – 7.33 (m, 2H), 7.27 – 7.24 (m, 3H), 4.29 (q, *J* = 7.1 Hz, 6H), 3.50 (dd, *J* = 11.4, 2.2 Hz, 1H), 1.84 – 1.72 (m, 2H), 1.68 – 1.59 (m, 1H), 1.55 – 1.44 (m, 1H), 1.29 (t, *J* = 7.1 Hz, 9H), 0.98 (t, *J* = 7.1 Hz, 3H). **^{13}C NMR (125 MHz, CDCl$_3$)** δ 165.90, 131.69, 128.21, 127.88, 123.61, 88.78, 83.16, 69.00, 62.23, 36.34, 33.38, 21.41, 14.08, 13.80. **ATR-FTIR (cm^{-1}):** 2962, 2931, 2873, 2235, 1743, 1598, 1490, 1444, 1261, 1051, 862, 757, 692. **ESI-MS:** calculated $[C_{22}H_{28}O_6 + Na]^+$: 411.1778, found: 411.1772. $[\alpha]^{20}_{D}$ = -56.7 (c = 0.99, CH_2Cl_2). The product was analyzed by HPLC to determine the enantiomeric excess: 98% *ee* (IC, hexane/*i*-PrOH = 98/2, detector: 254 nm, flow rate: 1.0 mL/min), t_1(minor) = 12.4 min, t_2(major) = 15.5 min.

Triethyl (*R*)-2-methyl-6-phenylhex-3-yne-1,1,1-tricarboxylate (3ah)

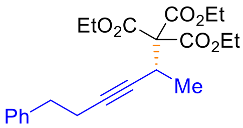

Colorless oil (56.1 mg, 96% yield). It was prepared according to the general procedure described above. **^{1}H NMR (500 MHz, CDCl$_3$)** δ 7.31 – 7.26 (m, 2H), 7.22 – 7.16 (m, 3H), 4.25 (q, *J* = 7.1 Hz, 6H), 3.47 – 3.39 (m, 1H), 2.77 (t, *J* = 7.7 Hz, 2H), 2.45 – 2.40 (m, 2H), 1.37 (d, *J* = 7.0 Hz, 3H), 1.27 (t, *J* = 7.1 Hz, 9H). **^{13}C NMR (125 MHz, CDCl$_3$)** δ 165.93, 140.97, 128.56, 128.42, 126.26, 81.80, 80.66, 68.97, 62.12, 35.44, 30.42, 21.09, 18.01, 14.07. **ATR-FTIR (cm^{-1}):** 2983, 2927, 2242, 1747, 1454, 1261, 1097, 862, 750, 703. **ESI-MS:** calculated $[C_{22}H_{28}O_6 + H]^+$: 389.1959, found: 389.1972. $[\alpha]^{20}_{D}$ = -16.6 (c = 1.12, CH_2Cl_2). The product was analyzed by HPLC to determine the enantiomeric excess: 92% *ee* (OJ-H, hexane/*i*-PrOH = 99/1, detector: 211 nm, flow rate: 1.0 mL/min), t_1(major) = 21.1 min, t_2(minor) = 26.3 min.

Triethyl (*R*)-2-methylhex-3-yne-1,1,1-tricarboxylate (3ai)

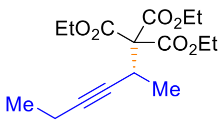

Colorless oil (26.0 mg, 55% yield). It was prepared according to the general procedure described above. **^{1}H NMR (500 MHz, $CDCl_3$)** δ 4.27 (q, J = 7.1 Hz, 6H), 3.46 – 3.38 (m, 1H), 2.18 – 2.09 (m, 2H), 1.37 (d, J = 7.0 Hz, 3H), 1.29 (t, J = 7.1 Hz, 9H), 1.08 (t, J = 7.5 Hz, 3H). **^{13}C NMR (125 MHz, $CDCl_3$)** δ 165.95, 83.80, 79.32, 69.06, 62.06, 30.36, 18.00, 14.16, 14.04, 12.48. **ATR-FTIR (cm^{-1}):** 2979, 2923, 2852, 2240, 1747, 1367, 1265, 1097, 862. **ESI-MS:** calculated $[C_{16}H_{24}O_6 + H]^+$: 313.1646, found: 313.1652. $[\alpha]^{20}_D$ = -12.6 (c = 0.99, CH_2Cl_2). The product was analyzed by HPLC to determine the enantiomeric excess: 92% *ee* (IC, hexane/*i*-PrOH = 99/1, detector: 211 nm, flow rate: 1.0 mL/min), t_1(minor) = 37.4 min, t_2(major) = 45.9 min.

Triethyl (*R*)-2-methylhept-3-yne-1,1,1-tricarboxylate (3aj)

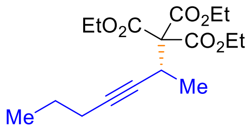

Colorless oil (46.0 mg, 94% yield). It was prepared according to the general procedure described above. **^{1}H NMR (500 MHz, $CDCl_3$)** δ 4.27 (q, J = 7.1 Hz, 6H), 3.47 – 3.39 (m, 1H), 2.14 – 2.07 (m, 2H), 1.51 – 1.43 (m, 2H), 1.38 (d, J = 7.0 Hz, 3H), 1.29 (t, J = 7.1 Hz, 9H), 0.95 (t, J = 7.4 Hz, 3H). **^{13}C NMR (125 MHz, $CDCl_3$)** δ 165.97, 82.34, 80.15, 69.09, 62.07, 30.39, 22.39, 20.80, 18.07, 14.04, 13.47. **ATR-FTIR (cm^{-1}):** 2981, 2935, 2244, 1747, 1367, 1232, 1097, 862. **ESI-MS:** calculated $[C_{17}H_{26}O_6 + H]^+$: 327.1802, found: 327.1794. $[\alpha]^{20}_D$ = -25.0 (c = 1.12, CH_2Cl_2). The product was analyzed by HPLC to determine the enantiomeric excess: 92% *ee* (IC, hexane/*i*-PrOH = 99/1, detector: 211 nm, flow rate: 1.0 mL/min), t_1(minor) = 31.6 min, t_2(major) = 38.8 min.

Dimethyl (*R*)-2-allyl-2-(hex-3-yn-2-yl)malonate (5)

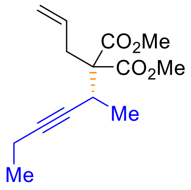

Colorless oil (0.71 g, 80% yield). It was prepared according to the procedure described above. **^{1}H NMR (500 MHz, $CDCl_3$)** δ 5.91 – 5.71 (m, 1H), 5.17 – 5.01 (m, 2H), 3.75 (s, 3H), 3.71 (s, 3H), 3.19 – 3.07 (m, 1H), 2.80 (d, J = 7.2 Hz, 2H), 2.22 – 2.09 (m, 2H), 1.27 (d, J = 7.0 Hz, 3H), 1.10 (t, J = 7.5 Hz, 3H). **^{13}C NMR (125 MHz, $CDCl_3$)** δ 170.57, 170.19, 133.29, 118.78, 84.79, 79.29, 61.51, 52.32, 52.23, 38.72, 30.89, 18.03, 14.33, 12.50. **ATR-FTIR (cm^{-1}):** 2977, 2921, 2850, 2244, 1735, 1639, 1376, 1228, 1033, 921. **ESI-MS:** calculated $[C_{14}H_{20}O_4 + H]^+$: 253.1434, found: 253.1428. $[\alpha]^{20}_D$ = -34.3 (c = 1.05, CH_2Cl_2). The product was analyzed by HPLC to determine the enantiomeric excess: 88% *ee* (IG, hexane/*i*-PrOH = 99/1, detector: 220 nm, flow rate: 0.3 mL/min), t_1(minor) = 18.2 min, t_2(major) = 21.0 min.

(*R*)-5-allyl-5-(hex-3-yn-2-yl)-2-thioxodihydropyrimidine-4,6(1H,5H)-dione (6)

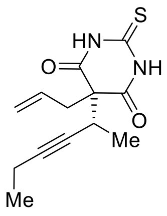

Yellow solid (0.41 g, 55% yield). It was prepared according to the procedure described above. **M.p.** 124-126 °C. **^{1}H NMR (500 MHz, $CDCl_3$)** δ 9.68 – 9.48 (m, 2H), 5.65 – 5.51 (m, 1H), 5.21 – 5.06 (m, 2H), 3.19 – 3.04 (m, 1H), 2.87 (dd, J = 13.0, 7.4 Hz, 1H), 2.67 (dd, J = 13.0, 7.3 Hz, 1H), 2.17 – 2.07 (m, 2H), 1.31 (d, J = 7.1 Hz, 3H), 1.06 (t, J = 7.5 Hz, 3H). **^{13}C NMR (125 MHz, $CDCl_3$)** δ 176.62, 169.95, 168.11, 130.84, 121.48, 87.02, 77.09, 59.85, 37.87, 36.04, 16.22, 13.89, 12.47. **ATR-FTIR (cm^{-1}):** 3218, 2977, 2937, 2242, 1733, 1697, 1523, 1359, 1247, 1155, 931. **ESI-MS:** calculated $[C_{13}H_{16}N_2O_2S + H]^+$: 265.1005, found:

265.1008. $[\alpha]^{20}_D$ = -49.1 (c = 0.94, CH_2Cl_2). The product was analyzed by HPLC to determine the enantiomeric excess: 88% *ee* (IF, hexane/*i*-PrOH = 90/10, detector: 300 nm, flow rate: 1.0 mL/min), t_1(major) = 16.6 min, t_2(minor) = 22.0 min.

Dimethyl (*R*)-2-ethyl-2-(pent-3-yn-2-yl)malonate (7)

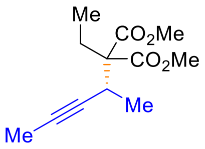

Colorless oil (0.59 g, 65% yield). It was prepared according to the procedure described above. **^{1}H NMR (500 MHz, CDCl$_3$)** δ 3.74 (s, 3H), 3.72 (s, 3H), 3.18 – 3.08 (m, 1H), 2.12 – 2.03 (m, 2H), 1.77 (d, *J* = 2.4 Hz, 3H), 1.24 (d, *J* = 7.0 Hz, 3H), 0.90 (t, *J* = 7.5 Hz, 3H). **^{13}C NMR (125 MHz, CDCl$_3$)** δ 171.14, 170.75, 79.19, 78.25, 61.69, 52.29, 52.14, 30.73, 27.40, 18.09, 9.42, 3.68. **ATR-FTIR (cm^{-1}):** 2981, 2952, 2923, 2246, 1735, 1434, 1382, 1240, 1136, 916. **ESI-MS:** calculated $[C_{12}H_{18}O_4 + H]^+$: 227.1278, found: 227.1284. $[\alpha]^{20}_D$ = -11.5 (c = 0.17, CH_2Cl_2). The product was analyzed by HPLC to determine the enantiomeric excess: 90% *ee* (IC, hexane/*i*-PrOH = 99/1, detector: 211 nm, flow rate: 0.3 mL/min), t_1(minor) = 32.4 min, t_2(major) = 35.2 min.

Dimethyl (*R*)-2-ethyl-2-(pentan-2-yl)malonate (8)

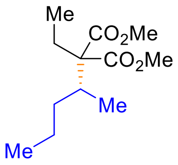

Colorless oil (0.59 g, 86% yield). It was prepared according to the procedure described above. **^{1}H NMR (500 MHz, CDCl$_3$)** δ 3.75 – 3.65 (m, 6H), 2.12 – 2.02 (m, 1H), 1.93 (q, *J* = 7.5 Hz, 2H), 1.49 – 1.38 (m, 2H), 1.26 – 1.17 (m, 1H), 1.00 – 0.94 (m, 1H), 0.93 – 0.80 (m, 9H). **^{13}C NMR (125 MHz, CDCl$_3$)** δ 172.01, 171.84, 62.99, 51.89, 51.87, 37.02, 35.07, 27.20, 21.36, 15.08, 14.25, 9.50. **ATR-FTIR (cm^{-1}):** 2954, 2875, 1735, 1434, 1386, 1236, 1133, 914, 804. **ESI-MS:** calculated $[C_{12}H_{22}O_4 + H]^+$: 231.1591, found: 231.1592. $[\alpha]^{20}_D$ = 10.2 (c = 0.79, CH_2Cl_2).

(*R*)-5-ethyl-5-(pentan-2-yl)-2-thioxodihydropyrimidine-4,6(1H,5H)-dione (9)

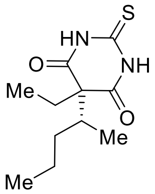

Yellow solid (0.81 g, 67% yield). It was prepared according to the procedure described above. **M.p.** 142-144 °C. **^{1}H NMR (500 MHz, CDCl$_3$)** δ 9.09 (brs, 2H), 2.20 – 2.04 (m, 3H), 1.50 – 1.36 (m, 2H), 1.23 – 1.12 (m, 2H), 1.04 (d, *J* = 6.9 Hz, 3H), 0.90 – 0.83 (m, 6H). **^{13}C NMR (125 MHz, CDCl$_3$)** δ 176.12, 170.53, 170.16, 61.39, 43.08, 34.02, 28.76, 20.83, 14.47, 14.06, 9.95. **ATR-FTIR (cm^{-1}):** 3259, 2985, 2931, 2873, 1735, 1672, 1542, 1427, 1365, 1222, 1172, 840. **ESI-MS:** calculated $[C_{11}H_{18}N_2O_2S + H]^+$: 243.1162, found: 243.1164. $[\alpha]^{20}_D$ = 11.2 (c = 0.90, CH_2Cl_2). The product was analyzed by HPLC to determine the enantiomeric excess: 90% *ee* (IF, hexane/*i*-PrOH = 90/10, detector: 300 nm, flow rate: 1.0 mL/min), t_1(major) = 13.7 min, t_2(minor) = 15.9 min.

(*R*)-5-ethyl-5-(pentan-2-yl)pyrimidine-2,4,6(1H,3H,5H)-trione (10)

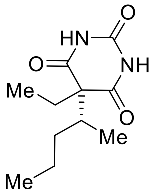

Yellow solid (0.79 g, 70% yield). It was prepared according to the procedure described above. **M.p.** 107-108 °C. **^{1}H NMR (500 MHz, CDCl$_3$)** δ 8.90 (brs, 2H), 2.20 – 2.03 (m, 3H), 1.50 – 1.38 (m, 2H), 1.23 – 1.11 (m, 2H), 1.04 (d, *J* = 6.9 Hz, 3H), 0.89 – 0.82 (m, 6H). **^{13}C NMR (125 MHz, CDCl$_3$)** δ 172.91, 172.57, 149.56, 61.12, 42.55, 34.01, 28.73, 20.87, 14.38, 14.07, 9.90.

ATR-FTIR (cm^{-1}): 3234, 2962, 2875, 1710, 1427, 1359, 1218, 1149, 792. **ESI-MS:** calculated $[C_{11}H_{18}N_2O_3 + H]^+$: 227.1390, found: 227.1394. $[\alpha]^{20}_D$ = 10.7 (c = 0.80, CH_2Cl_2). The product was analyzed by HPLC to determine the enantiomeric excess: 90% *ee* (IF, hexane/*i*-PrOH = 95/5, detector: 211 nm, flow rate: 1.0 mL/min), t_1(major) = 40.8 min, t_2(minor) = 45.2 min.

(*R*)-3-(4-((4'-(trifluoromethyl)-[1,1'-biphenyl]-3-yl)methoxy)phenyl)hex-4-ynoic acid (11)

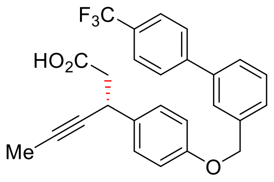

Yellow oil (1.62 g, 88% yield). It was prepared according to the procedure described above. **^{1}H NMR (400 MHz, $CDCl_3$)** δ 7.71 – 7.63 (m, 5H), 7.54 (d, *J* = 6.9 Hz, 1H), 7.50 – 7.42 (m, 2H), 7.31 (d, *J* = 8.3 Hz, 2H), 6.95 (d, *J* = 8.3 Hz, 2H), 5.10 (s, 2H), 4.10 – 4.01 (m, 1H), 2.87 – 2.64 (m, 2H), 1.82 (s, 3H). **^{19}F NMR (375 MHz, $CDCl_3$)** δ -62.34 (s). **^{13}C NMR (100 MHz, $CDCl_3$)** δ 177.25, 157.92, 144.52, 140.26, 137.99, 133.57, 129.60 (q, *J* = 32.0 Hz), 129.40, 128.58, 127.60, 127.38, 127.04, 126.46, 125.86 (q, *J* = 3.0 Hz), 124.41 (q, *J* = 271.0 Hz), 115.10, 79.50, 79.26, 70.01, 43.42, 33.27, 3.77. **ATR-FTIR (cm^{-1}):** 2921, 2237, 1712, 1508, 1328, 1243, 1124, 842, 790, 700. **ESI-MS:** calculated $[C_{26}H_{21}F_3O_3 + H]^+$: 439.1516 found: 439.1524. $[\alpha]^{20}_D$ = -10.2 (c = 1.11, CH_2Cl_2). The product was analyzed by HPLC to determine the enantiomeric excess: 94% *ee* (AD-H, hexane/*i*-PrOH = 80/20, detector: 254 nm, flow rate: 1.0 mL/min), t_1(major) = 12.5 min, t_2(minor) = 20.6 min.

(*S*)-3-methyl-5-phenylpent-4-ynoic acid (12)

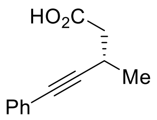

Colorless oil (0.69 g, 91% yield). It was prepared according to the procedure described above. **^{1}H NMR (400 MHz, $CDCl_3$)** δ 7.42 – 7.35 (m, 2H), 7.28 – 7.26 (m, 3H), 3.23 – 3.11 (m, 1H), 2.76 – 2.65 (m, 1H), 2.63 – 2.46 (m, 1H), 1.34 (d, *J* = 6.9 Hz, 3H). **^{13}C NMR (125 MHz, $CDCl_3$)** δ 177.61 131.77, 128.32, 127.96, 123.53, 92.28, 81.42, 41.50, 23.32, 20.87. **ATR-FTIR (cm^{-1}):** 2926, 2210, 1780, 1710, 1520, 1470, 846, 750. **ESI-MS:** calculated $[C_{12}H_{12}O_2 + H]^+$: 189.0910, found: 189.0913.

(*R*)-3-methyl-5-phenylpentan-1-ol (13)

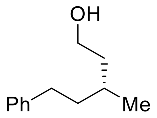

Colorless oil (0.55 g, 84% yield). It was prepared according to the procedure described above. **^{1}H NMR (400 MHz, $CDCl_3$)** δ 7.30 – 7.24 (m, 2H), 7.22 – 7.13 (m, 3H), 3.75 – 3.63 (m, 2H), 2.74 – 2.51 (m, 2H), 1.70 – 1.60 (m, 3H), 1.52 – 1.39 (m, 2H), 1.31 (brs, 1H), 0.97 (d, *J* = 6.5 Hz, 3H). **^{13}C NMR (100 MHz, $CDCl_3$)** δ 142.94, 128.46, 128.44, 125.77, 61.20, 39.91, 39.15, 33.48, 29.34, 19.66. **ATR-FTIR (cm^{-1}):** 3344, 2929, 2869, 1604, 1496, 1456, 746, 698. **ESI-MS:** calculated $[C_{12}H_{18}O + H]^+$: 179.1430, found: 179.1434. $[\alpha]^{20}_D$ = 14.0 (c = 0.71, CH_2Cl_2). The product was analyzed by HPLC to determine the enantiomeric excess: 93% *ee* (OJ-H, hexane/*i*-PrOH = 98/2, detector: 254 nm, flow rate: 1.0 mL/min), t_1(minor) = 24.9 min, t_2(major) = 31.4 min.

(*R*)-3-methyl-5-phenylpentanal (14)

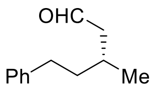

Colorless oil (47.0 mg, 79% yield). It was prepared according to the procedure described above. **^{1}H NMR (500 MHz, $CDCl_3$)** δ 9.74 (t, *J* = 2.2 Hz, 1H), 7.31

– 7.25 (m, 2H), 7.23 – 7.12 (m, 3H), 2.72 – 2.56 (m, 2H), 2.48 – 2.40 (m, 1H), 2.31 – 2.24 (m, 1H), 2.15 – 2.07 (m, 1H), 1.71 – 1.63 (m, 1H), 1.60 – 1.52 (m, 1H), 1.03 (d, J = 6.7 Hz, 3H). **^{13}C NMR (125 MHz, $CDCl_3$)** δ 202.88, 142.20, 128.53, 128.43, 125.98, 51.08, 38.76, 33.39, 27.92, 19.97. **ATR-FTIR (cm^{-1}):** 2927, 2856, 1724, 1496, 1409, 1380, 744, 700. **ESI-MS:** calculated $[C_{12}H_{16}O + H]^+$: 177.1274, found: 177.1275. $[\alpha]^{20}_D$ = 20.8 (c = 1.03, CH_2Cl_2). The product was analyzed by HPLC to determine the enantiomeric excess: 93% *ee* (IB, hexane/*i*-PrOH = 98/2, detector: 310 nm, flow rate: 1.0 mL/min), t_1(major) = 9.3 min, t_2(minor) = 10.0 min.

(*R*)-3-methyl-5-phenylpentanenitrile (15)

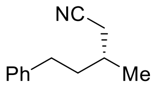

Colorless oil (15.7 mg, 60% yield). It was prepared according to the procedure described above. **^{1}H NMR (400 MHz, $CDCl_3$)** δ 7.34 – 7.26 (m, 2H), 7.26 – 7.13 (m, 3H), 2.73 – 2.58 (m, 2H), 2.38 – 2.25 (m, 2H), 1.94 – 1.83 (m, 1H), 1.83 – 1.72 (m, 1H), 1.69 – 1.60 (m, 1H), 1.14 (d, J = 6.7 Hz, 3H). **^{13}C NMR (101 MHz, $CDCl_3$)** δ 141.47, 128.62, 128.39, 126.18, 118.80, 37.59, 33.20, 30.02, 24.61, 19.45. **ATR-FTIR (cm^{-1}):** 2960, 2923, 2856, 2246, 1602, 1454, 1384, 800, 698. **ESI-MS:** calculated $[C_{12}H_{15}N + H]^+$: 174.1277, found: 174.1285. $[\alpha]^{20}_D$ = -2.2 (c = 0.66, EtOH). The product was analyzed by HPLC to determine the enantiomeric excess: 93% *ee* (OD-H, hexane/*i*-PrOH = 95/5, detector: 211 nm, flow rate: 1.0 mL/min), t_1(major) = 15.1 min, t_2(minor) = 16.4 min.

10. X-ray crystallography data

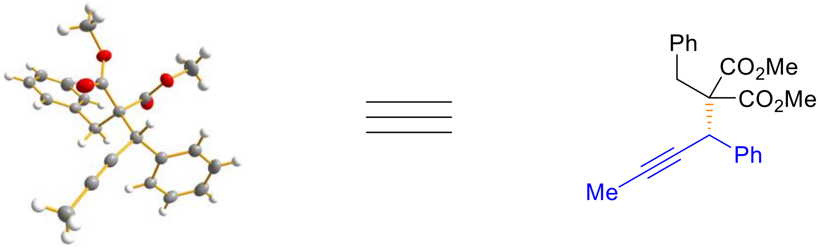

3e

Chemical formula	$C_{22}H_{22}O_4$
Formula weight	350.39
Space group	P1
Z	2
α, Å	8.7530(2)
b, Å	10.4823(2)
c, Å	11.9201(3)
α, °	98
β, °	104
γ, °	114
V, $Å^3$	932.02(4)
Flack parameter	0.06(7)

11. NMR spectra

^{1}H NMR spectrum of **1a**

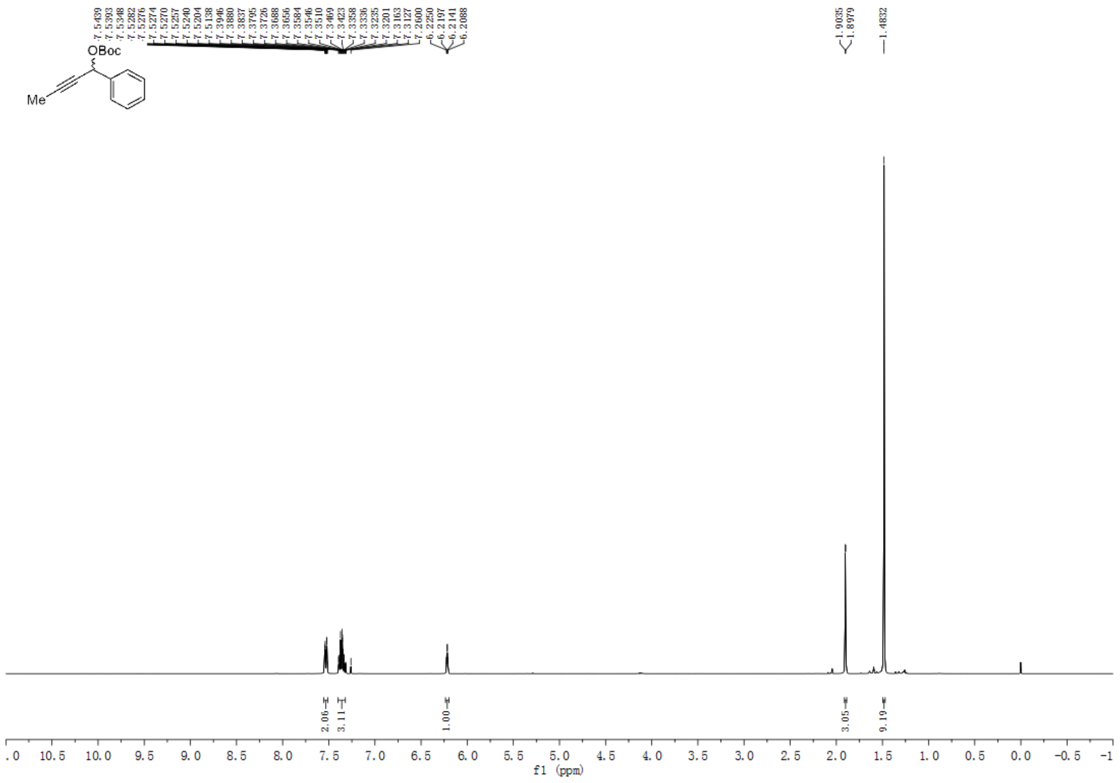

^{13}C NMR spectrum of **1a**

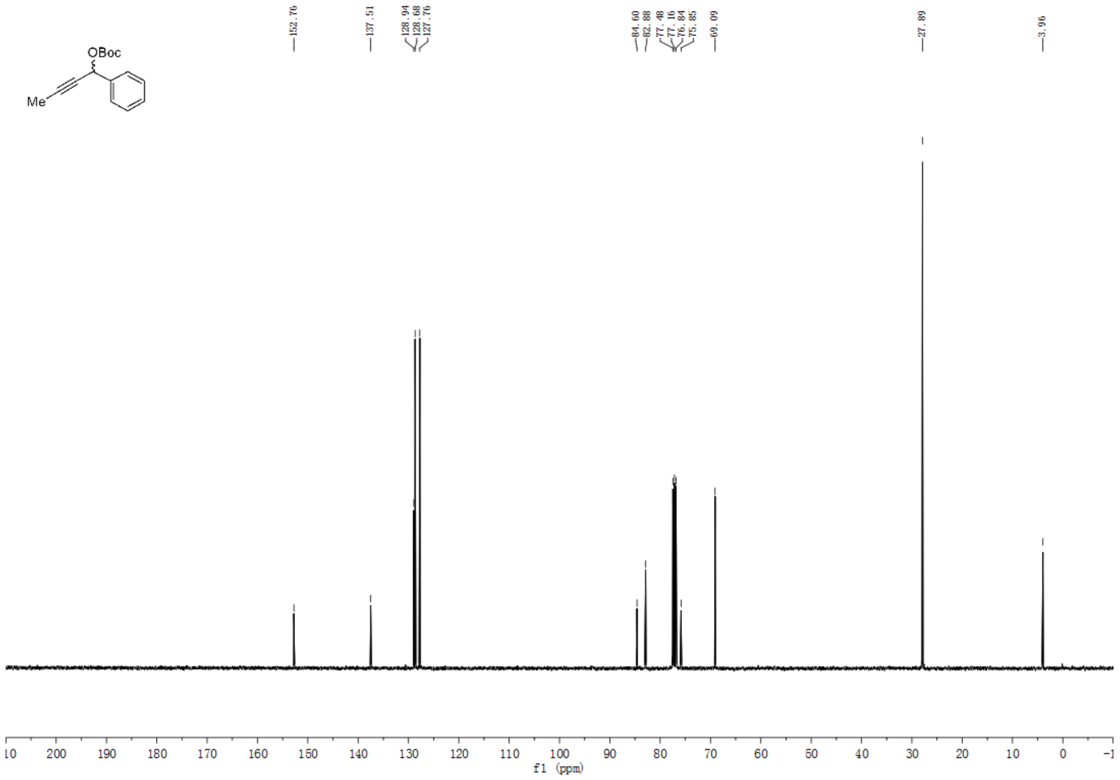

^{1}H NMR spectrum of **1b**

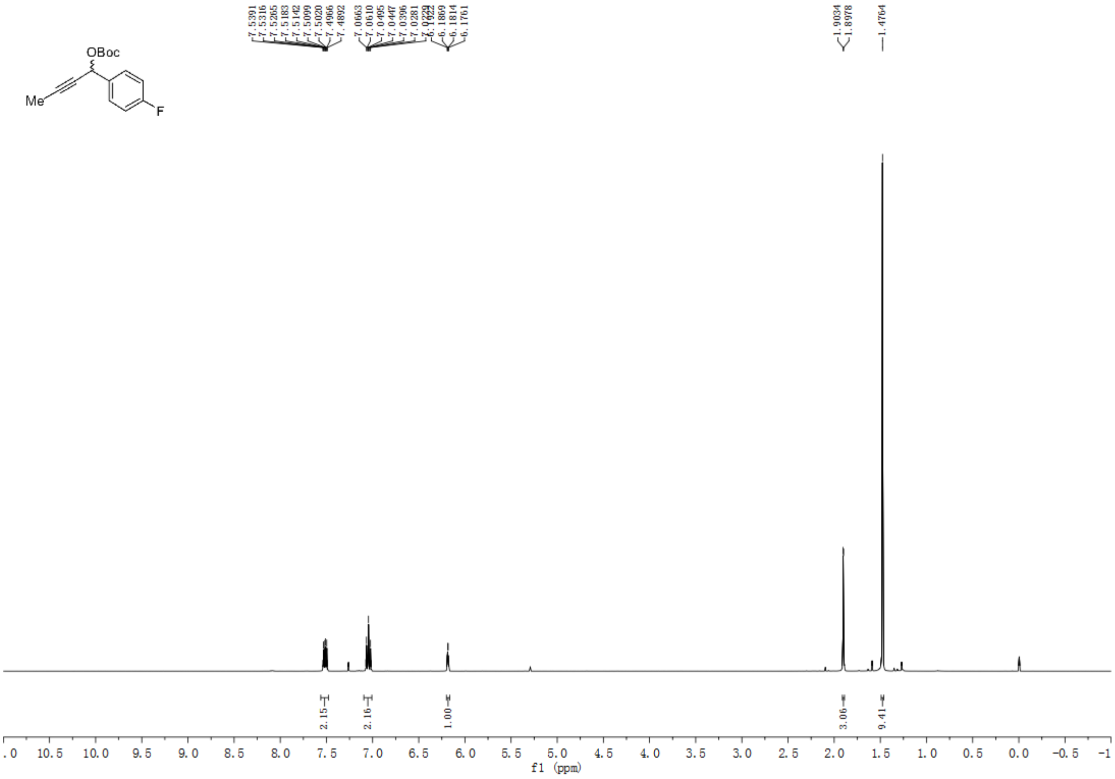

^{13}C NMR spectrum of **1b**

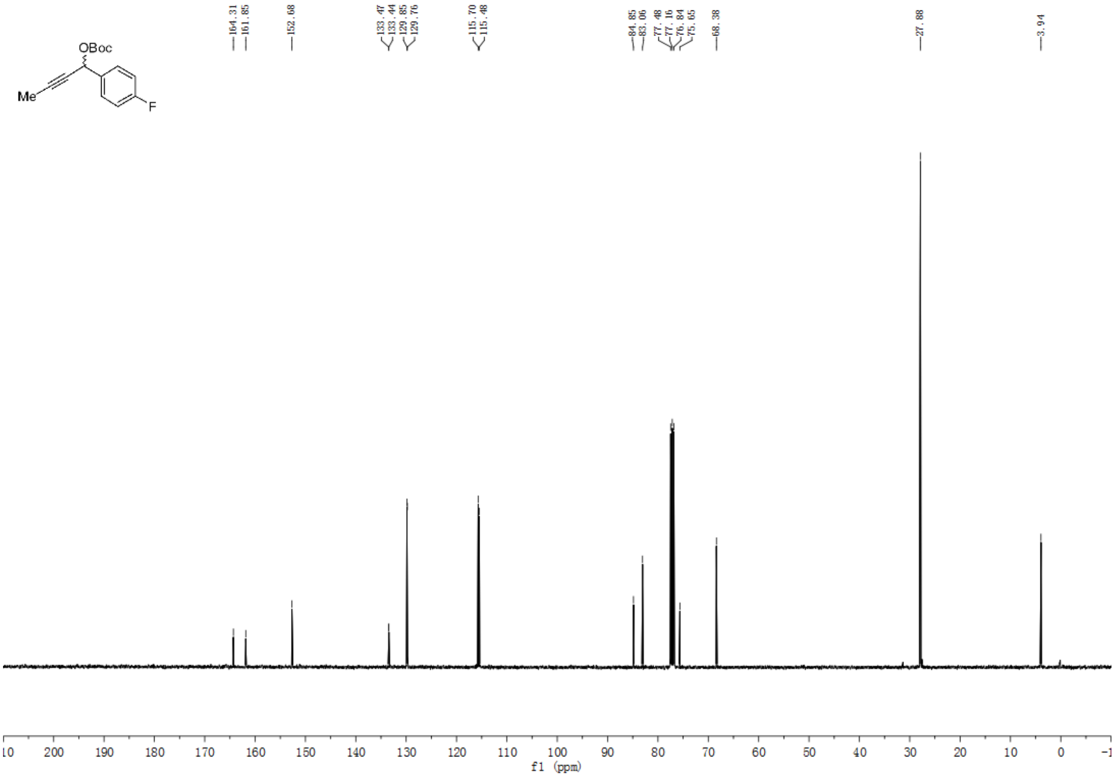

^{19}F NMR spectrum of **1b**

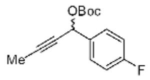

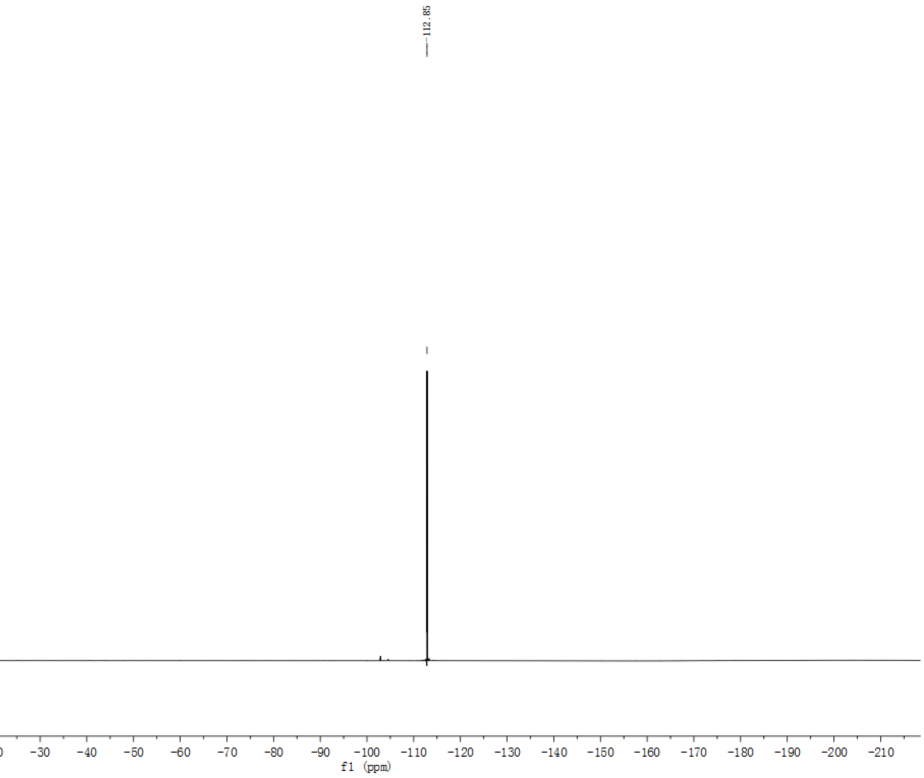

^{1}H NMR spectrum of **1c**

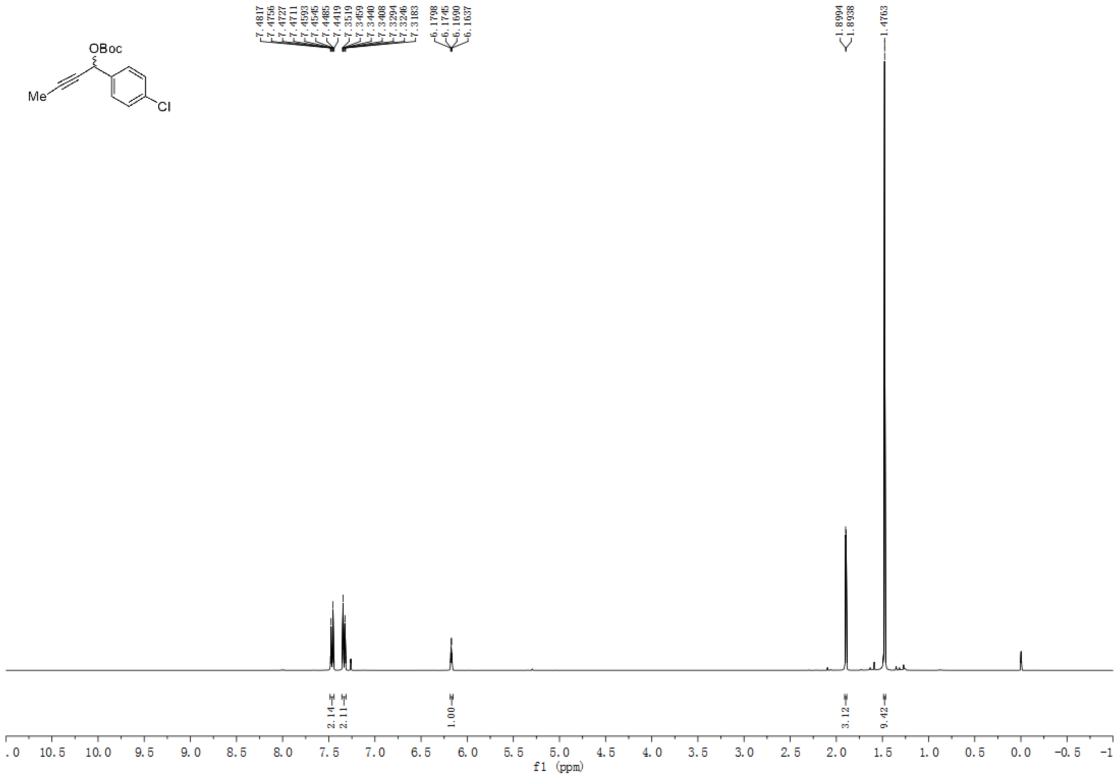

^{13}C NMR spectrum of **1c**

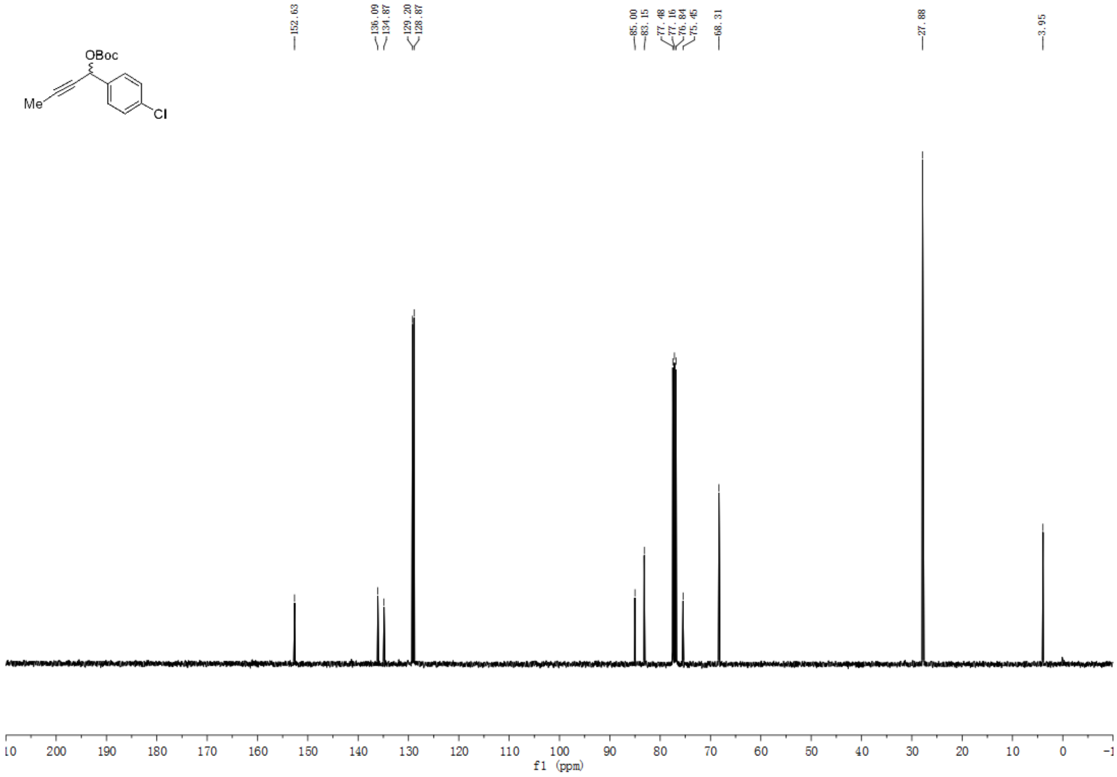

^{1}H NMR spectrum of **1d**

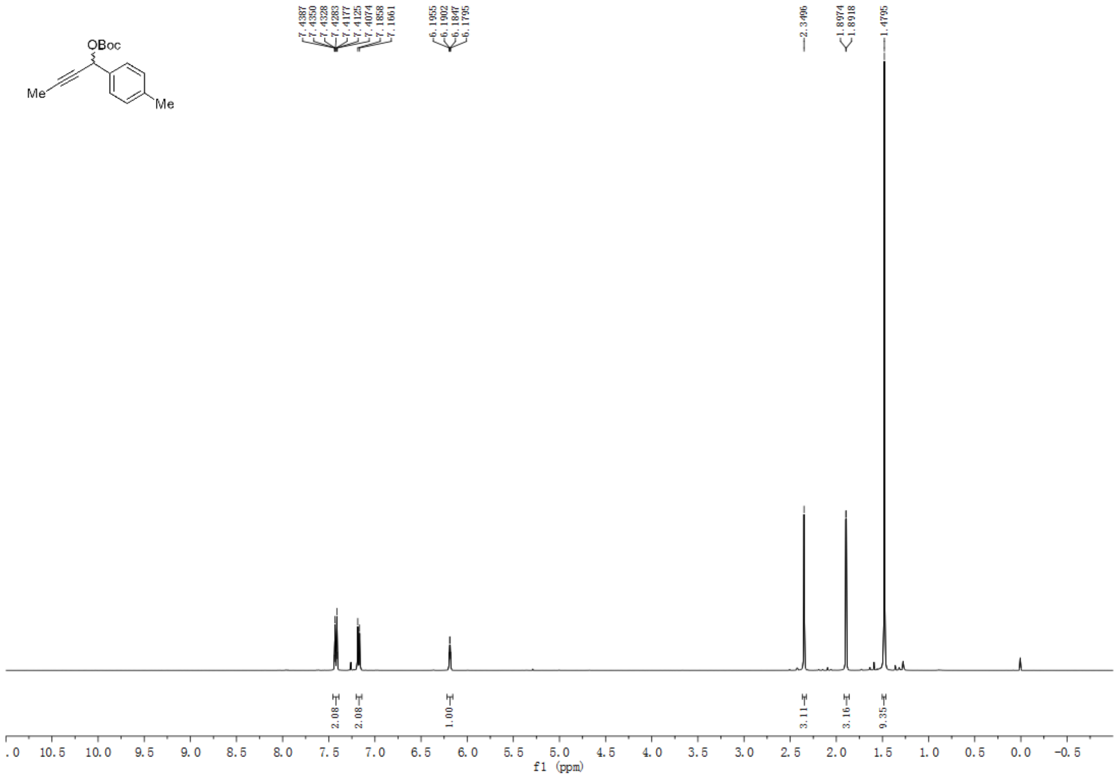

^{13}C NMR spectrum of **1d**

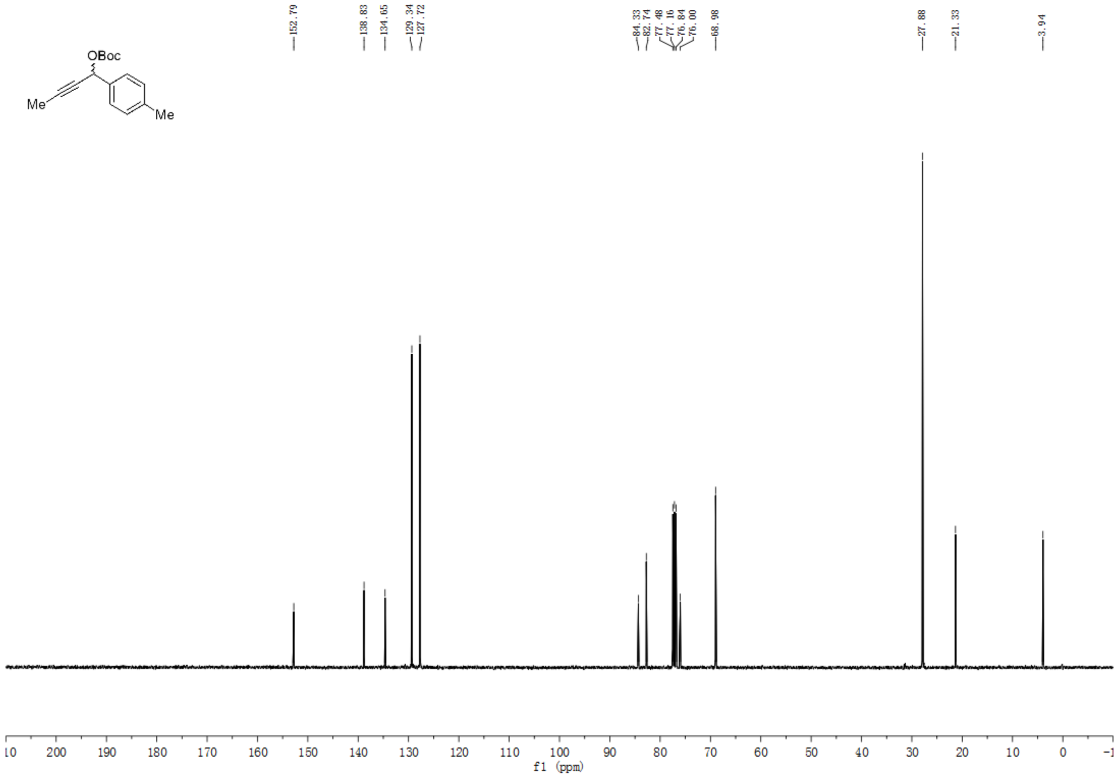

^{1}H NMR spectrum of **1e**

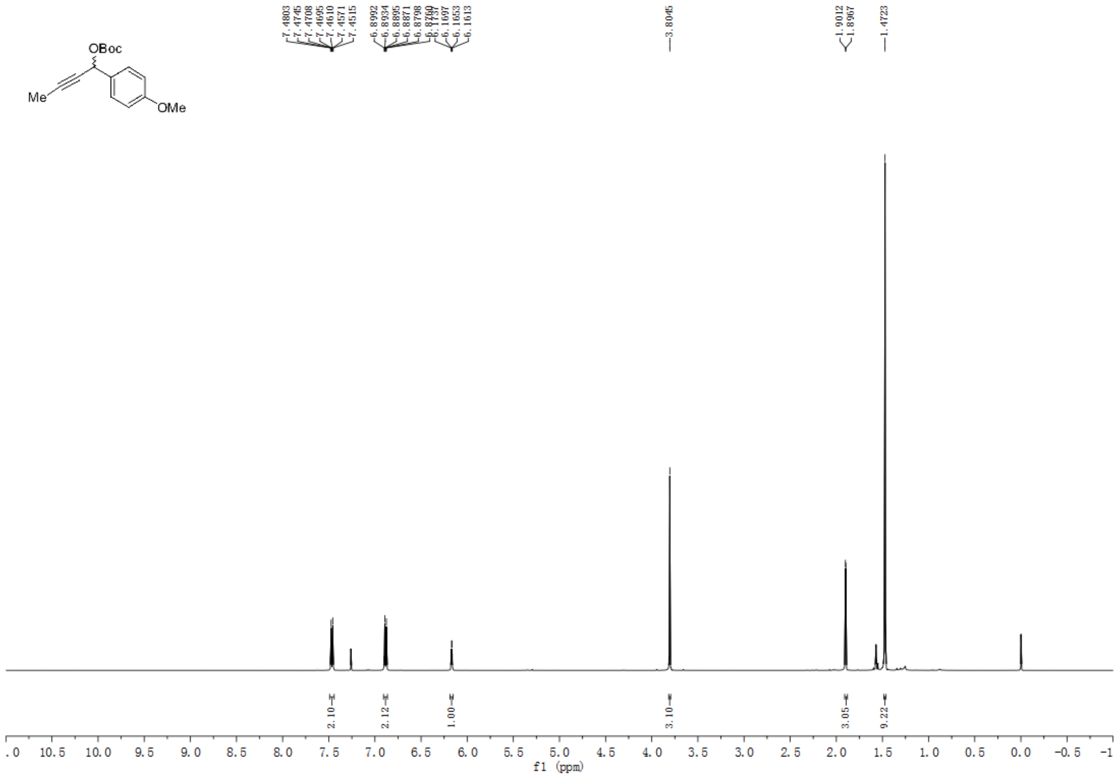

^{13}C NMR spectrum of **1e**

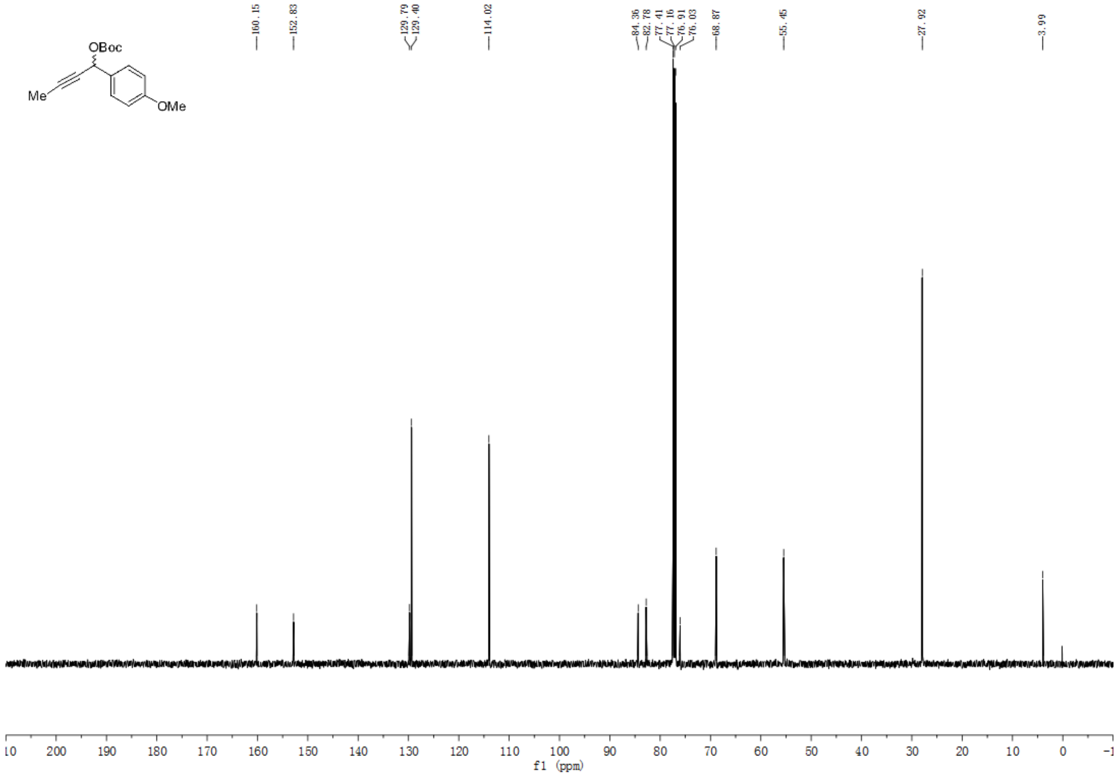

^{1}H NMR spectrum of **1f**

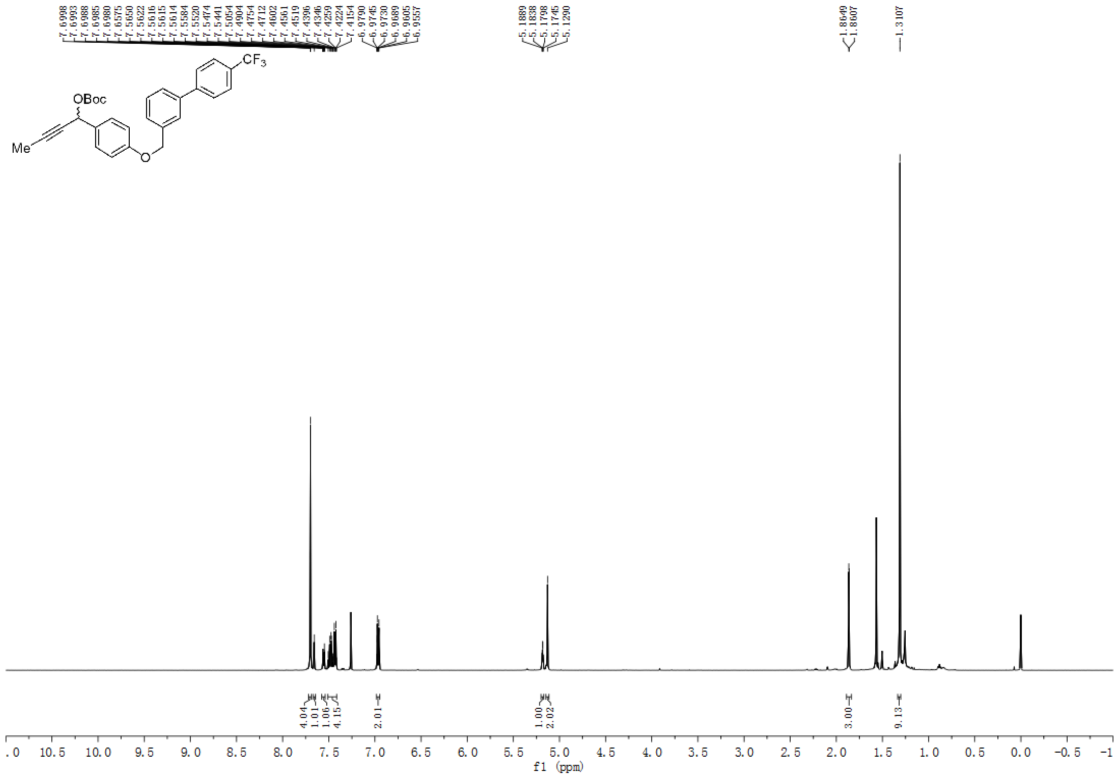

^{13}C NMR spectrum of **1f**

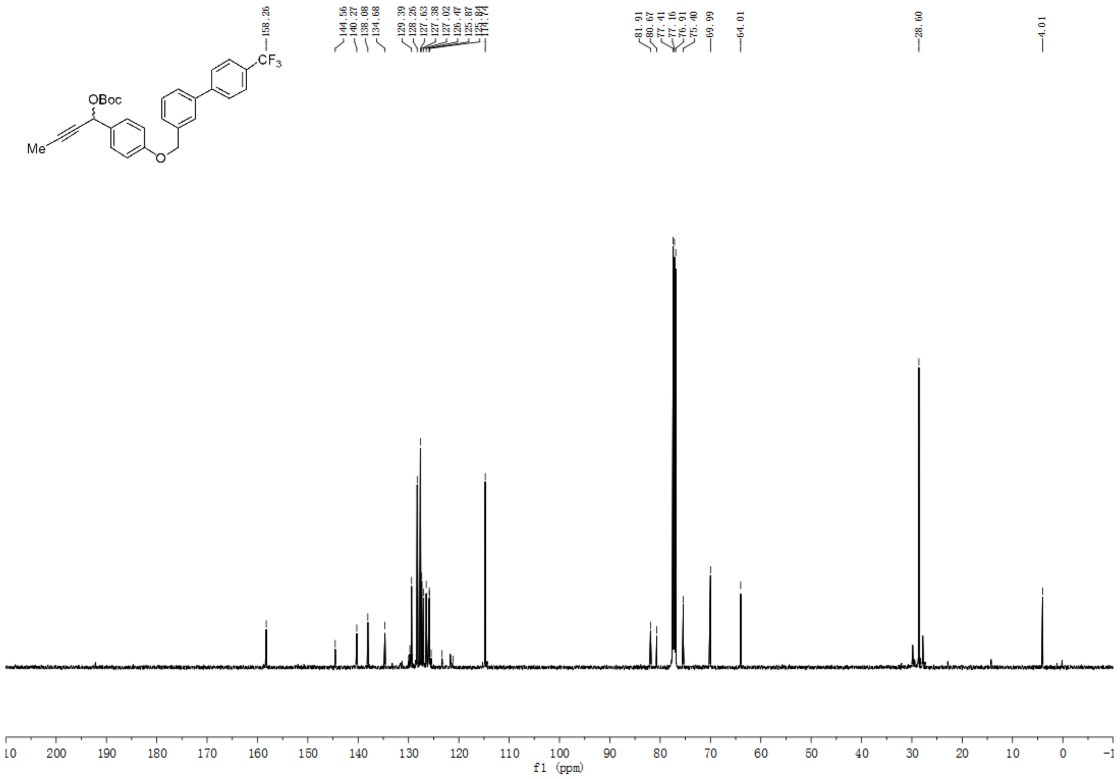

^{19}F NMR spectrum of **1f**

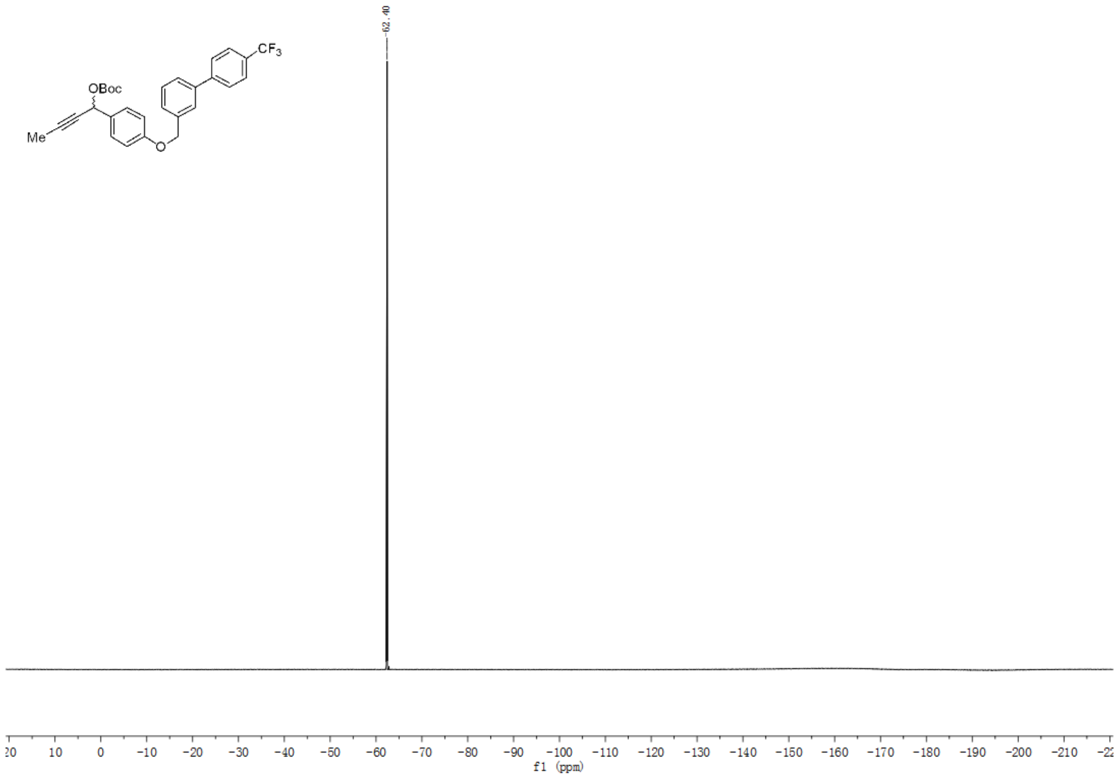

^{1}H NMR spectrum of **1g**

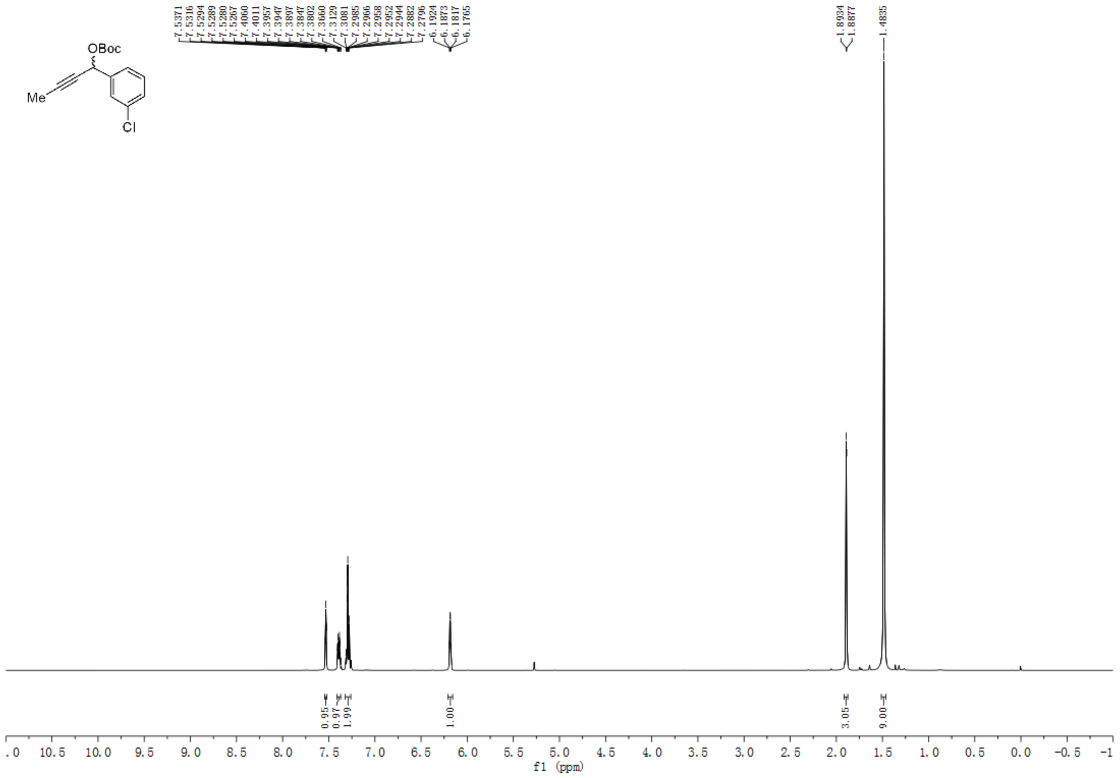

^{13}C NMR spectrum of **1g**

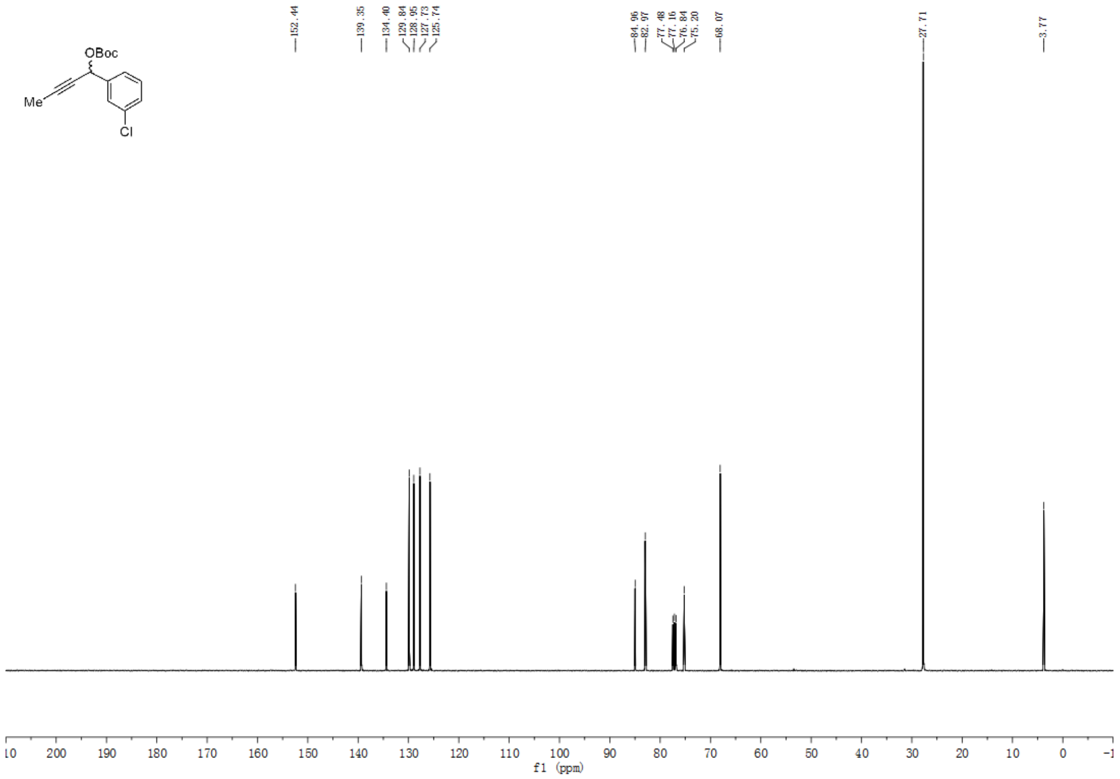

^{1}H NMR spectrum of **1h**

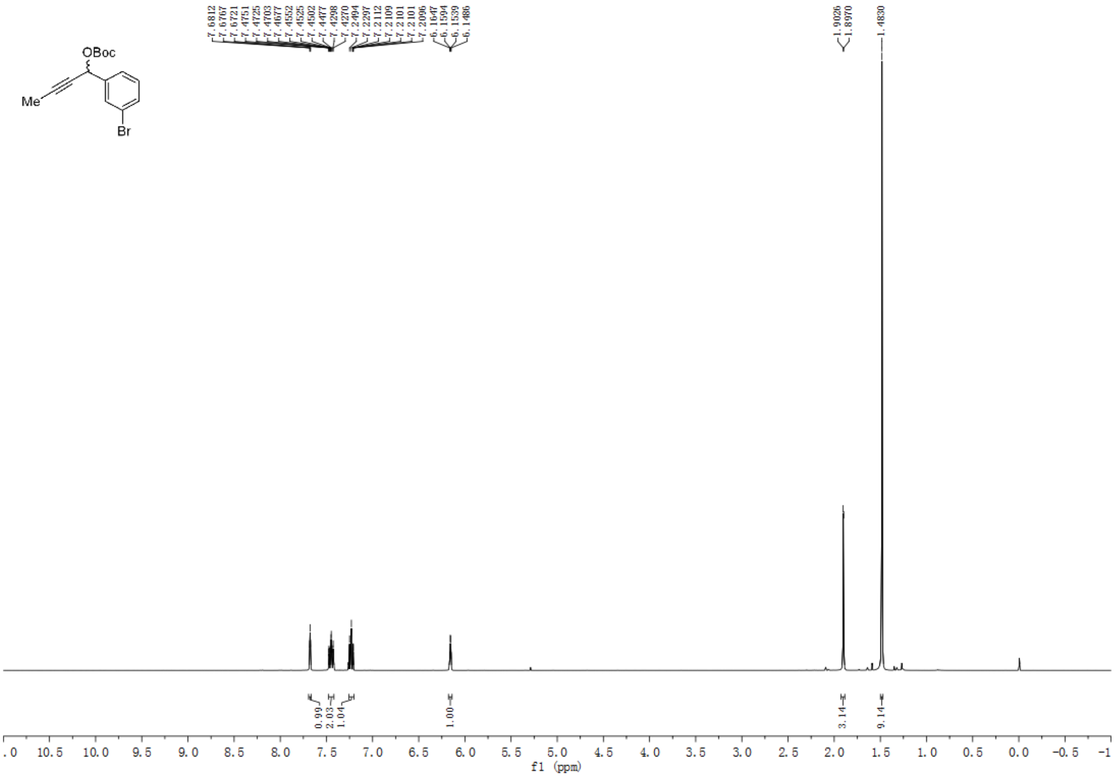

^{13}C NMR spectrum of **1h**

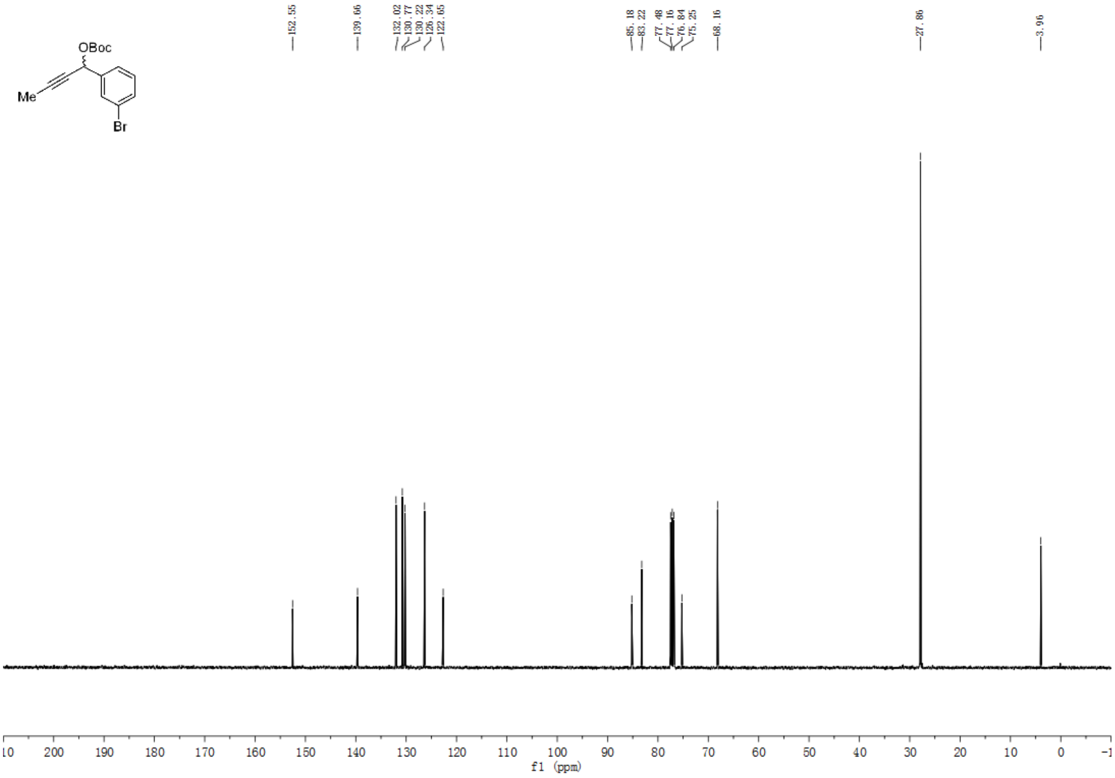

^{1}H NMR spectrum of **1i**

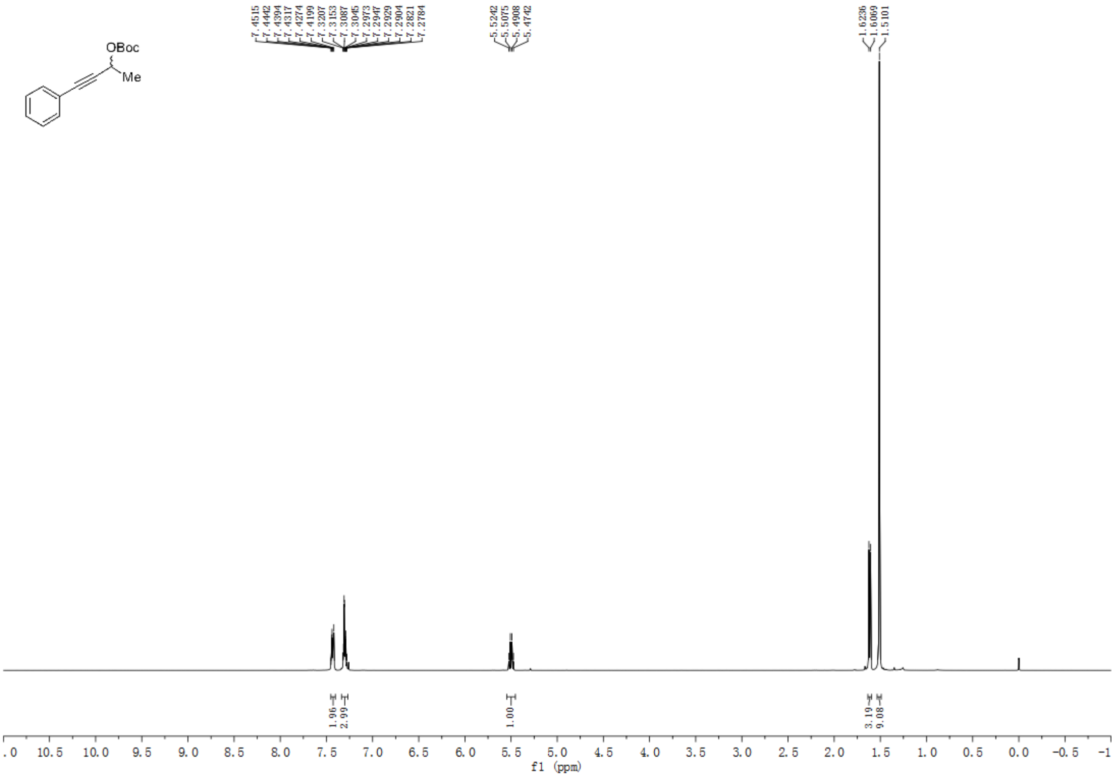

^{13}C NMR spectrum of **1i**

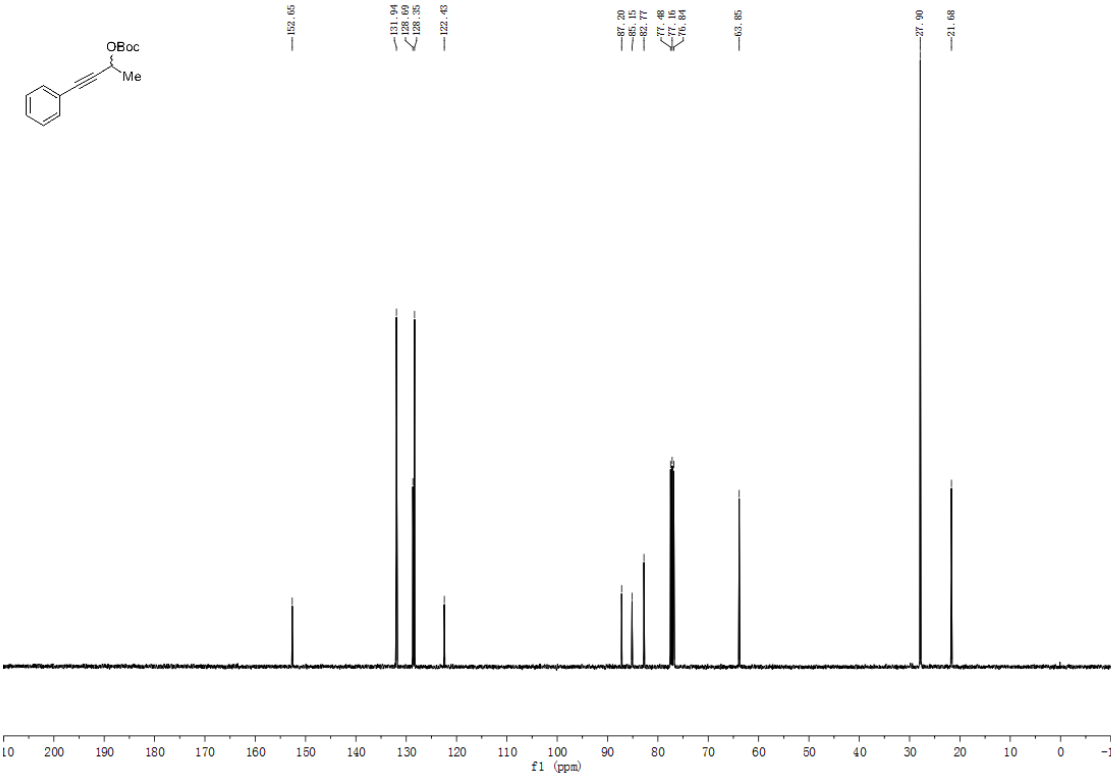

^{1}H NMR spectrum of **1j**

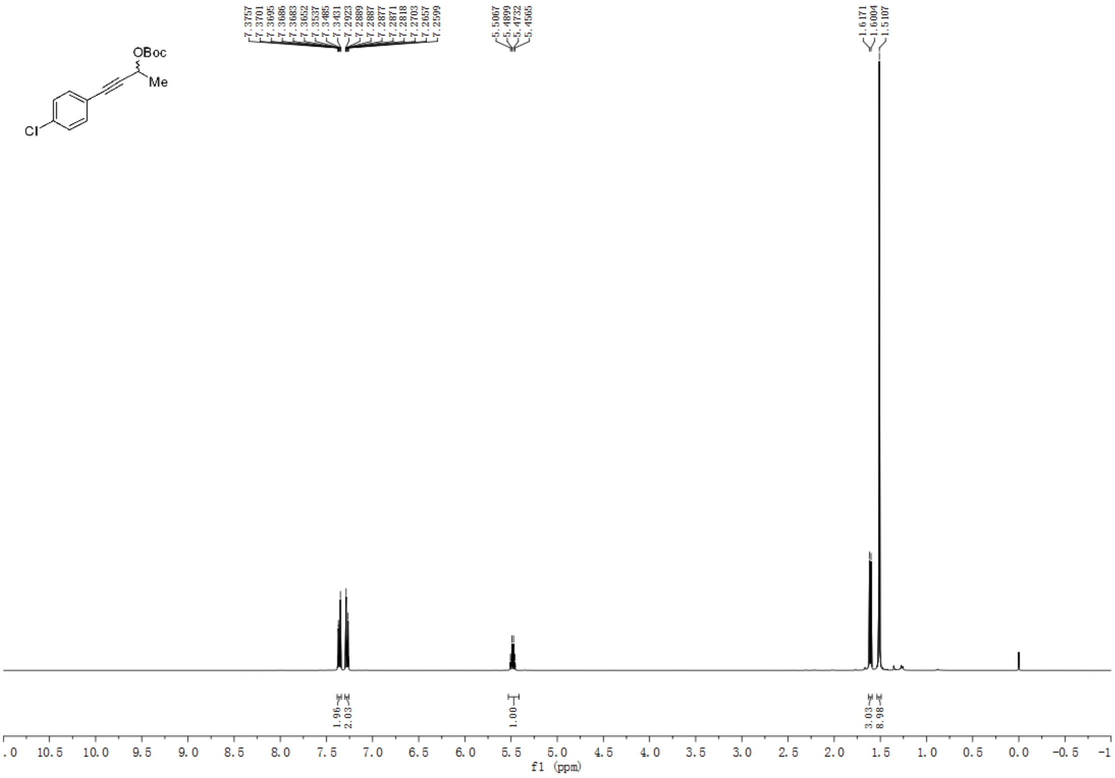

^{13}C NMR spectrum of **1j**

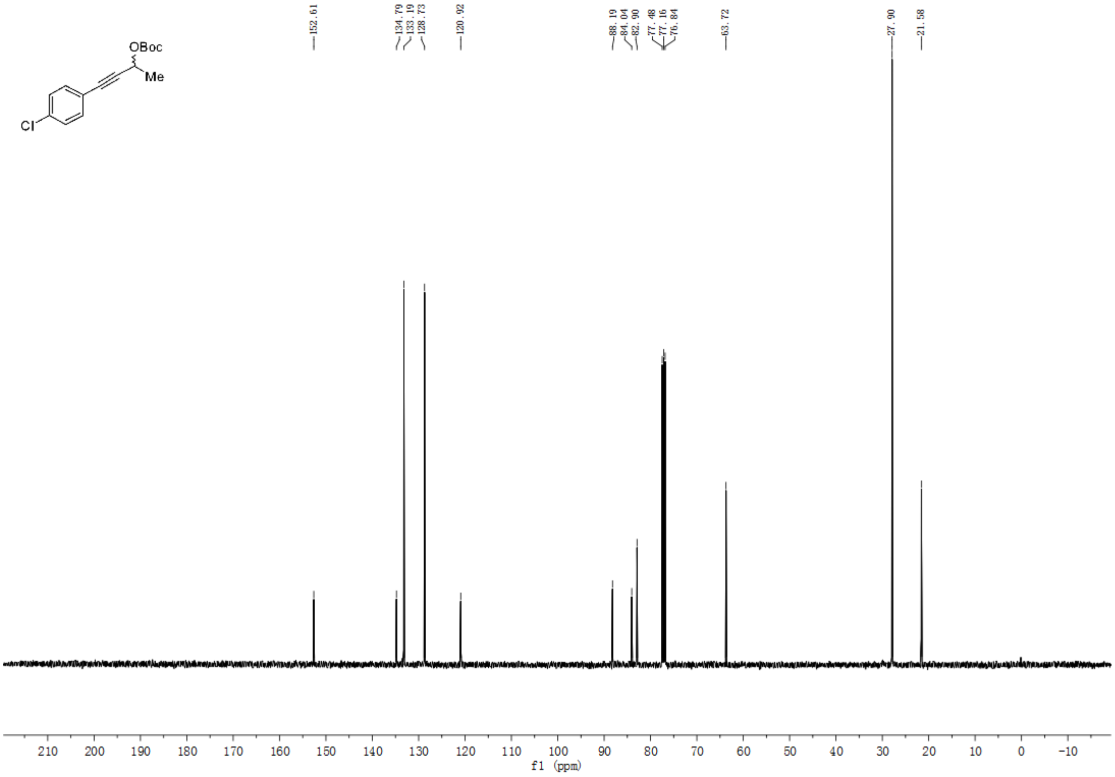

^{1}H NMR spectrum of **1k**

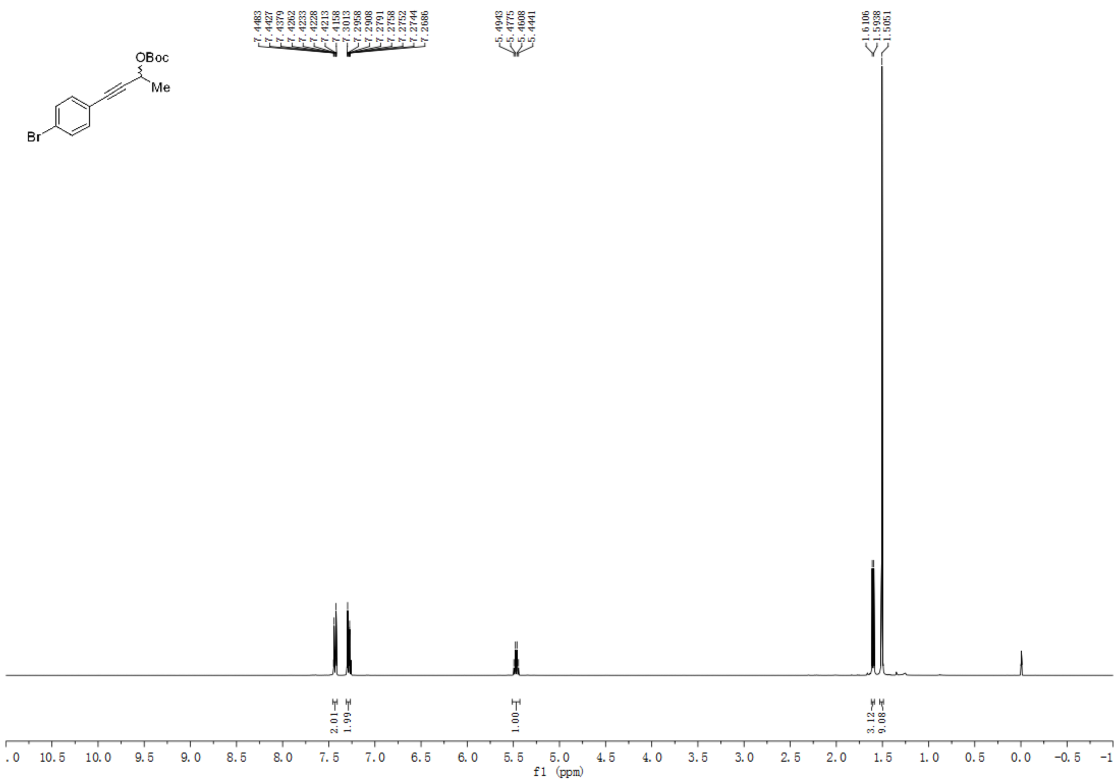

^{13}C NMR spectrum of **1k**

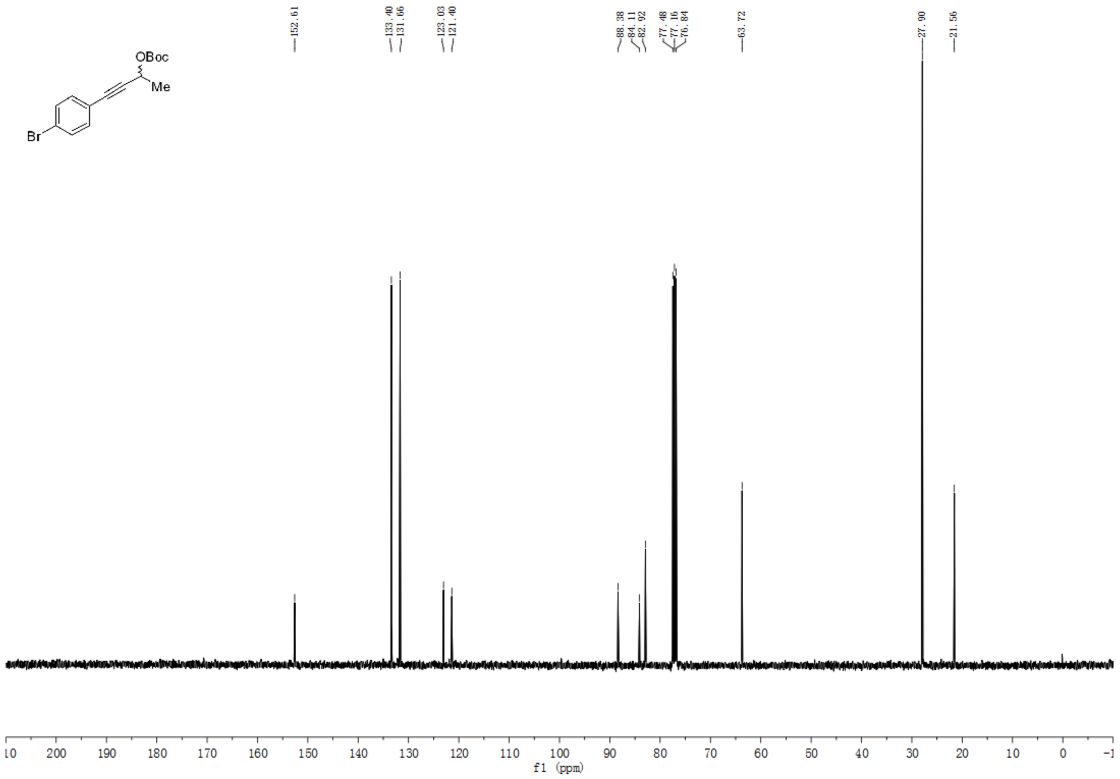

^{1}H NMR spectrum of **1l**

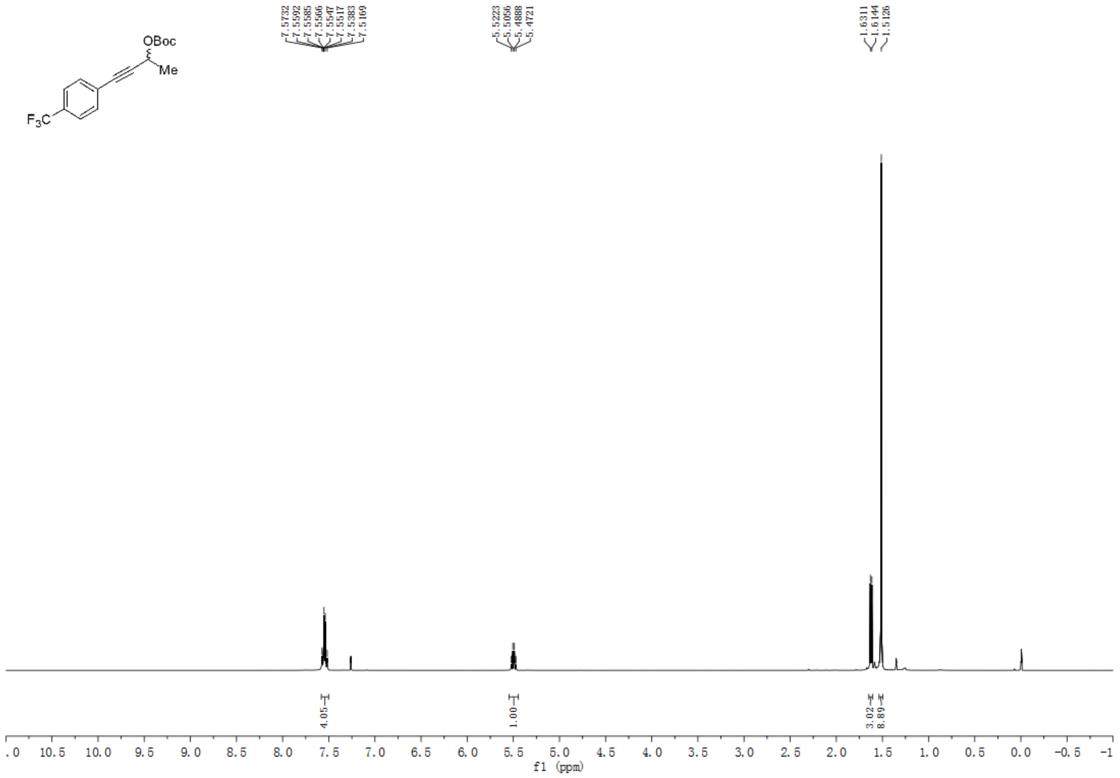

^{13}C NMR spectrum of **1l**

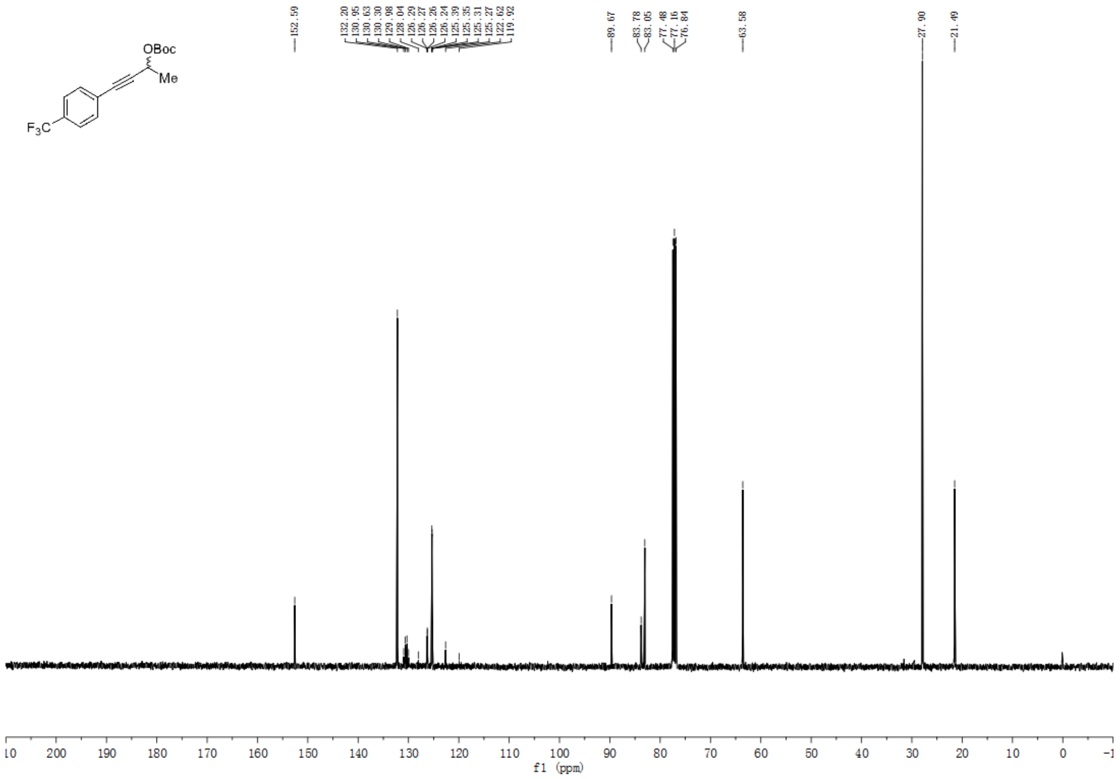

^{19}F NMR spectrum of **1l**

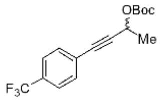

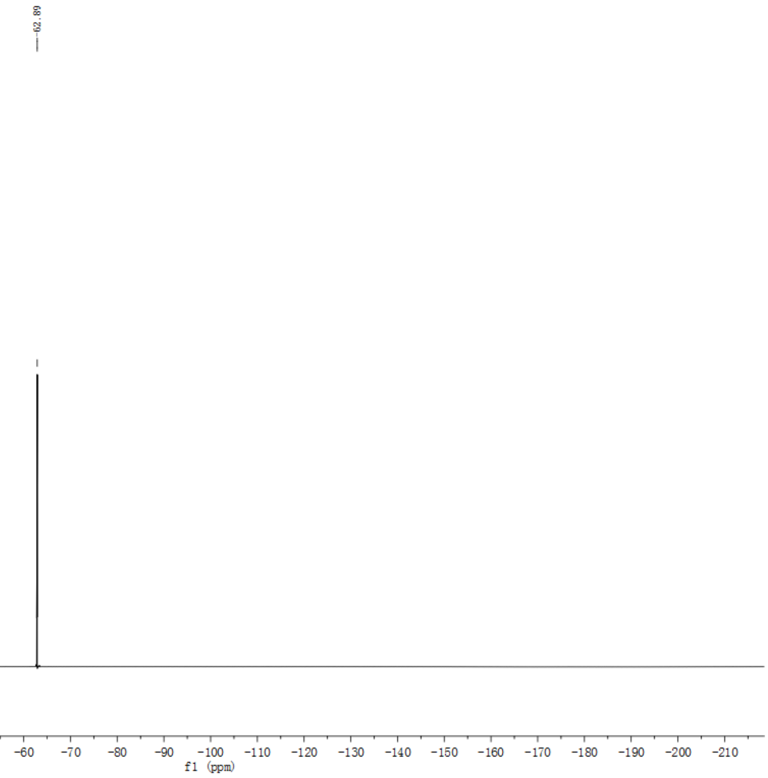

^{1}H NMR spectrum of **1m**

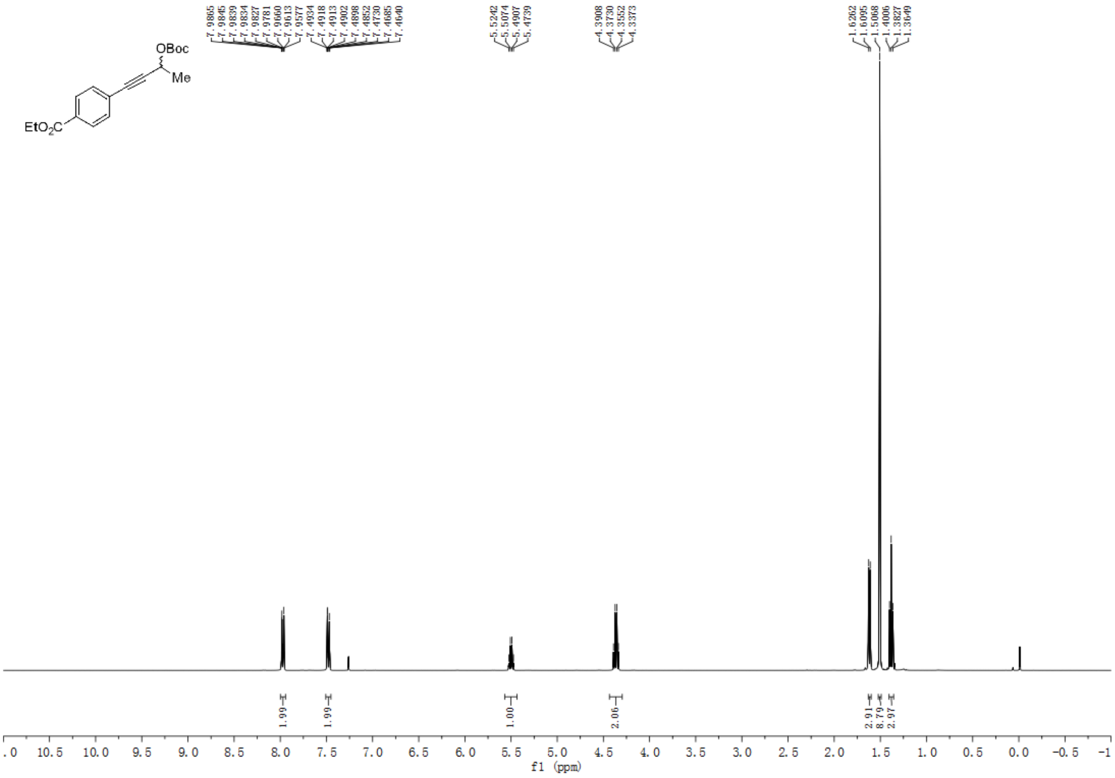

^{13}C NMR spectrum of **1m**

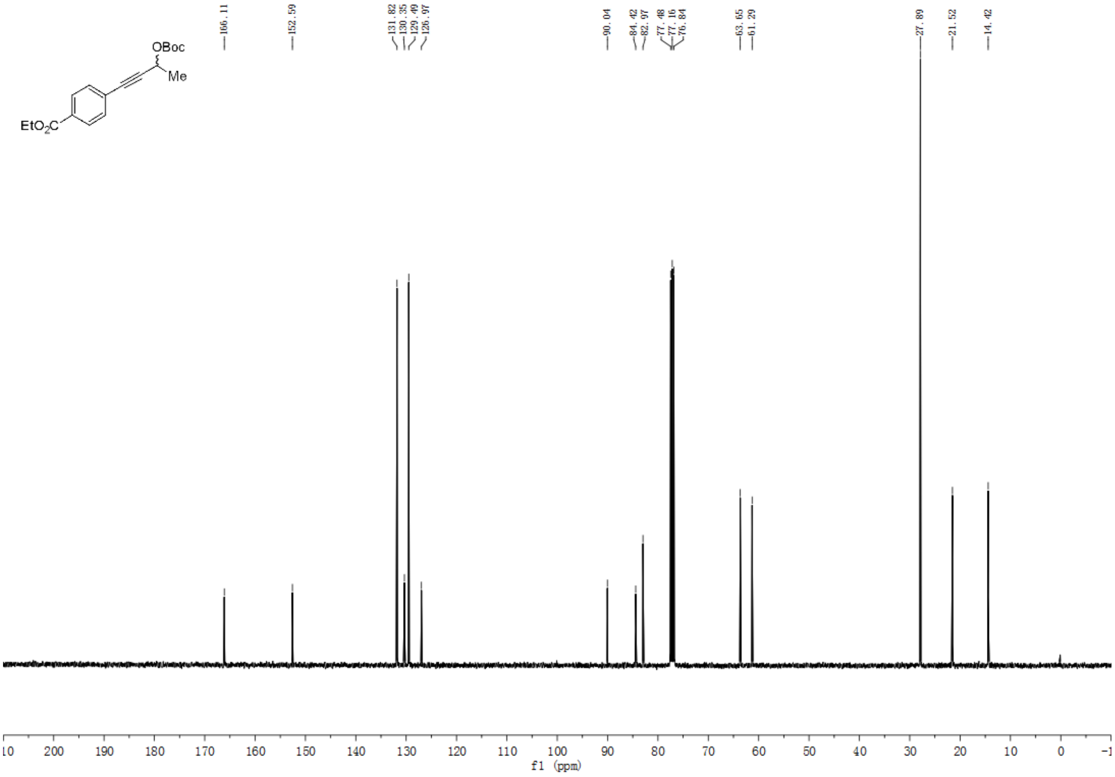

^{1}H NMR spectrum of **1n**

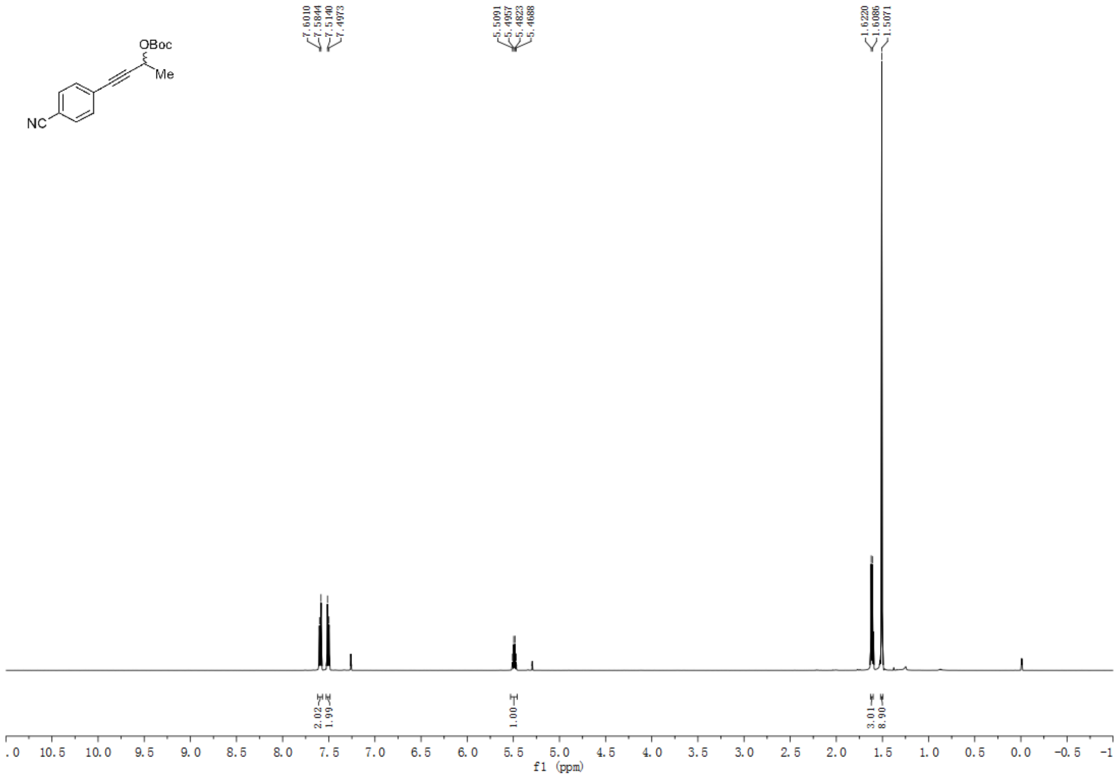

^{13}C NMR spectrum of **1n**

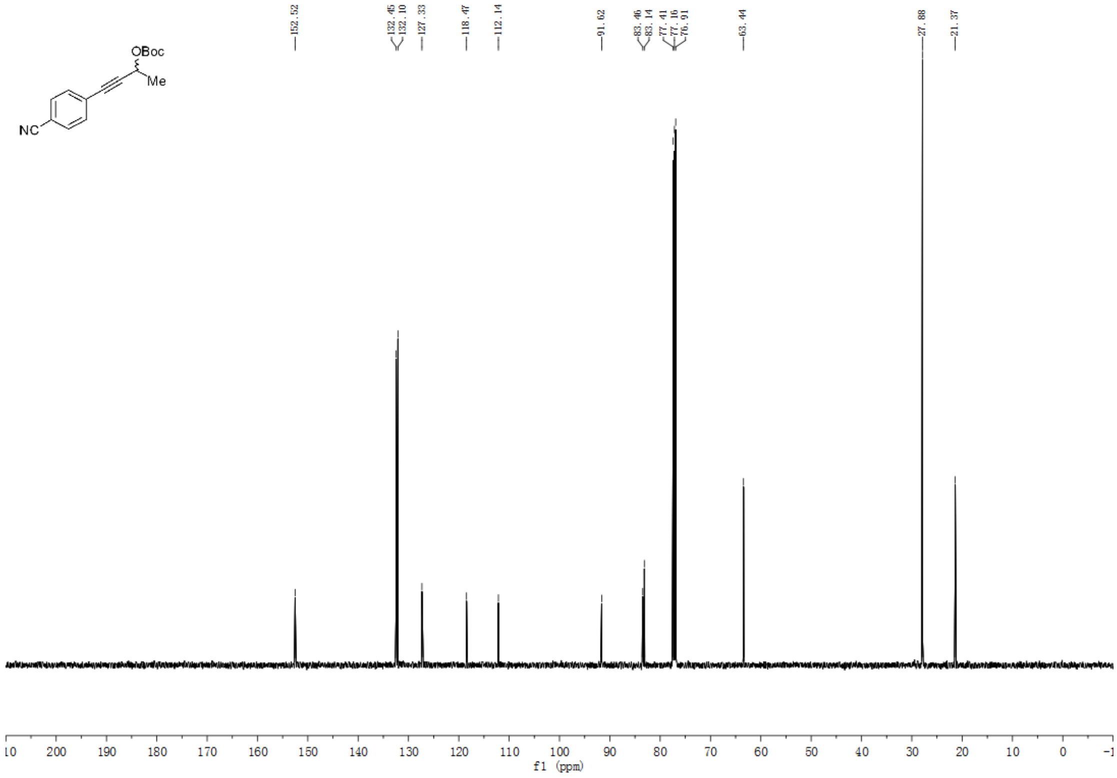

^{1}H NMR spectrum of **1o**

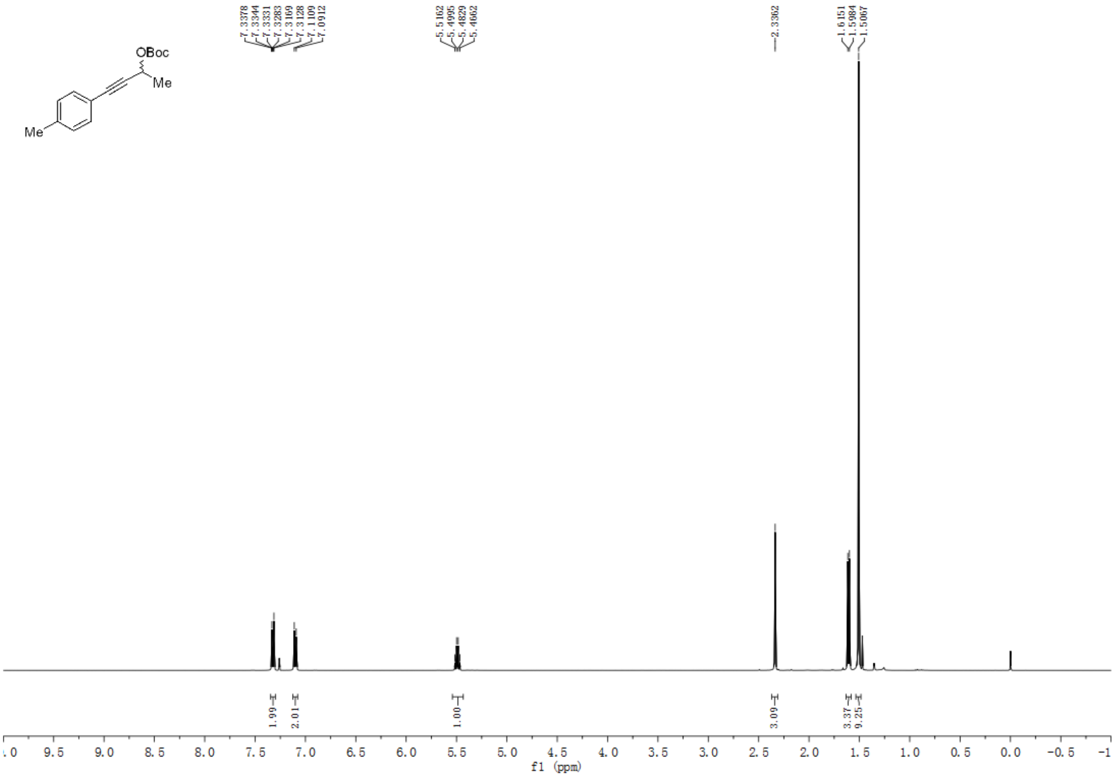

^{13}C NMR spectrum of **1o**

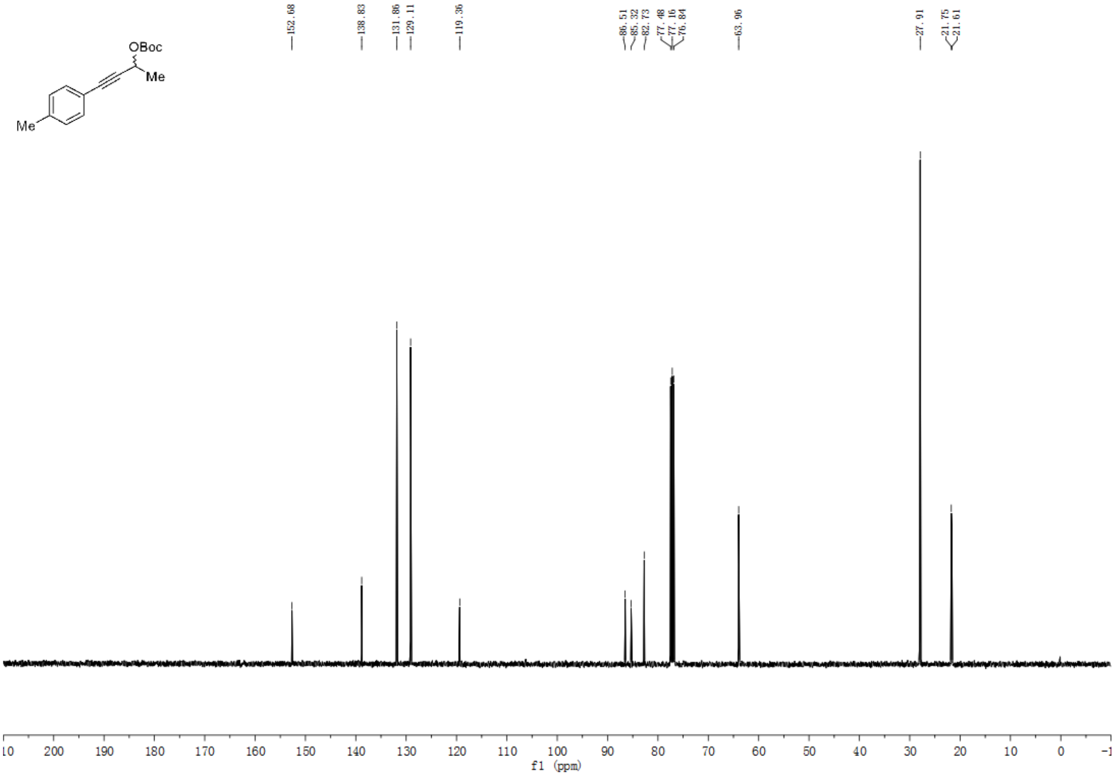

^{1}H NMR spectrum of **1p**

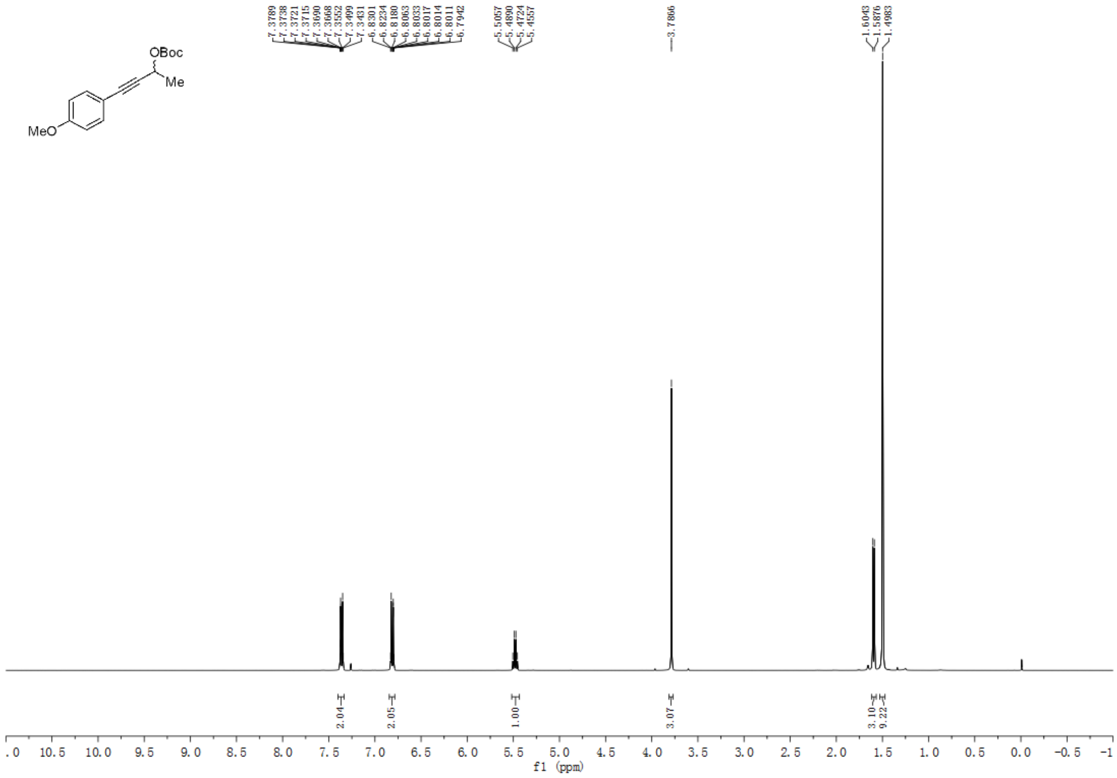

^{13}C NMR spectrum of **1p**

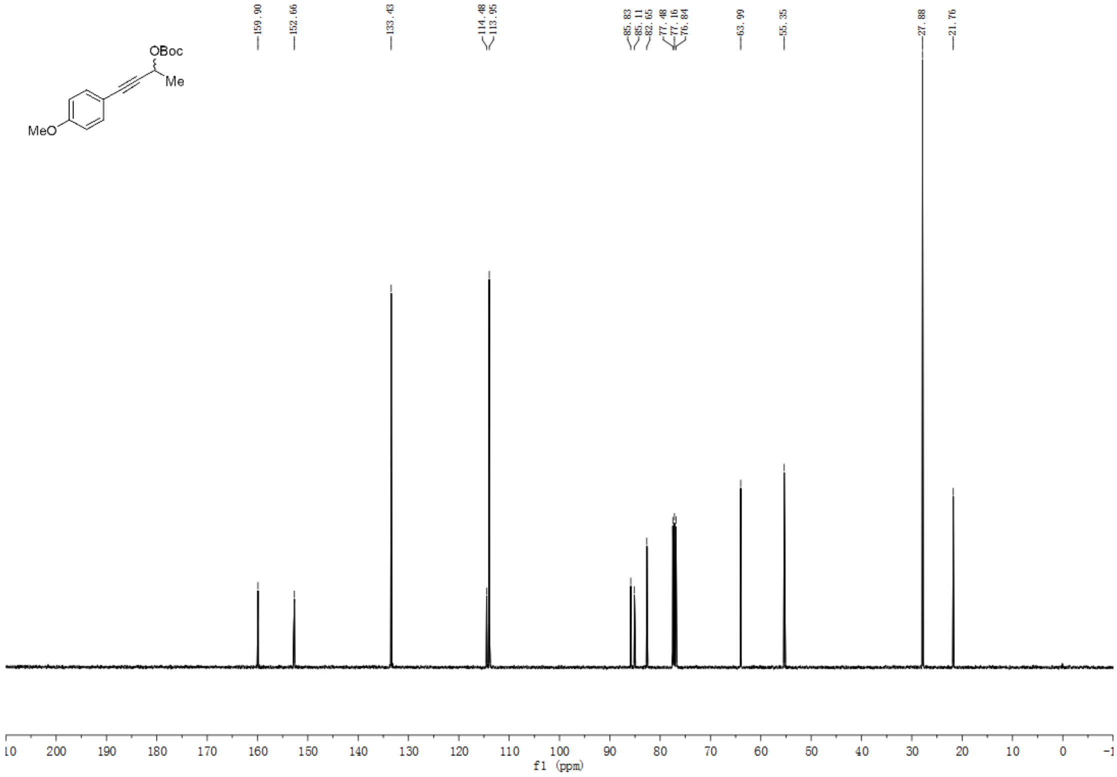

^{1}H NMR spectrum of **1q**

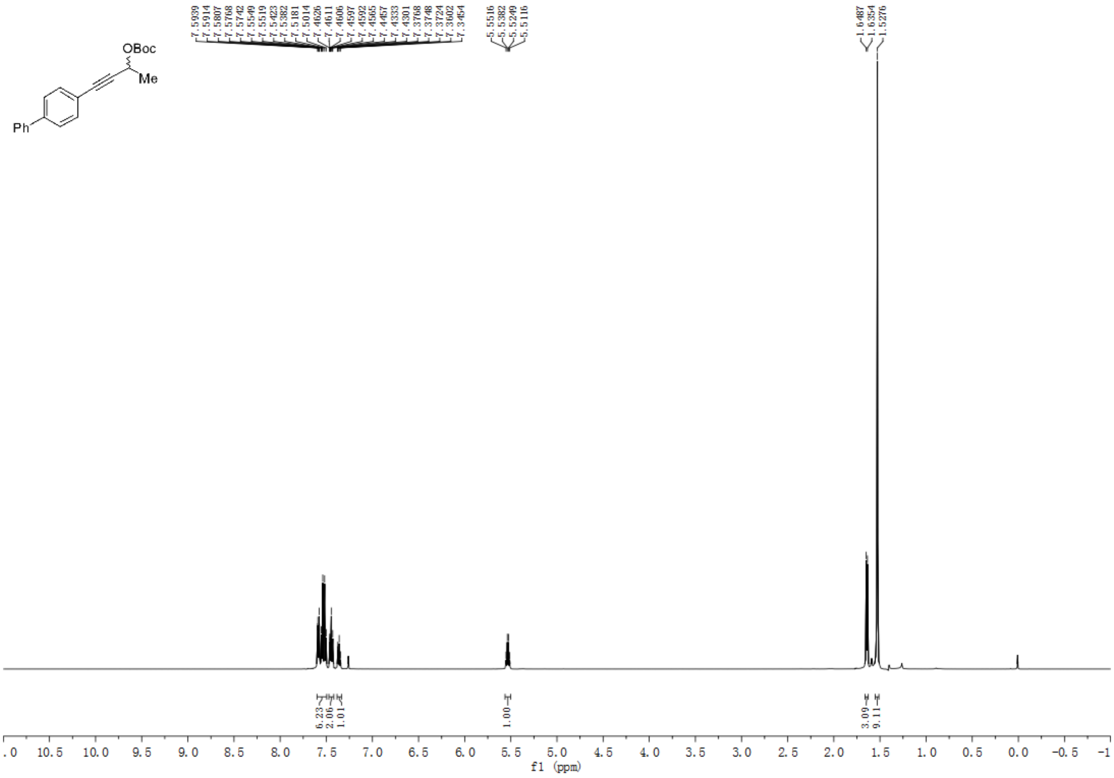

^{13}C NMR spectrum of **1q**

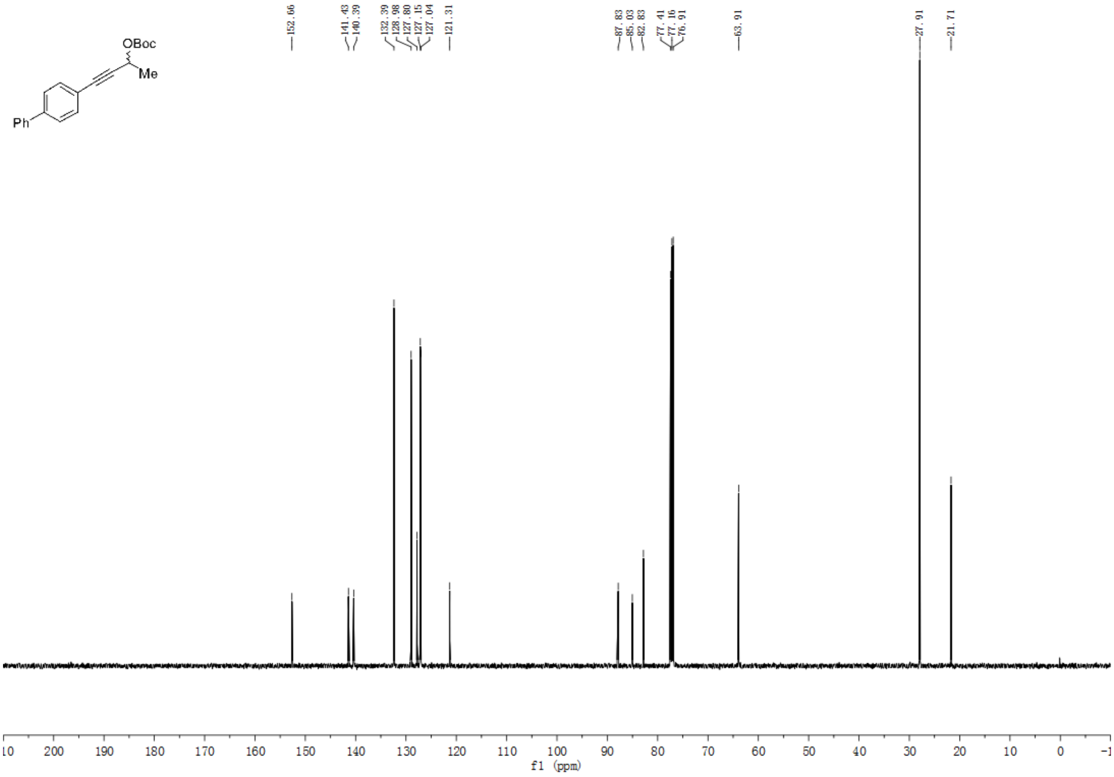

^{1}H NMR spectrum of **1r**

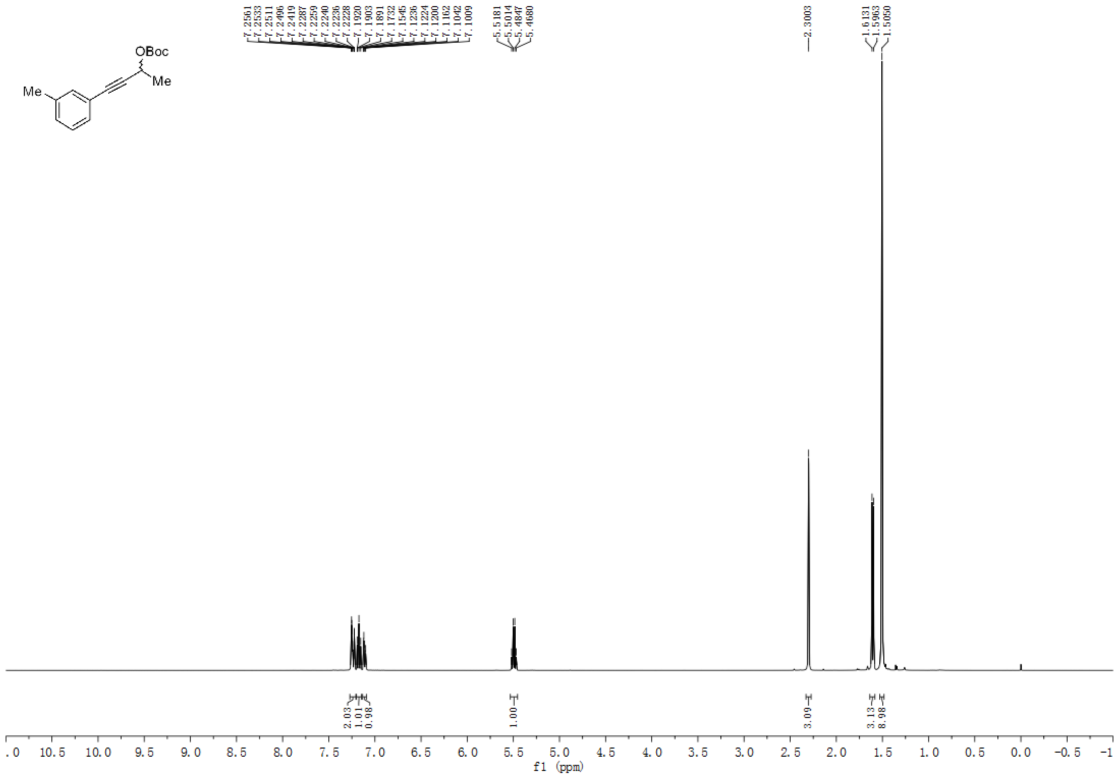

^{13}C NMR spectrum of **1r**

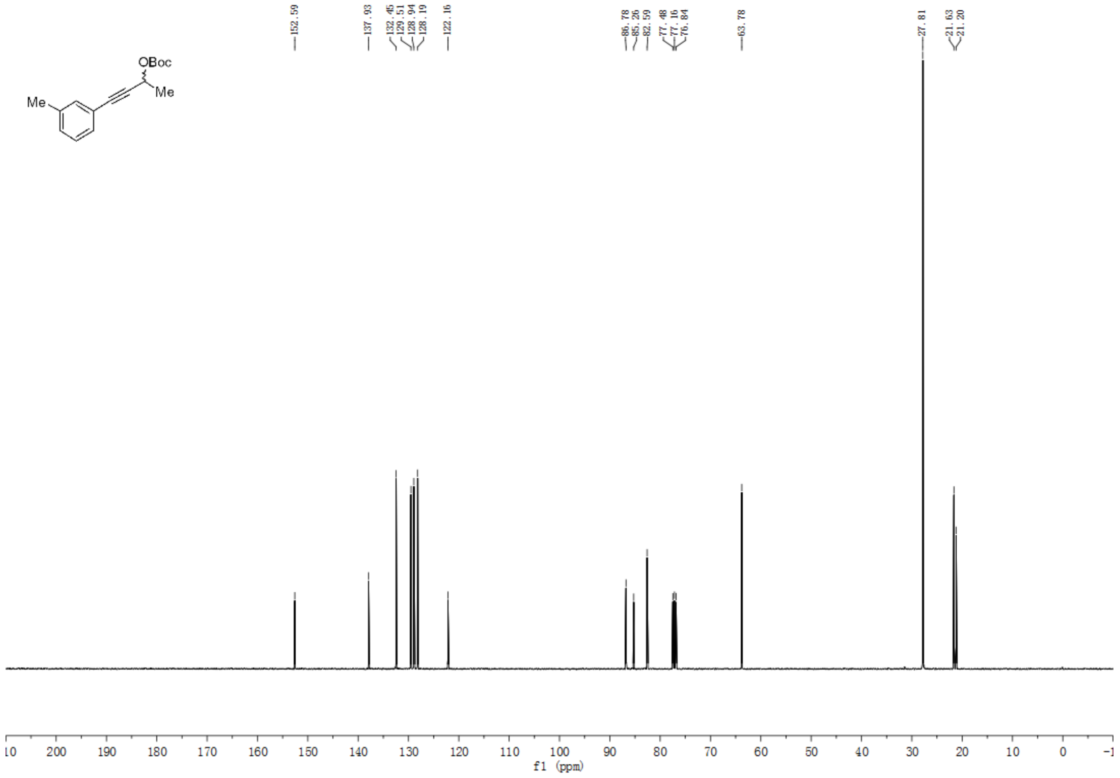

^{1}H NMR spectrum of **1s**

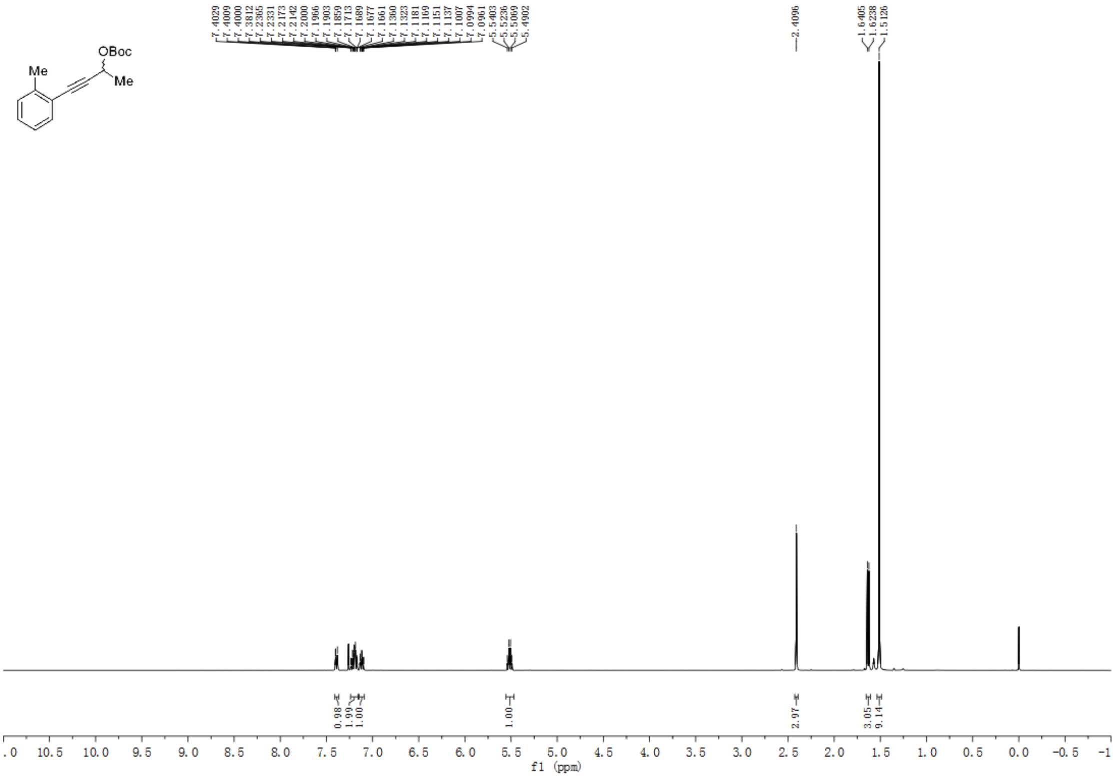

^{13}C NMR spectrum of **1s**

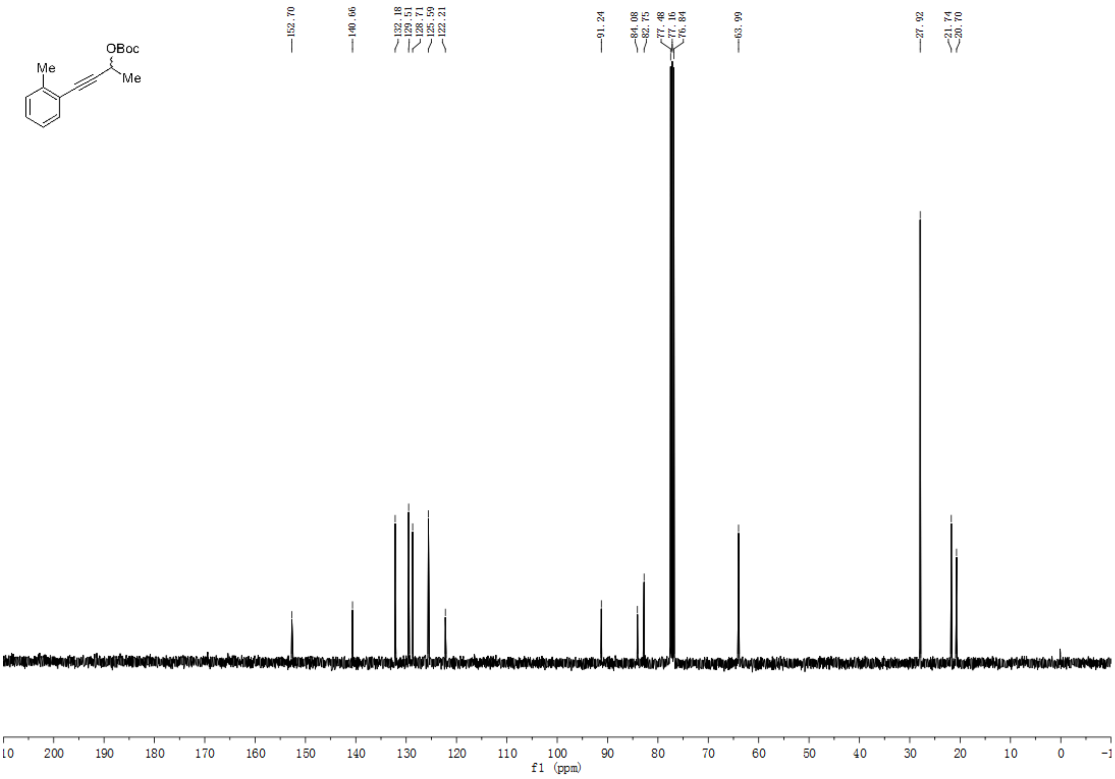

^{1}H NMR spectrum of **1t**

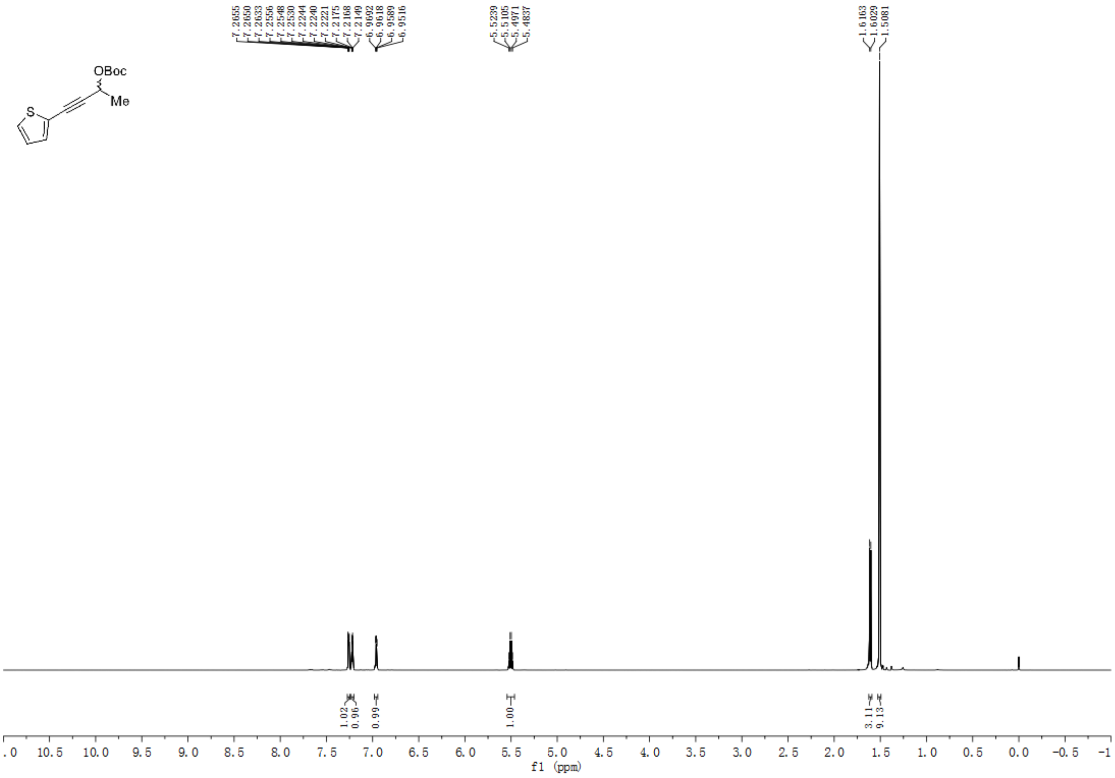

^{13}C NMR spectrum of **1t**

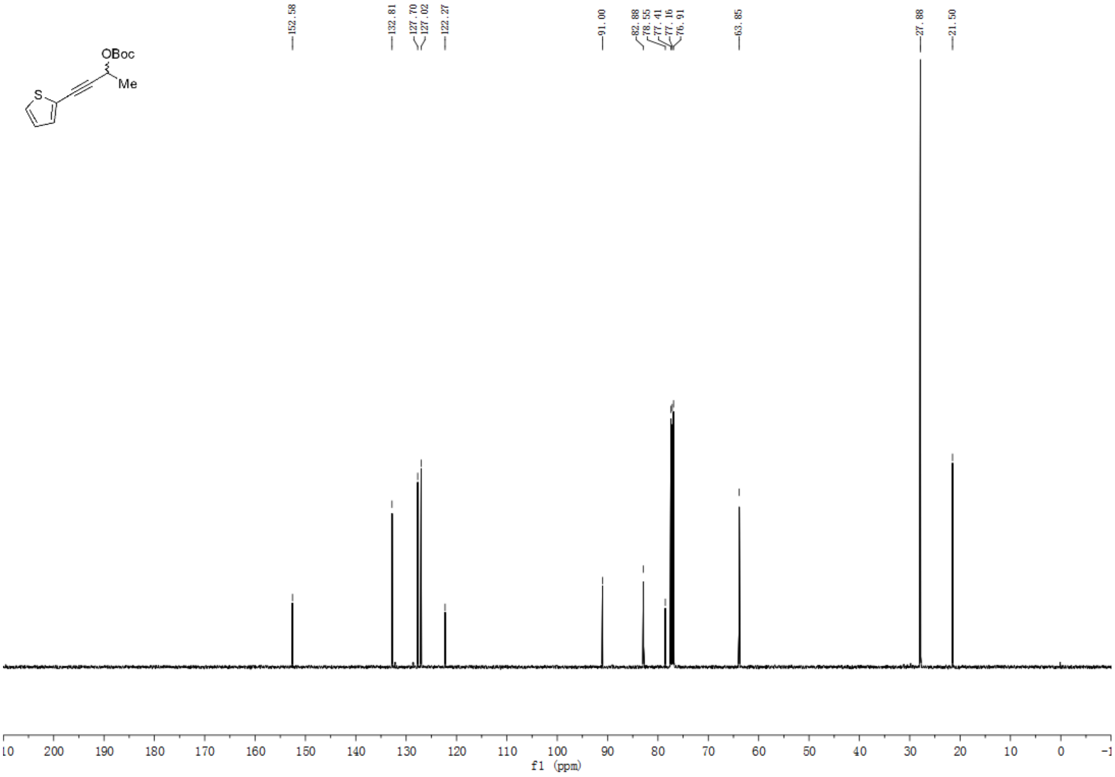

^{1}H NMR spectrum of **1u**

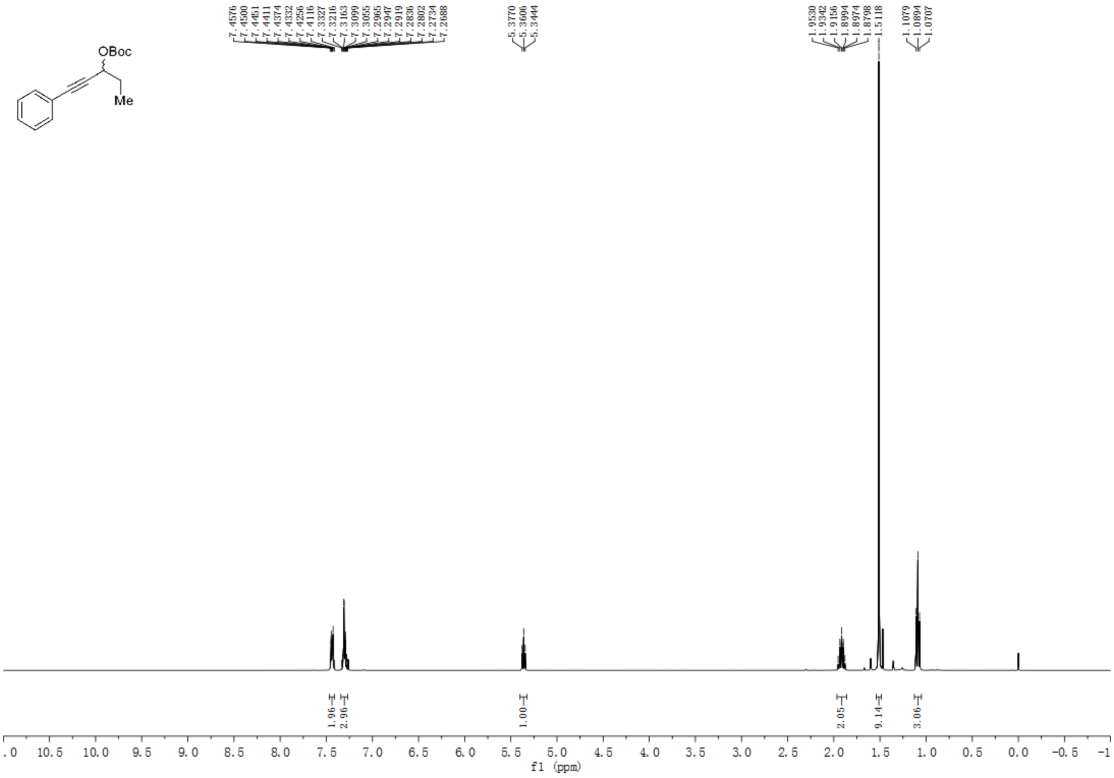

^{13}C NMR spectrum of **1u**

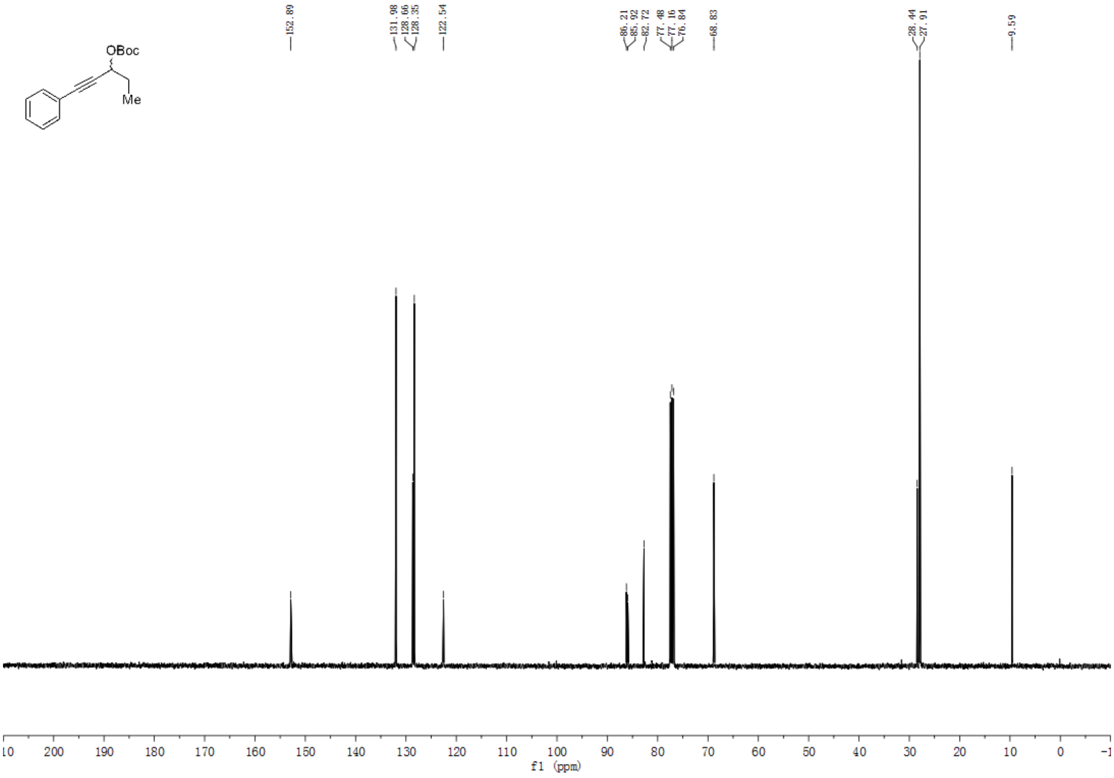

^{1}H NMR spectrum of **1v**

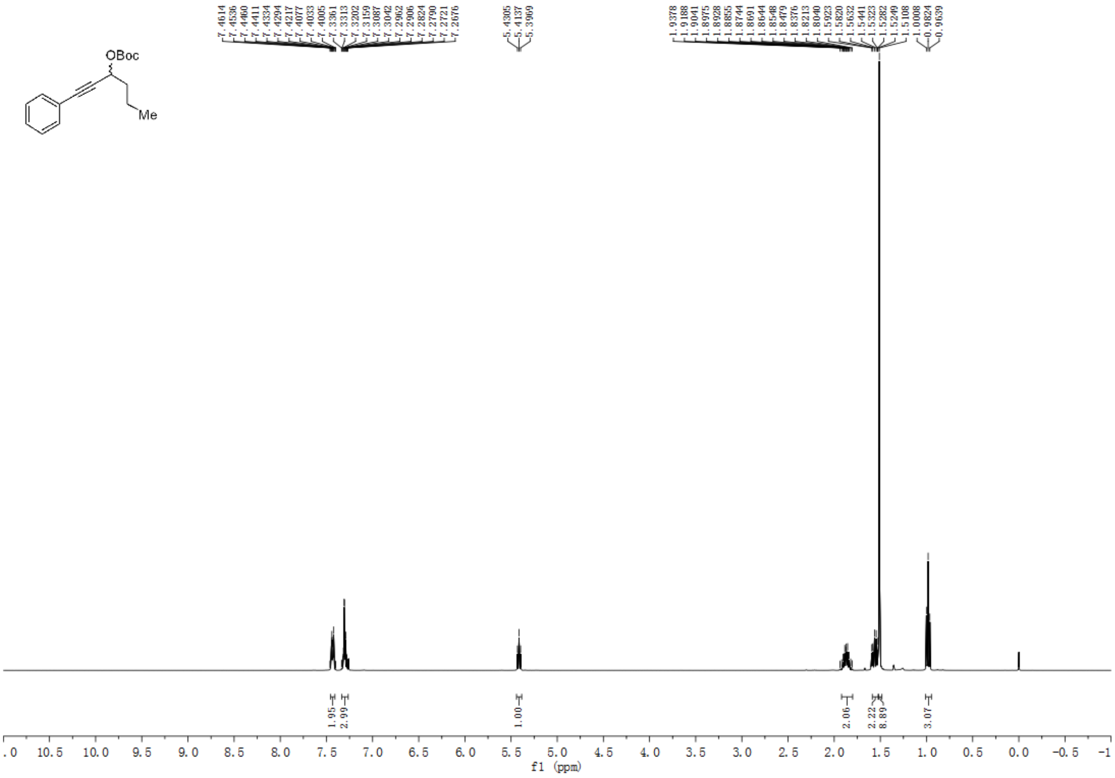

^{13}C NMR spectrum of **1v**

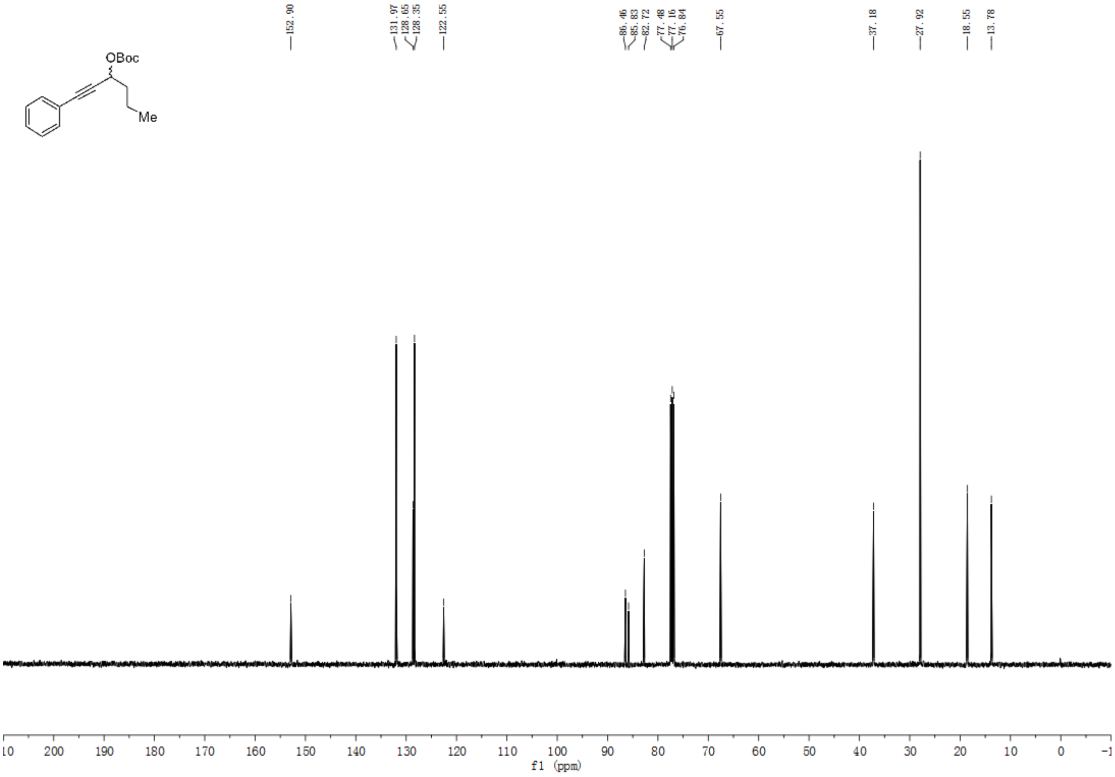

^{1}H NMR spectrum of **1w**

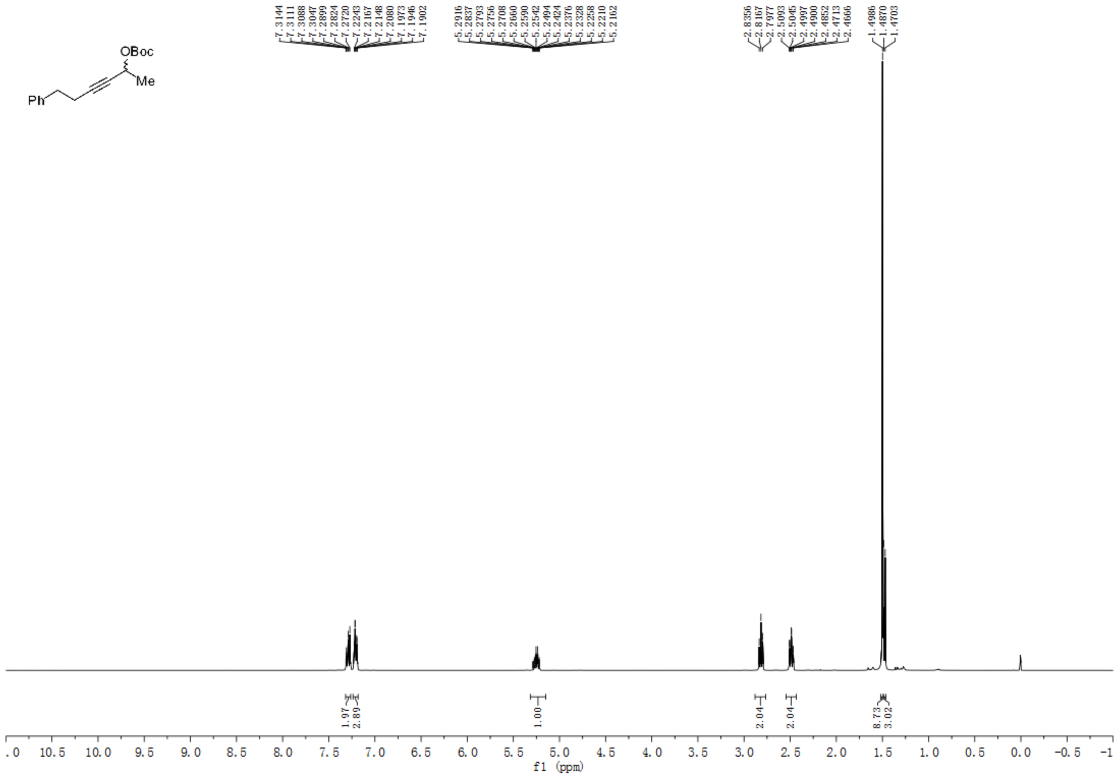

^{13}C NMR spectrum of **1w**

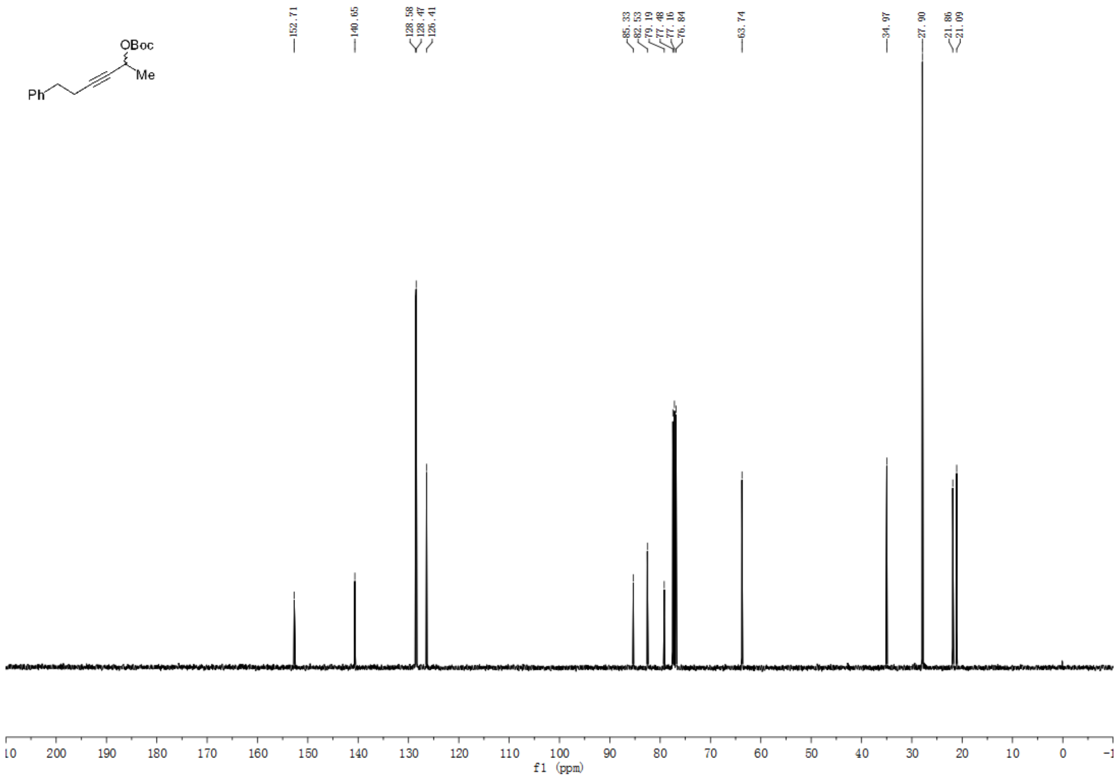

^{1}H NMR spectrum of **1x**

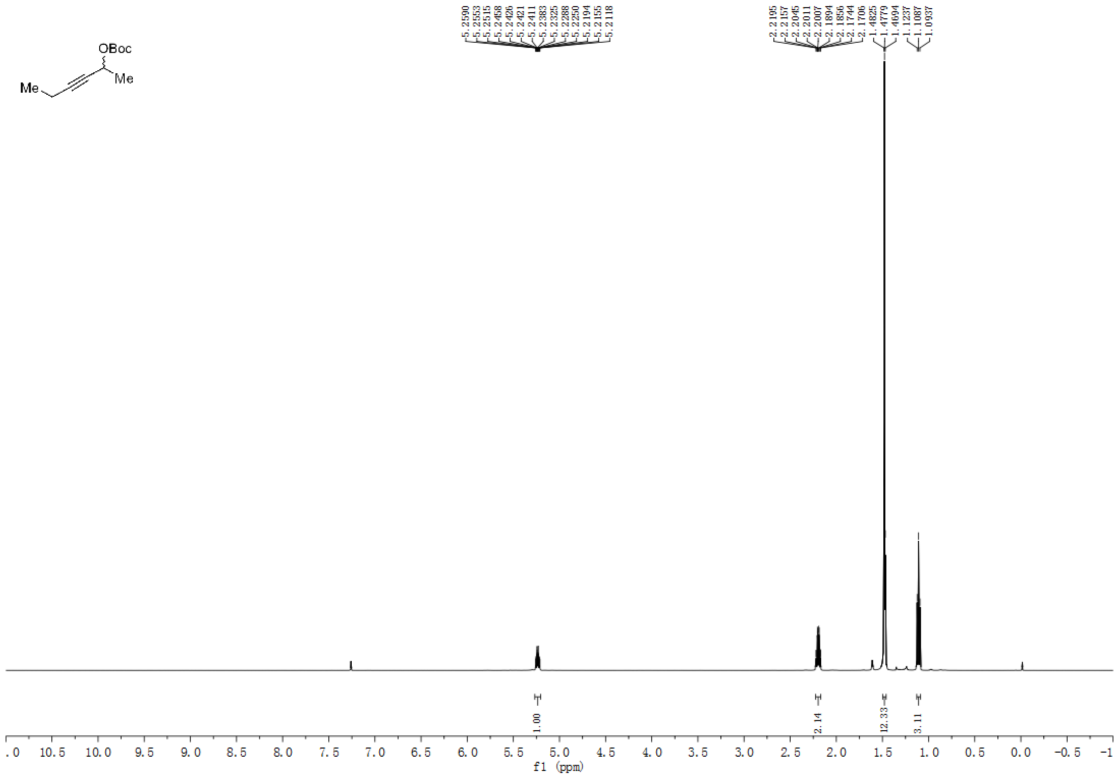

^{13}C NMR spectrum of **1x**

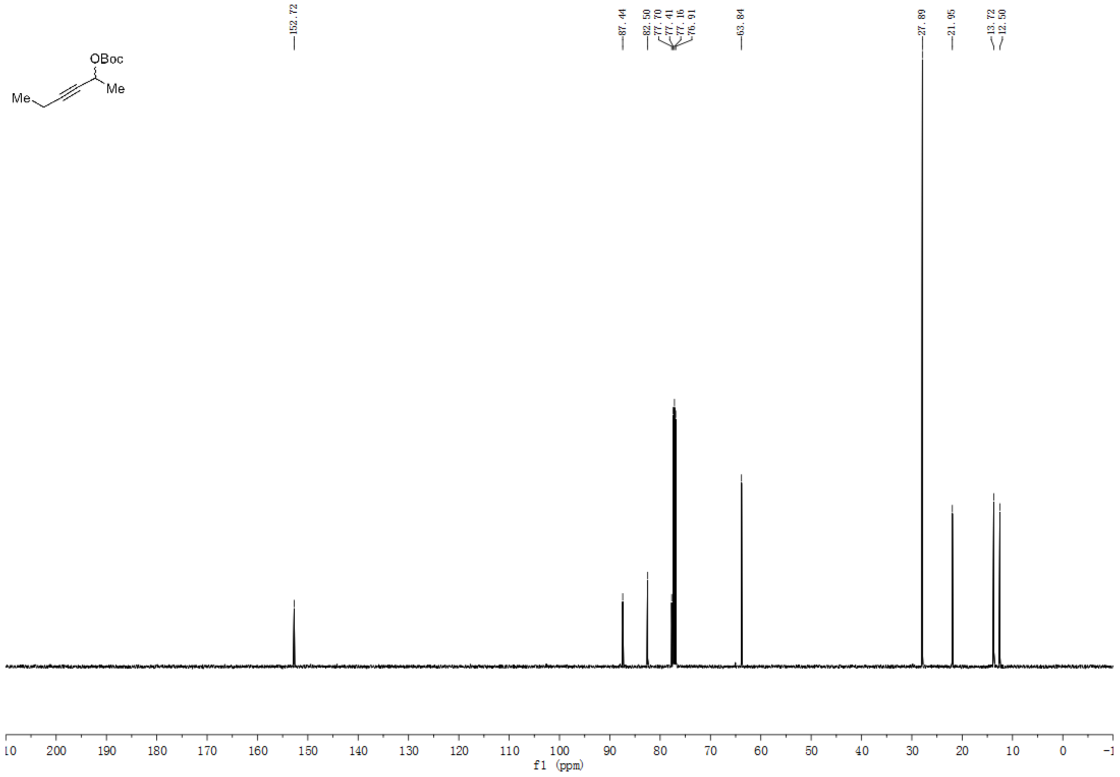

^{1}H NMR spectrum of **1y**

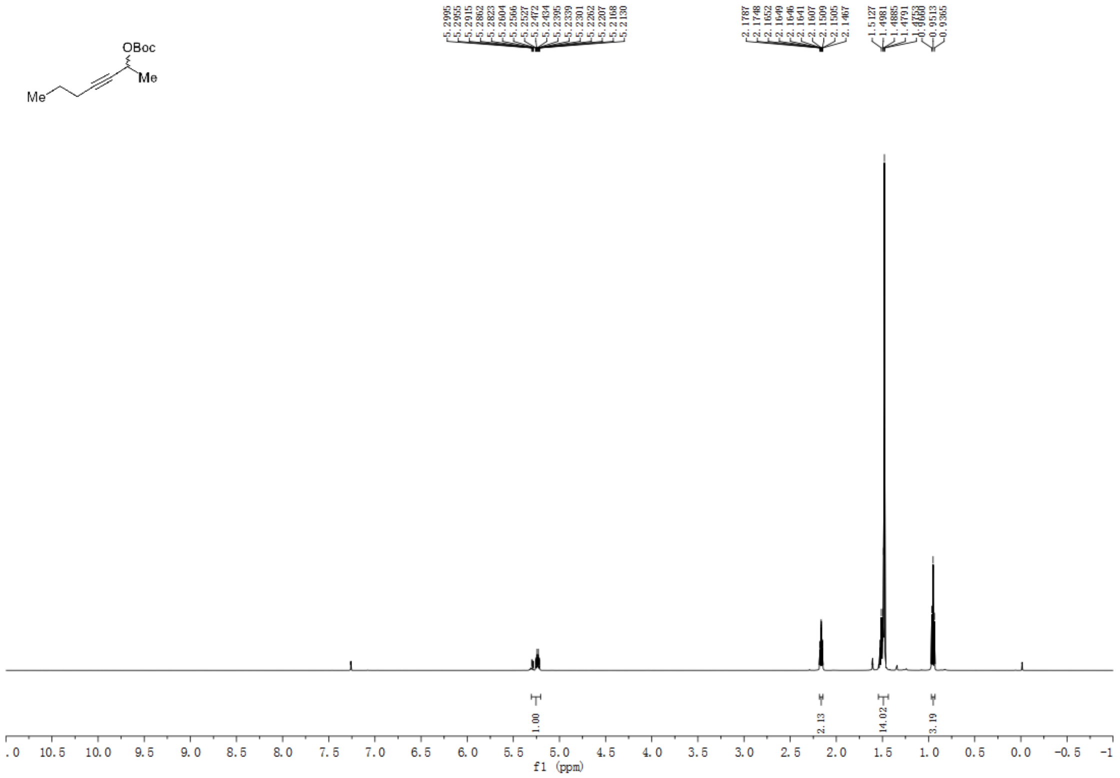

^{13}C NMR spectrum of **1y**

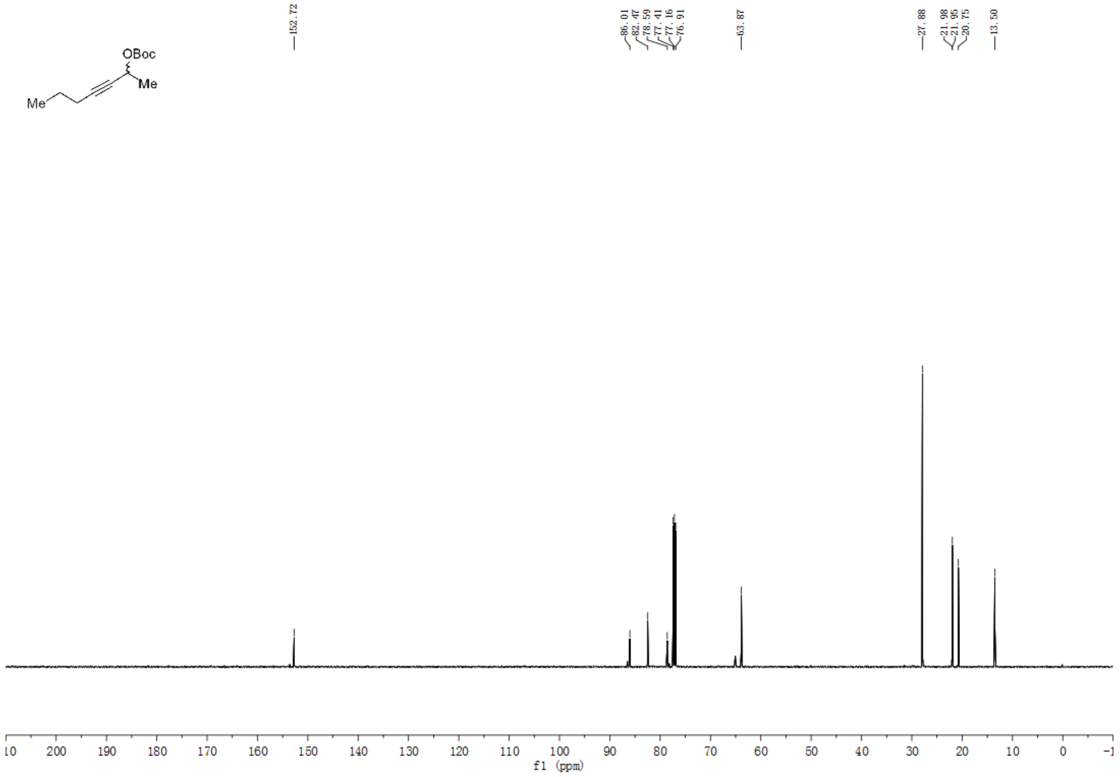

^{1}H NMR spectrum of **1z**

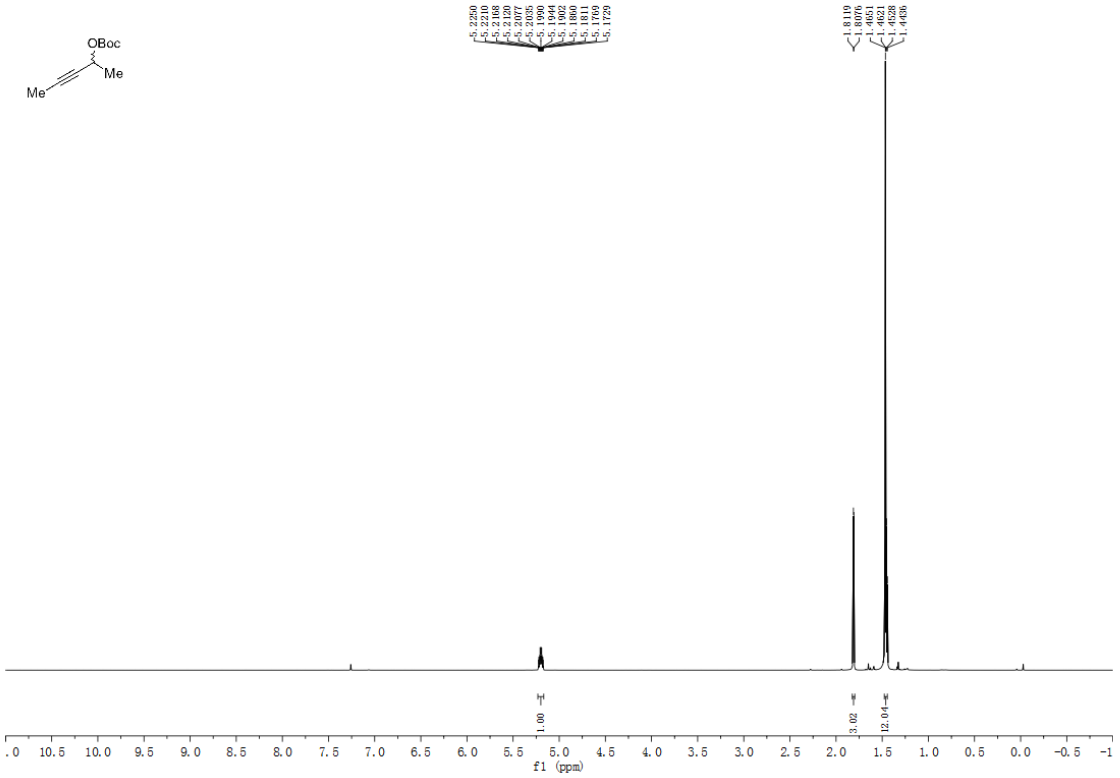

^{13}C NMR spectrum of **1z**

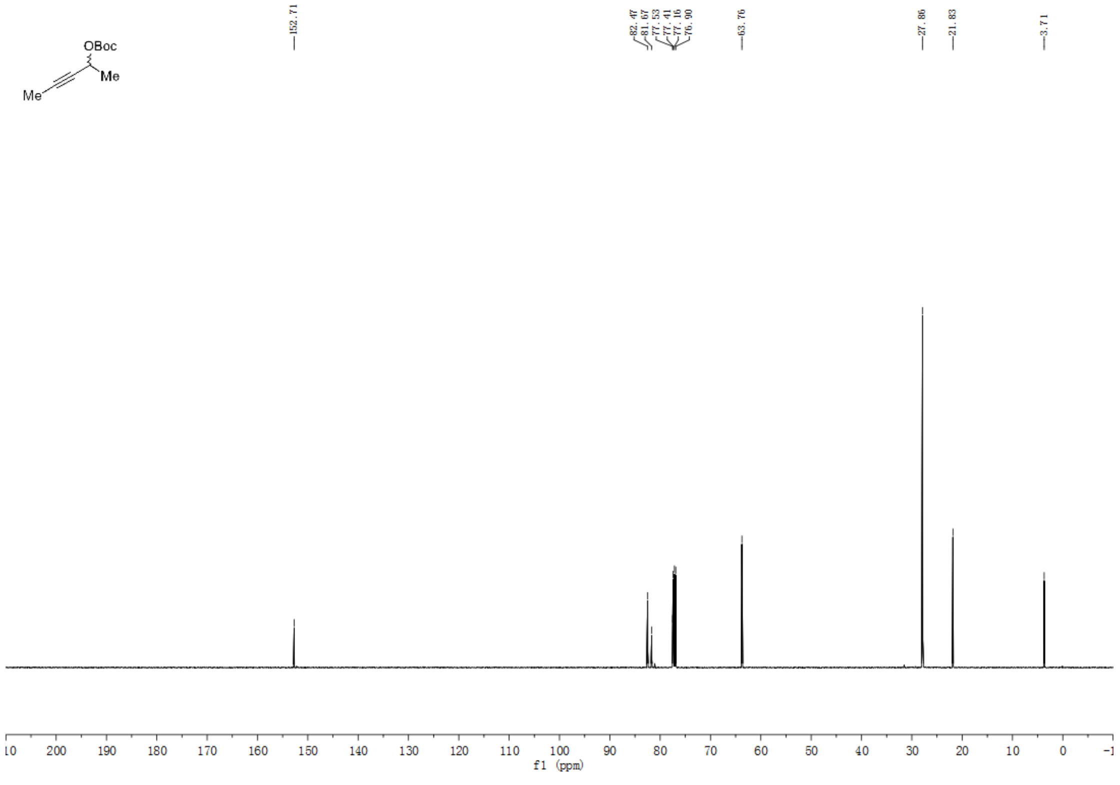

^{1}H NMR spectrum of **3a**

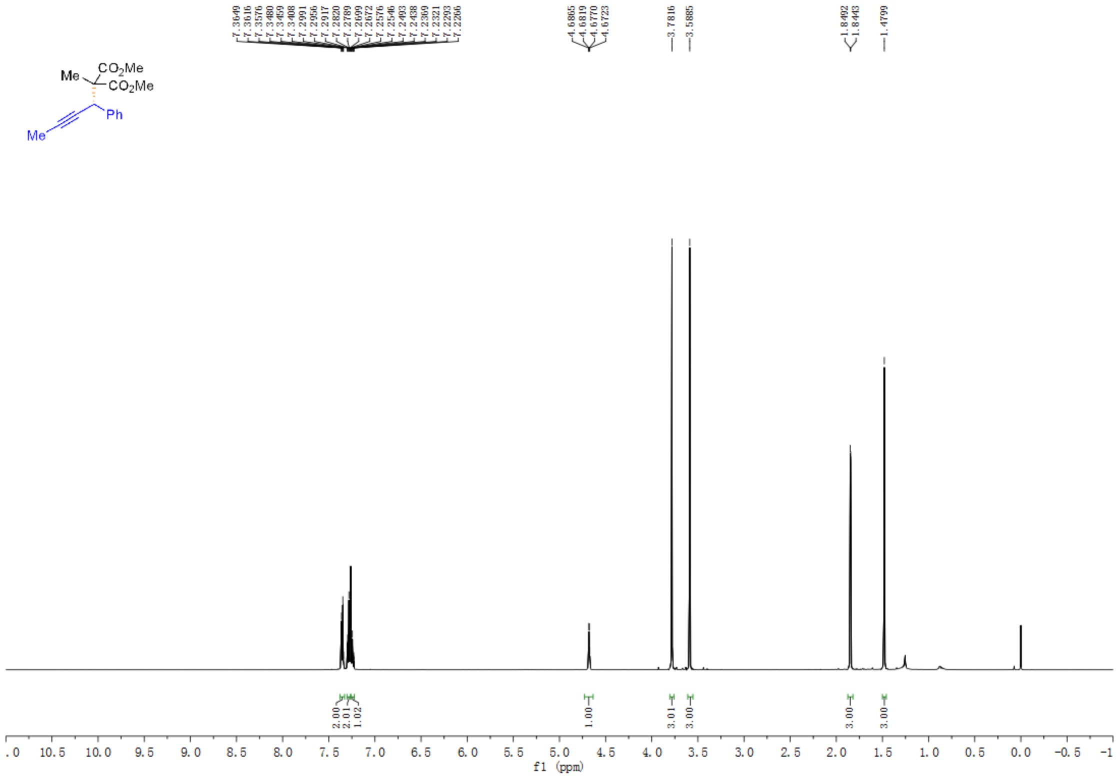

^{13}C NMR spectrum of **3a**

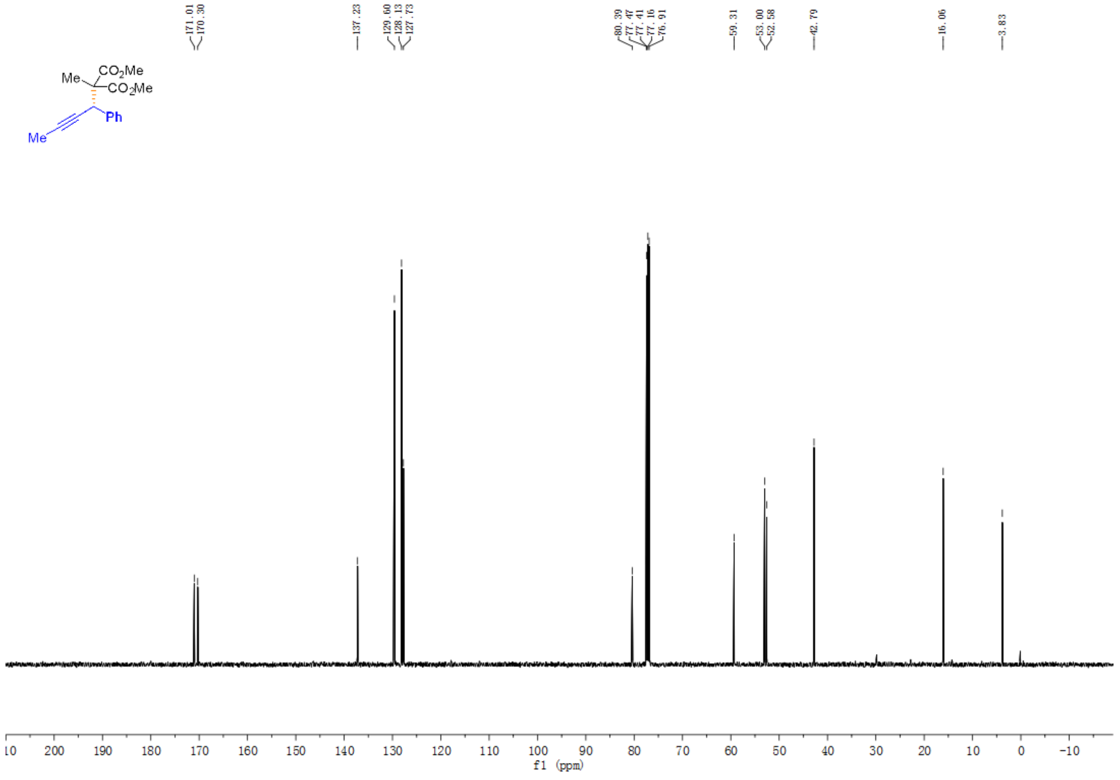

^{1}H NMR spectrum of **3b**

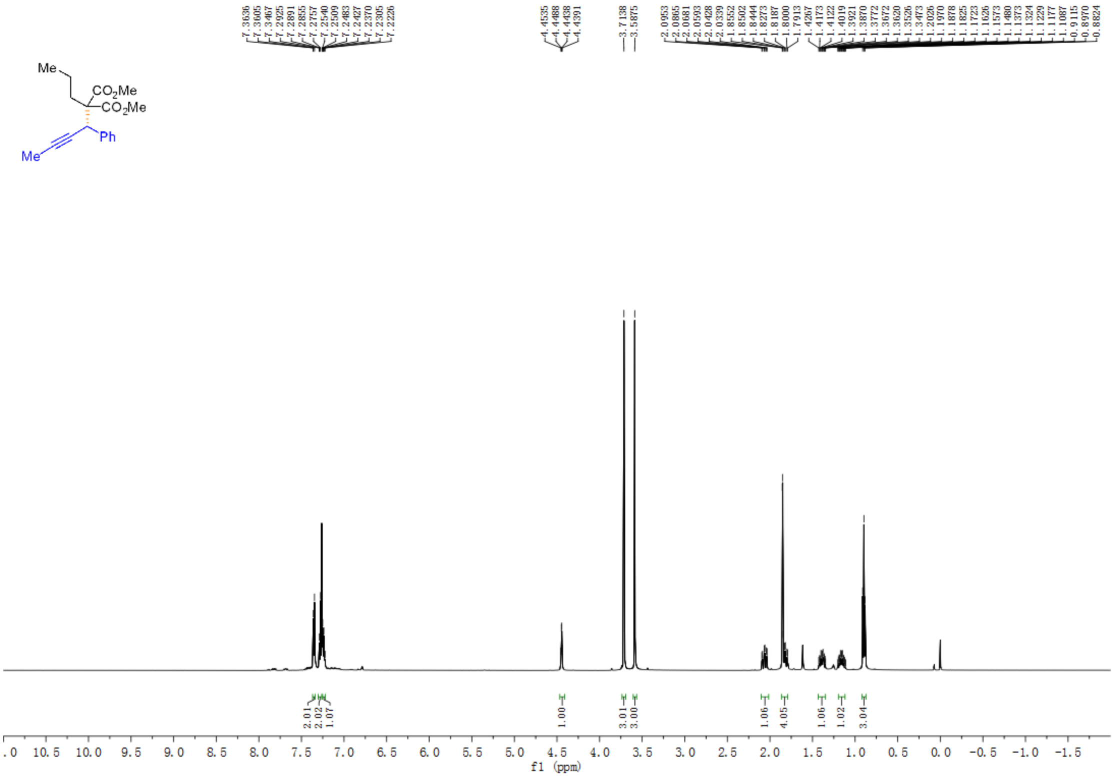

^{13}C NMR spectrum of **3b**

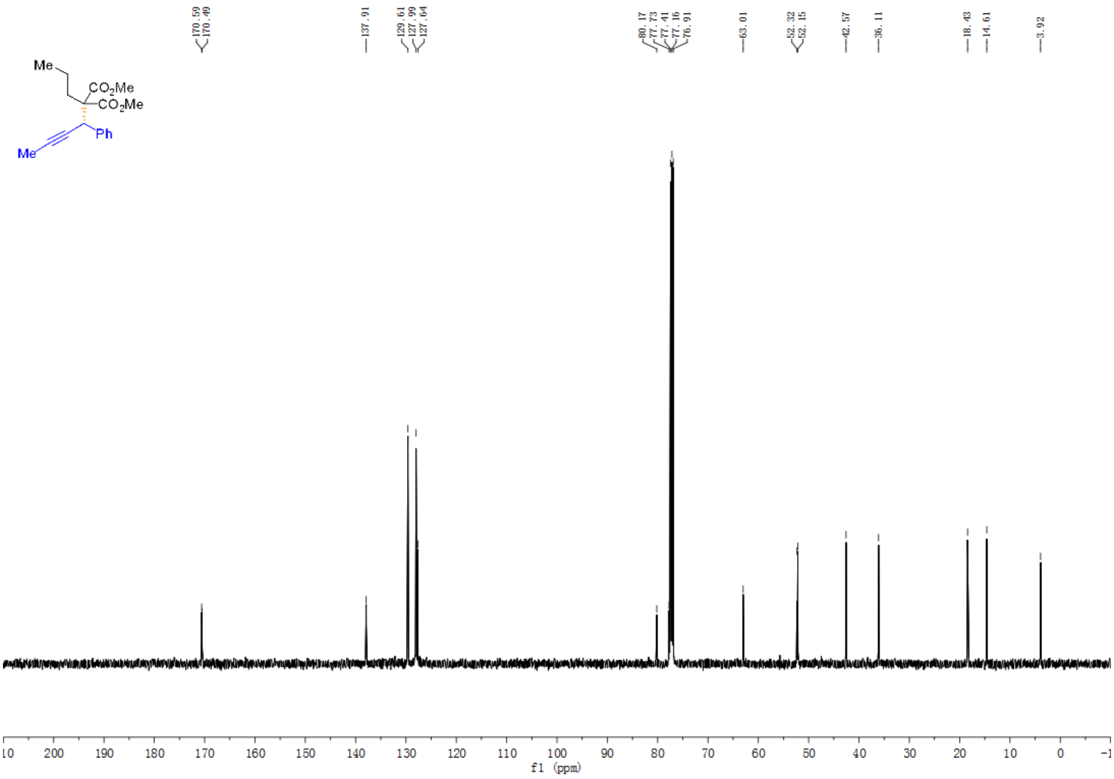

^{1}H NMR spectrum of **3c**

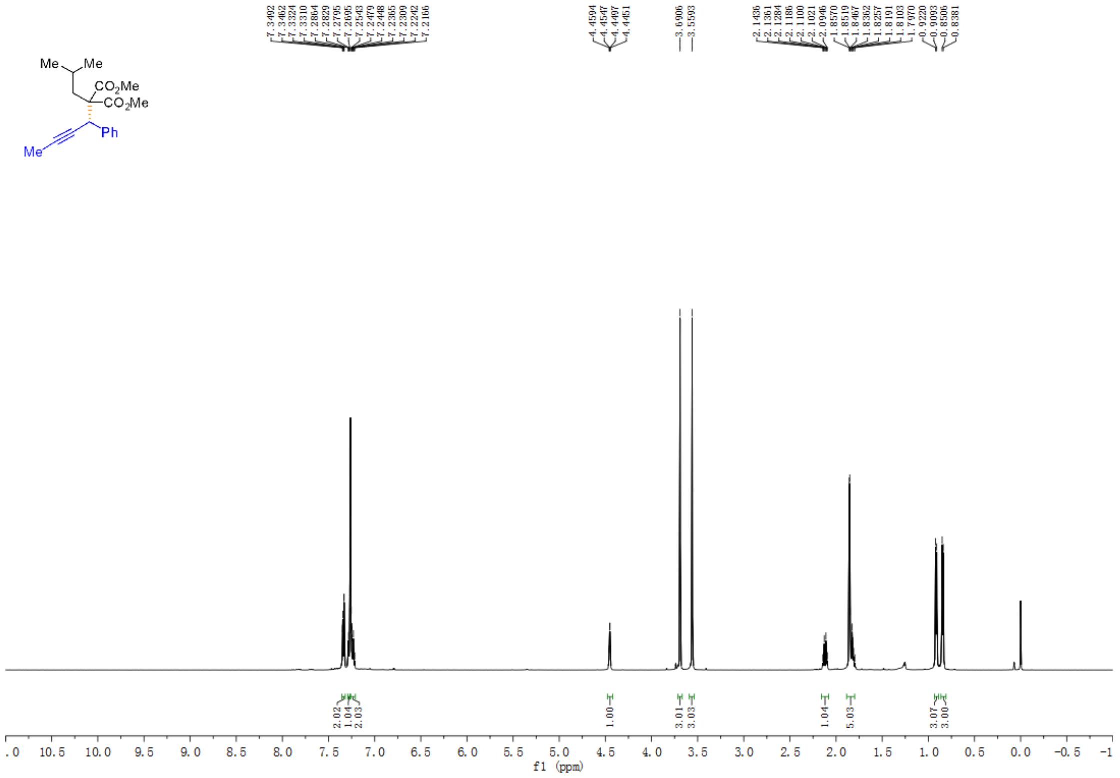

^{13}C NMR spectrum of **3c**

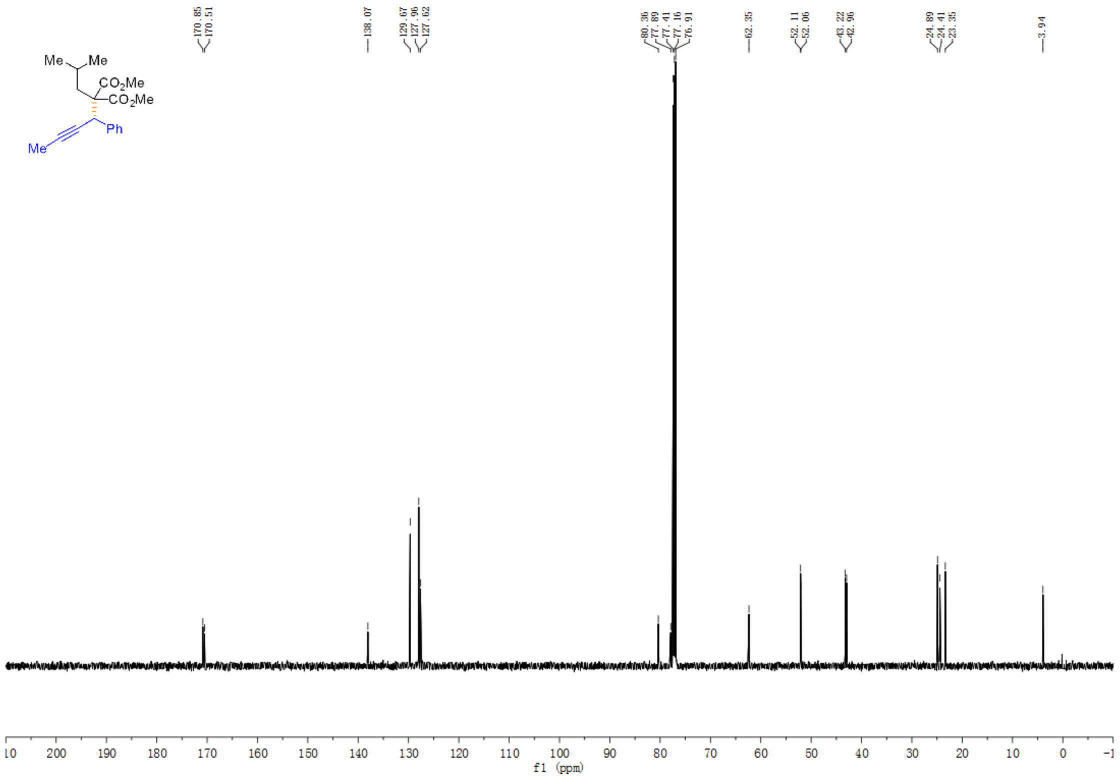

^{1}H NMR spectrum of **3d**

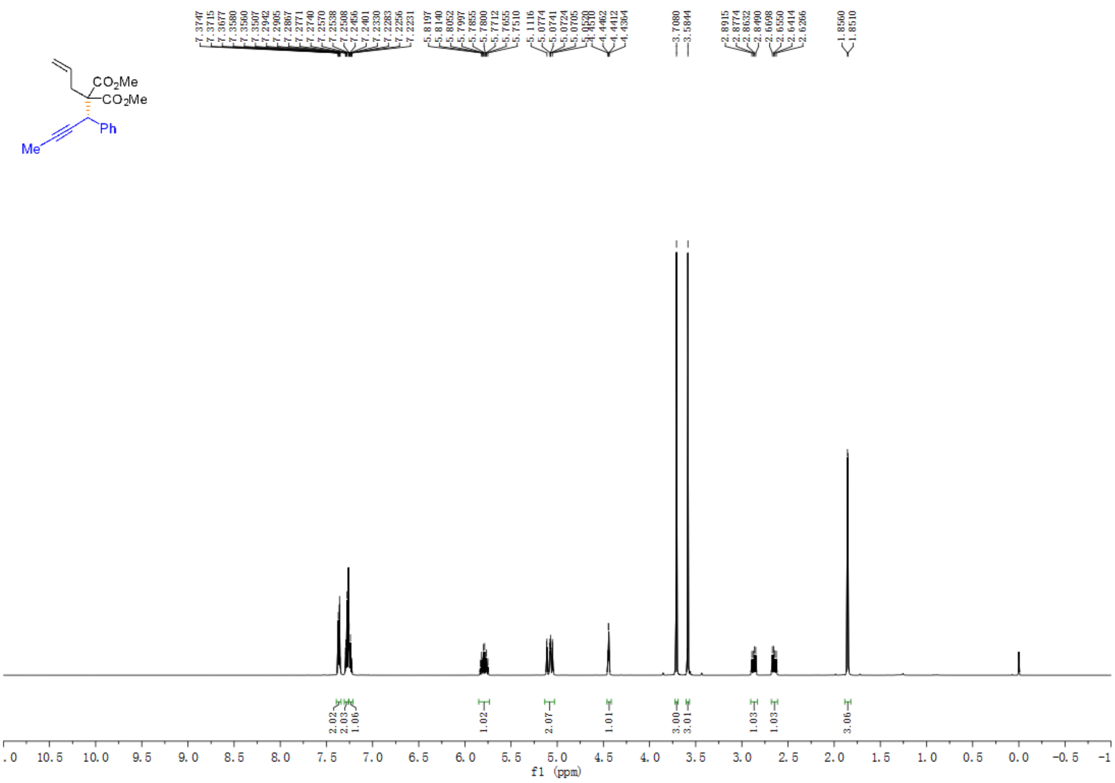

^{13}C NMR spectrum of **3d**

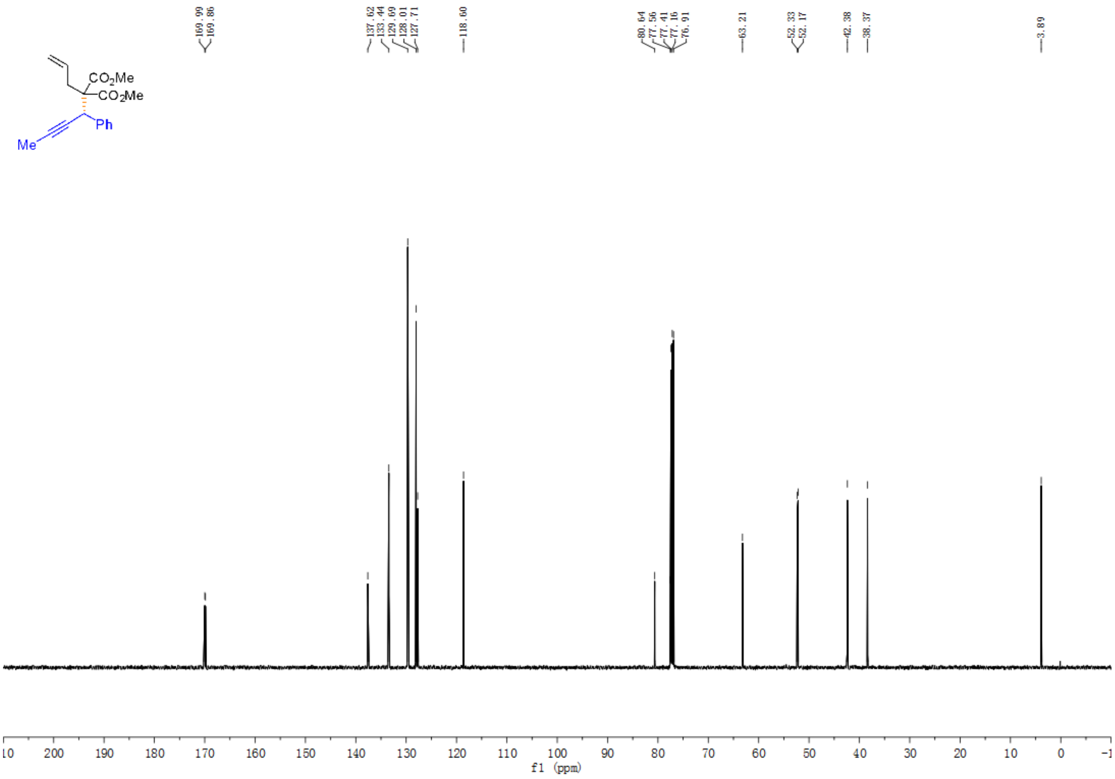

^{1}H NMR spectrum of **3e**

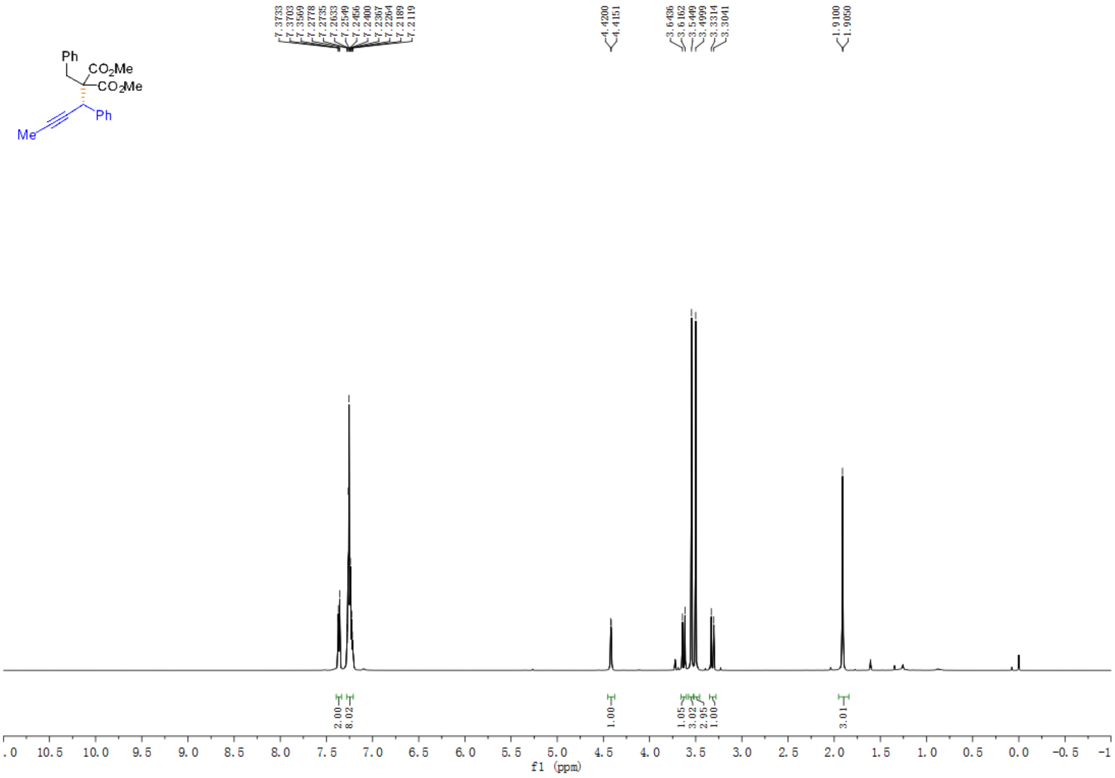

^{13}C NMR spectrum of **3e**

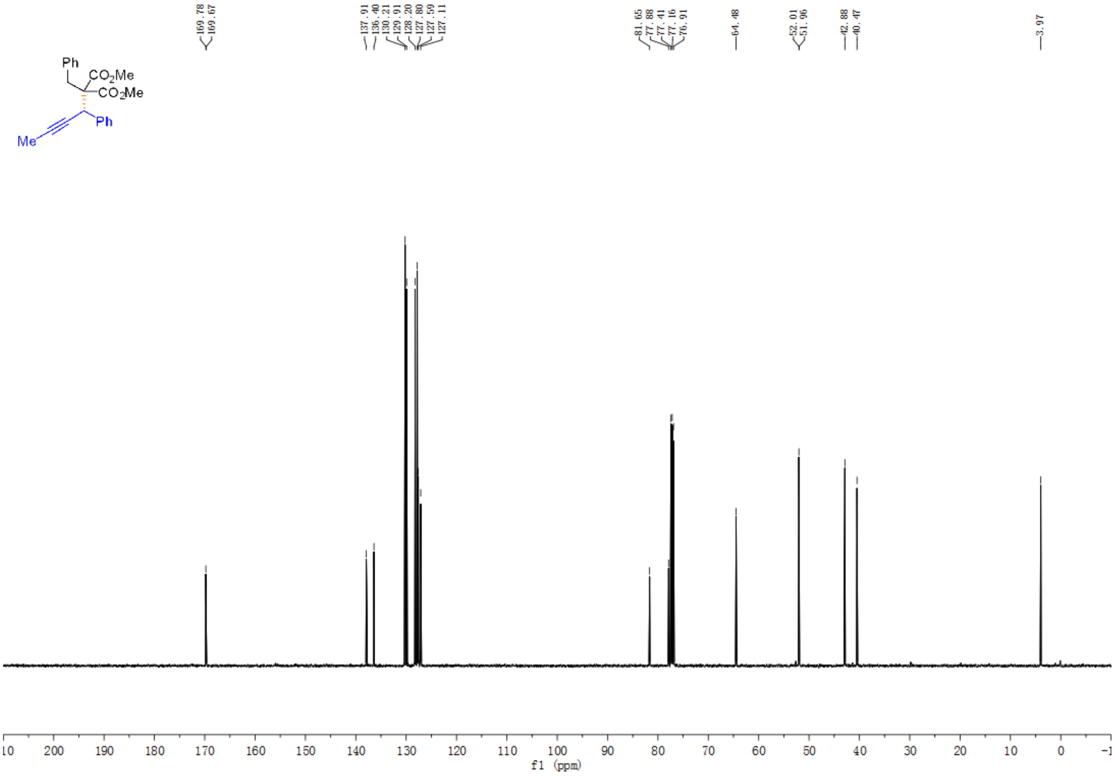

^{1}H NMR spectrum of **3f**

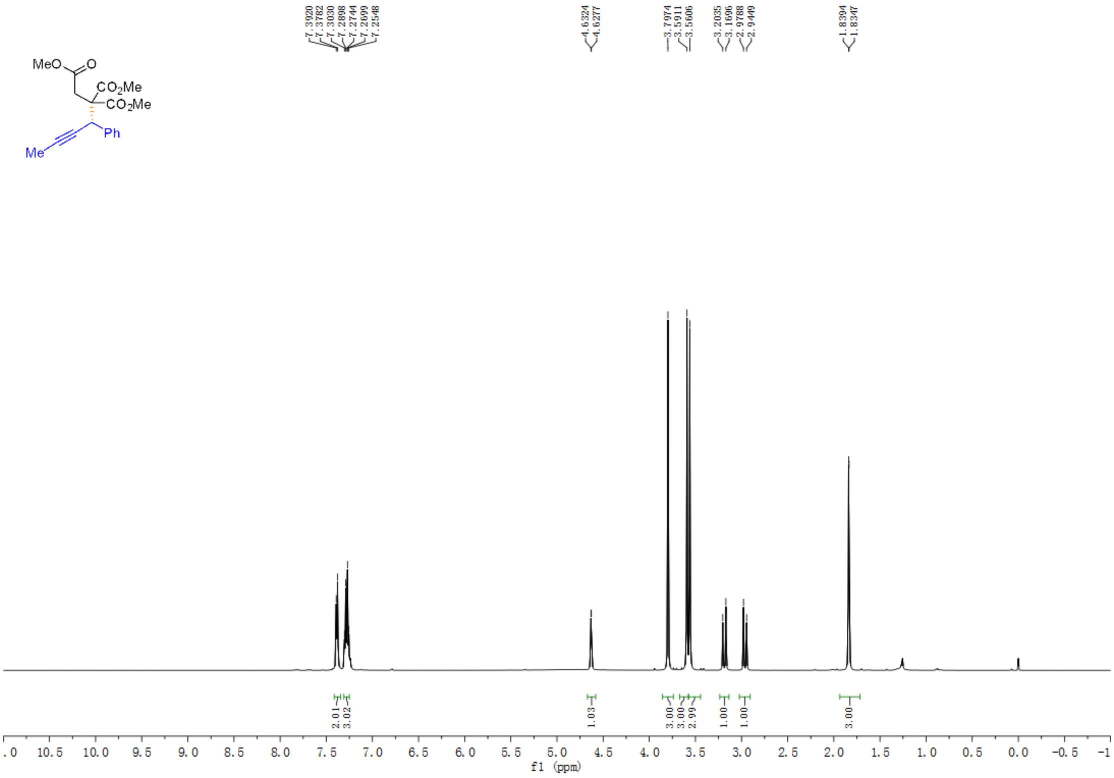

^{13}C NMR spectrum of **3f**

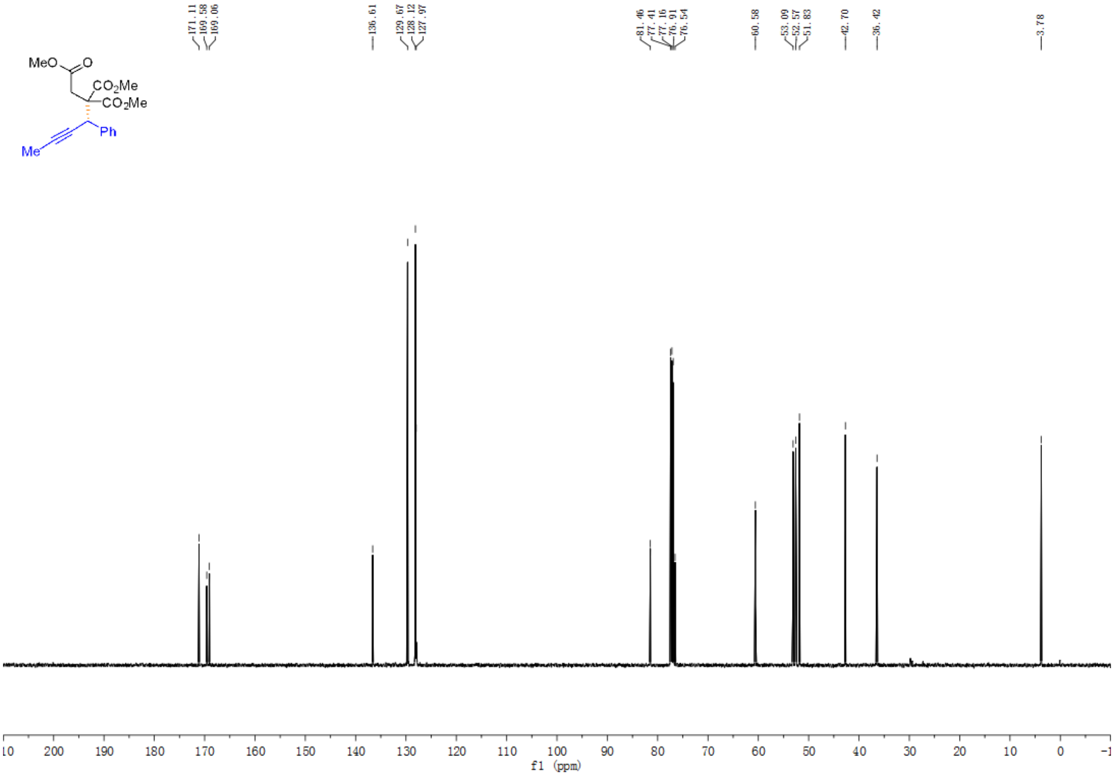

^{1}H NMR spectrum of **3g**

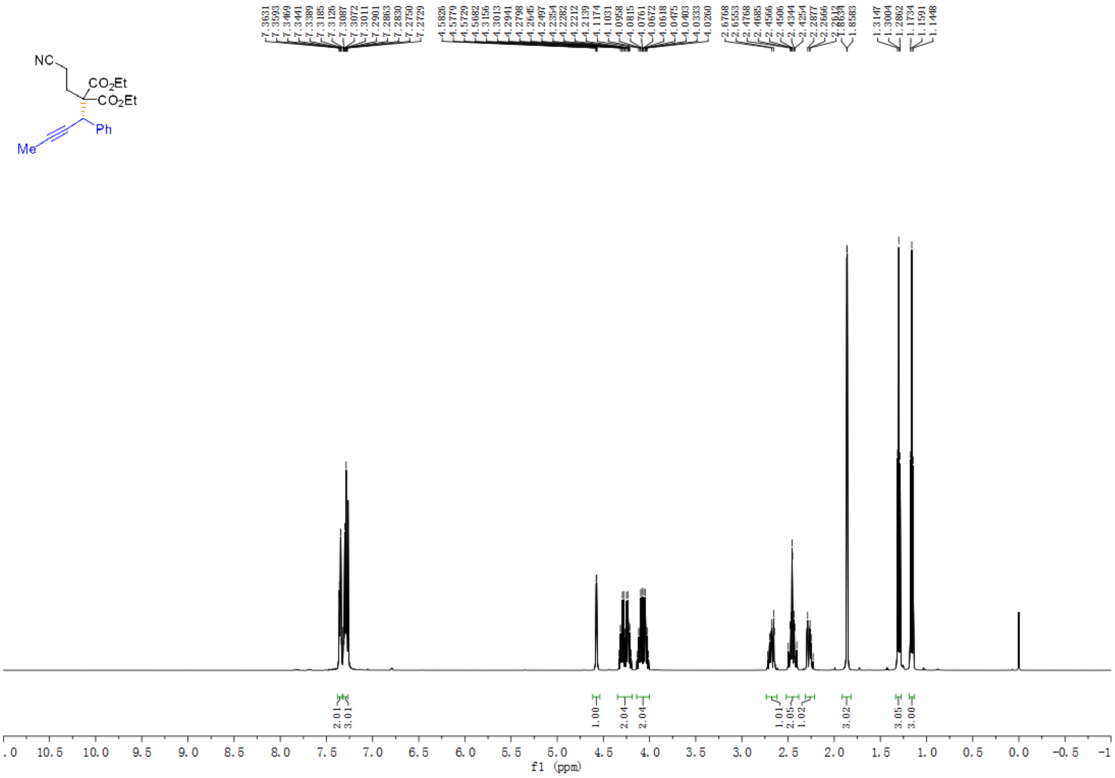

^{13}C NMR spectrum of **3g**

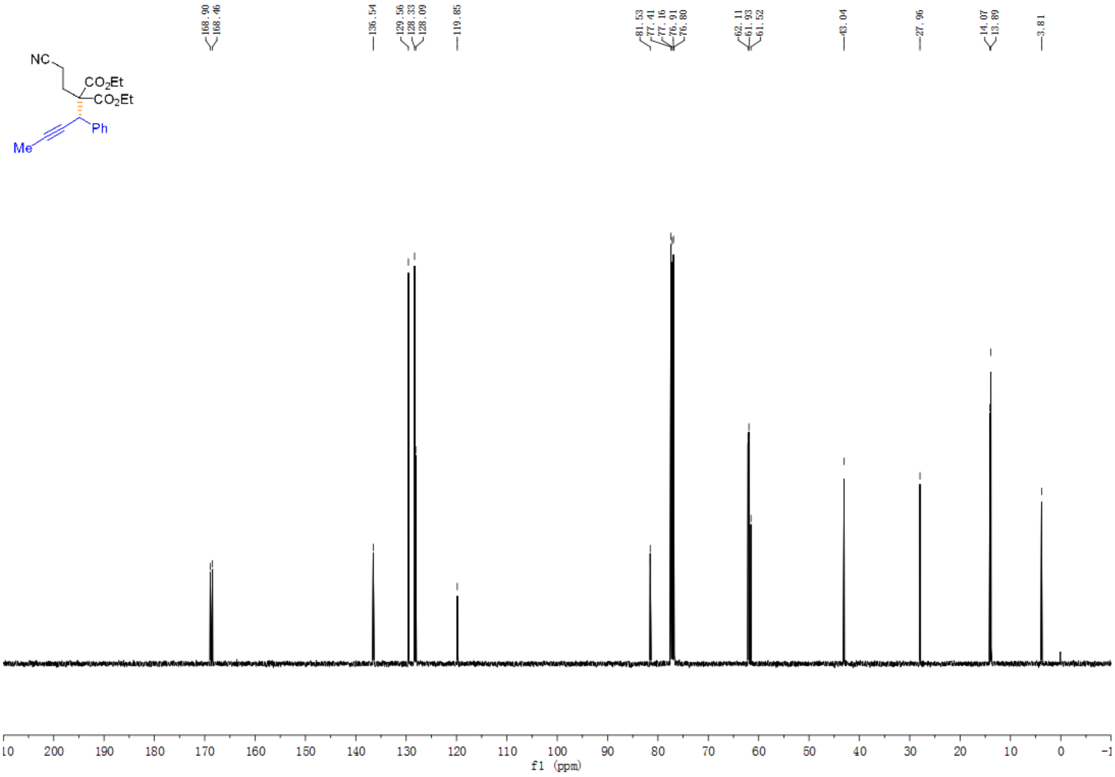

^{1}H NMR spectrum of **3h**

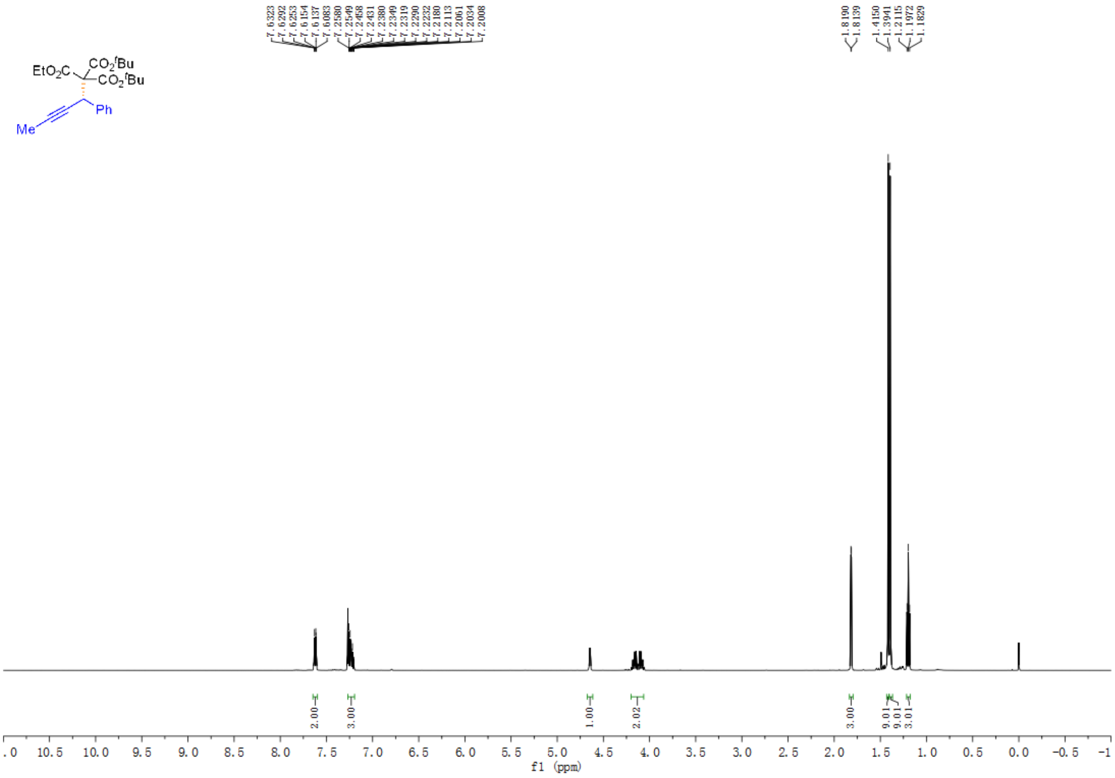

^{13}C NMR spectrum of **3h**

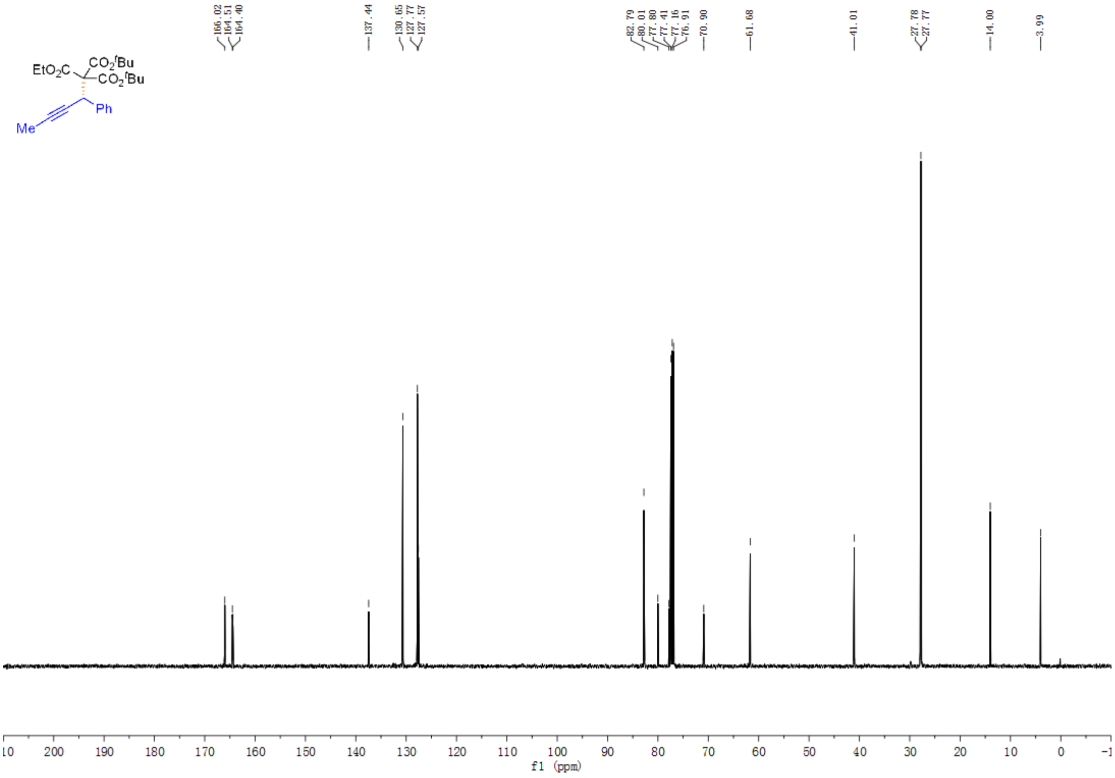

^{1}H NMR spectrum of **3i**

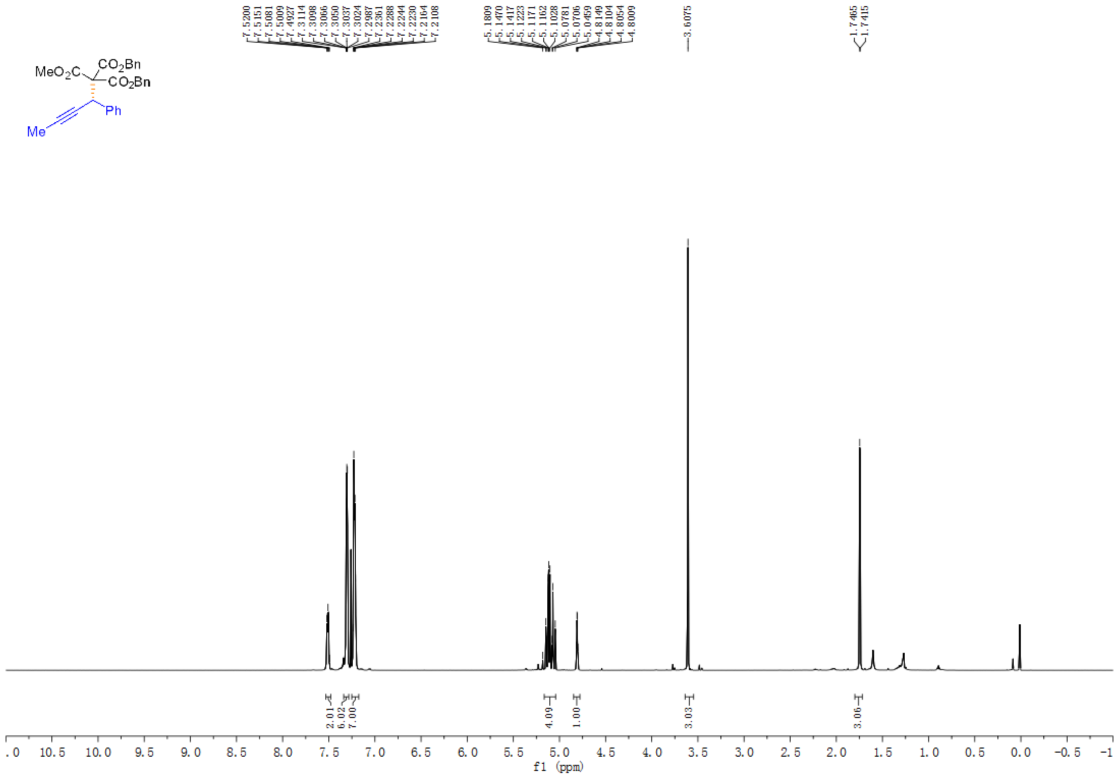

^{13}C NMR spectrum of **3i**

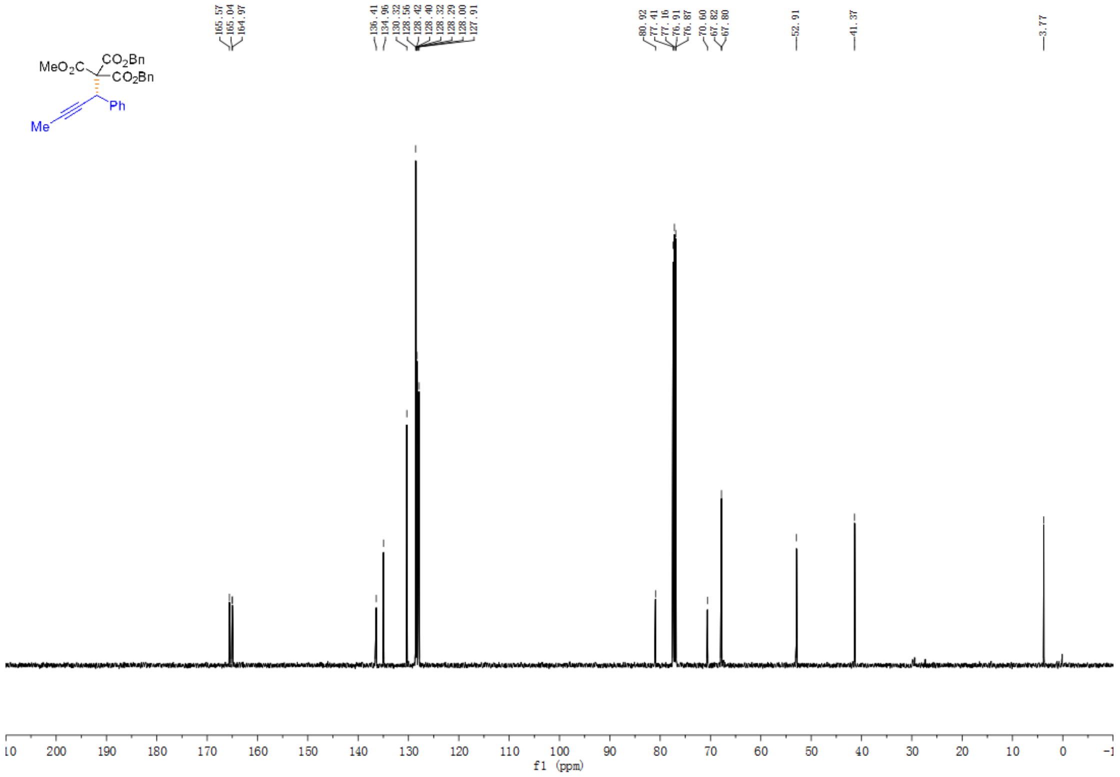

^{1}H NMR spectrum of **3j**

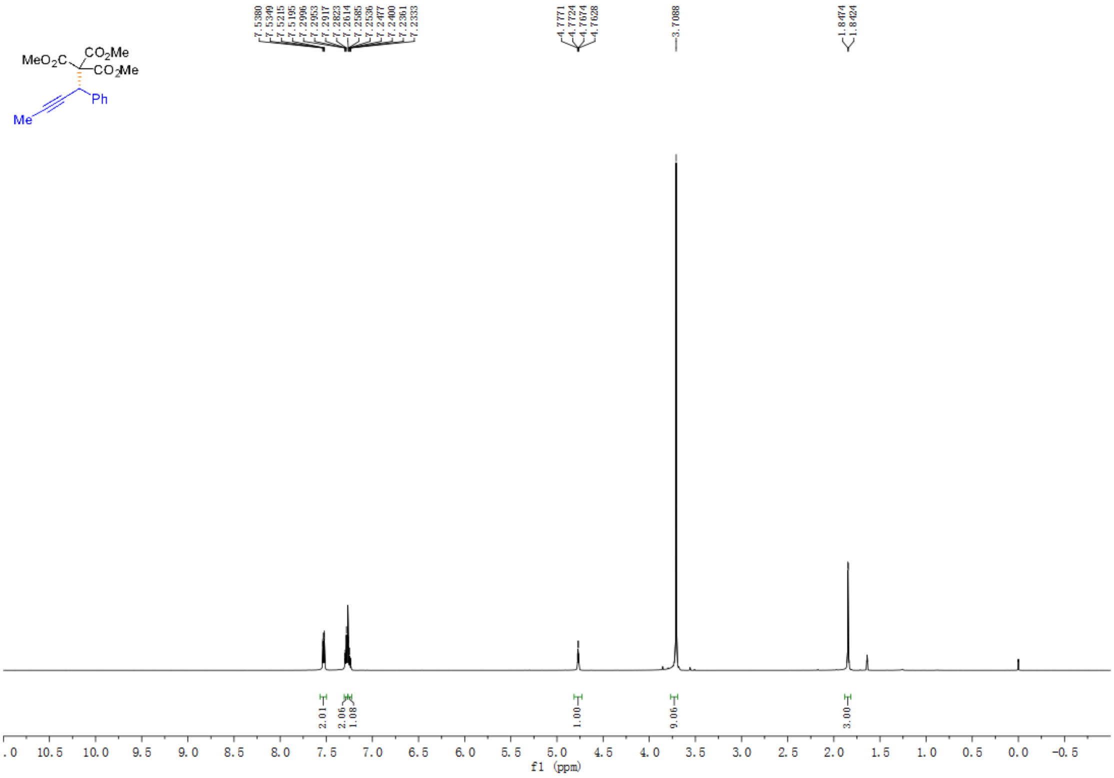

^{13}C NMR spectrum of **3j**

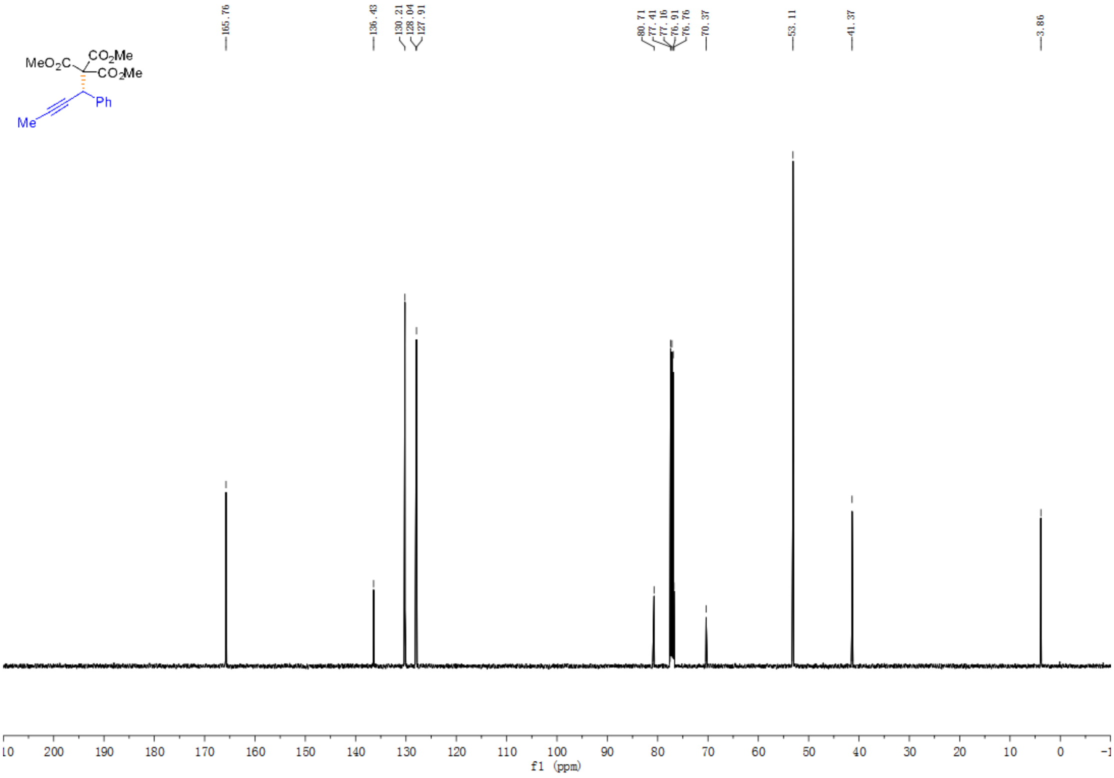

^{1}H NMR spectrum of **3k**

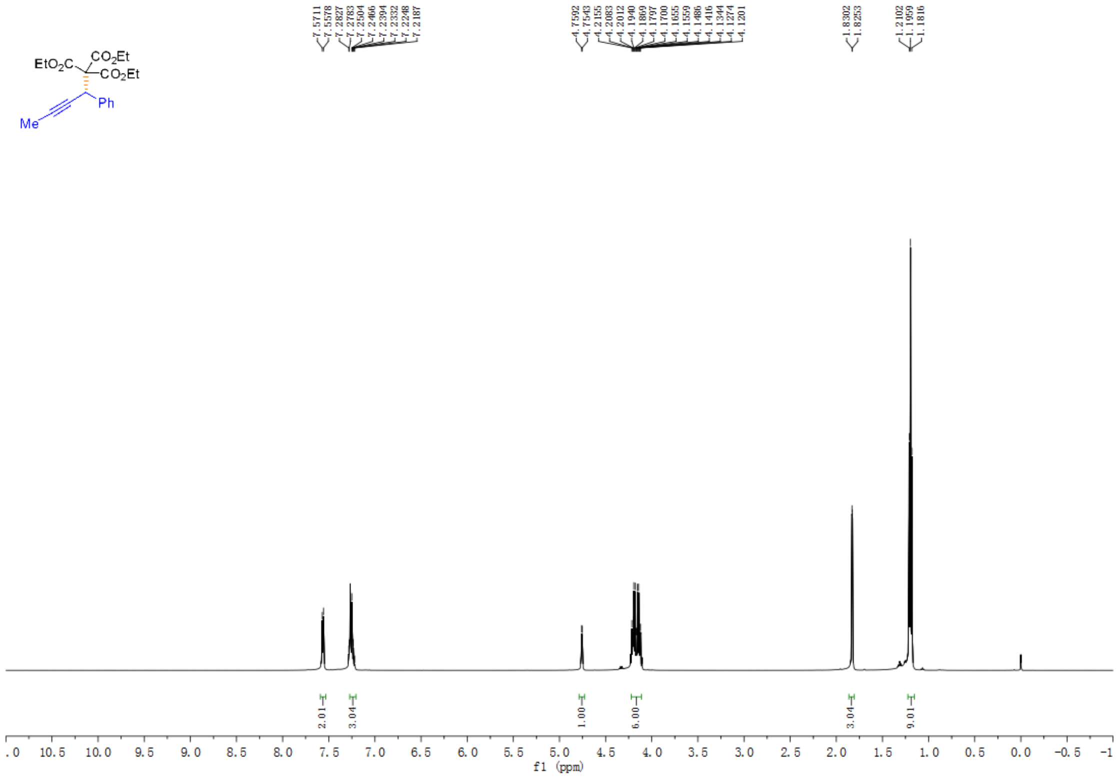

^{13}C NMR spectrum of **3k**

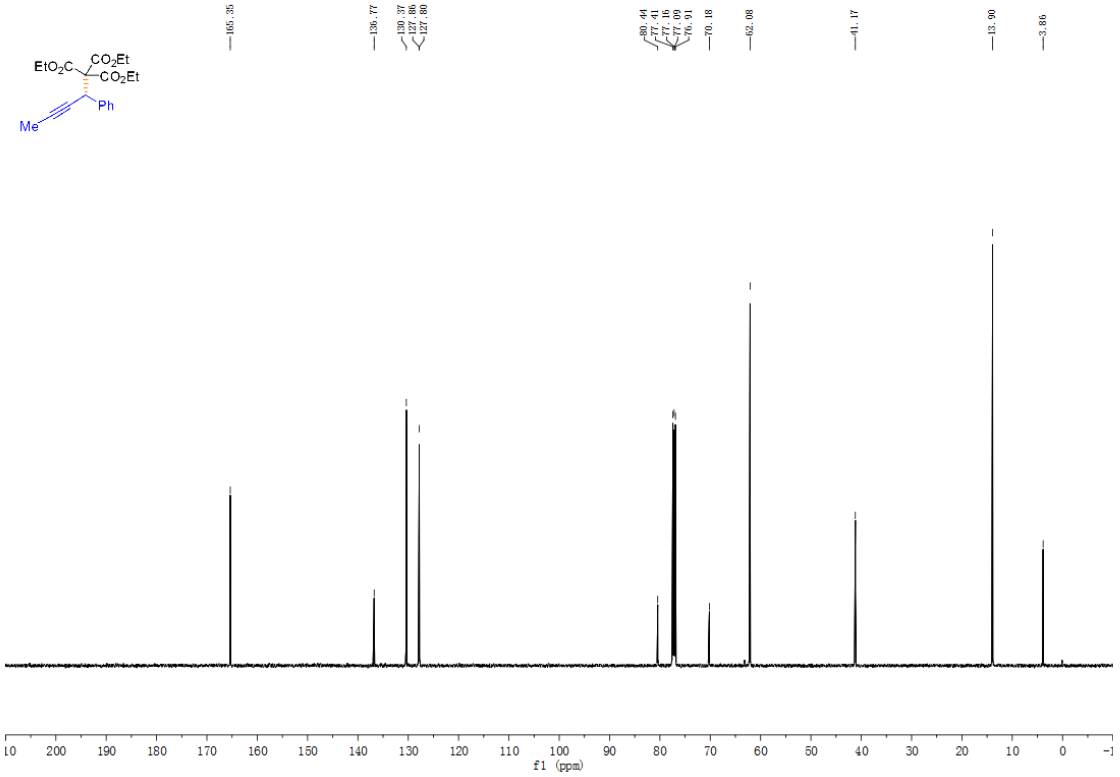

^{1}H NMR spectrum of **3l**

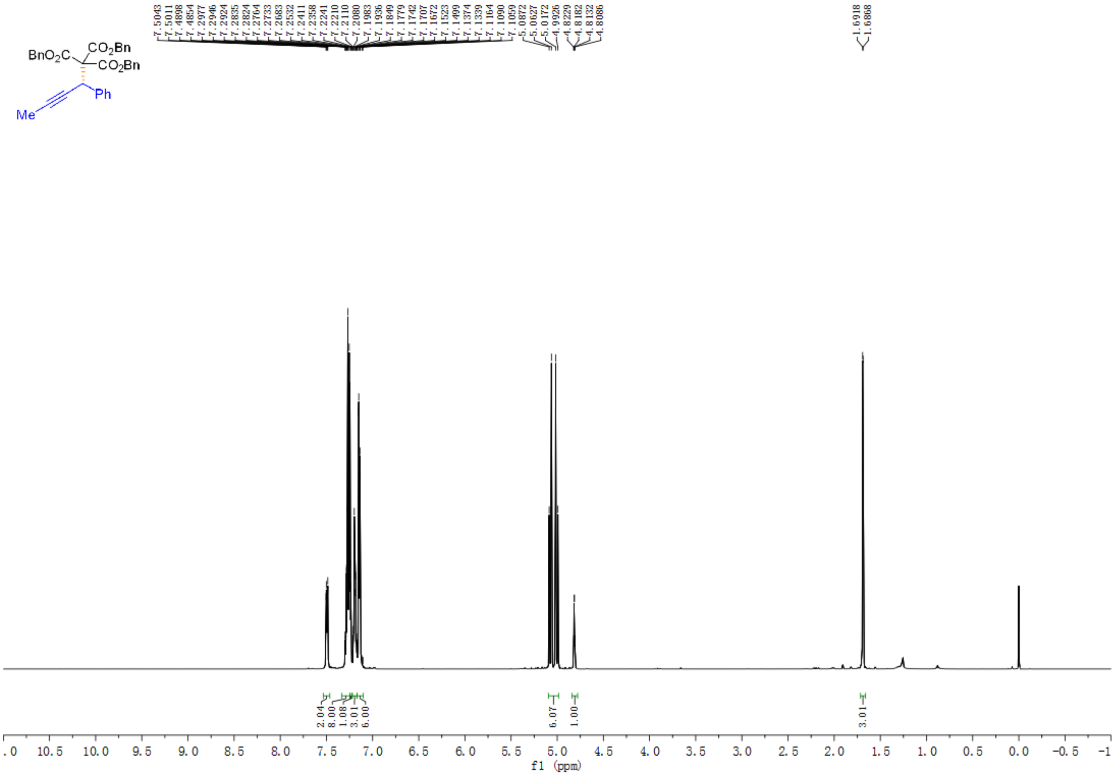

^{13}C NMR spectrum of **3l**

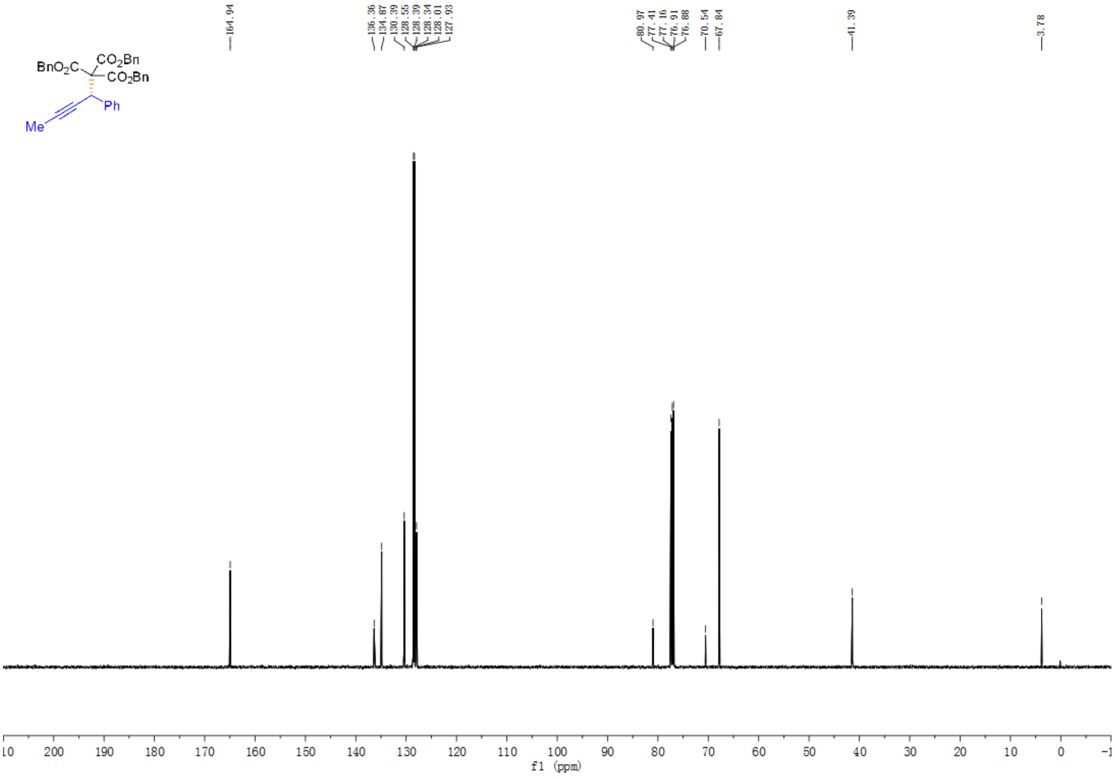

^{1}H NMR spectrum of **3m**

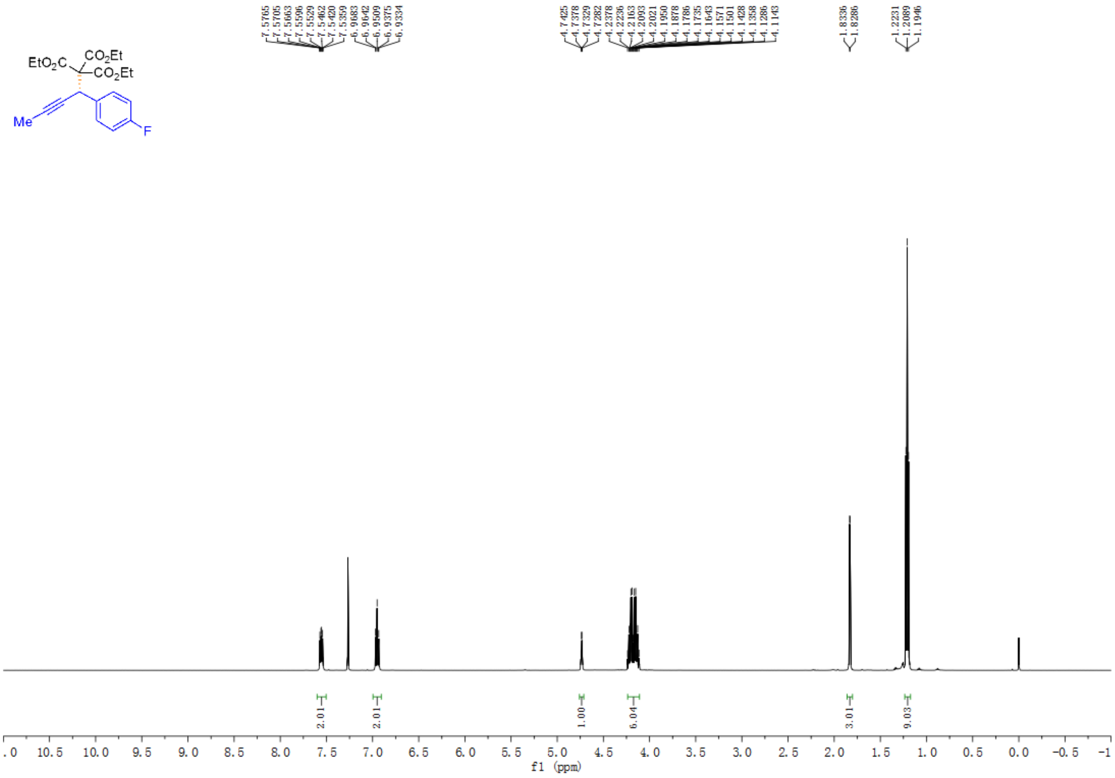

^{13}C NMR spectrum of **3m**

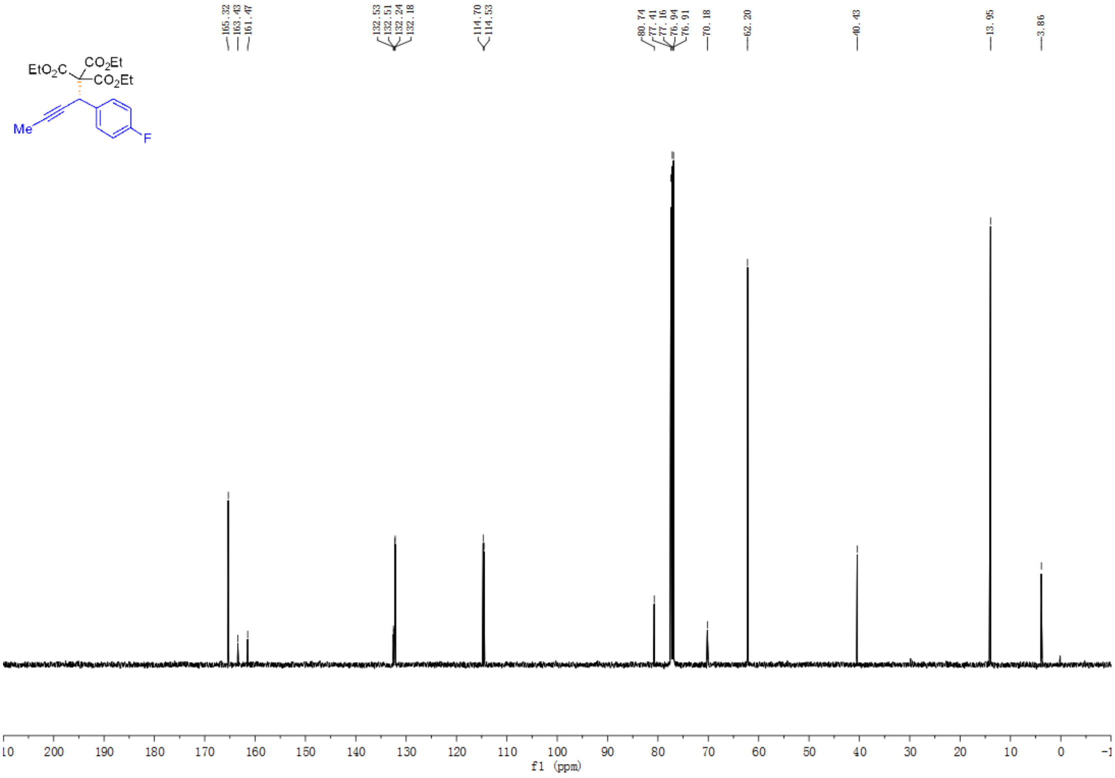

^{19}F NMR spectrum of **3m**

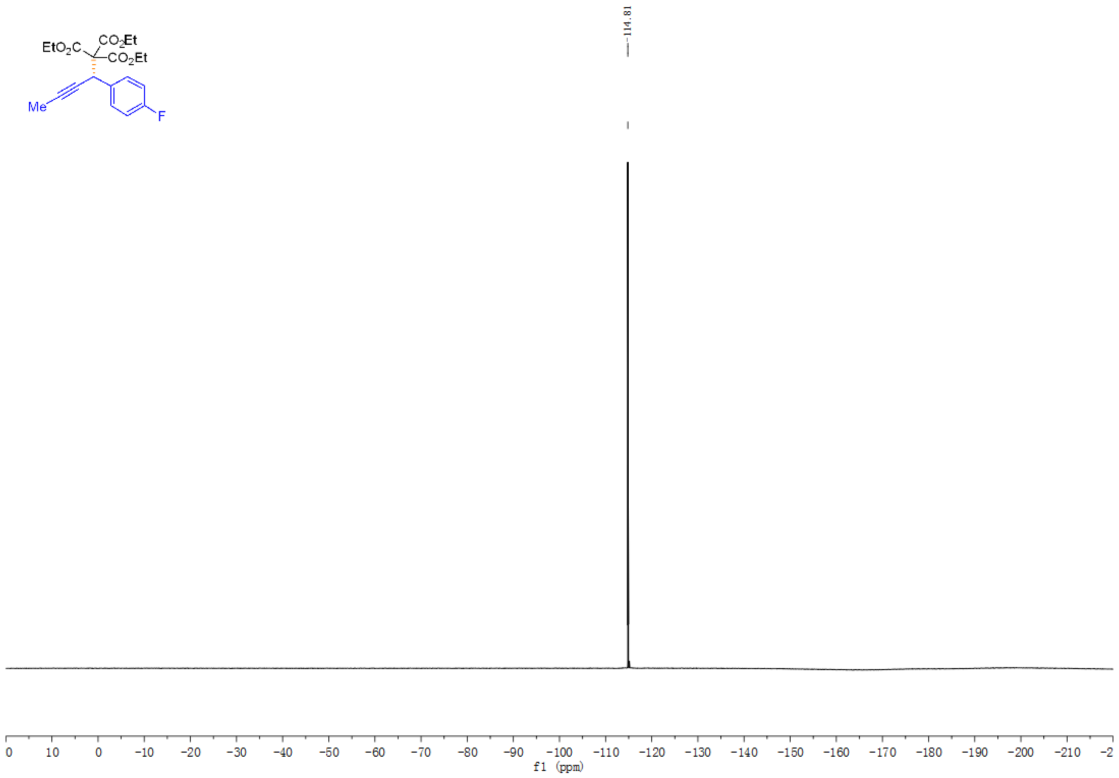

^{1}H NMR spectrum of **3n**

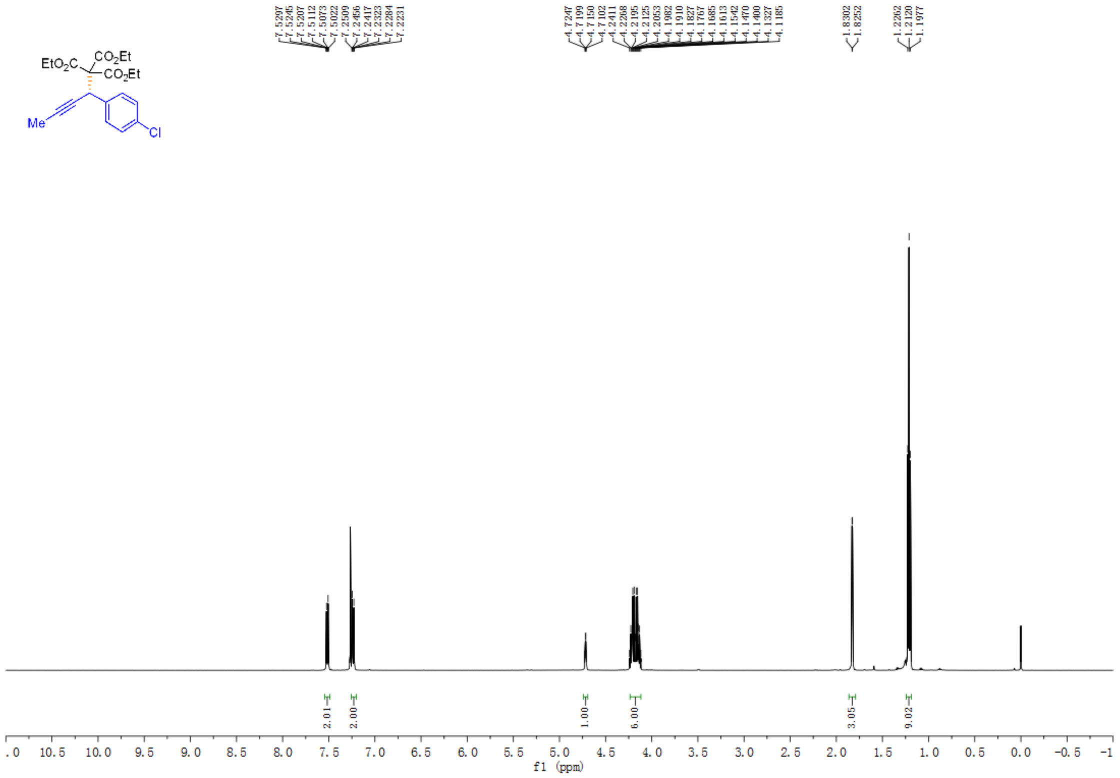

^{13}C NMR spectrum of **3n**

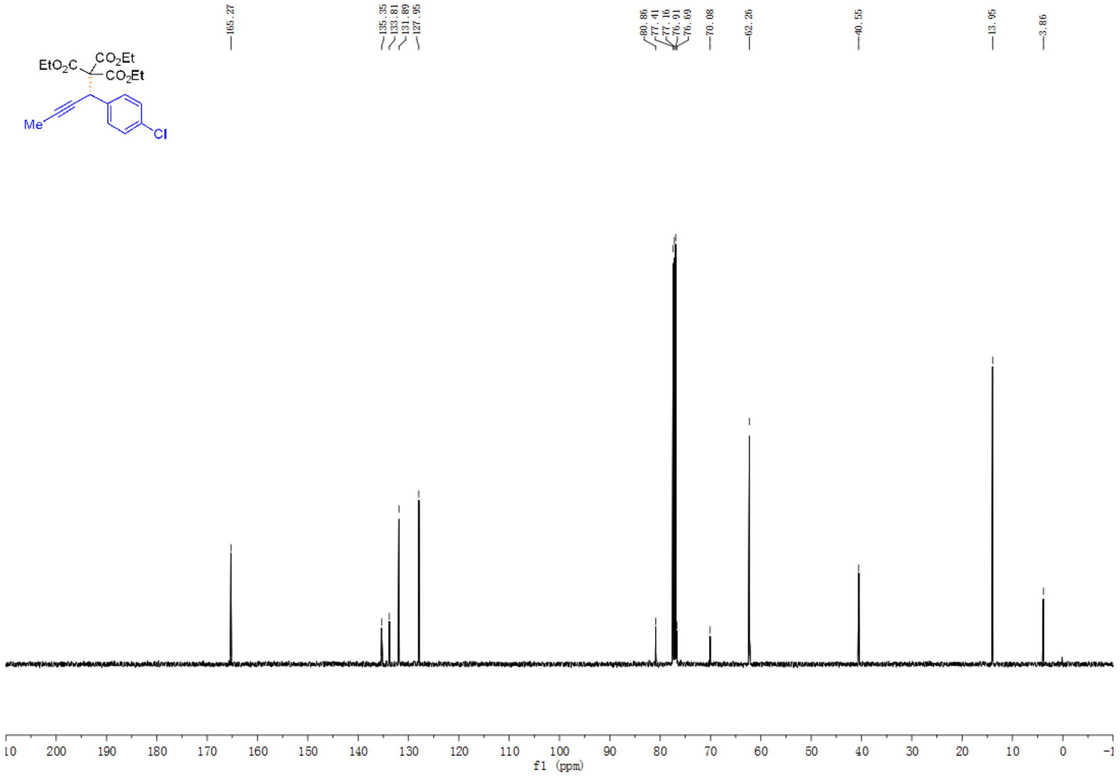

^{1}H NMR spectrum of **3o**

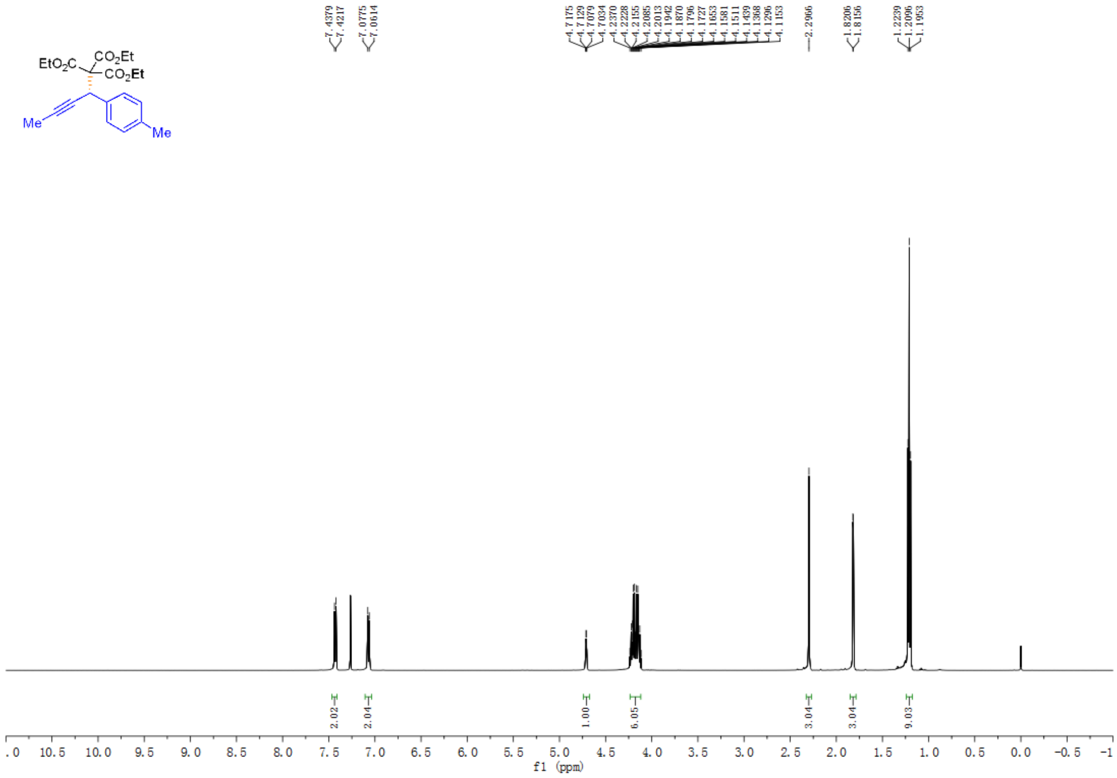

^{13}C NMR spectrum of **3o**

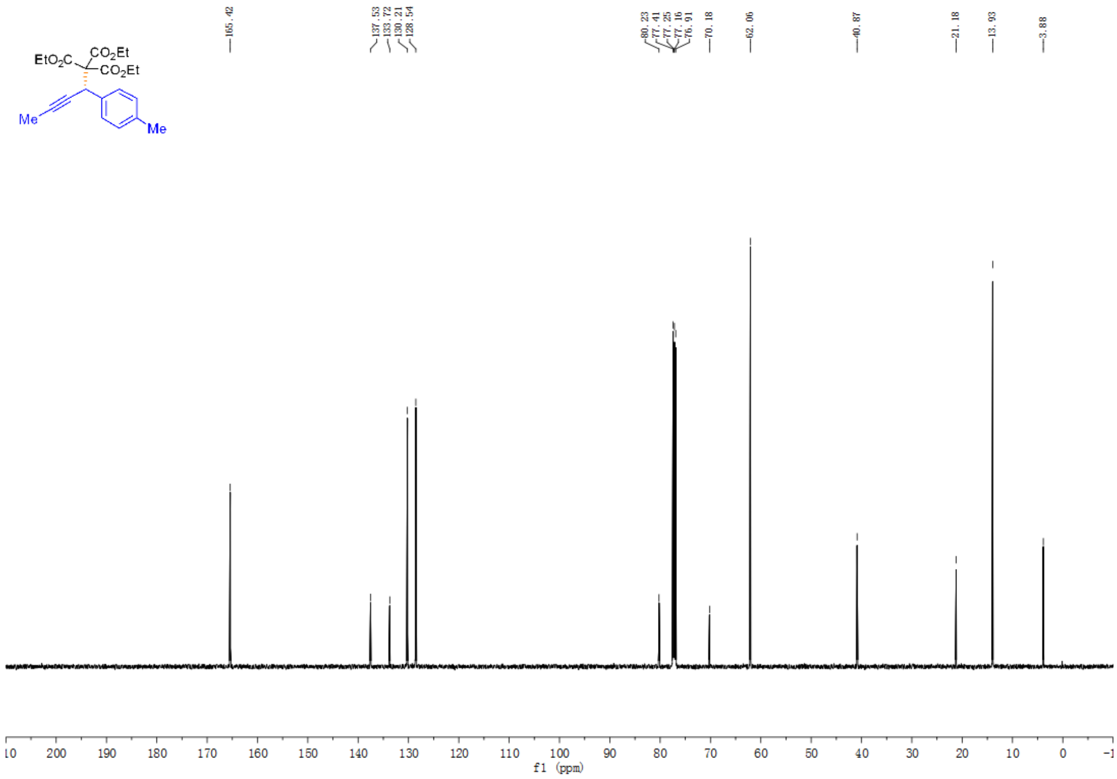

^{1}H NMR spectrum of **3p**

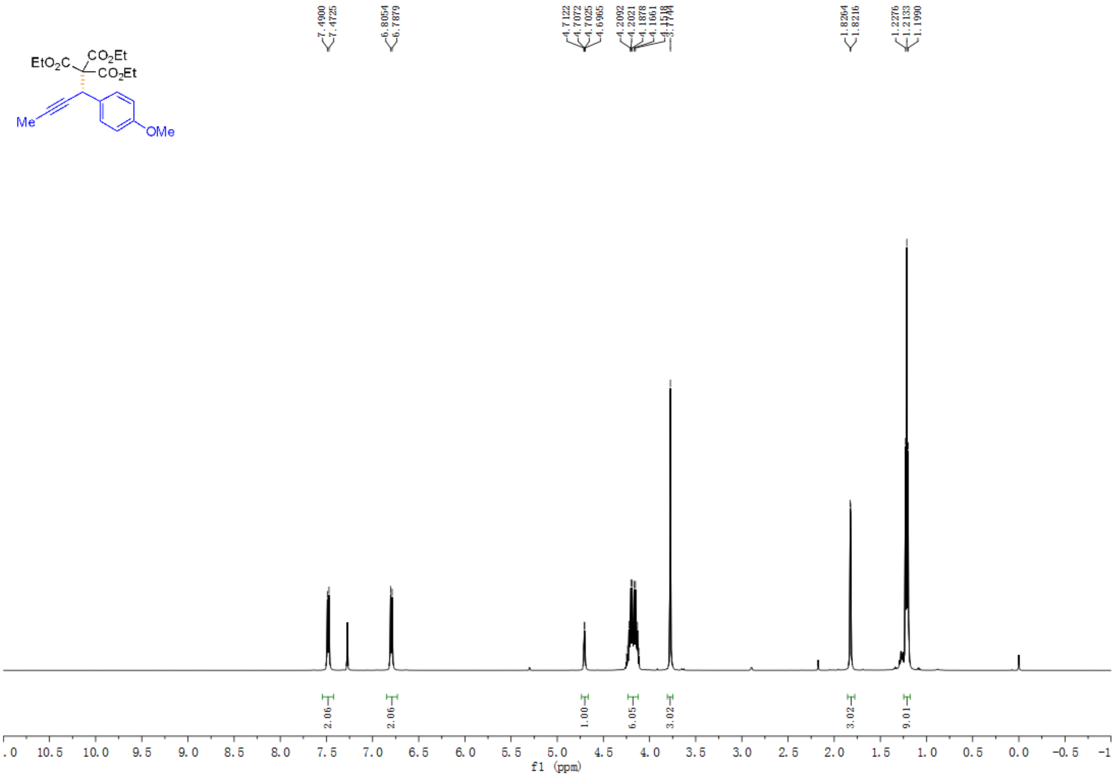

^{13}C NMR spectrum of **3p**

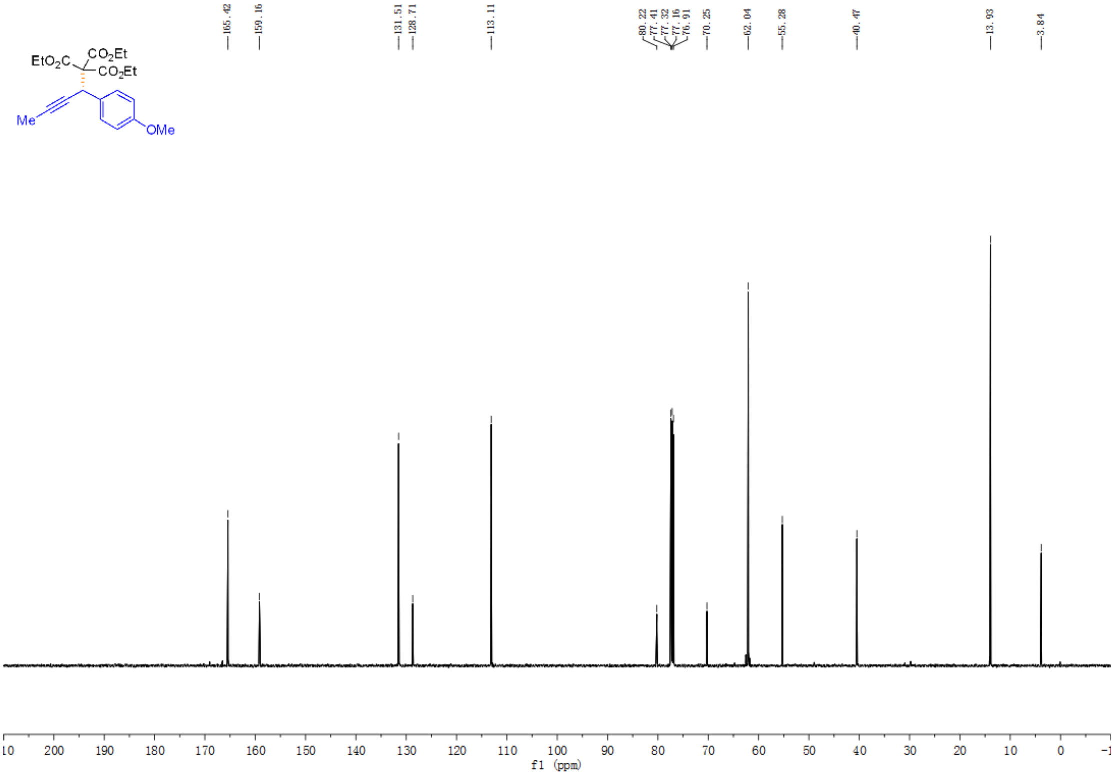

^{1}H NMR spectrum of **3q**

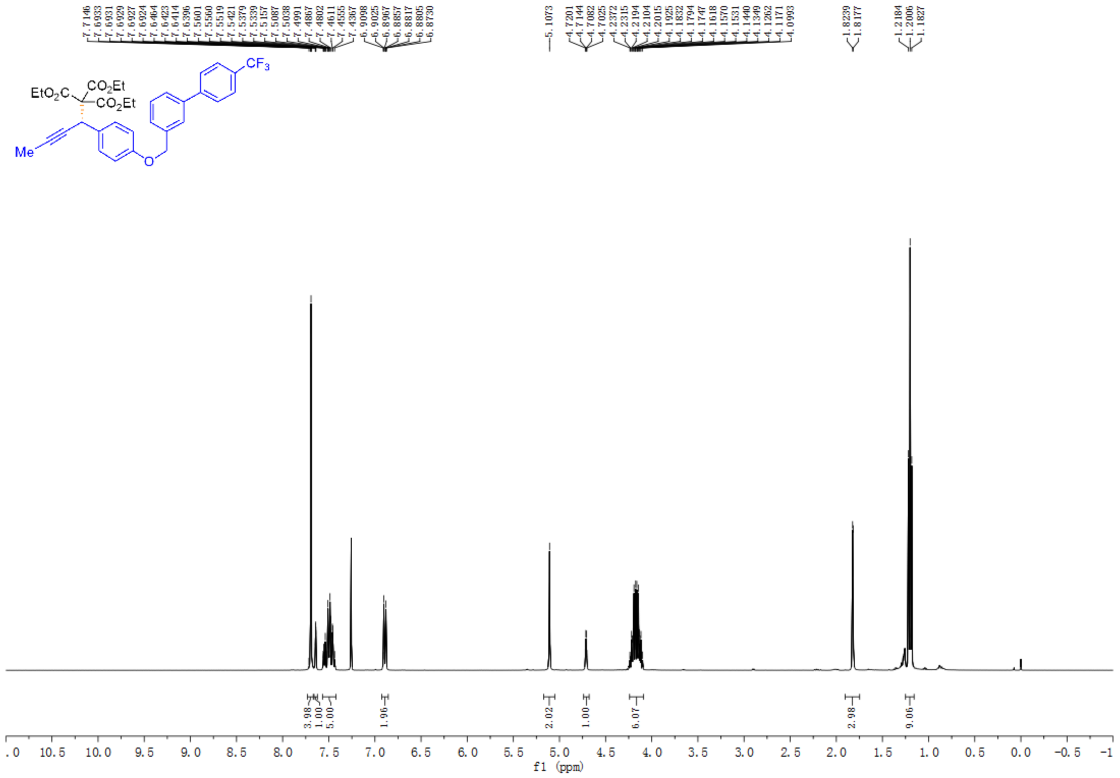

^{13}C NMR spectrum of **3q**

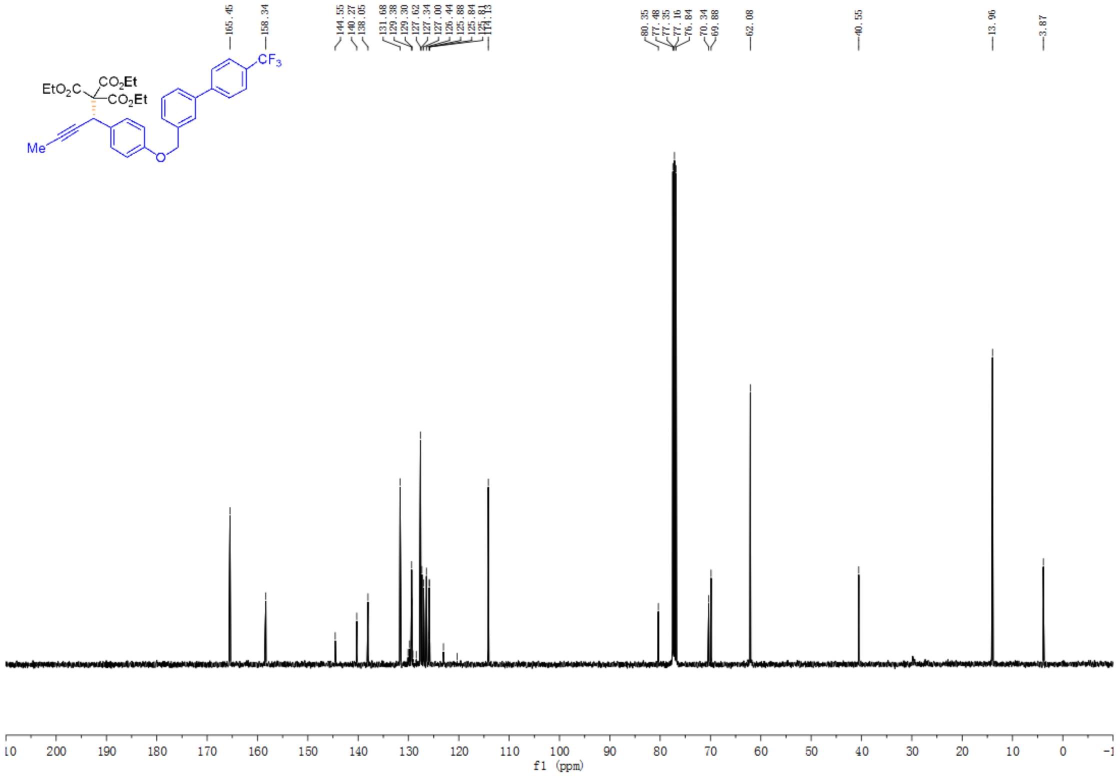

^{19}F NMR spectrum of **3q**

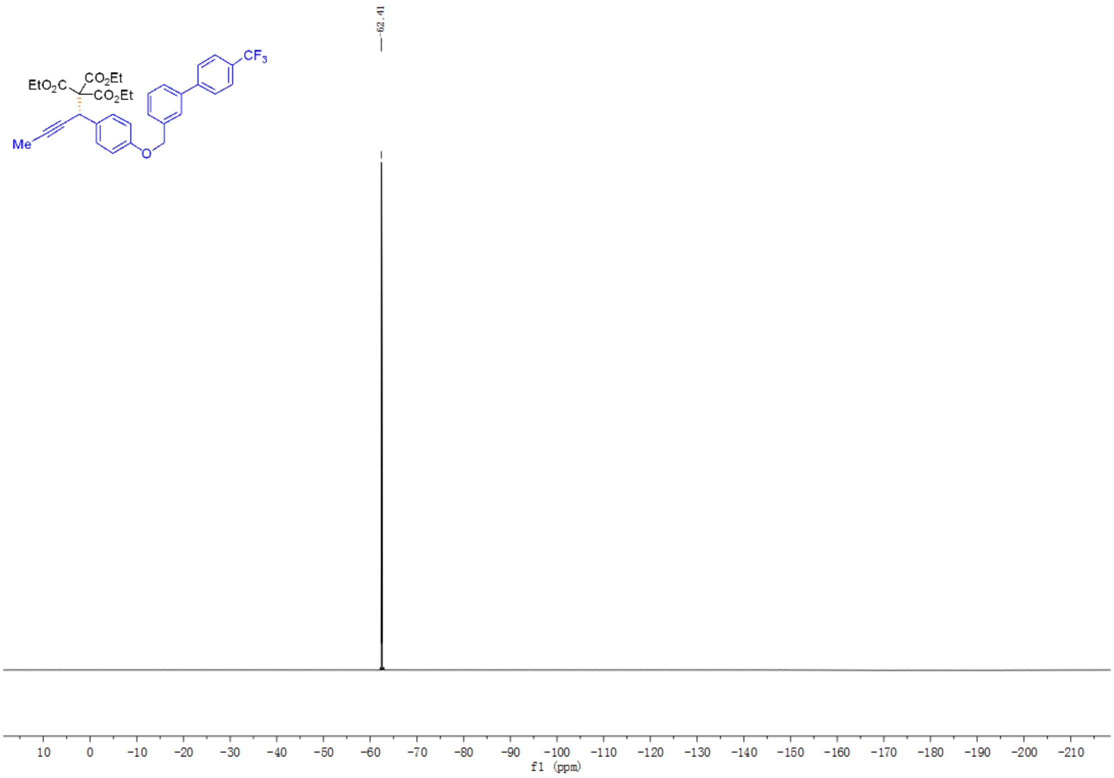

^{1}H NMR spectrum of **3r**

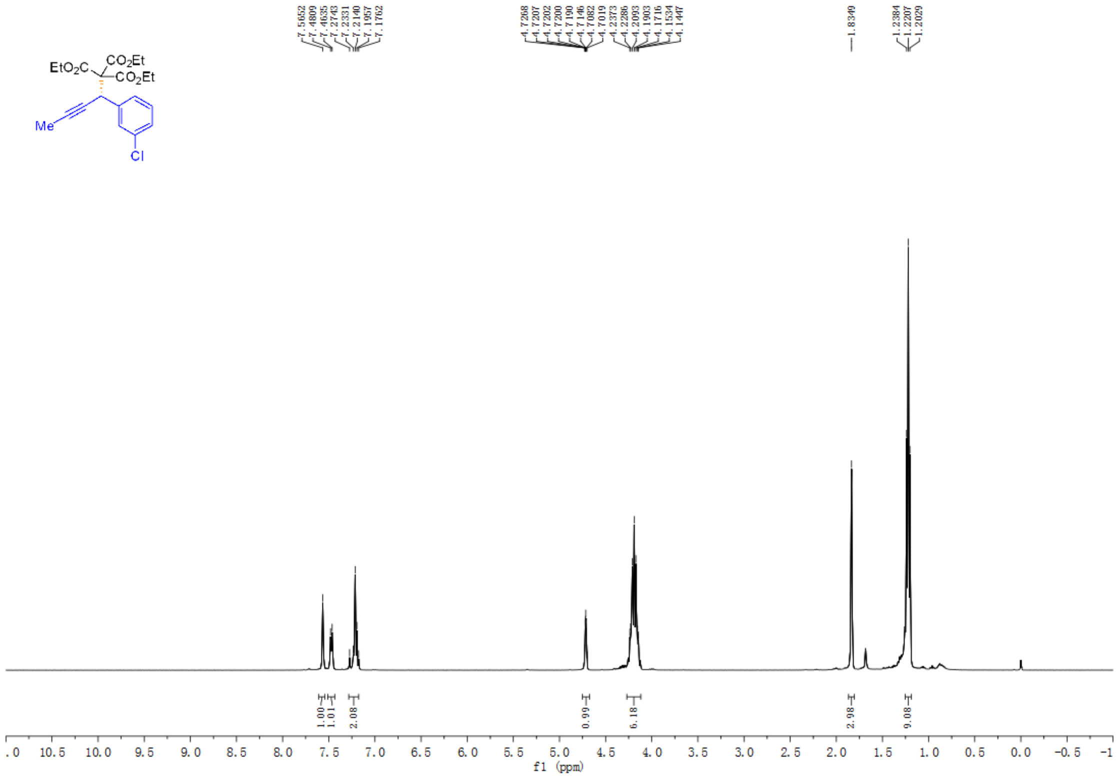

^{13}C NMR spectrum of **3r**

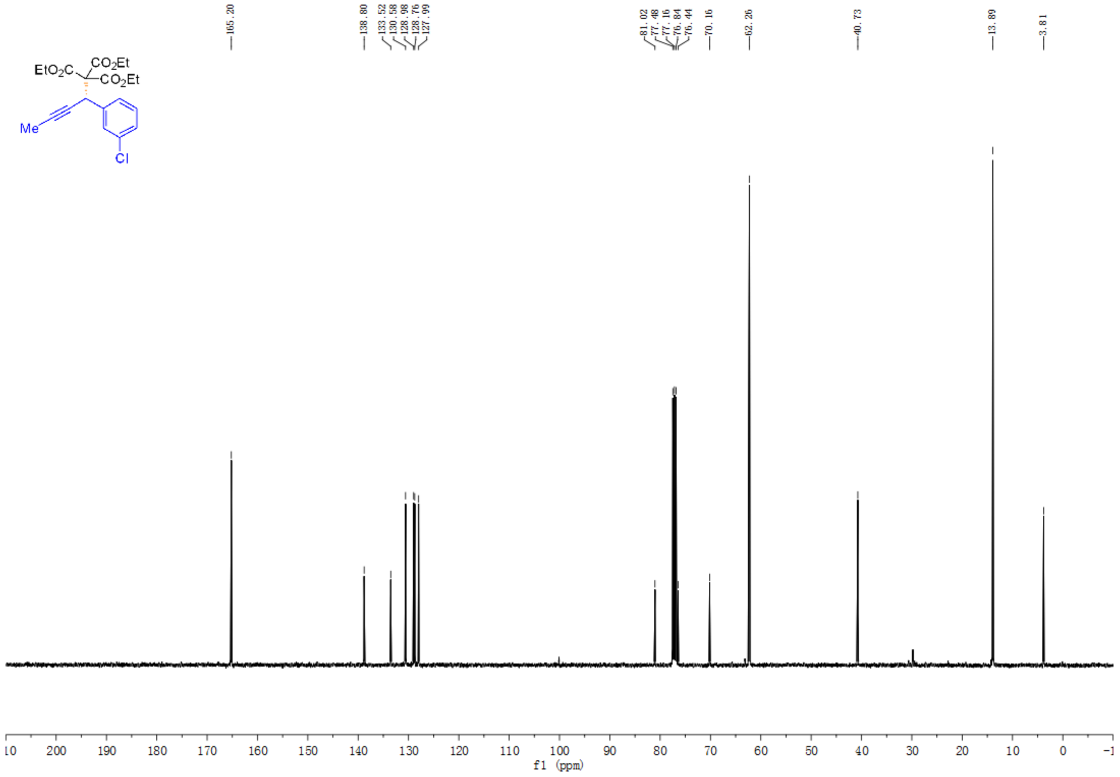

^{1}H NMR spectrum of **3s**

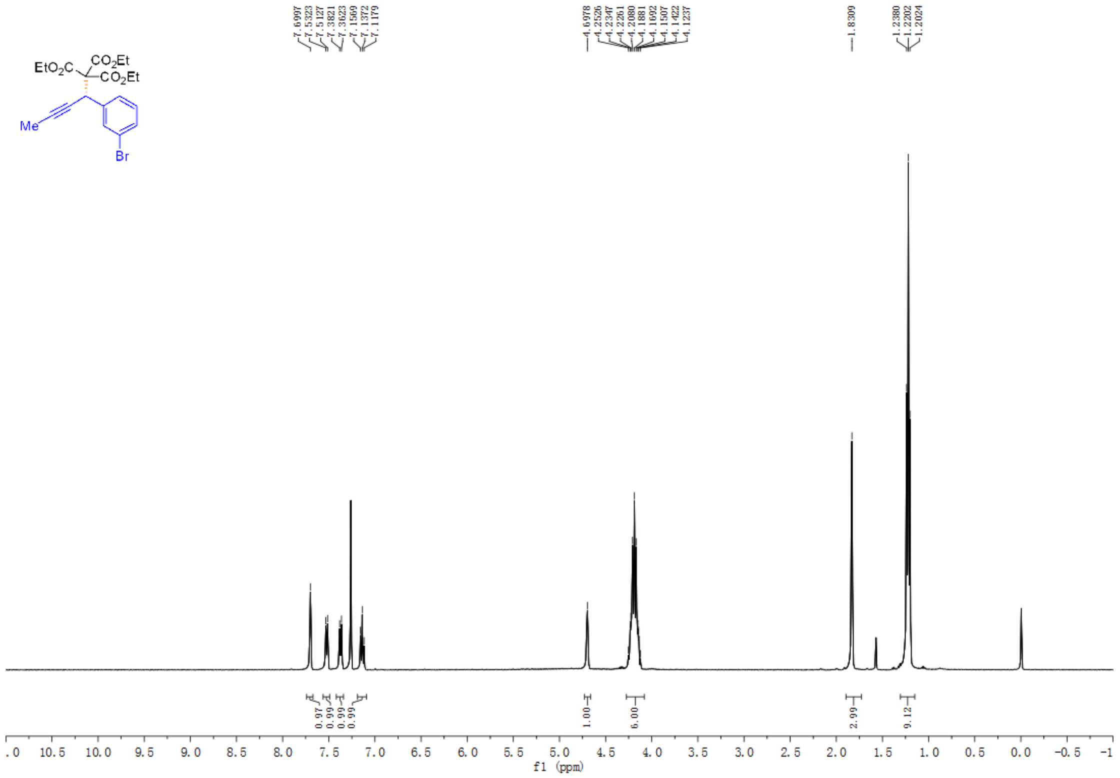

^{13}C NMR spectrum of **3s**

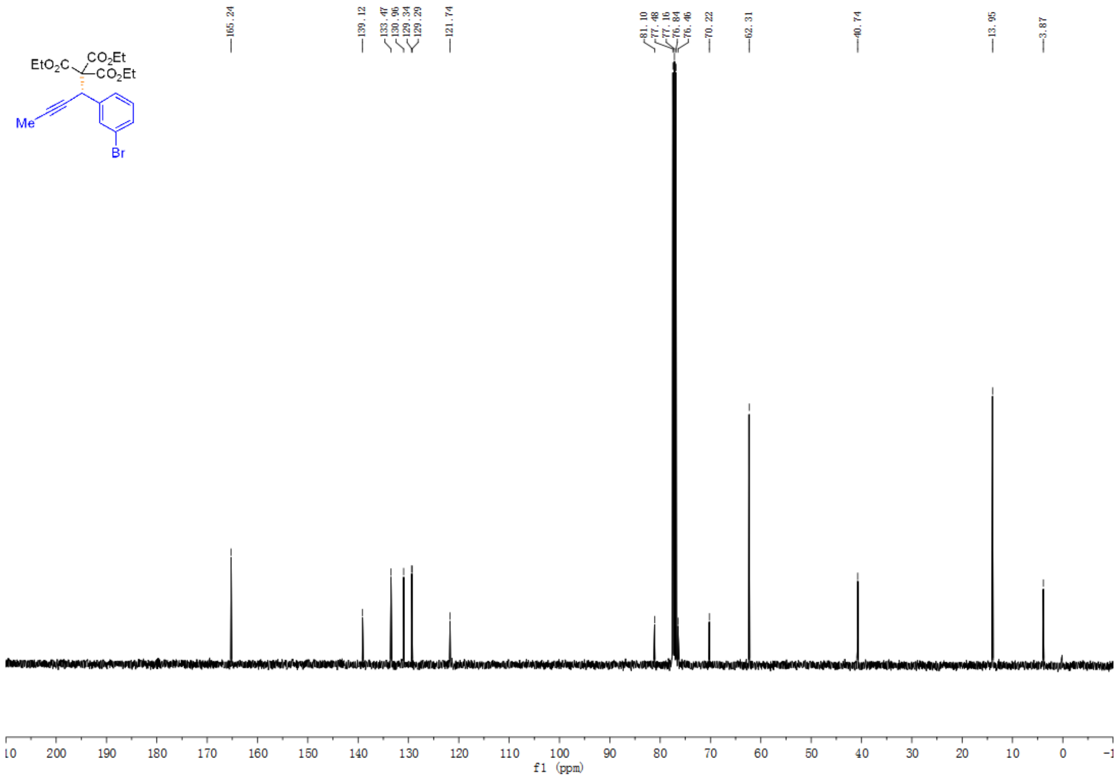

^{1}H NMR spectrum of **3t**

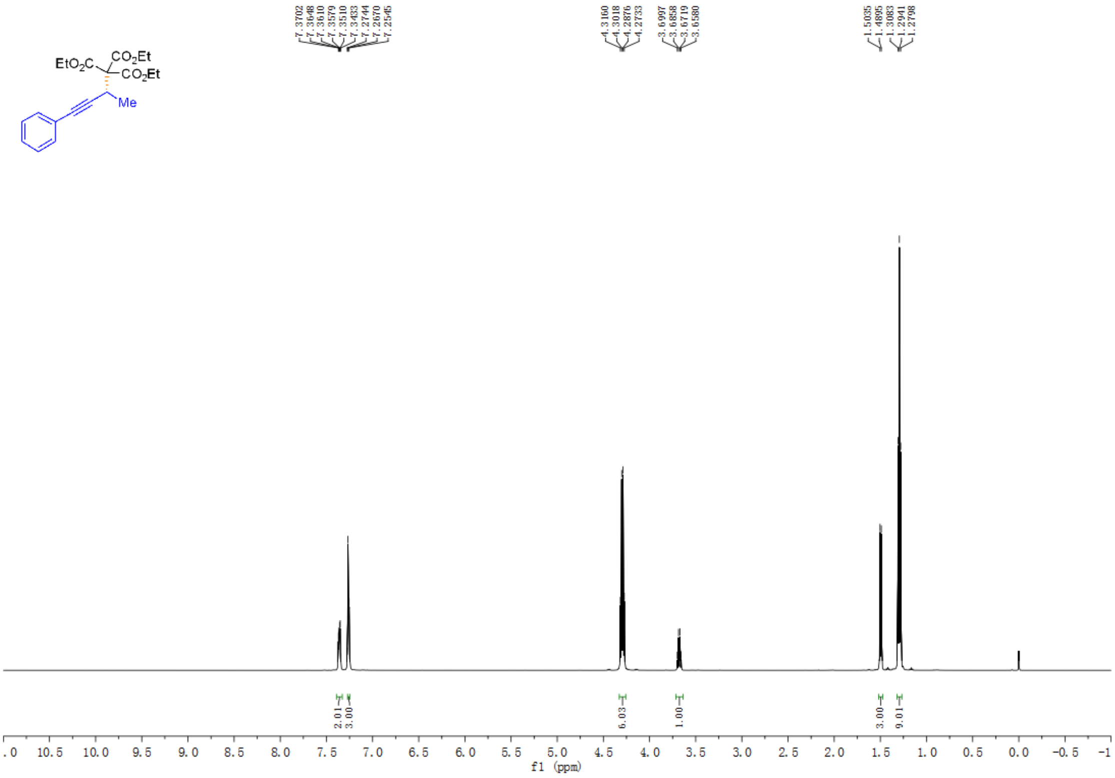

^{13}C NMR spectrum of **3t**

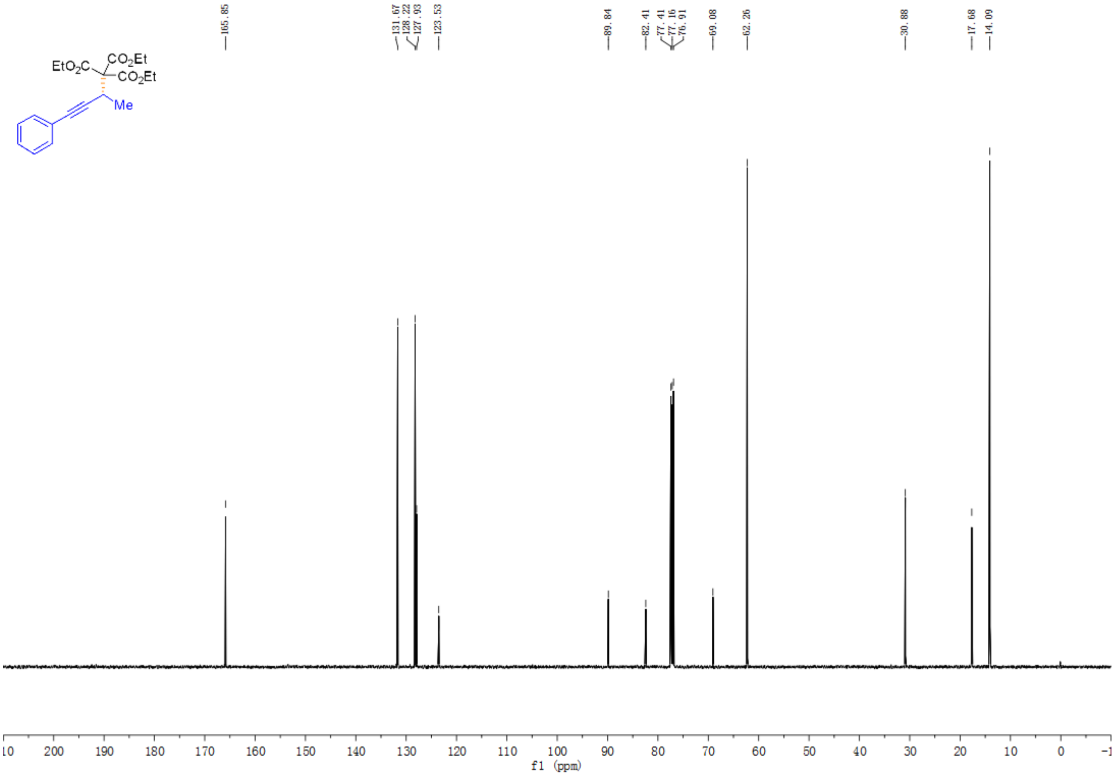

^{1}H NMR spectrum of **3u**

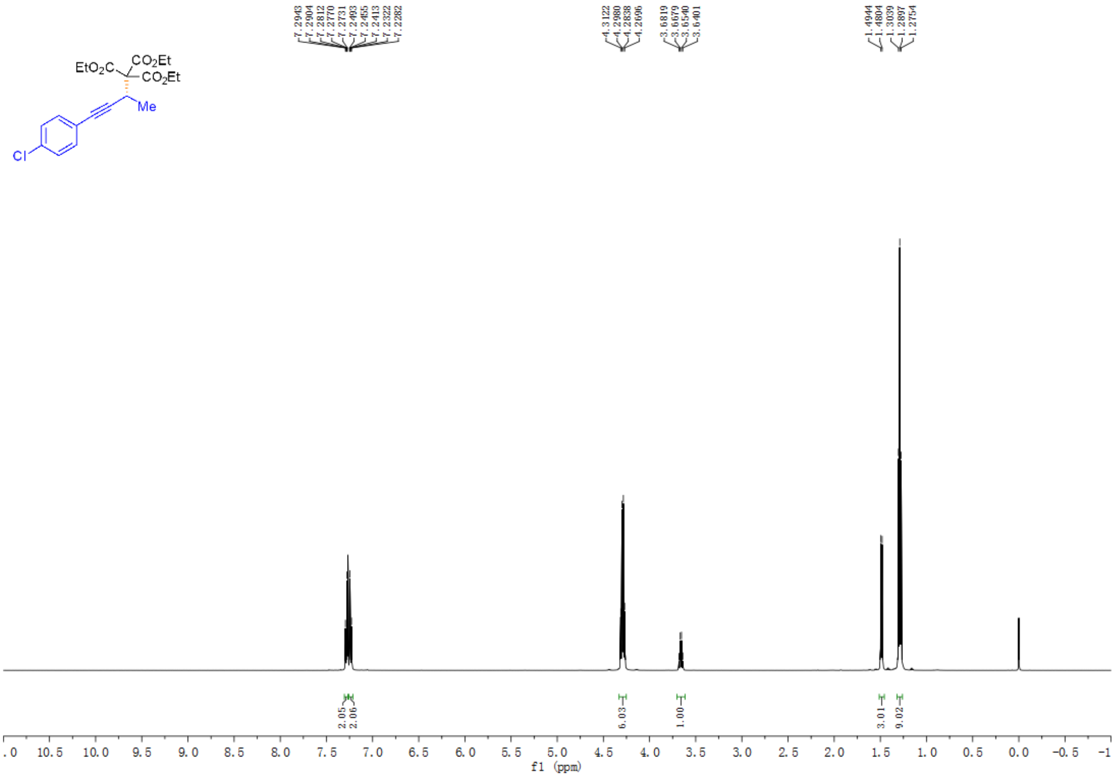

^{13}C NMR spectrum of **3u**

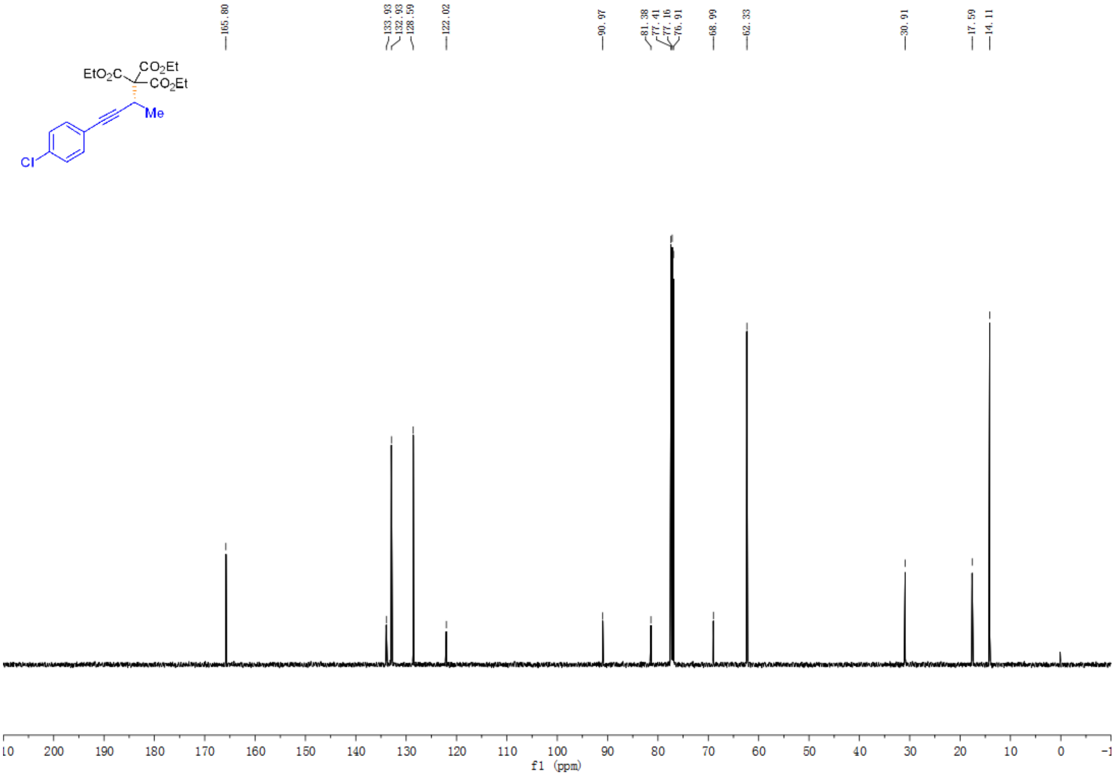

^{1}H NMR spectrum of **3v**

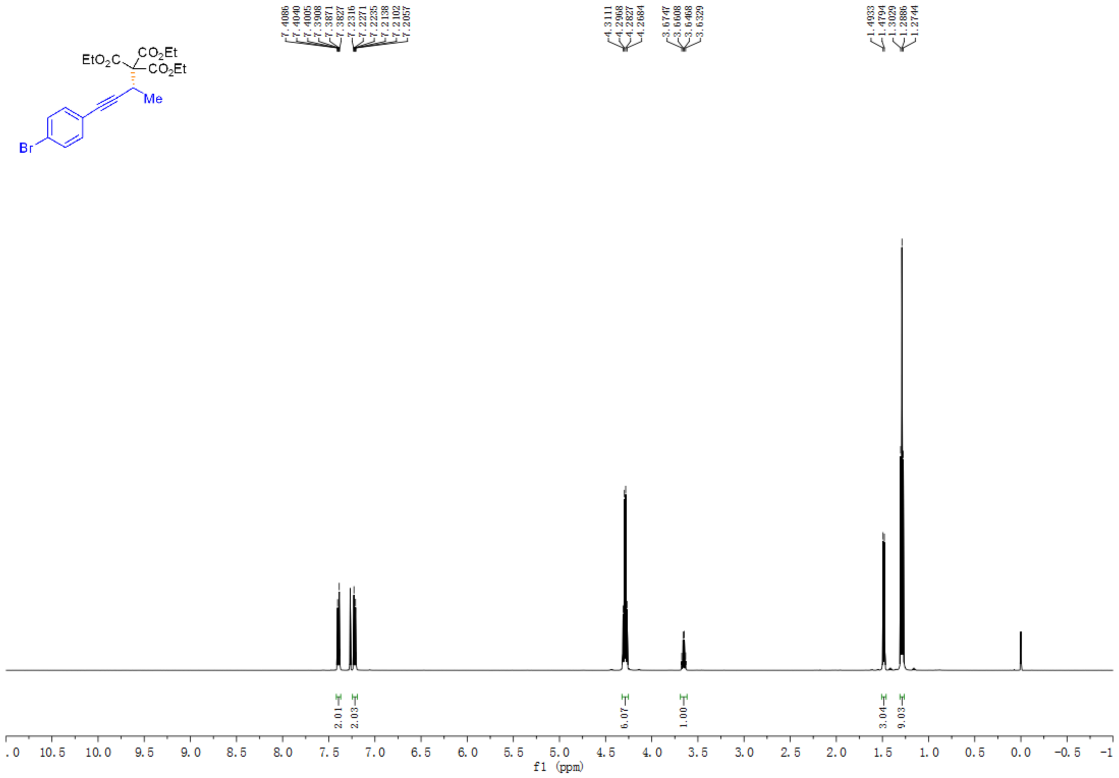

^{13}C NMR spectrum of **3v**

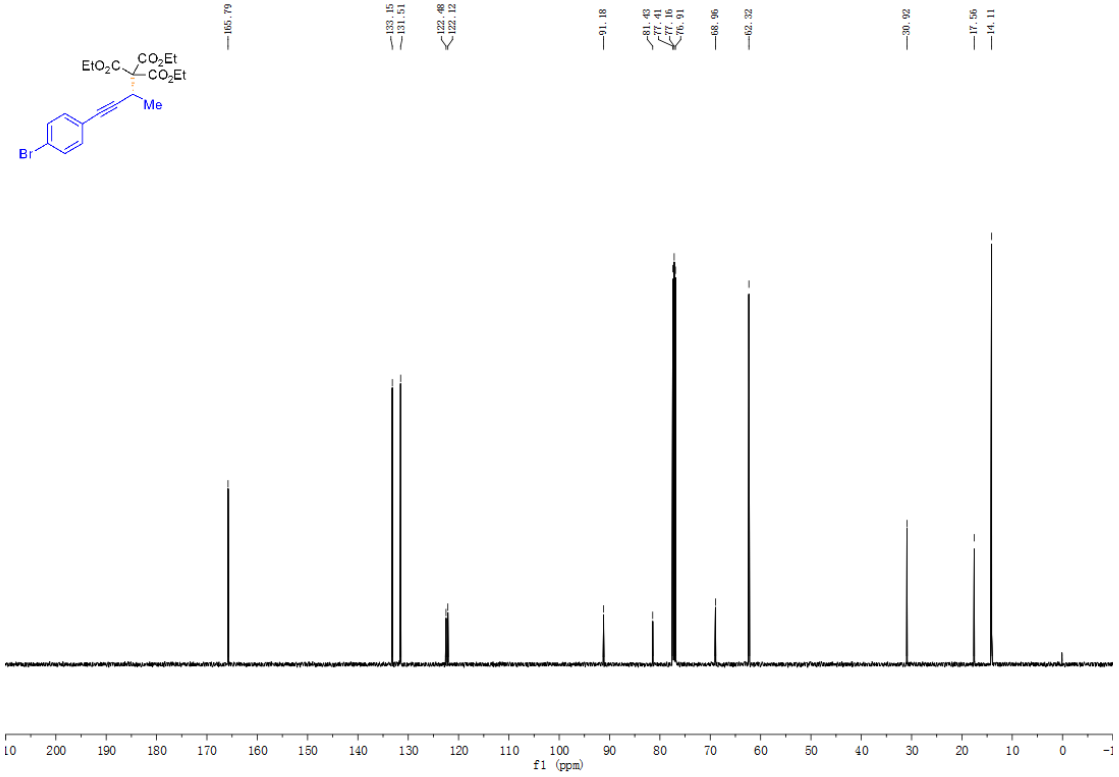

^{1}H NMR spectrum of **3w**

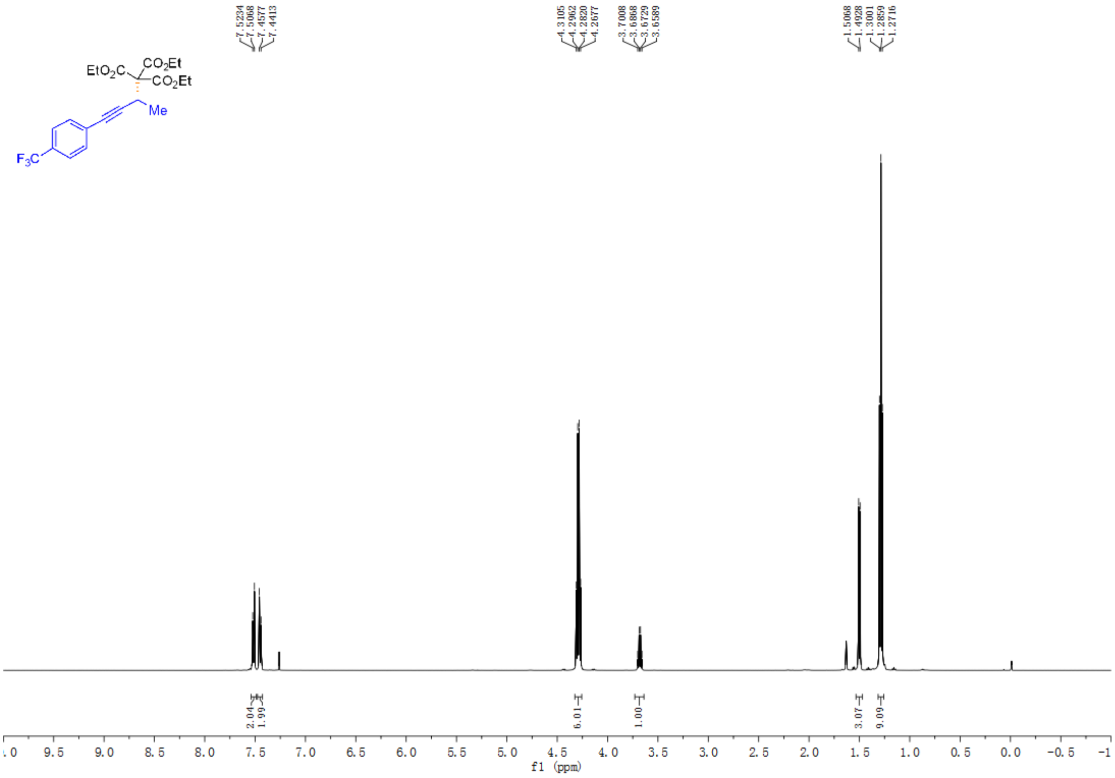

^{13}C NMR spectrum of **3w**

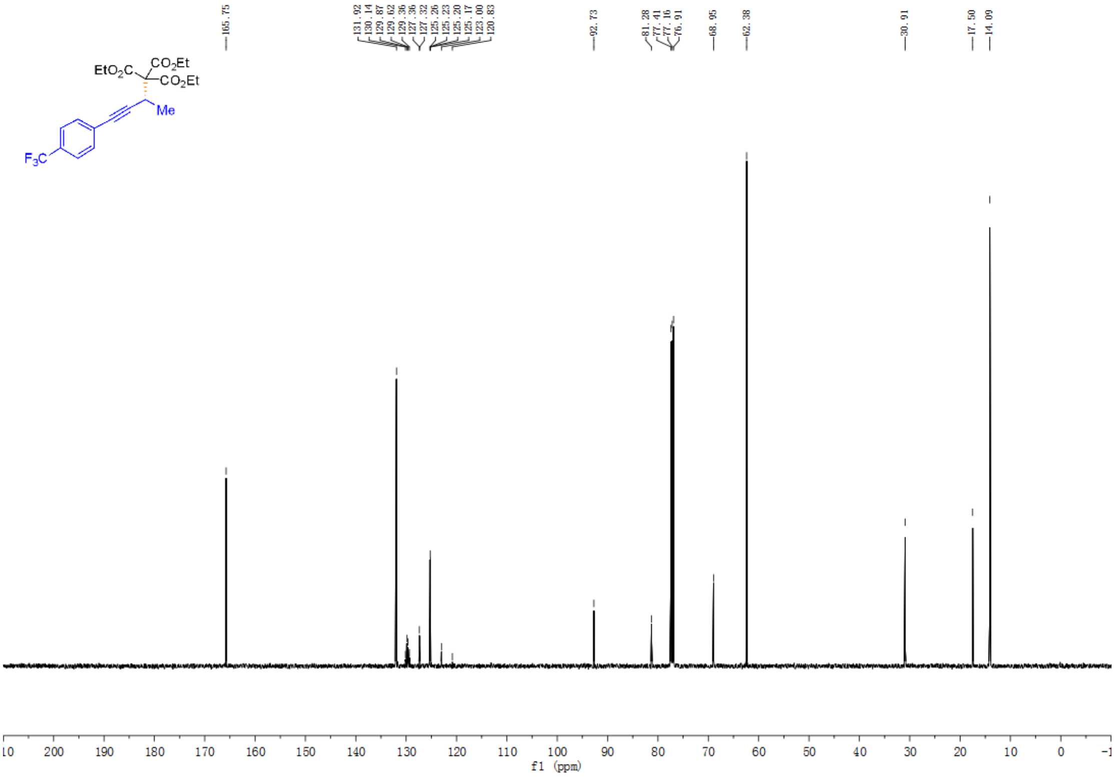

^{19}F NMR spectrum of **3w**

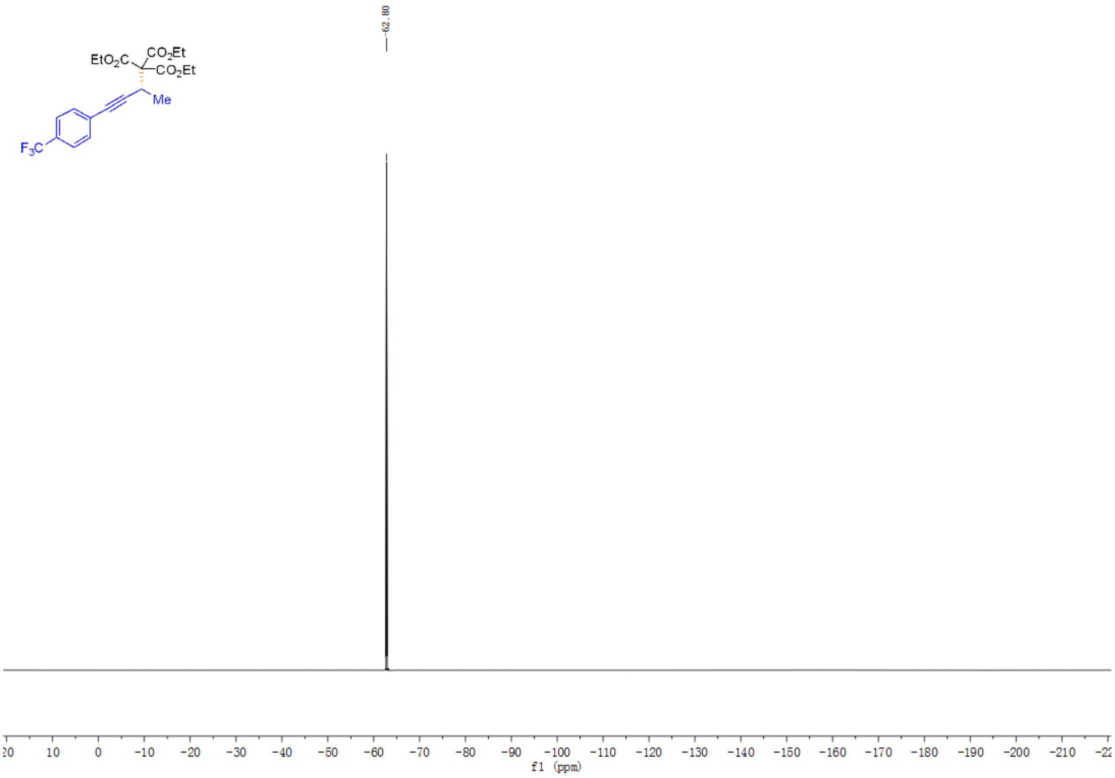

^{1}H NMR spectrum of **3x**

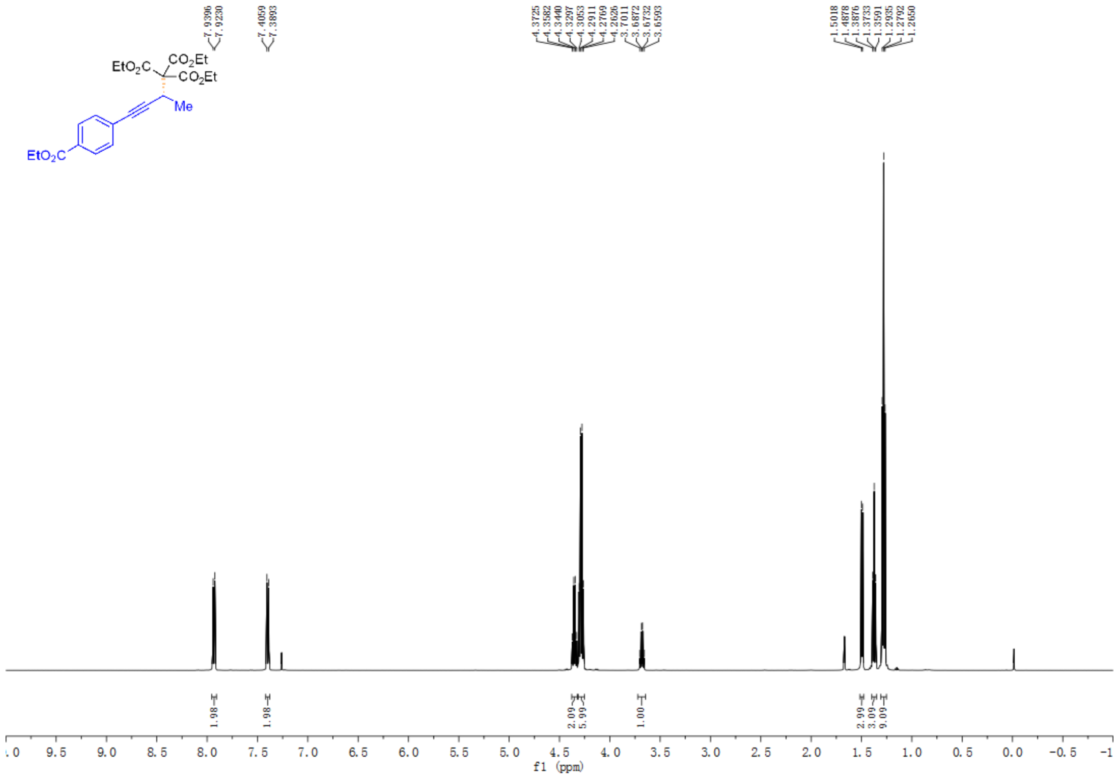

^{13}C NMR spectrum of **3x**

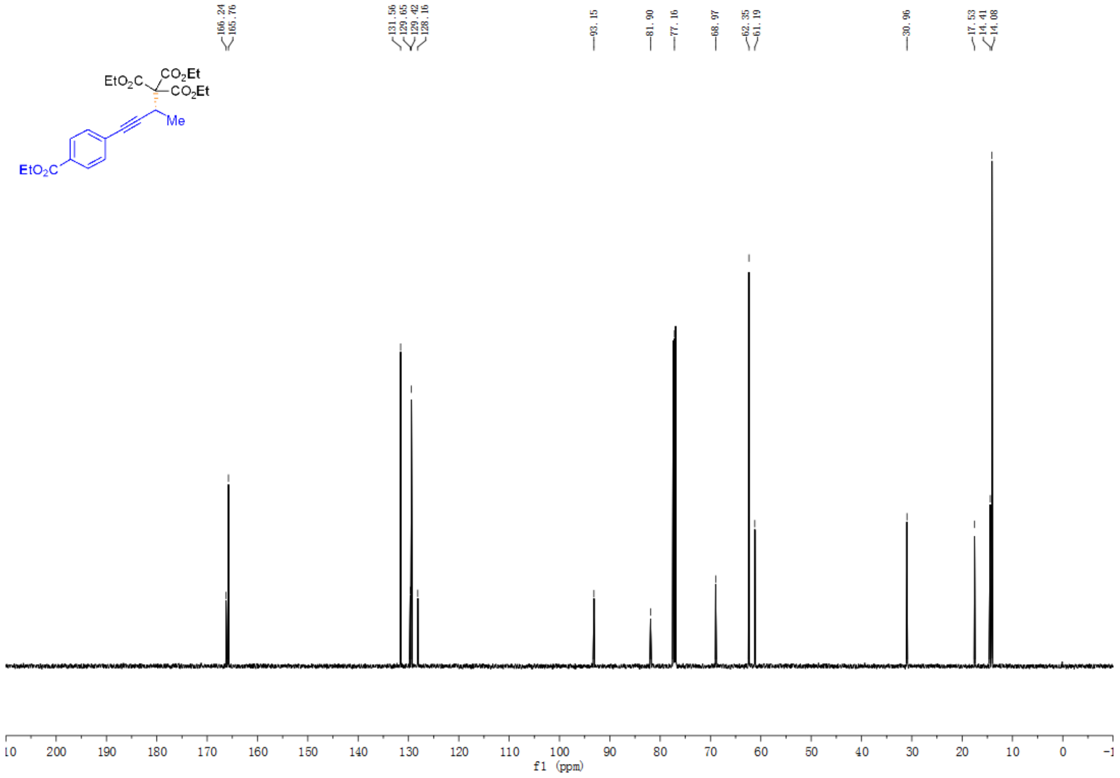

^{1}H NMR spectrum of **3y**

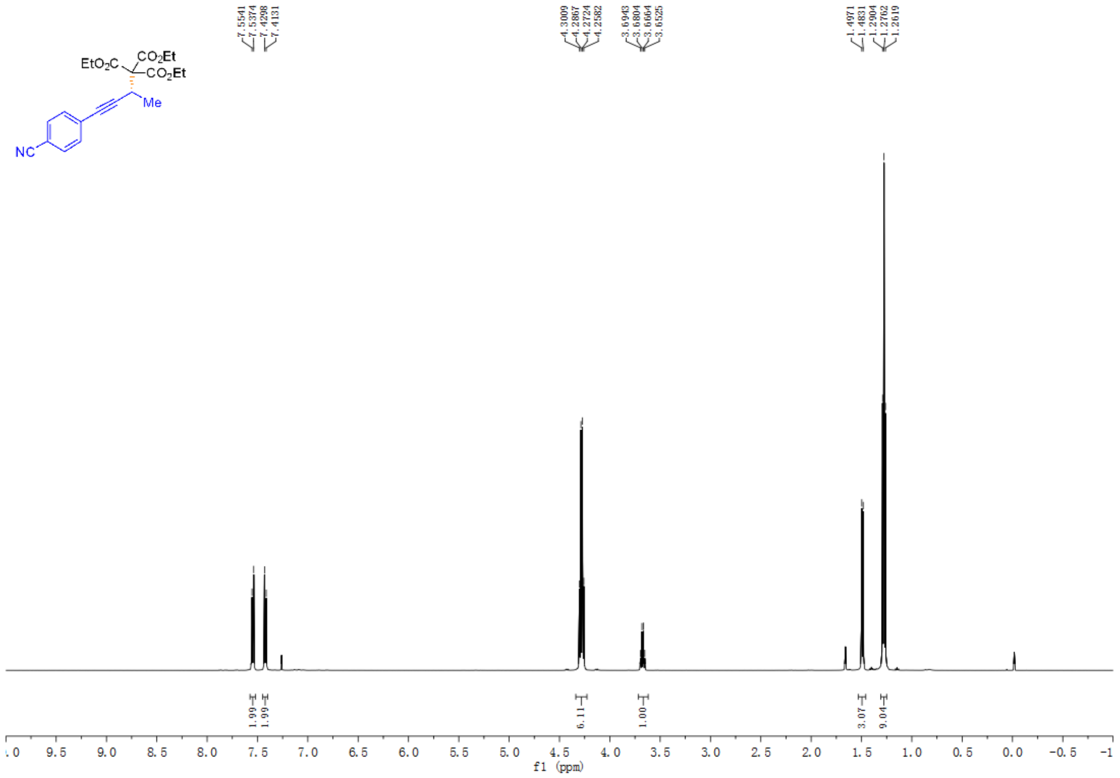

^{13}C NMR spectrum of **3y**

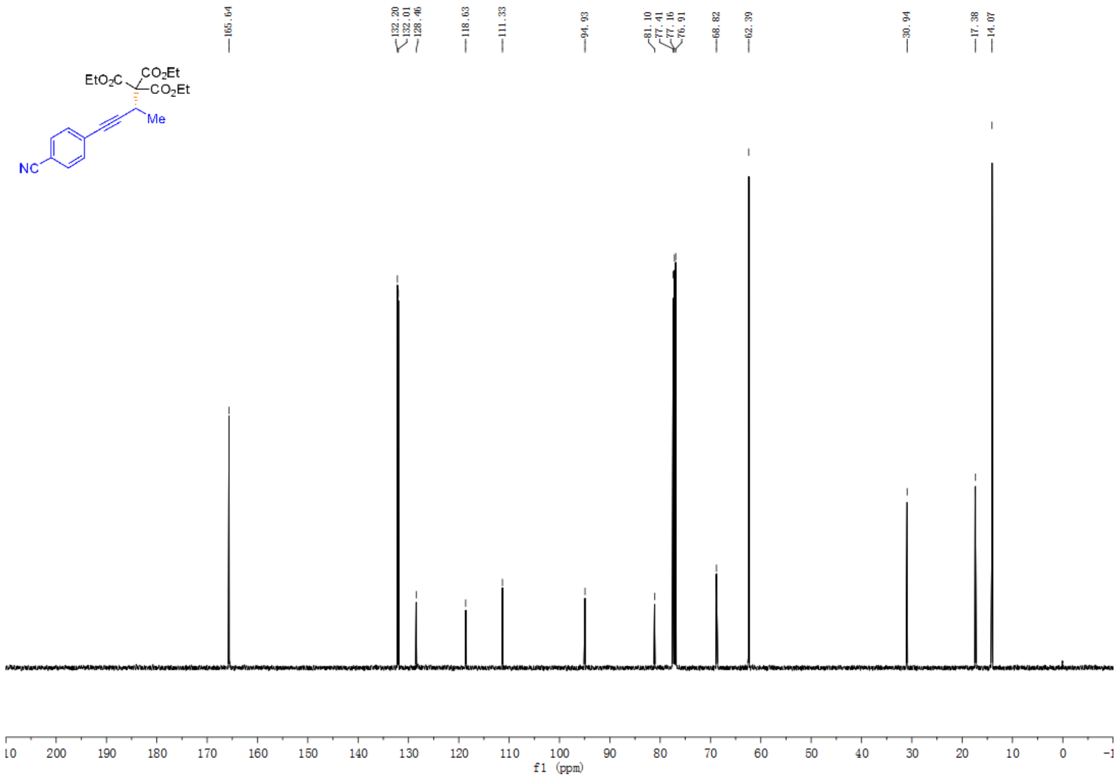

^{1}H NMR spectrum of **3z**

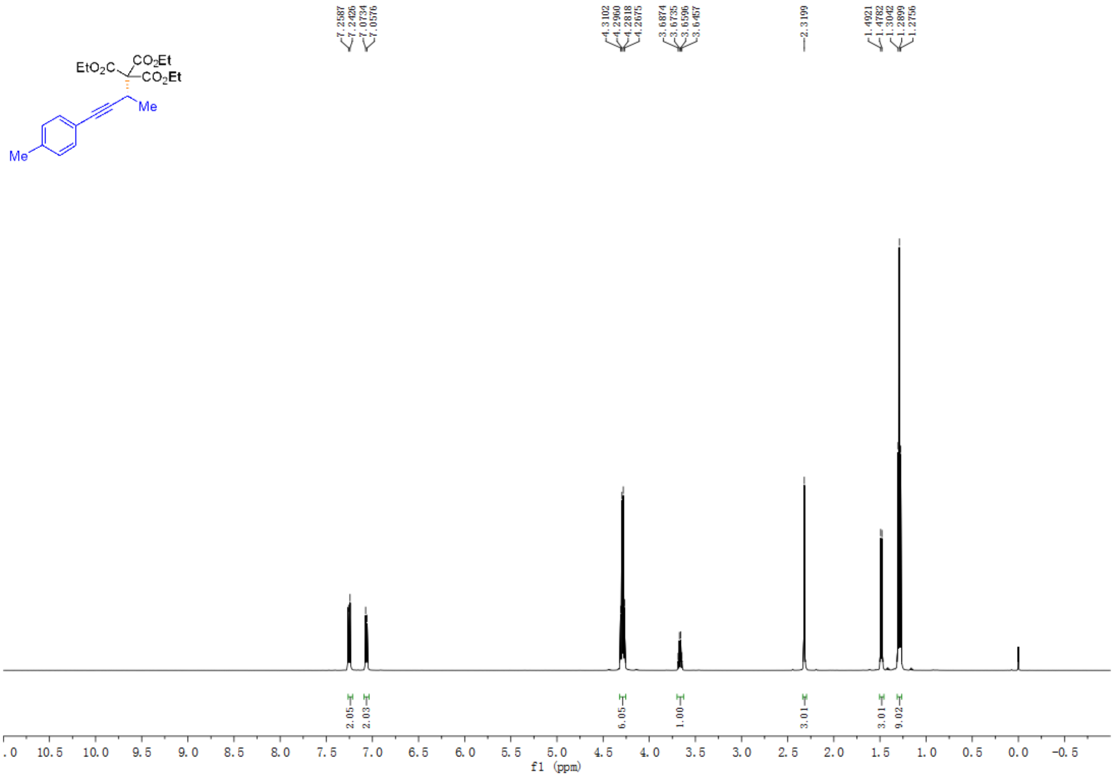

^{13}C NMR spectrum of **3z**

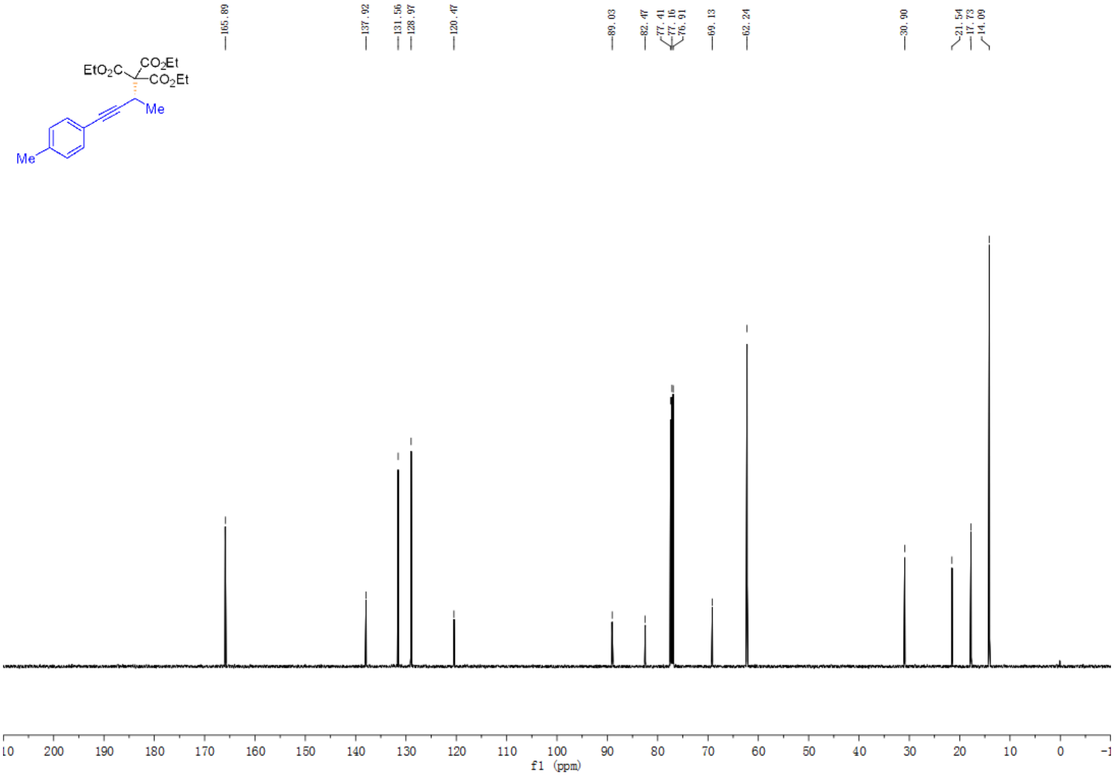

^{1}H NMR spectrum of **3aa**

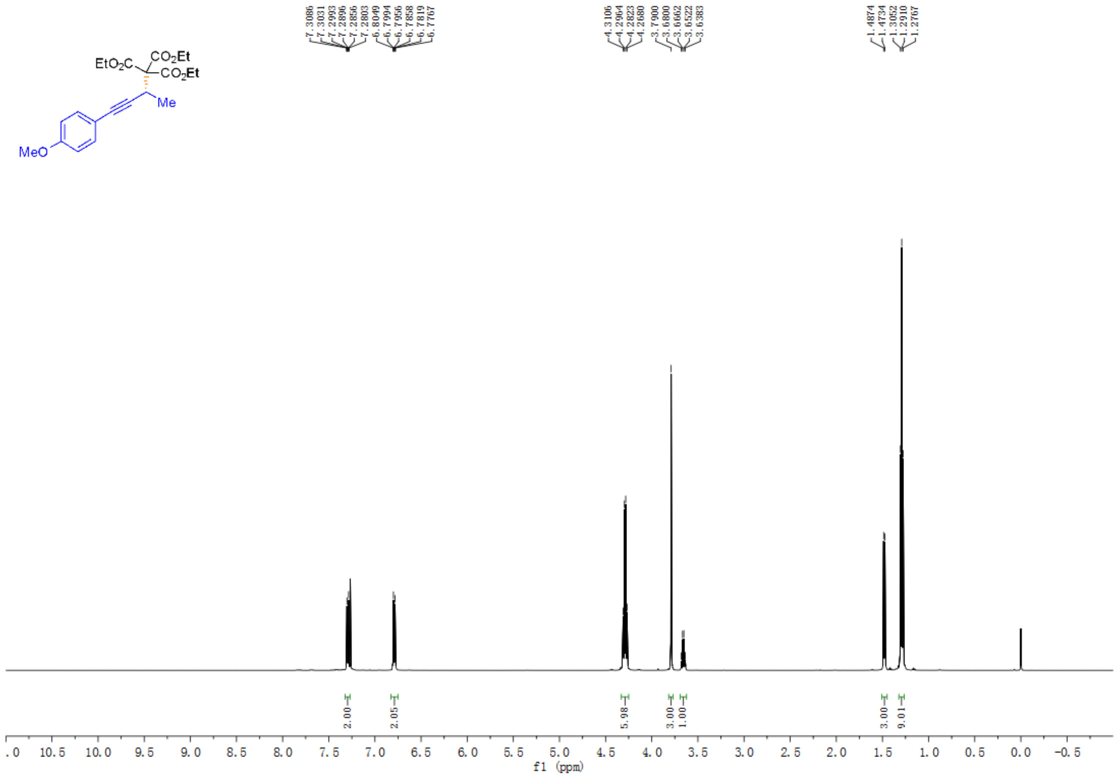

^{13}C NMR spectrum of **3aa**

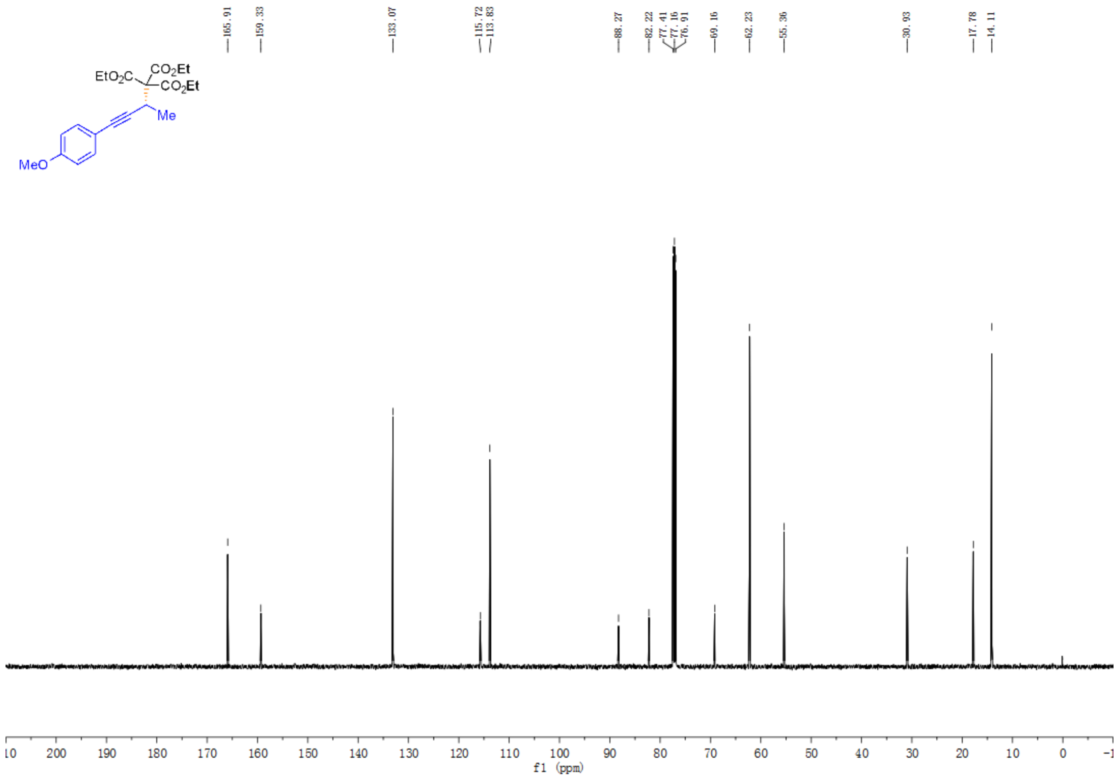

^{1}H NMR spectrum of **3ab**

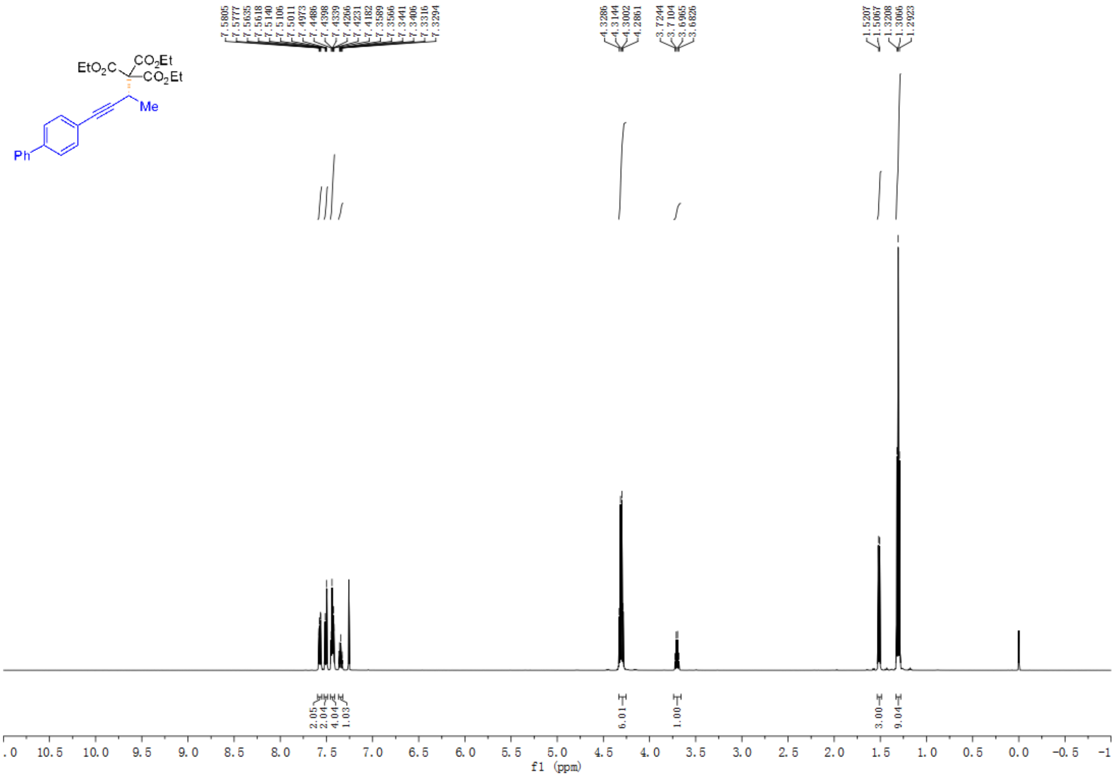

^{13}C NMR spectrum of **3ab**

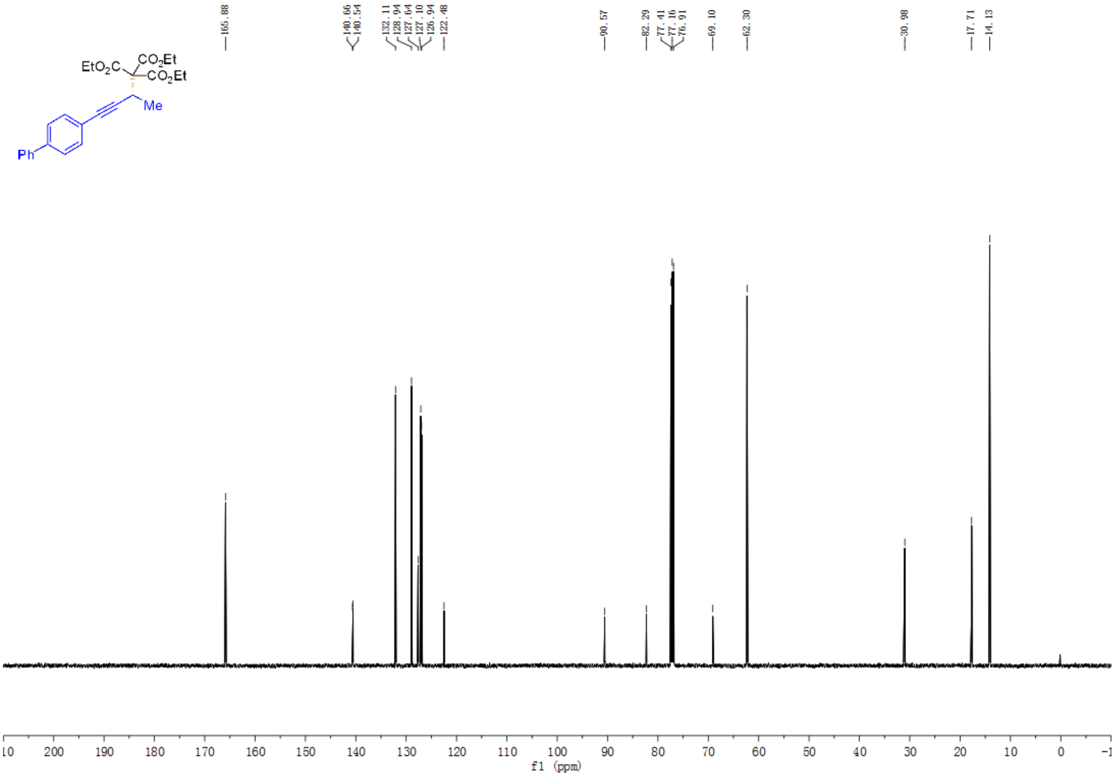

^{1}H NMR spectrum of **3ac**

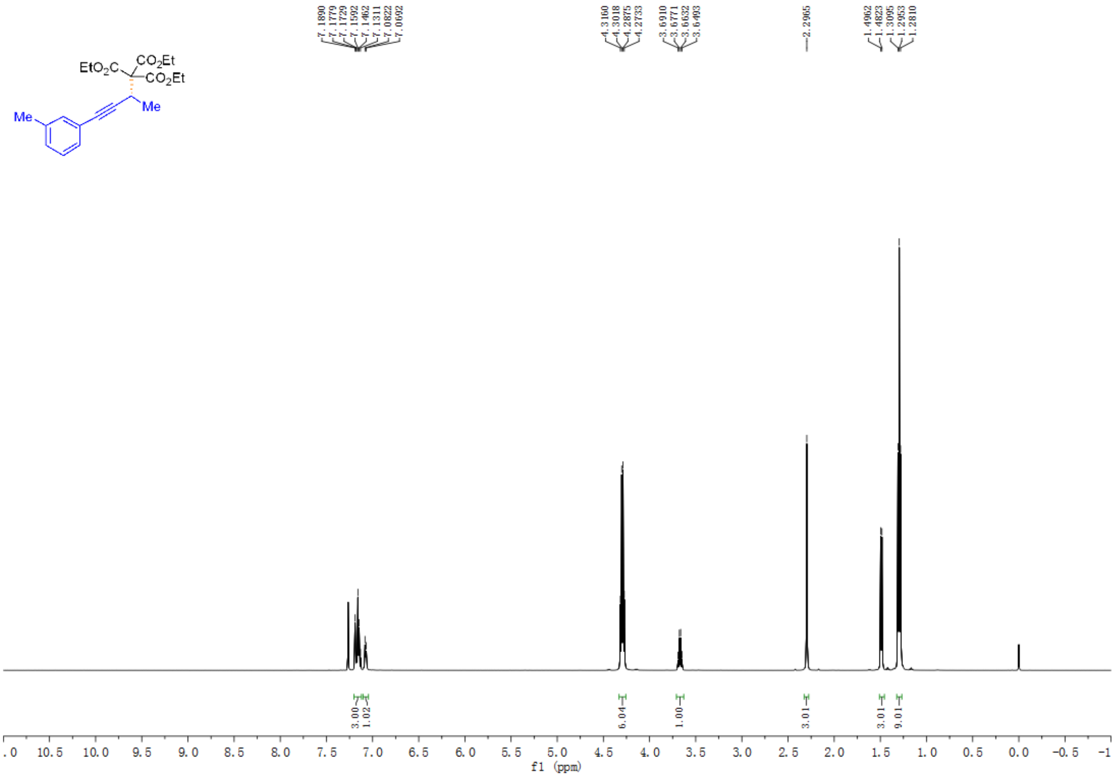

^{13}C NMR spectrum of **3ac**

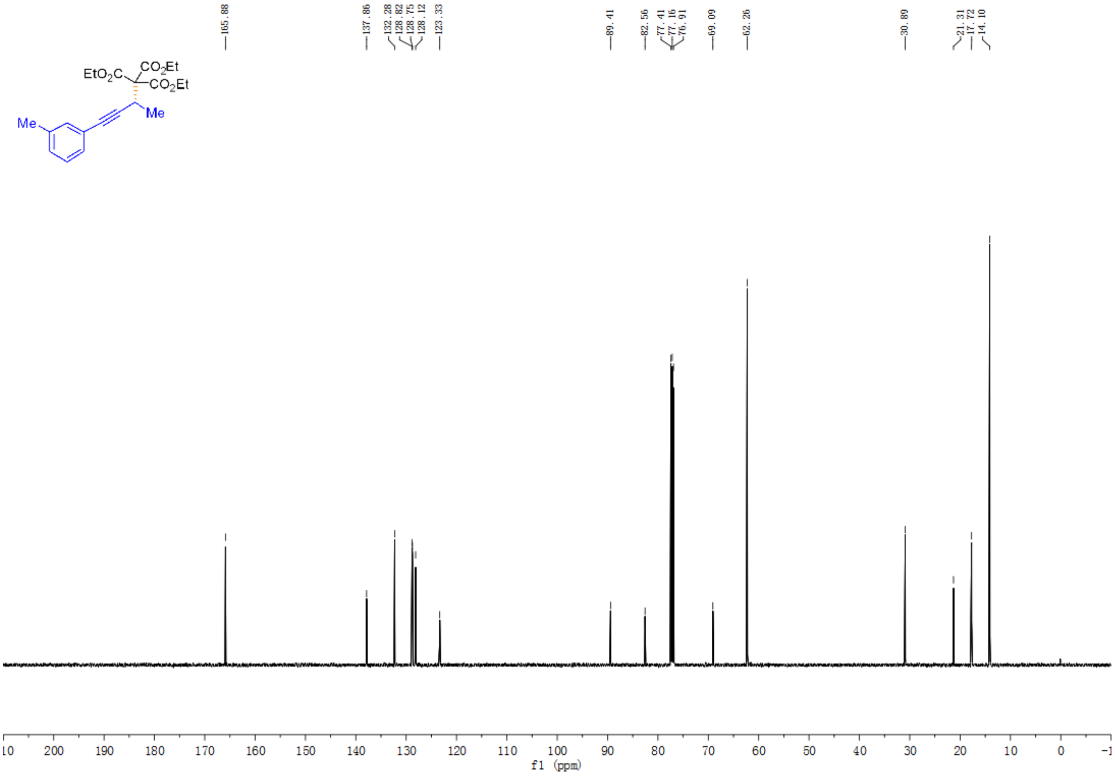

^{1}H NMR spectrum of **3ad**

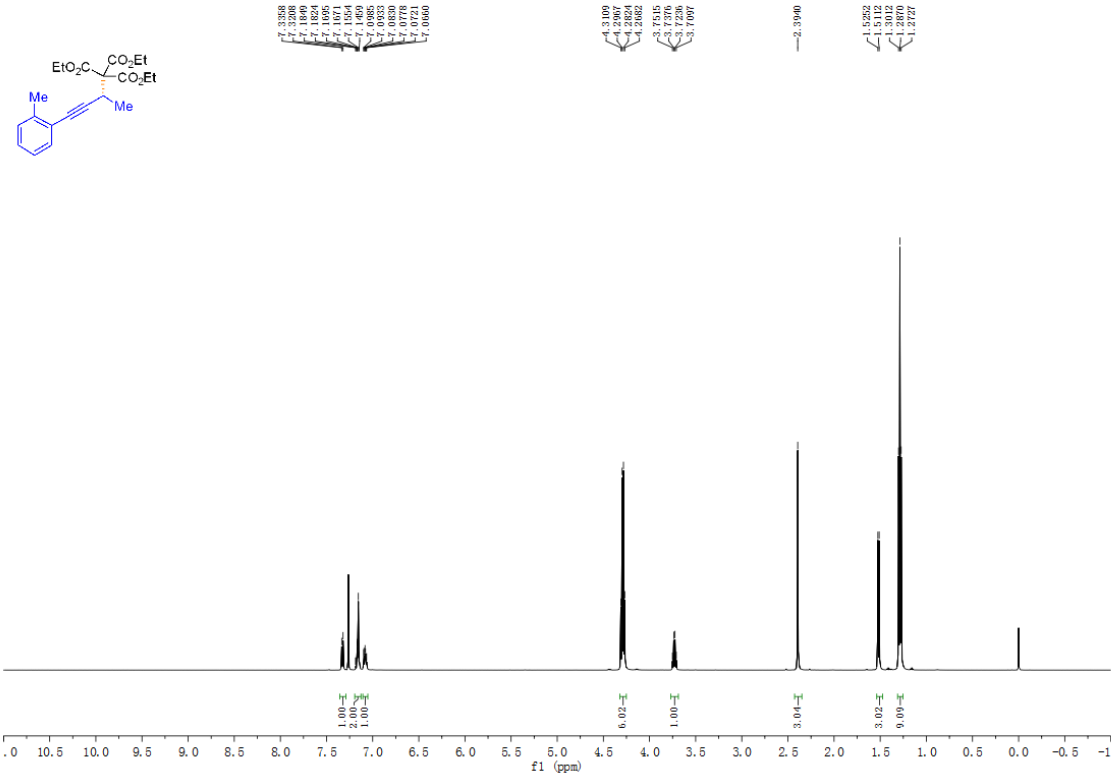

^{13}C NMR spectrum of **3ad**

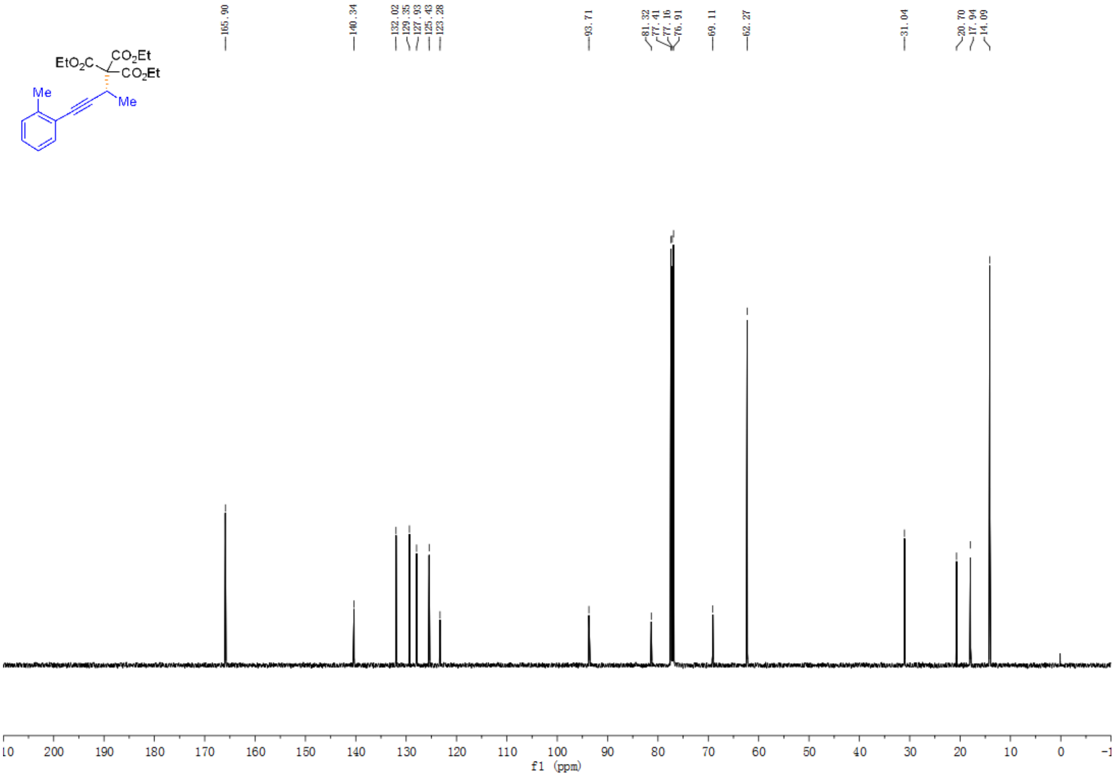

^{1}H NMR spectrum of **3ae**

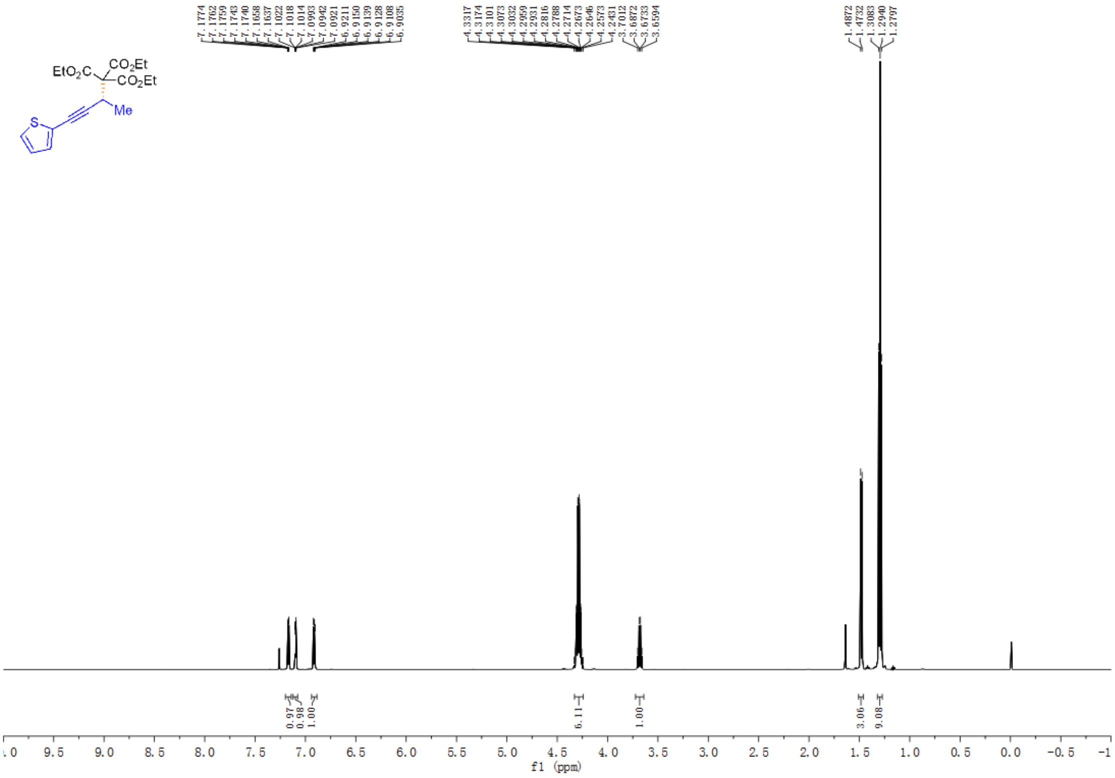

^{13}C NMR spectrum of **3ae**

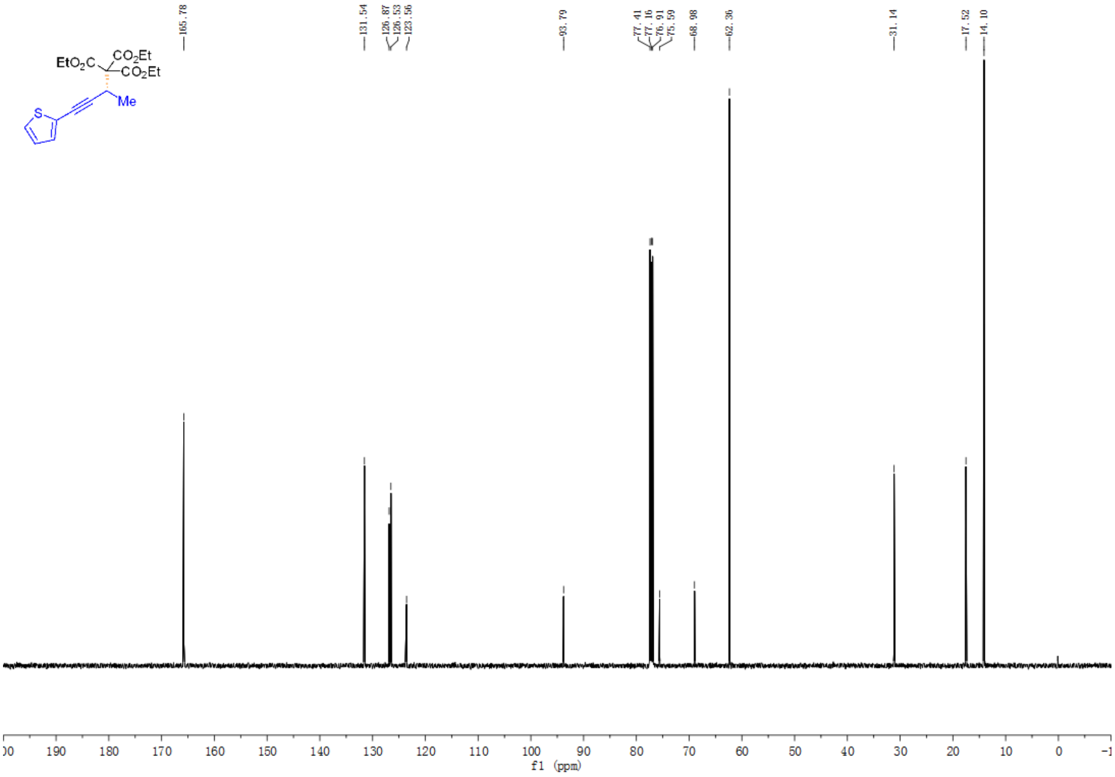

^{1}H NMR spectrum of **3af**

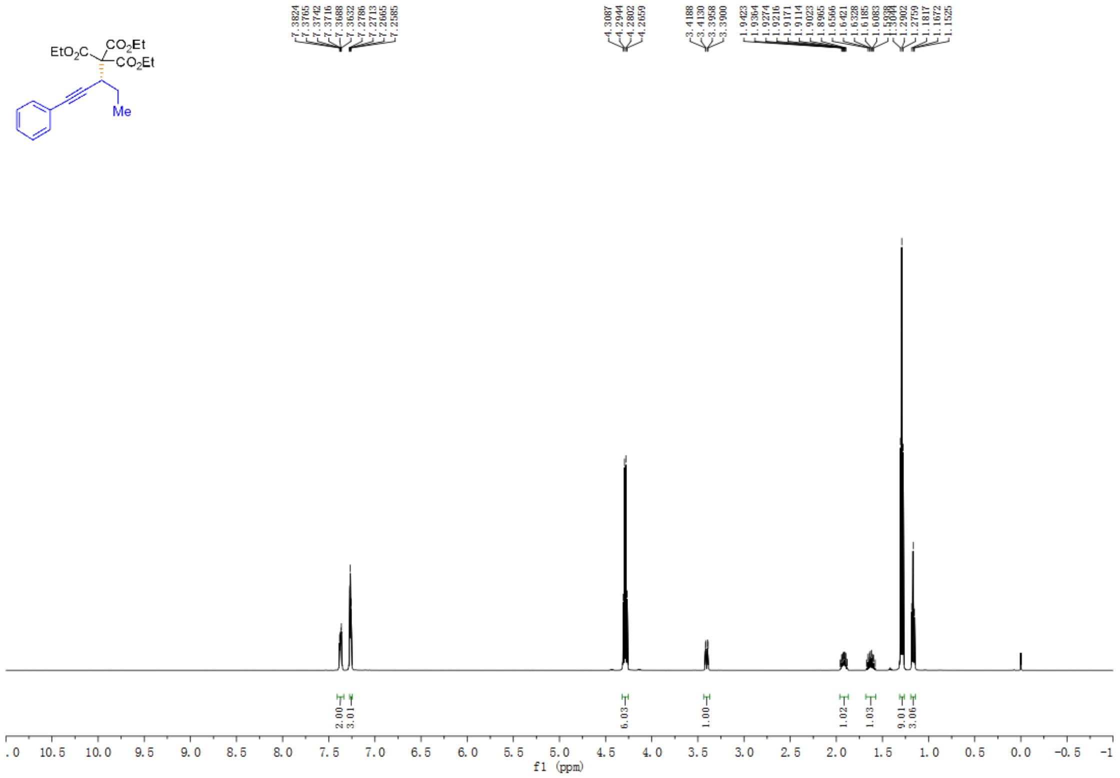

^{13}C NMR spectrum of **3af**

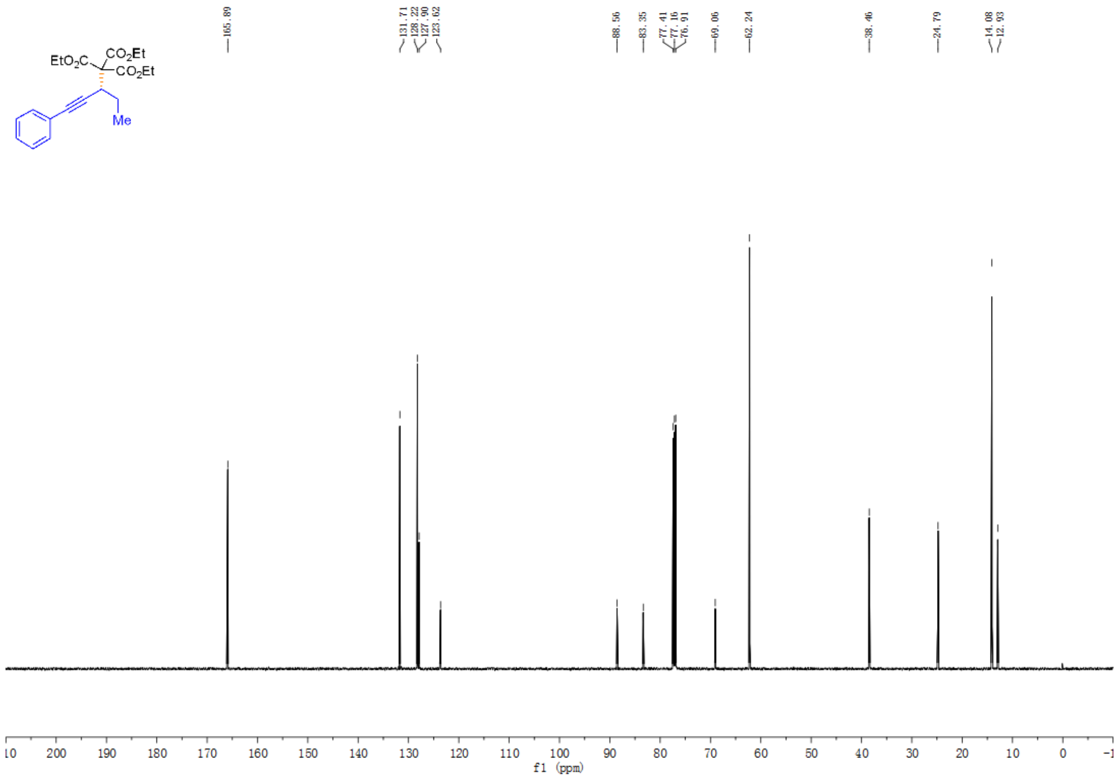

^{1}H NMR spectrum of **3ag**

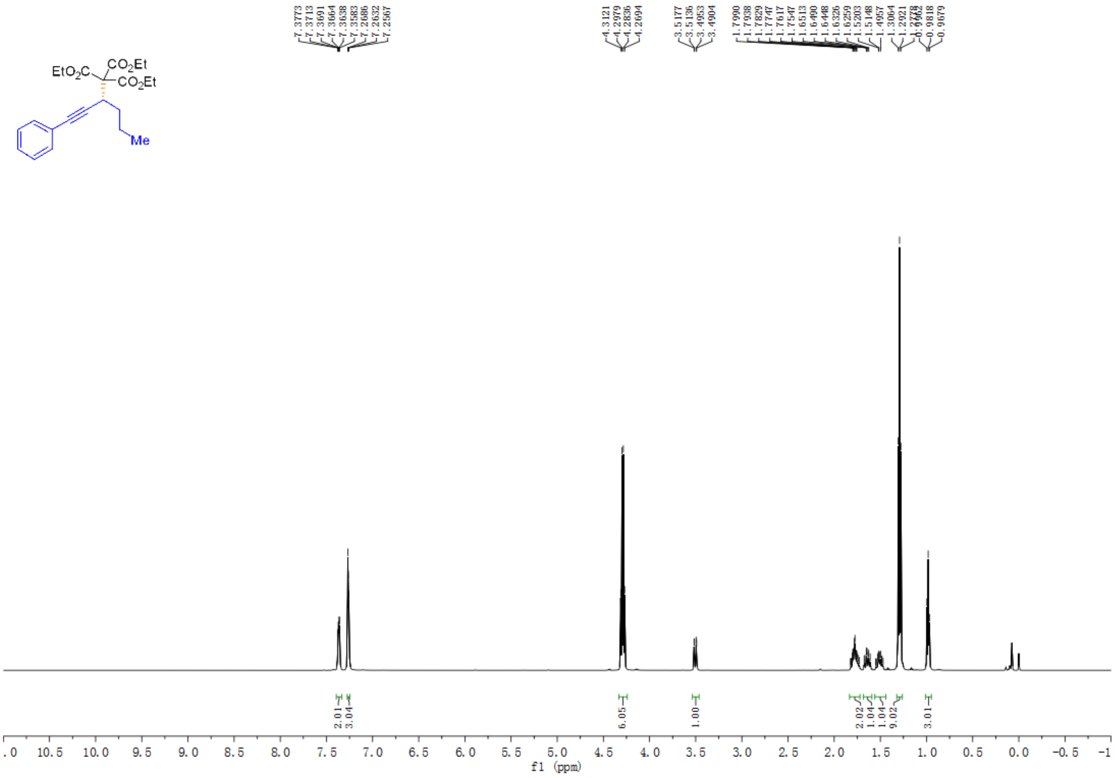

^{13}C NMR spectrum of **3ag**

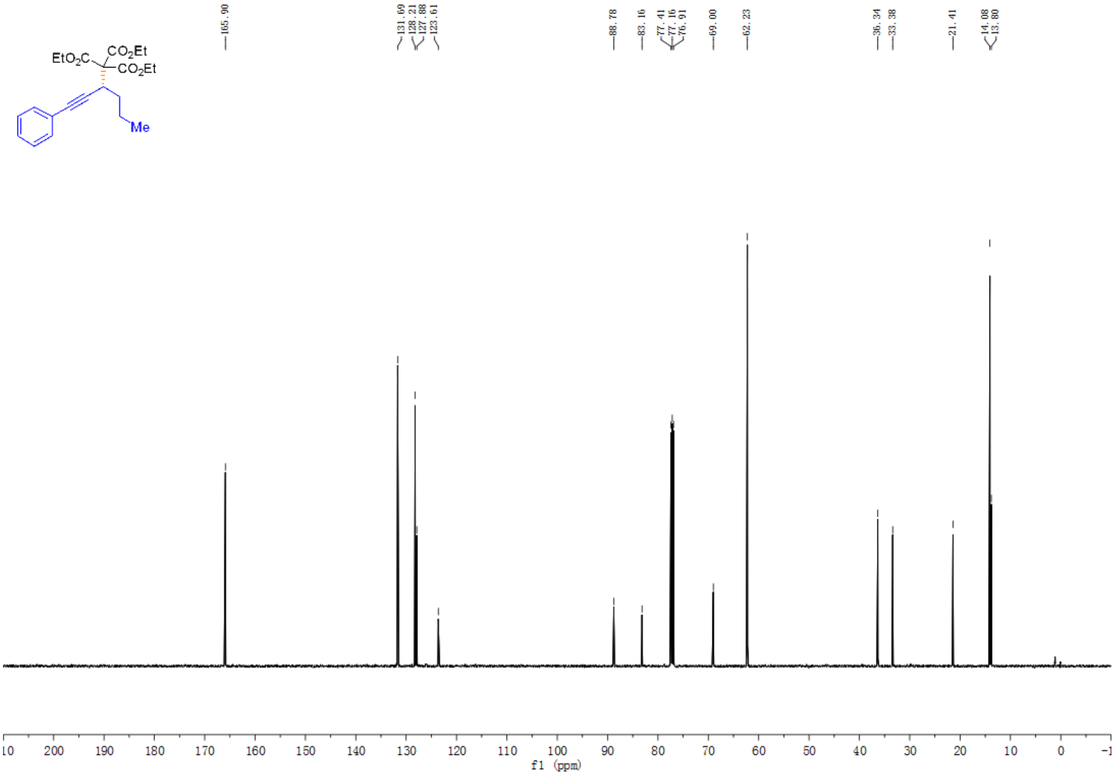

^{1}H NMR spectrum of **3ah**

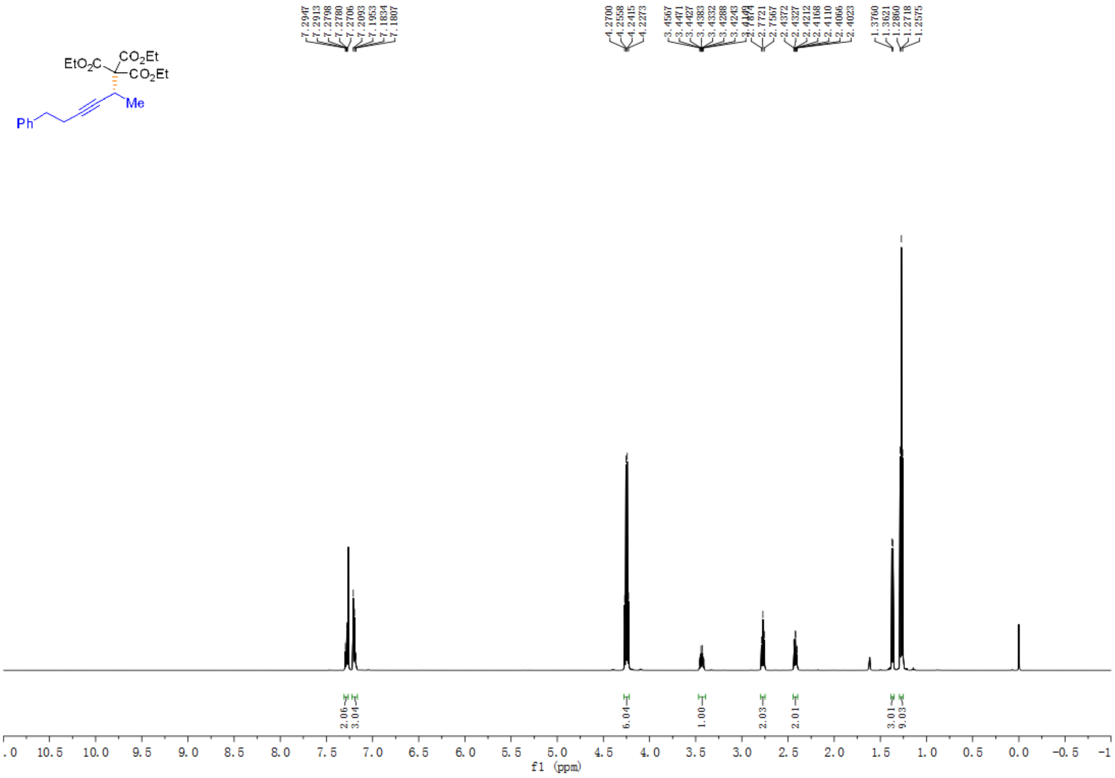

^{13}C NMR spectrum of **3ah**

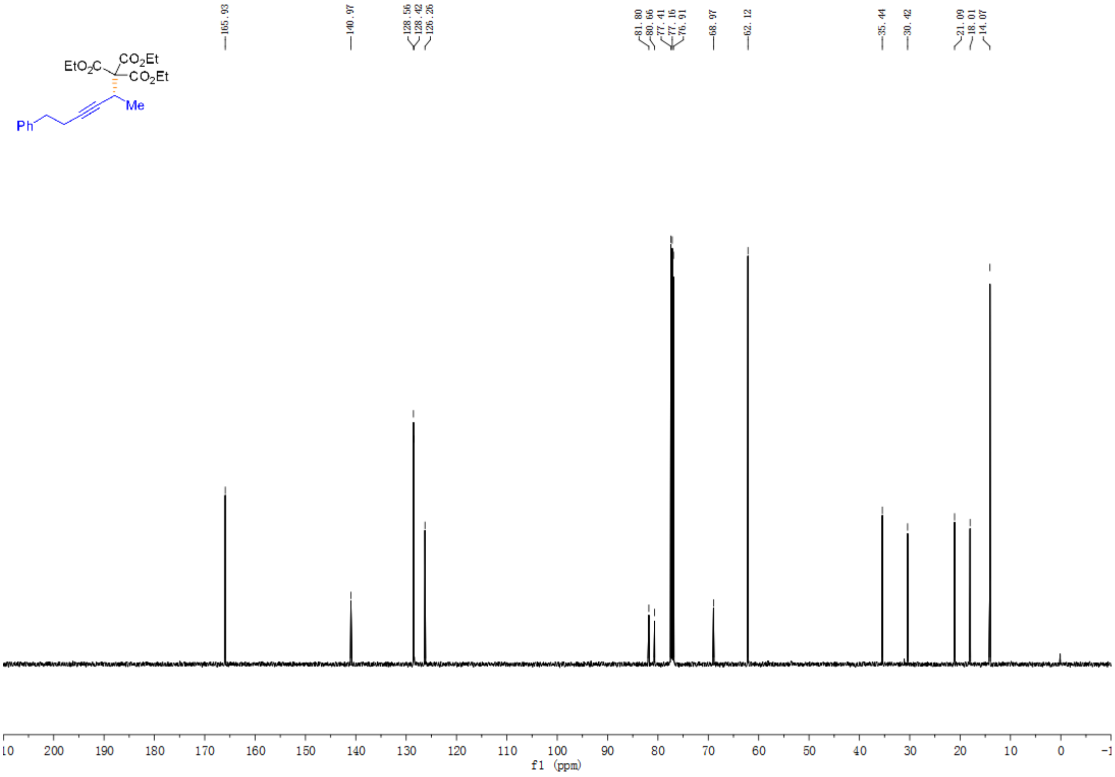

^{1}H NMR spectrum of **3ai**

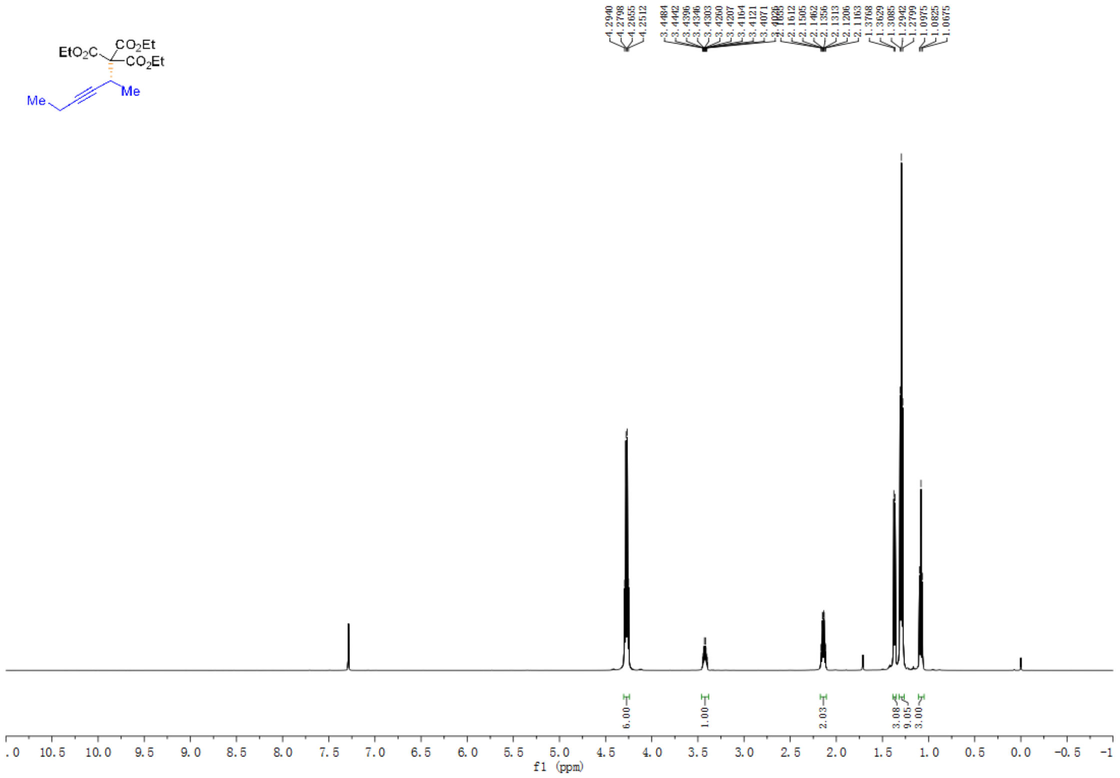

^{13}C NMR spectrum of **3ai**

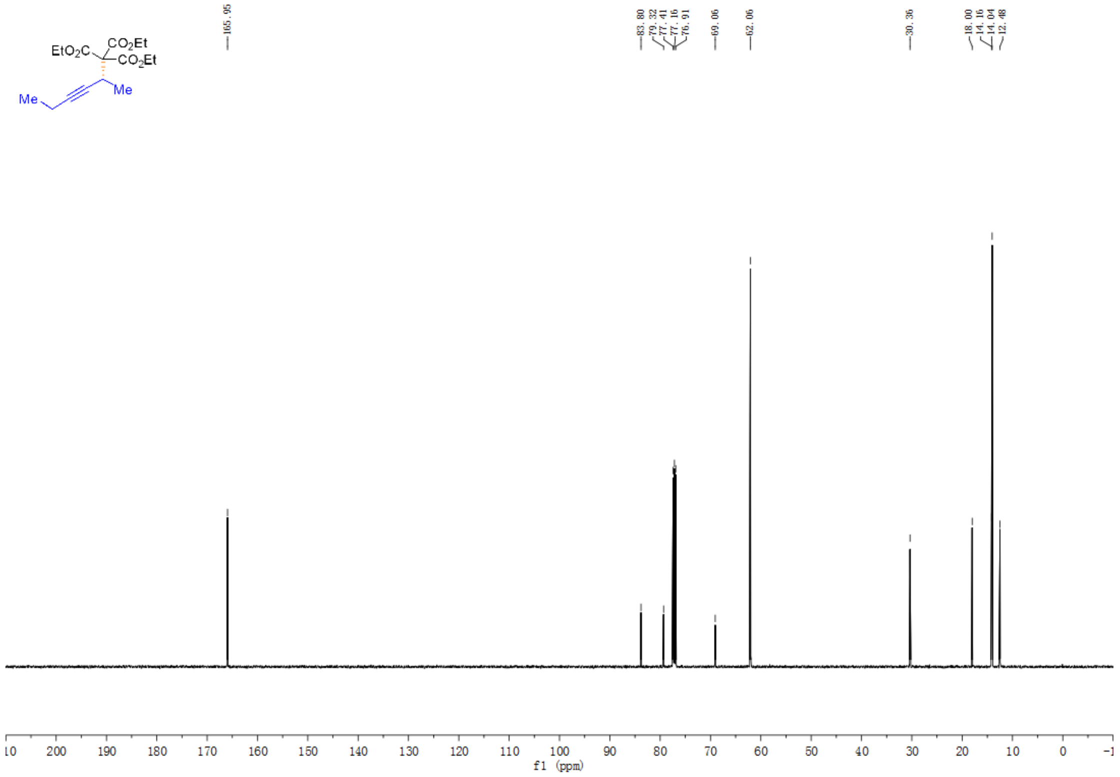

^{1}H NMR spectrum of **3aj**

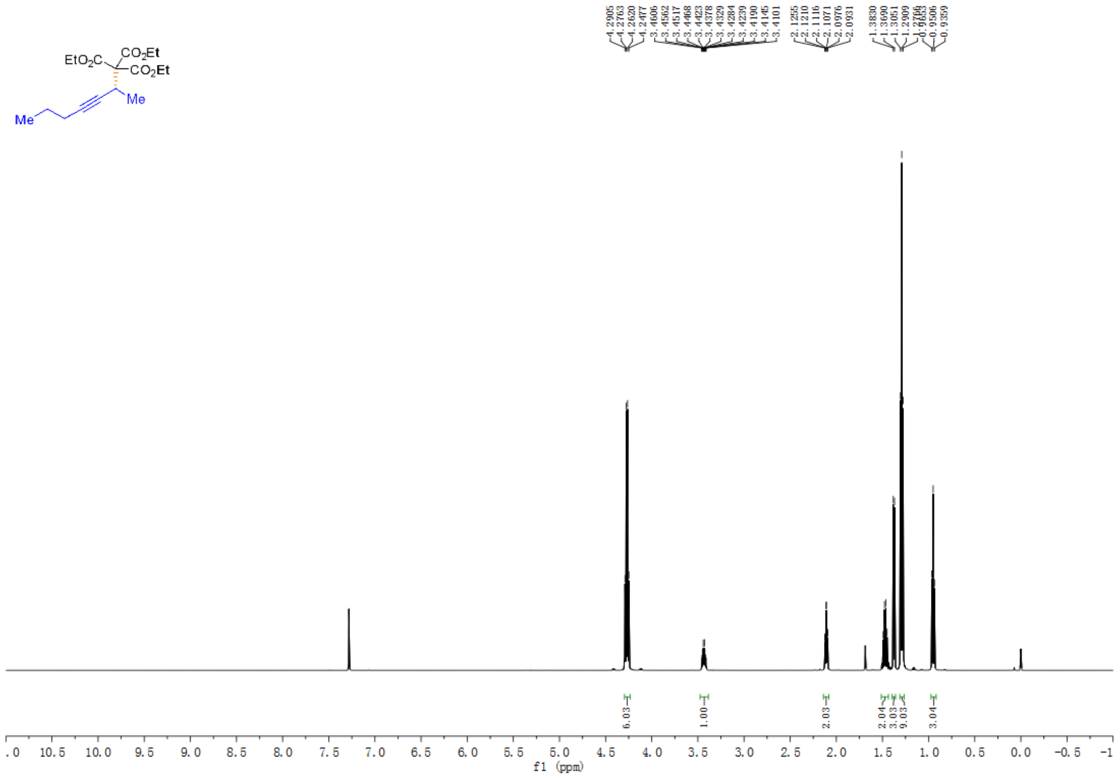

^{13}C NMR spectrum of **3aj**

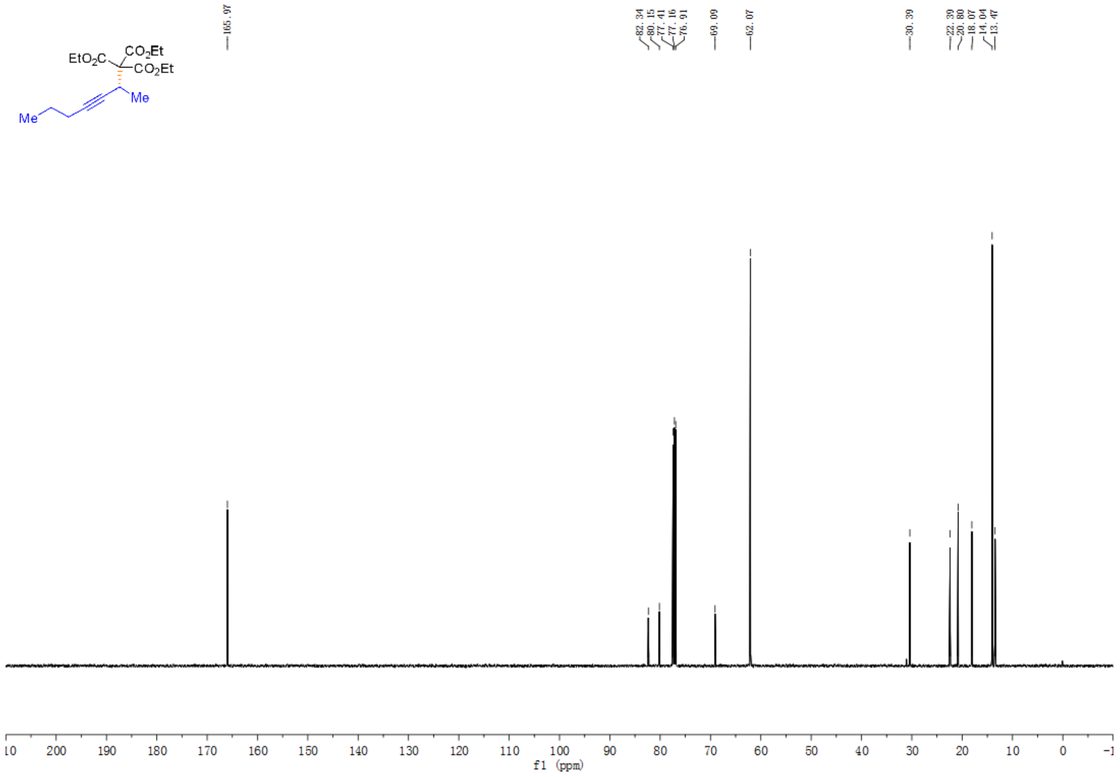

^{1}H NMR spectrum of **5**

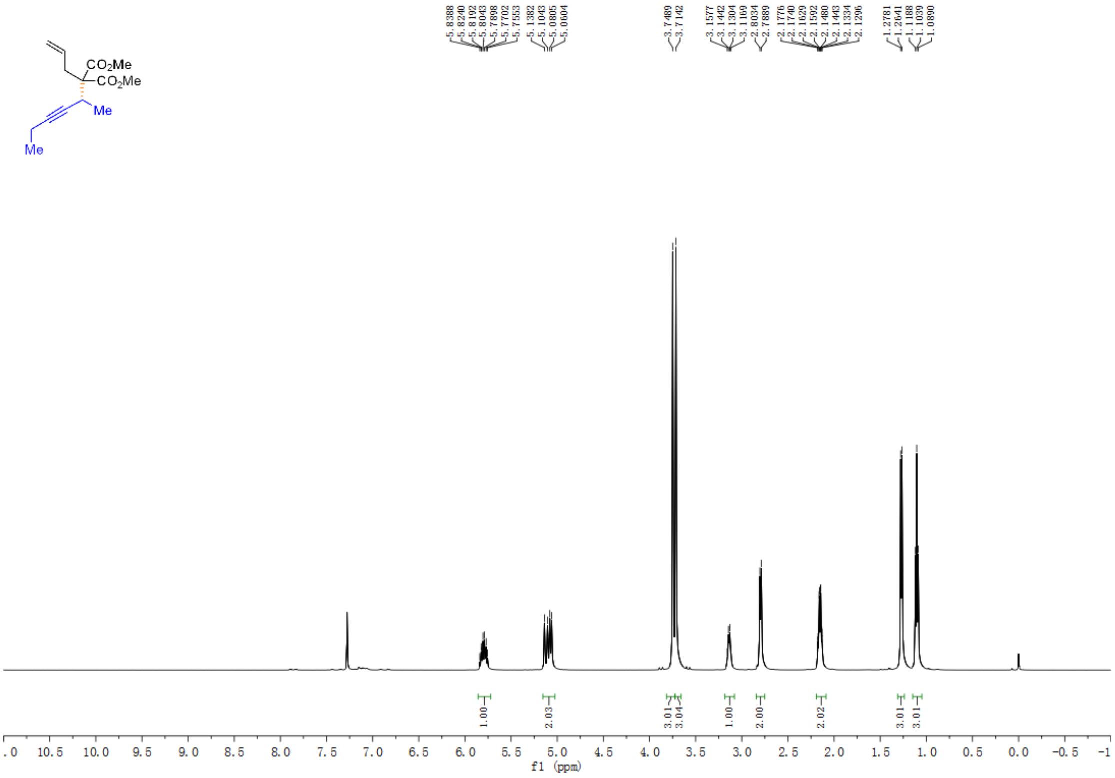

^{13}C NMR spectrum of **5**

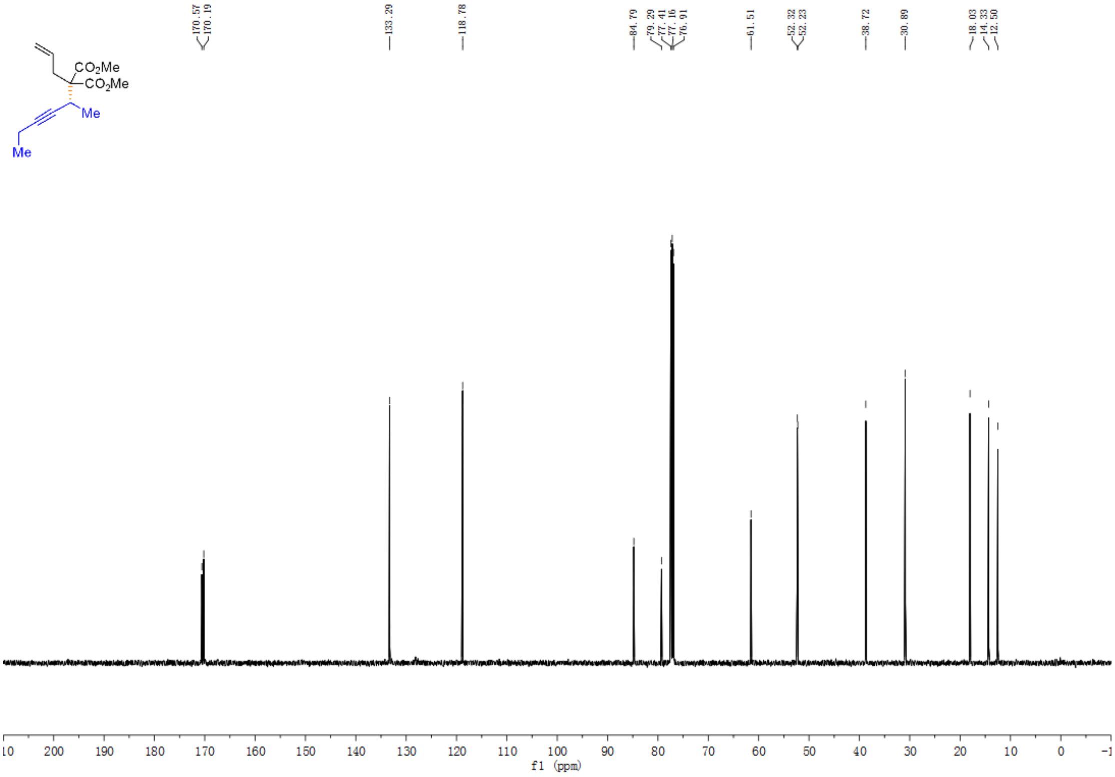

^{1}H NMR spectrum of **6**

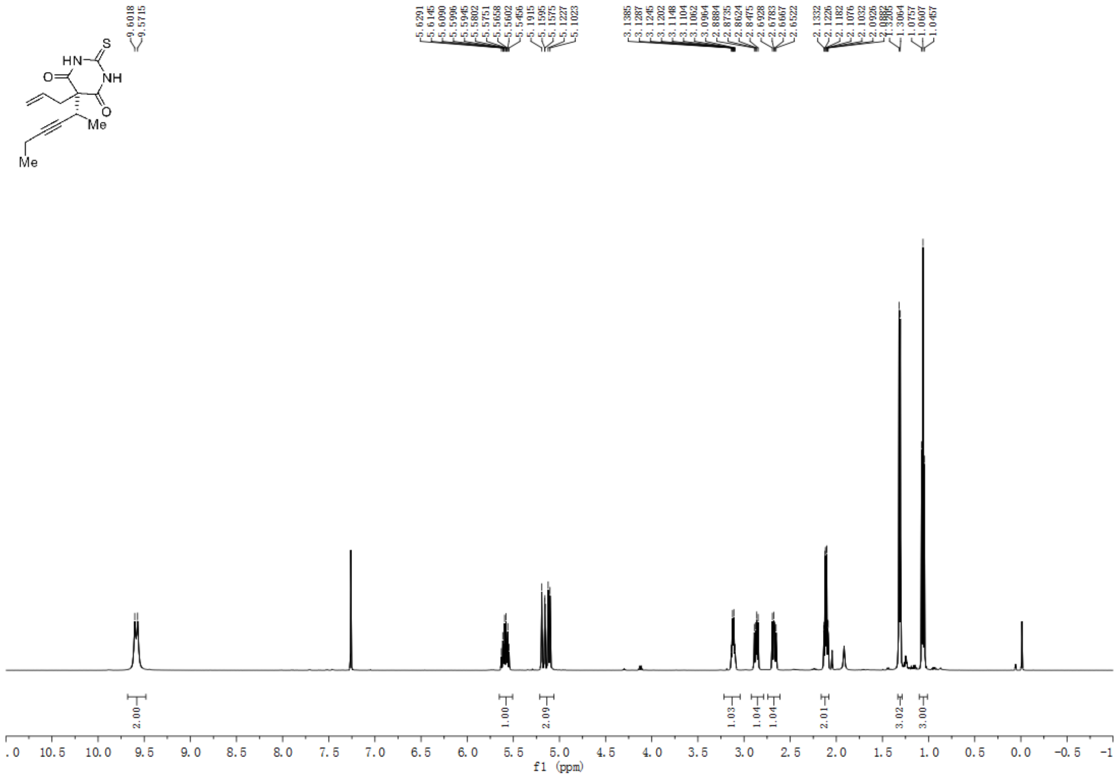

^{13}C NMR spectrum of **6**

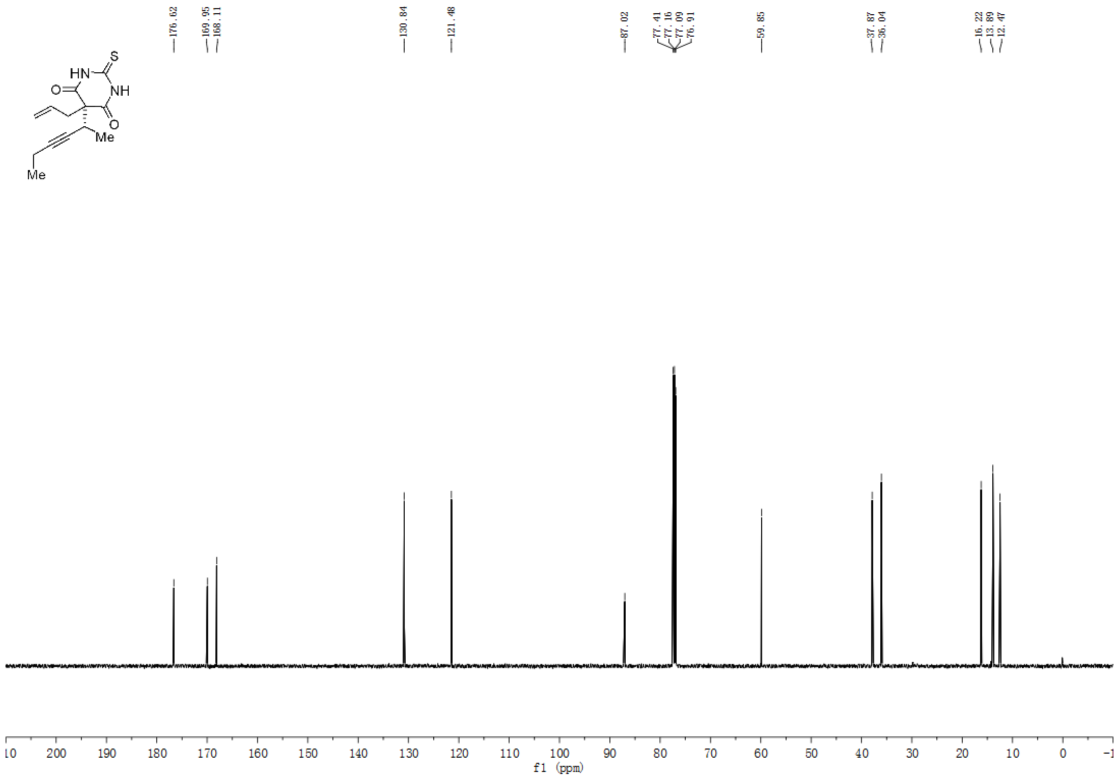

^{1}H NMR spectrum of **7**

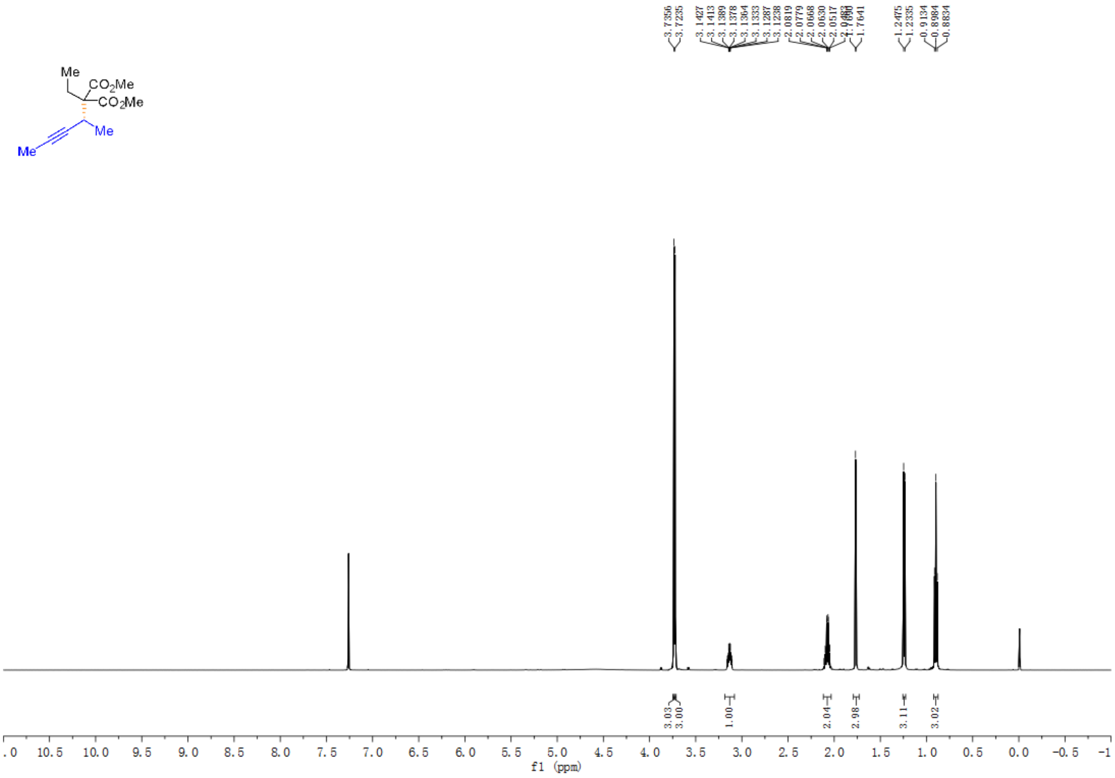

^{13}C NMR spectrum of **7**

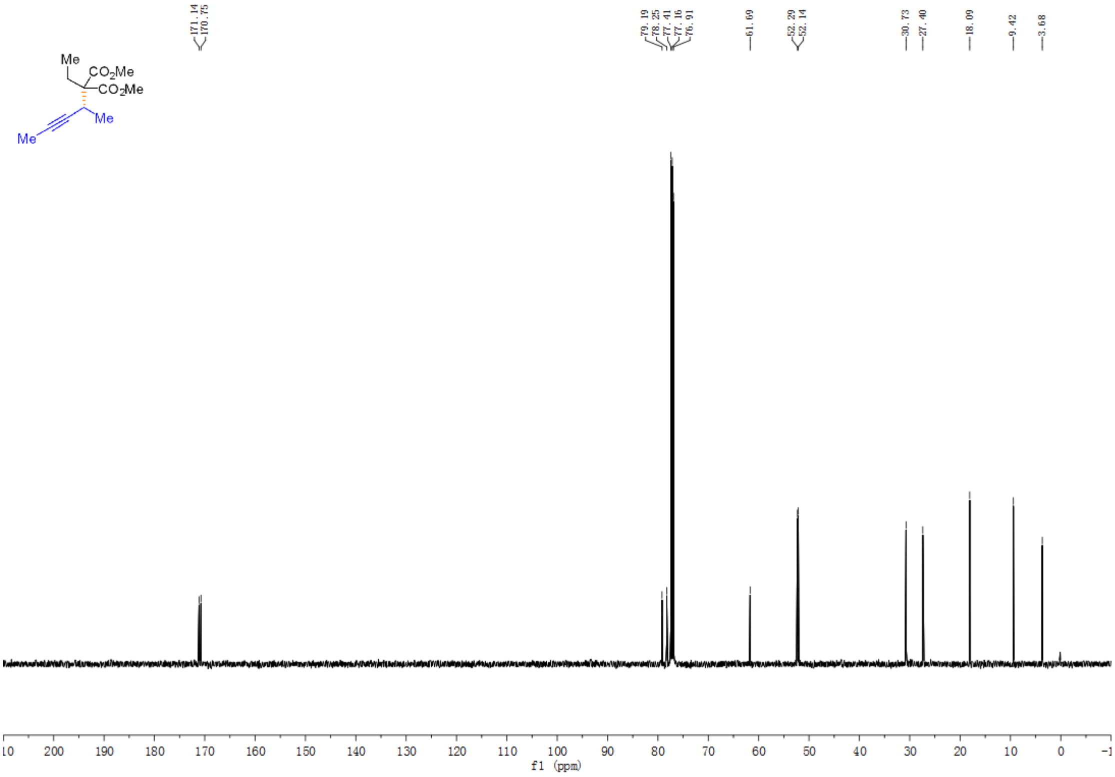

^{1}H NMR spectrum of **8**

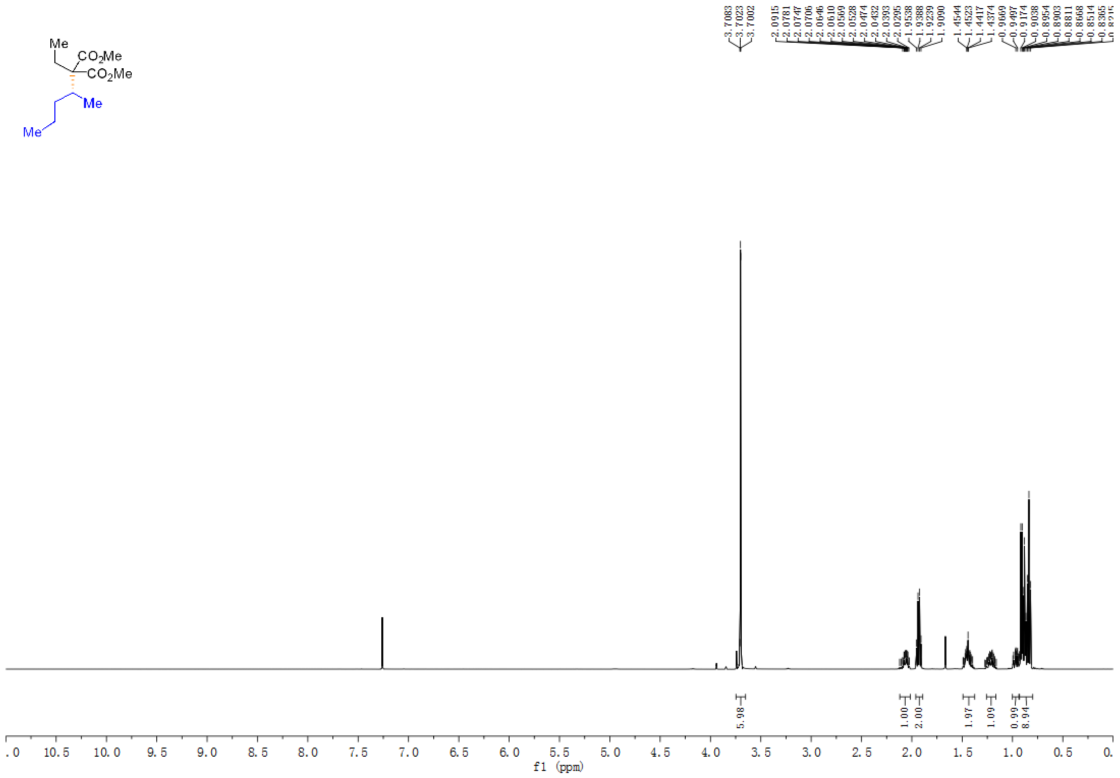

^{13}C NMR spectrum of **8**

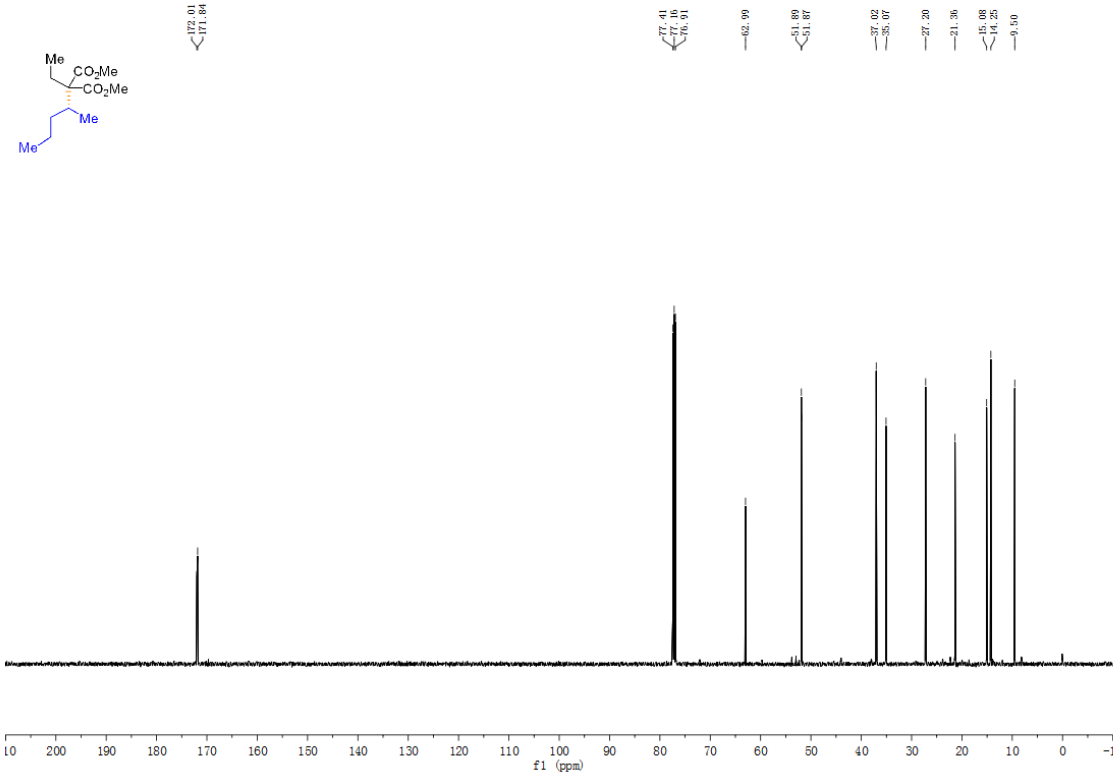

^{1}H NMR spectrum of **9**

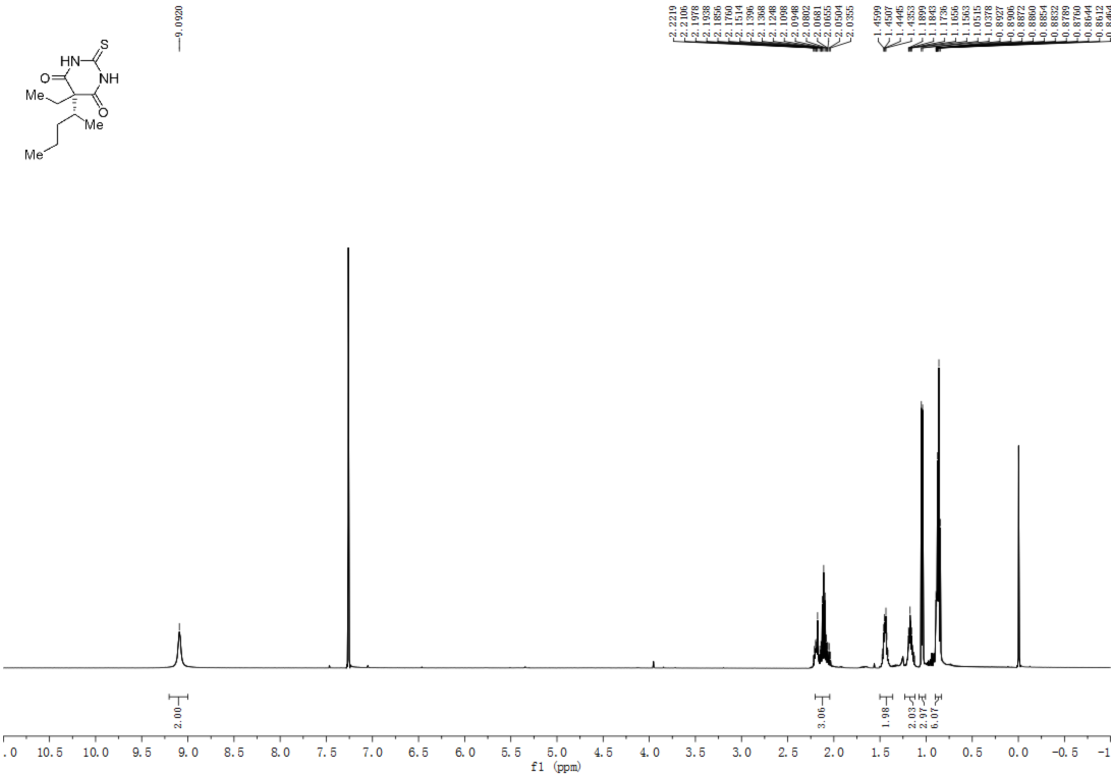

^{13}C NMR spectrum of **9**

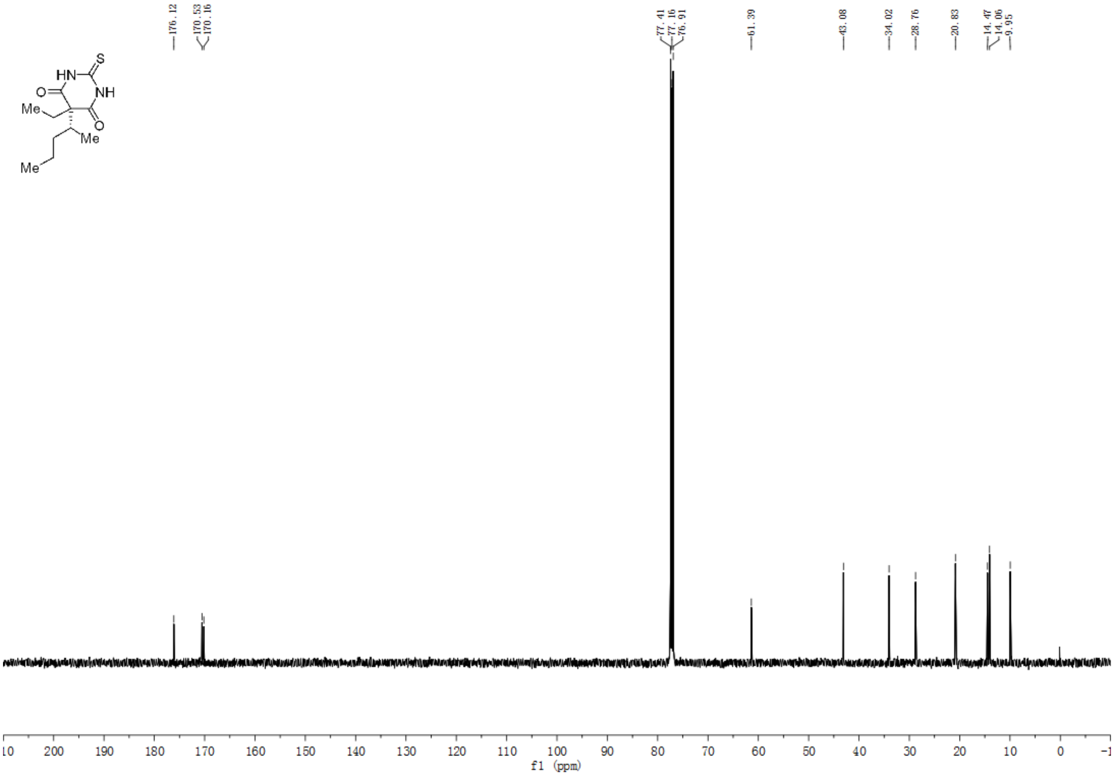

^{1}H NMR spectrum of **10**

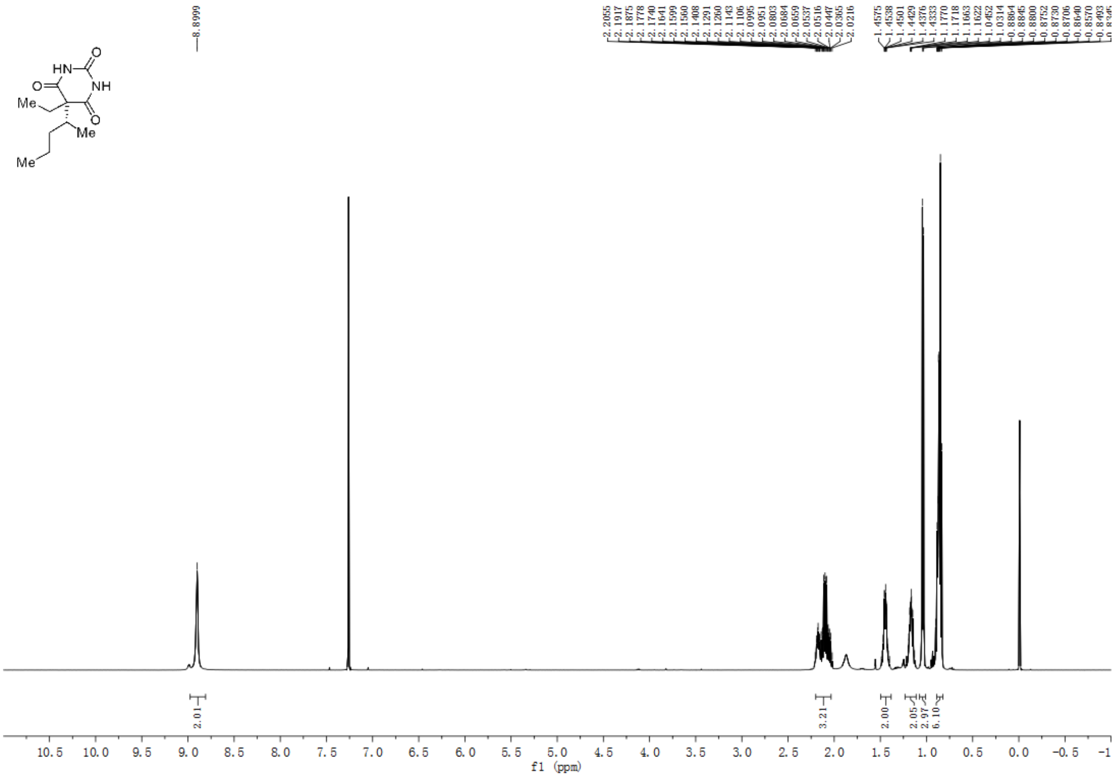

^{13}C NMR spectrum of **10**

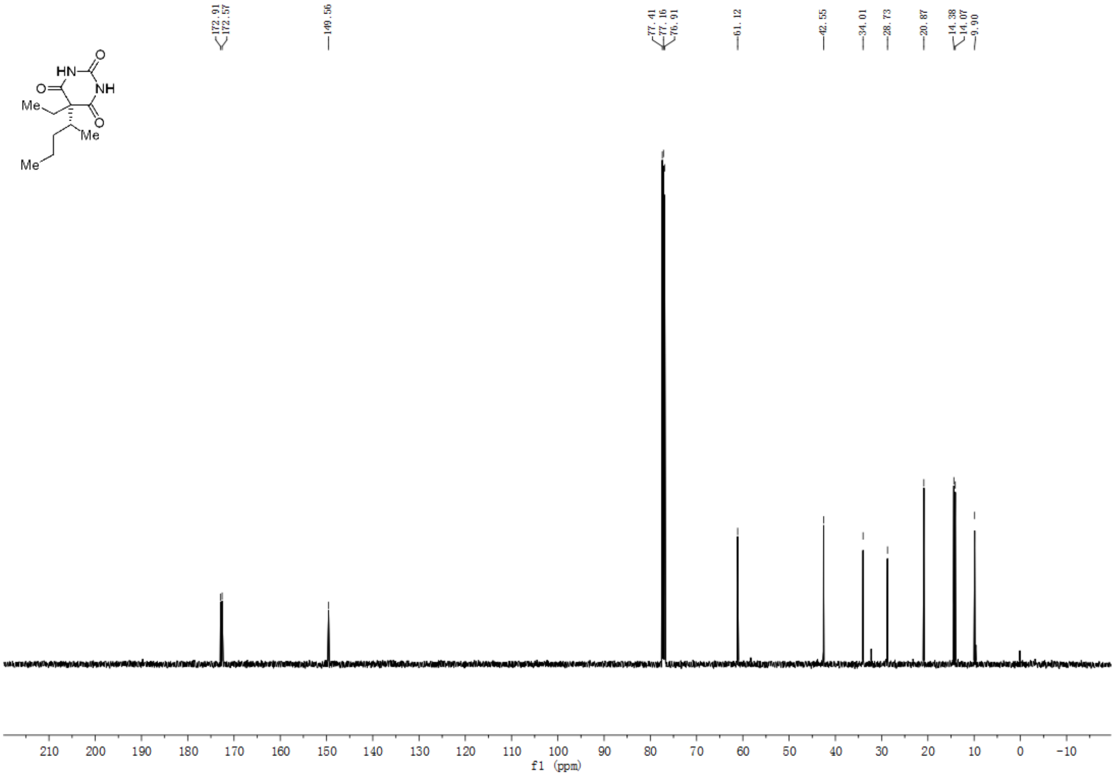

^{1}H NMR spectrum of **11**

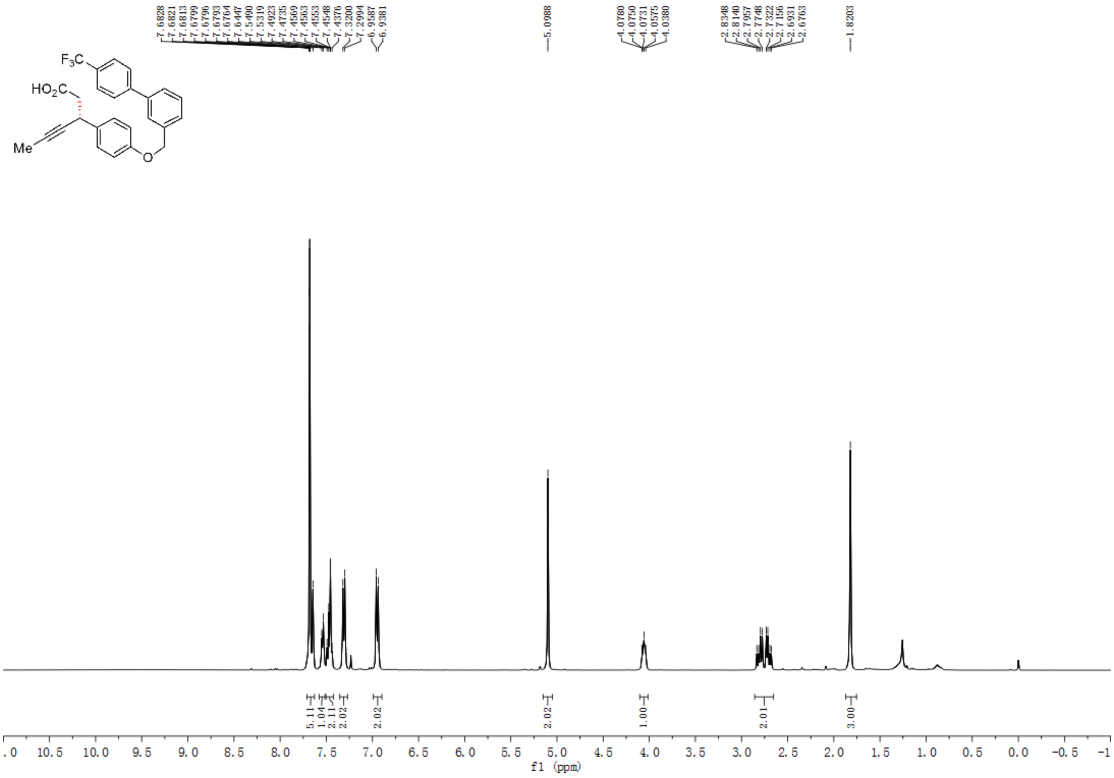

^{13}C NMR spectrum of **11**

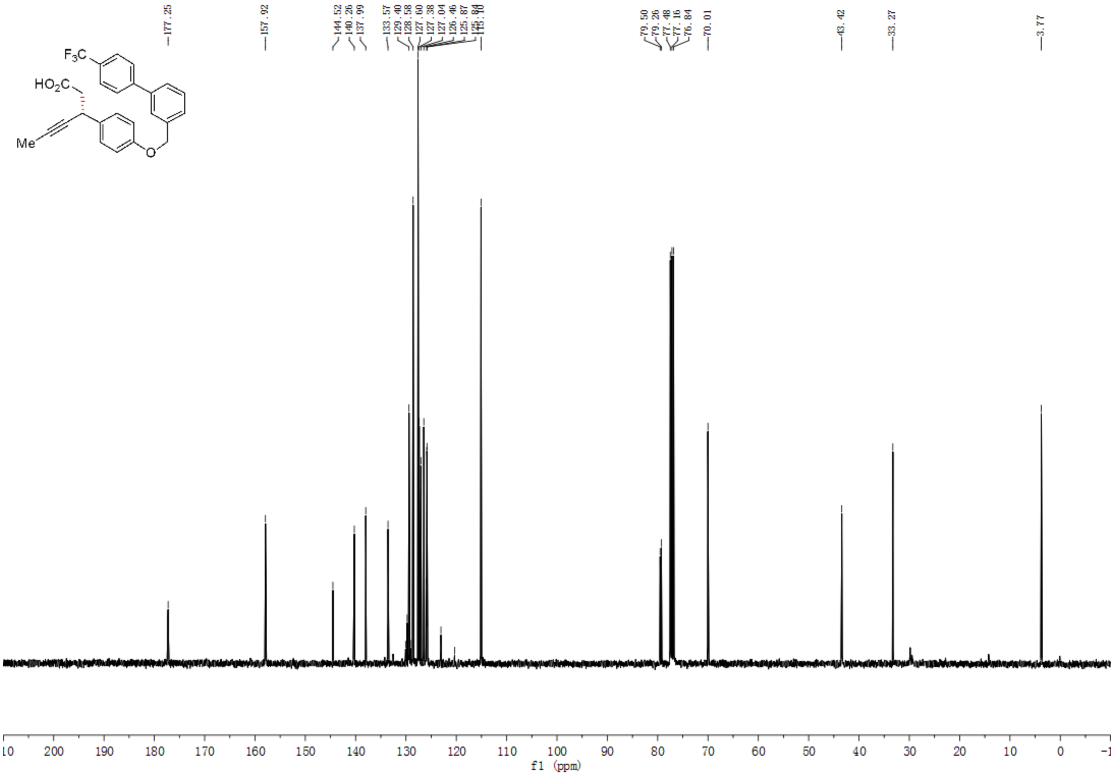

^{19}F NMR spectrum of **11**

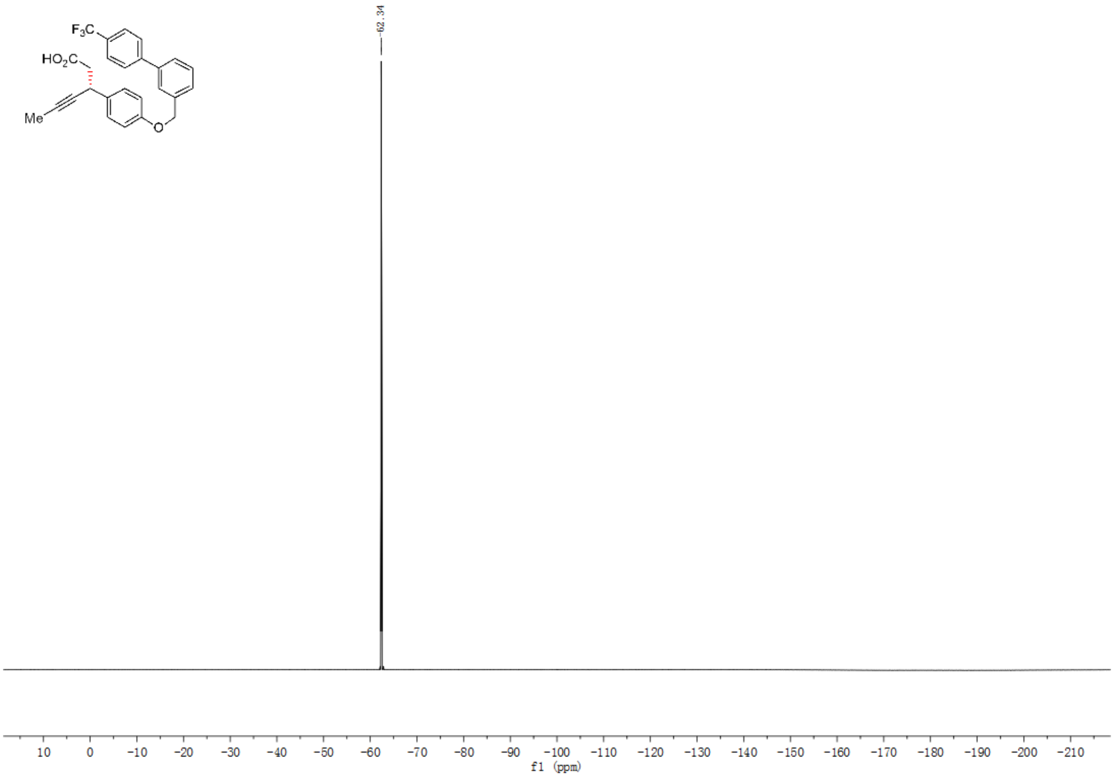

^{1}H NMR spectrum of **12**

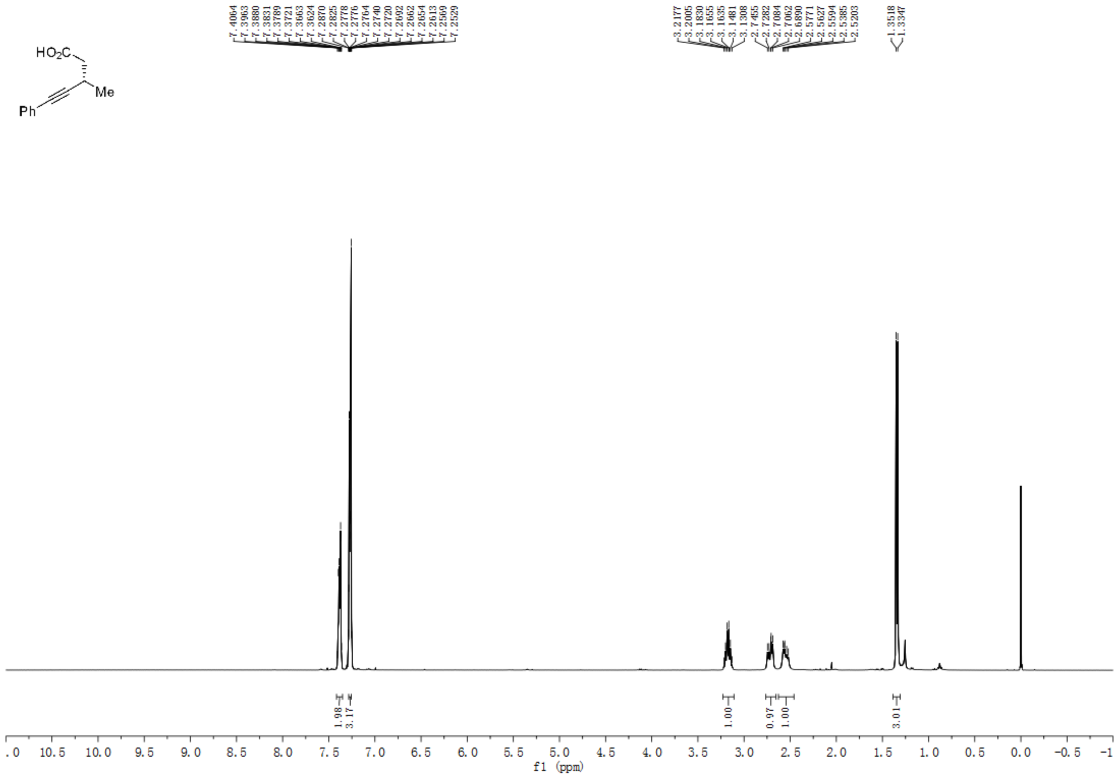

^{13}C NMR spectrum of **12**

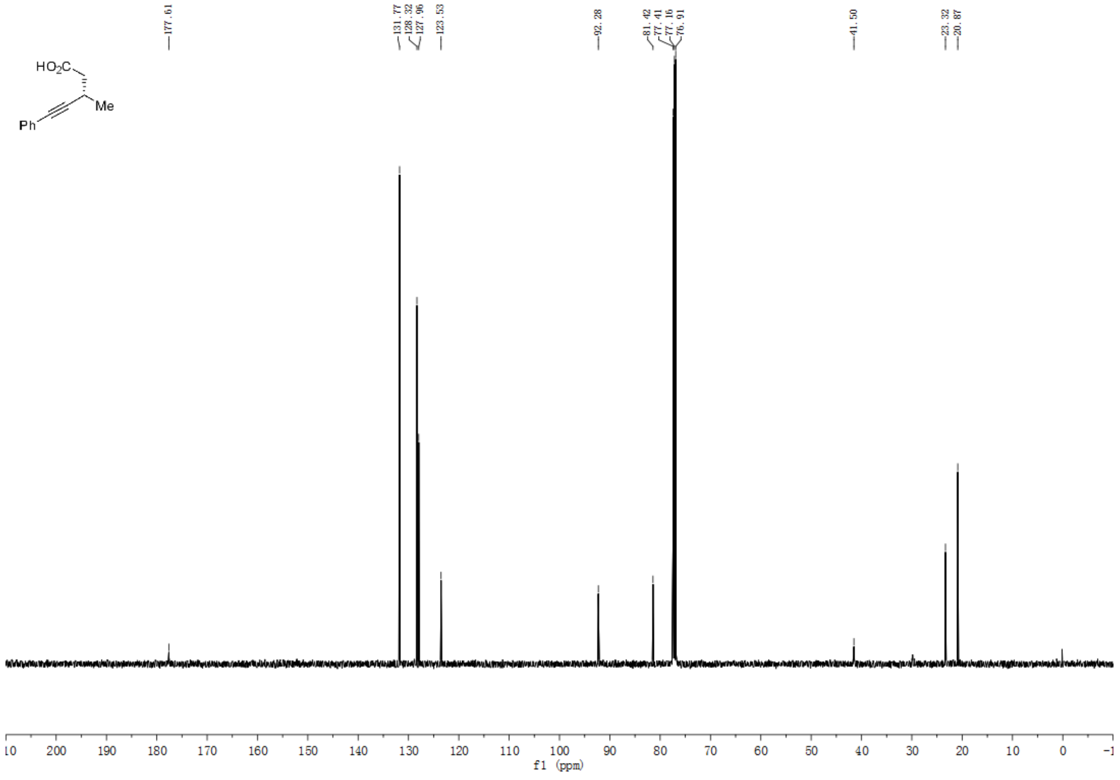

^{1}H NMR spectrum of **13**

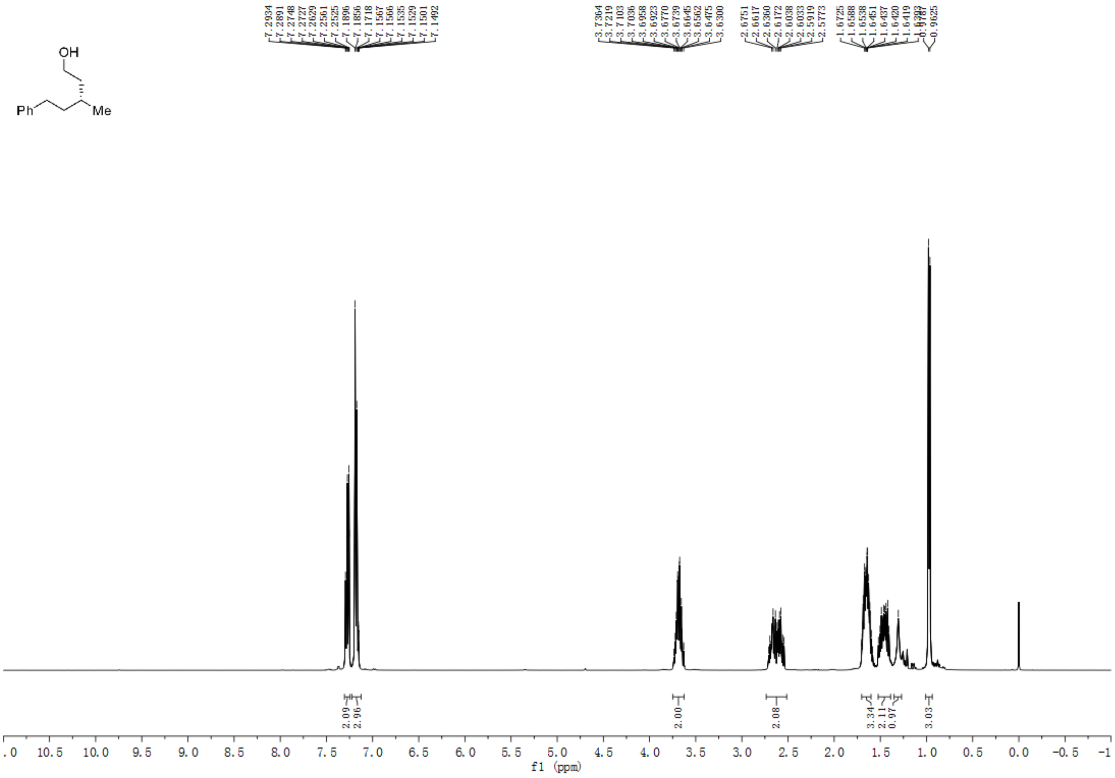

^{13}C NMR spectrum of **13**

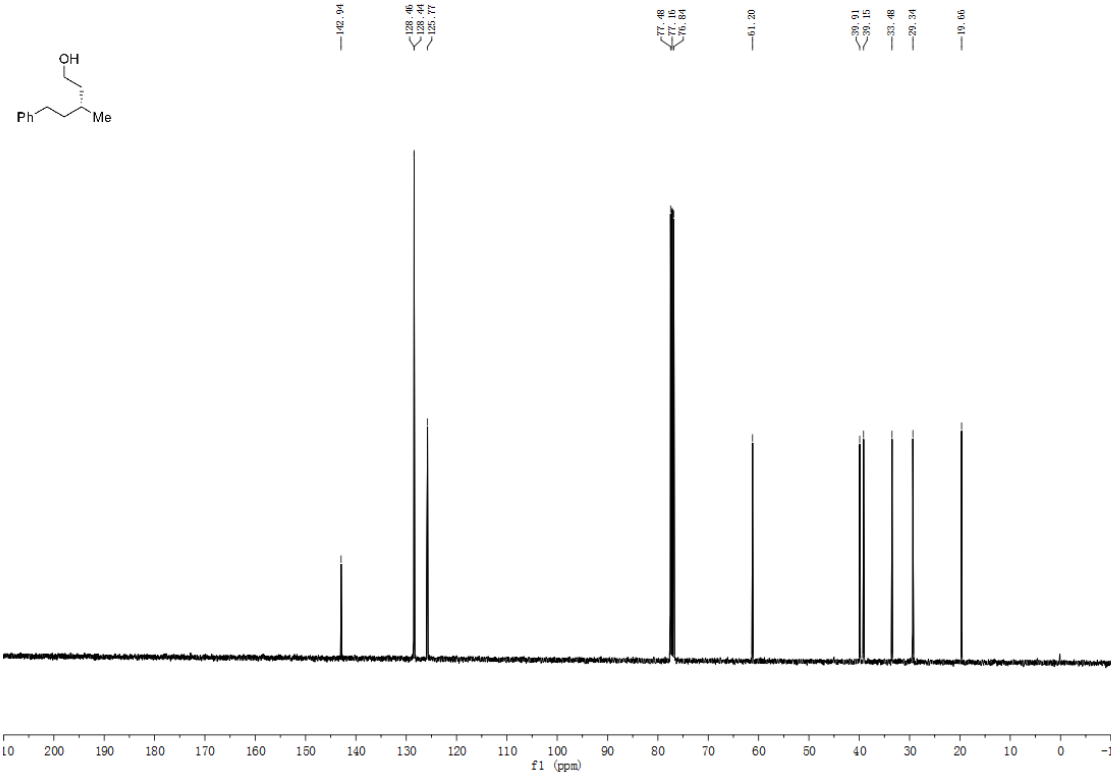

^{1}H NMR spectrum of **14**

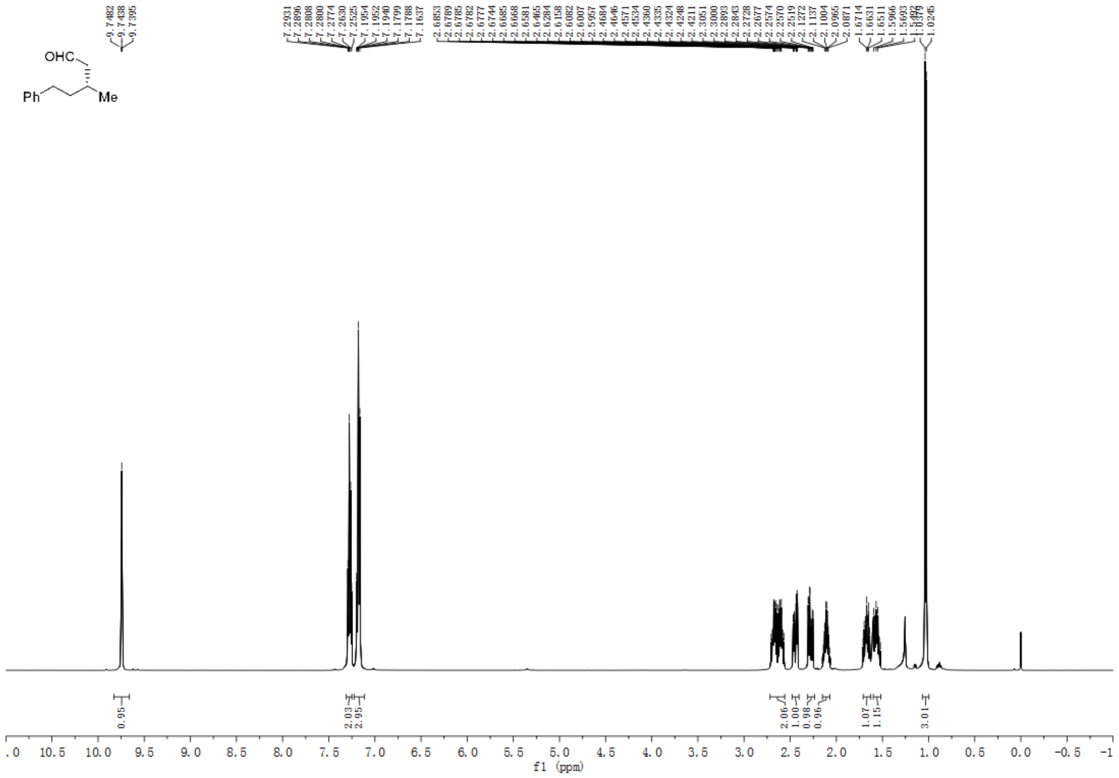

^{13}C NMR spectrum of **14**

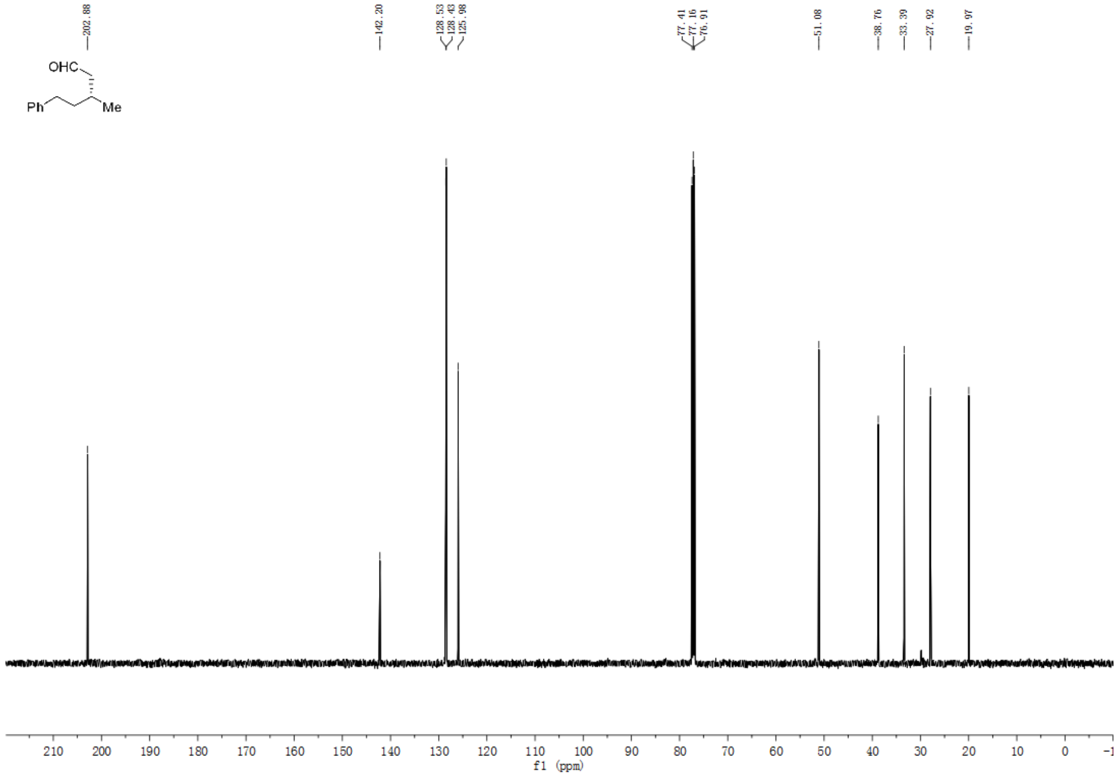

^{1}H NMR spectrum of **15**

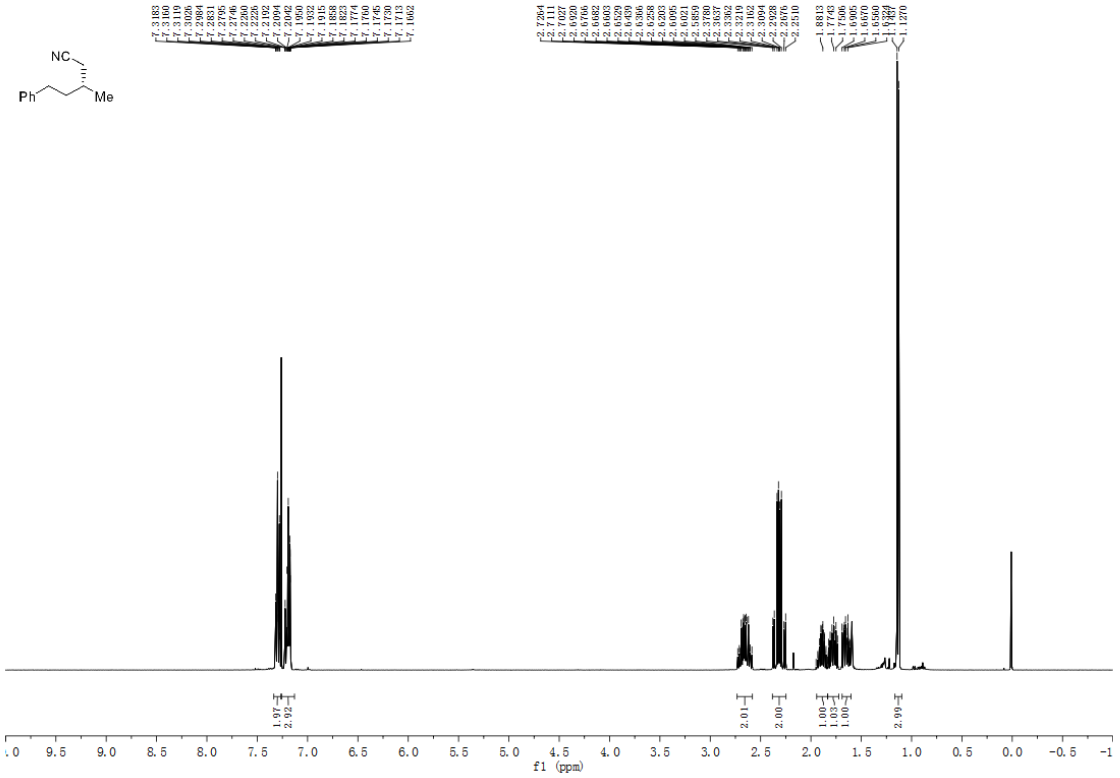

^{13}C NMR spectrum of **15**

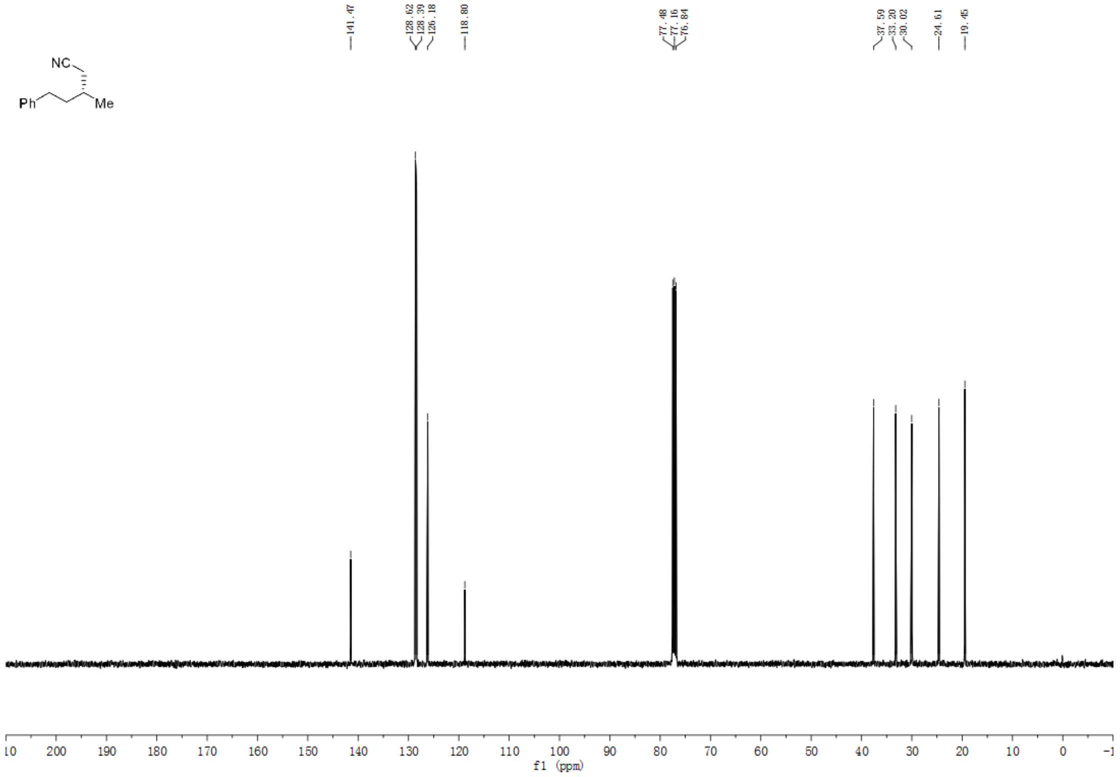

12. HPLC traces

Rac-**3a**

SAMPLE INFORMATION

Sample Name:	cxh-7-25-3-IC-2%-rac	Acquired By:	System
Sample Type:	Unknown	Sample Set Name:	
Vial:	24	Acq. Method Set:	2%
Injection #:	1	Processing Method	bgfb
Injection Volume:	10.00 ul	Channel Name:	211.0nm
Run Time:	30.0 Minutes	Proc. Chnl. Descr.:	2998 PDA 211.0 nm (2998
Date Acquired:	5/7/2020 11:33:02 AM CST		
Date Processed:	8/21/2020 9:05:38 PM CST		

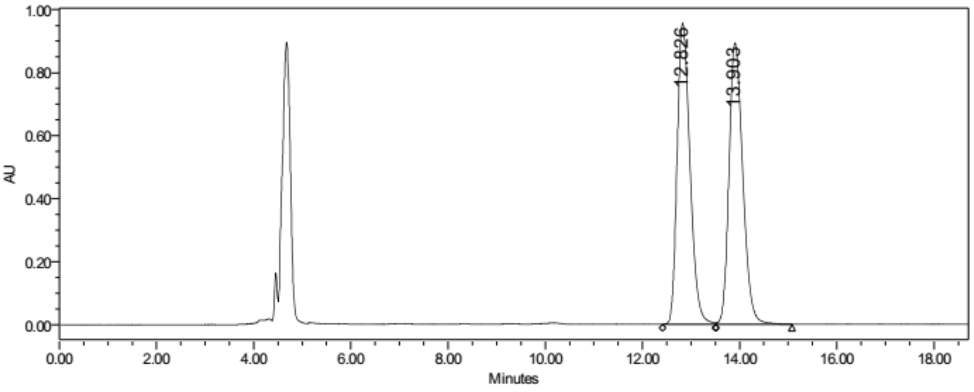

	RT	Area	% Area	Height
1	12.826	17475178	49.81	956128
2	13.903	17605247	50.19	893532

Asy-**3a**

SAMPLE INFORMATION

Sample Name:	cxh-7-51-1-IC-2%	Acquired By:	System
Sample Type:	Unknown	Sample Set Name:	0511
Vial:	7	Acq. Method Set:	2%
Injection #:	1	Processing Method	3a
Injection Volume:	10.00 ul	Channel Name:	211.0nm
Run Time:	18.0 Minutes	Proc. Chnl. Descr.:	2998 PDA 211.0 nm (2998
Date Acquired:	5/11/2020 4:27:34 PM CST		
Date Processed:	8/21/2020 9:07:30 PM CST		

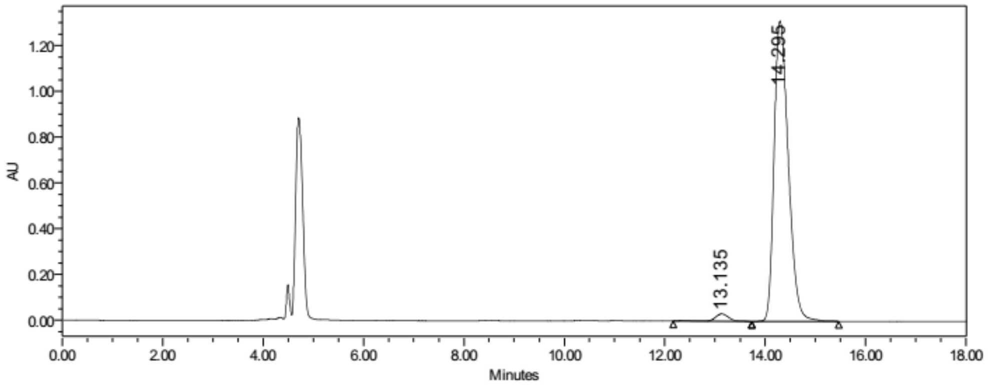

	RT	Area	% Area	Height
1	13.135	629394	2.41	32385
2	14.295	25484981	97.59	1312752

Rac-**3b**

SAMPLE INFORMATION

Sample Name:	cxh-7-42-1--RAC-IC-1%	Acquired By:	System
Sample Type:	Unknown	Sample Set Name:	
Vial:	5	Acq. Method Set:	1%
Injection #:	1	Processing Method	dcvs
Injection Volume:	10.00 ul	Channel Name:	211.0nm
Run Time:	40.0 Minutes	Proc. Chnl. Descr.:	2998 PDA 211.0 nm (2998
Date Acquired:	6/4/2020 4:55:57 PM CST		
Date Processed:	8/21/2020 9:10:40 PM CST		

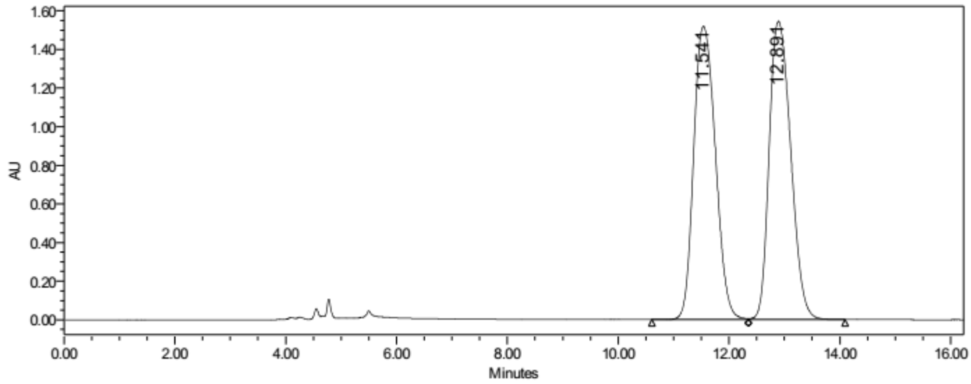

	RT	Area	% Area	Height
1	11.541	41392626	49.98	1519231
2	12.891	41429655	50.02	1544944

Asy-**3b**

SAMPLE INFORMATION

Sample Name:	cxh-7-71-1-IC-1%	Acquired By:	System
Sample Type:	Unknown	Sample Set Name:	
Vial:	120	Acq. Method Set:	1%
Injection #:	1	Processing Method	ssda
Injection Volume:	10.00 ul	Channel Name:	211.0nm
Run Time:	18.0 Minutes	Proc. Chnl. Descr.:	2998 PDA 211.0 nm (2998
Date Acquired:	6/4/2020 5:17:17 PM CST		
Date Processed:	8/21/2020 9:11:35 PM CST		

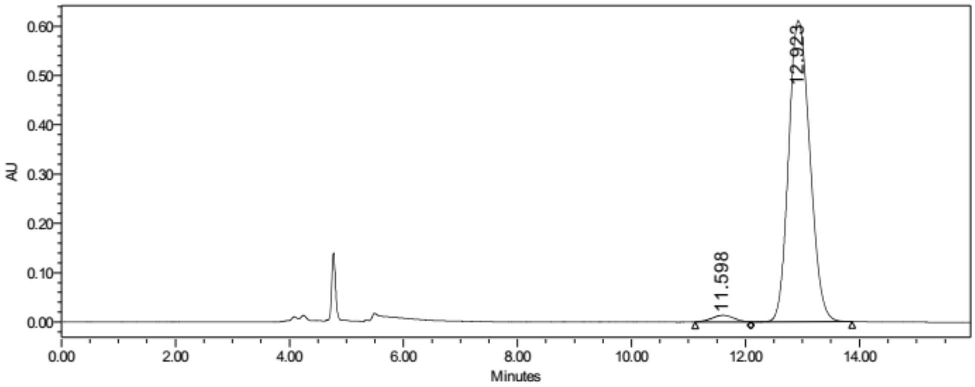

	RT	Area	% Area	Height
1	11.598	354463	2.19	13233
2	12.923	15847839	97.81	610667

Rac-**3c**

SAMPLE INFORMATION

Sample Name:	cxh-7-41-1-RAC-AD-1%	Acquired By:	System
Sample Type:	Unknown	Sample Set Name:	
Vial:	83	Acq. Method Set:	1%
Injection #:	3	Processing Method	fhd
Injection Volume:	10.00 ul	Channel Name:	211.0nm
Run Time:	50.0 Minutes	Proc. Chnl. Descr.:	2998 PDA 211.0 nm (2998
Date Acquired:	6/4/2020 8:07:49 PM CST		
Date Processed:	8/21/2020 9:13:28 PM CST		

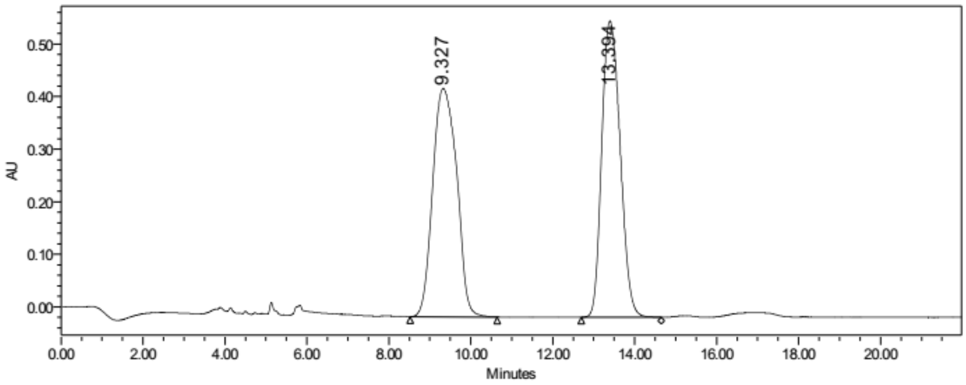

	RT	Area	% Area	Height
1	9.327	17569773	50.11	434487
2	13.394	17494907	49.89	563323

Asy-**3c**

SAMPLE INFORMATION

Sample Name:	cxh-7-71-3-AD-1%	Acquired By:	System
Sample Type:	Unknown	Sample Set Name:	
Vial:	28	Acq. Method Set:	1%
Injection #:	2	Processing Method	fdsgv
Injection Volume:	10.00 ul	Channel Name:	211.0nm
Run Time:	20.0 Minutes	Proc. Chnl. Descr.:	2998 PDA 211.0 nm (2998
Date Acquired:	6/4/2020 8:52:23 PM CST		
Date Processed:	8/21/2020 9:14:52 PM CST		

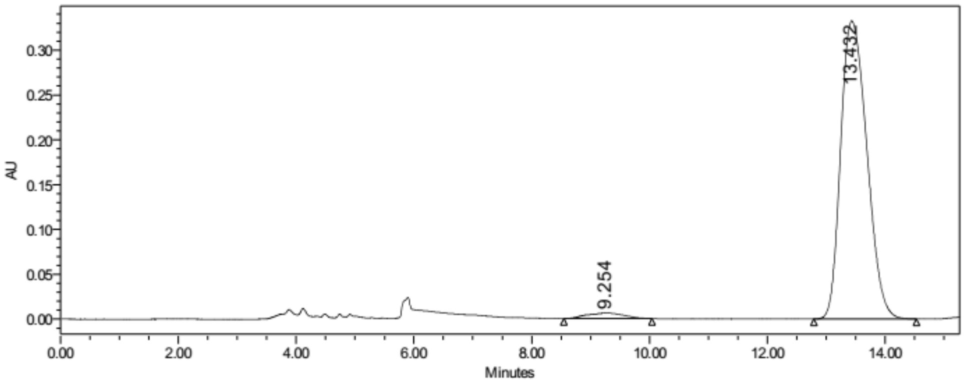

	RT	Area	% Area	Height
1	9.254	268634	2.54	6092
2	13.432	10317092	97.46	332407

Rac-**3d**

SAMPLE INFORMATION

Sample Name:	CXH-7-35-6-rac-ic-1%	Acquired By:	System
Sample Type:	Unknown	Sample Set Name:	
Vial:	101	Acq. Method Set:	1%
Injection #:	2	Processing Method	3d
Injection Volume:	10.00 ul	Channel Name:	211.0nm
Run Time:	50.0 Minutes	Proc. Chnl. Descr.:	2998 PDA 211.0 nm (2998
Date Acquired:	6/22/2020 7:34:28 PM CST		
Date Processed:	8/21/2020 9:18:17 PM CST		

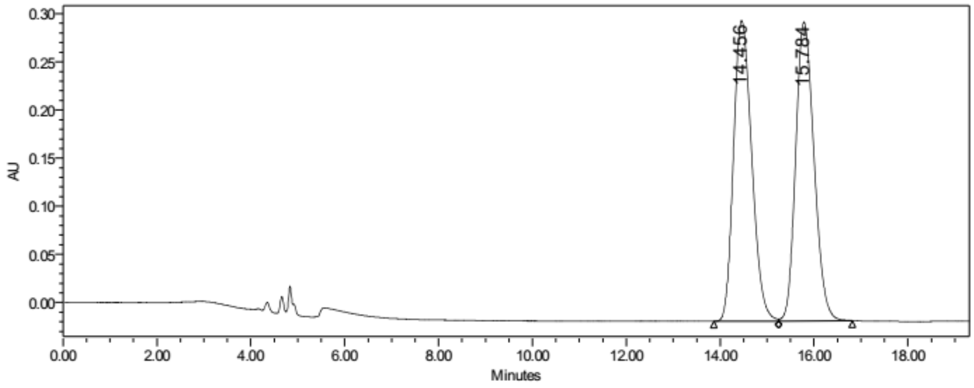

	RT	Area	% Area	Height
1	14.456	8464266	49.93	312349
2	15.784	8489117	50.07	310588

Asy-**3d**

SAMPLE INFORMATION

Sample Name:	CXH-7-73-3b-ic-1%	Acquired By:	System
Sample Type:	Unknown	Sample Set Name:	
Vial:	66	Acq. Method Set:	1%
Injection #:	1	Processing Method	3d2
Injection Volume:	10.00 ul	Channel Name:	211.0nm
Run Time:	50.0 Minutes	Proc. Chnl. Descr.:	2998 PDA 211.0 nm (2998
Date Acquired:	6/22/2020 7:54:49 PM CST		
Date Processed:	8/21/2020 9:19:20 PM CST		

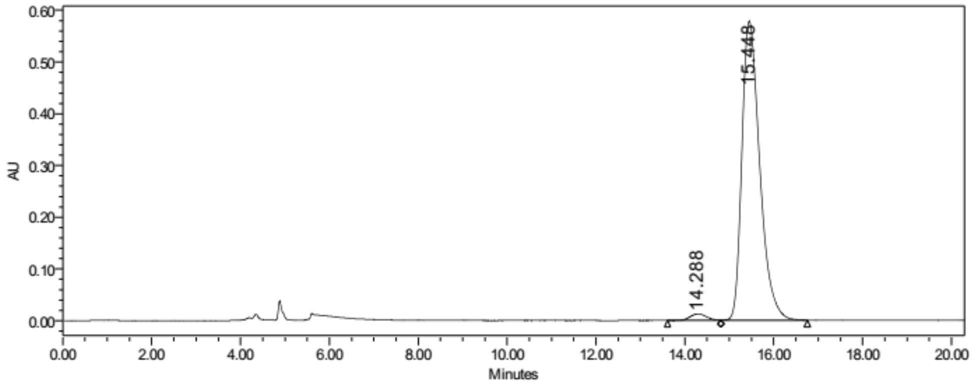

	RT	Area	% Area	Height
1	14.288	322777	1.95	12655
2	15.448	16244308	98.05	578480

Rac-**3e**

SAMPLE INFORMATION

Sample Name:	CXH-7-34-7--rac-AD-5%	Acquired By:	System
Sample Type:	Unknown	Sample Set Name:	
Vial:	64	Acq. Method Set:	5%
Injection #:	1	Processing Method	3e
Injection Volume:	10.00 ul	Channel Name:	222.0nm
Run Time:	50.0 Minutes	Proc. Chnl. Descr.:	2998 PDA 222.0 nm (2998
Date Acquired:	6/22/2020 3:21:01 PM CST		
Date Processed:	8/21/2020 9:22:40 PM CST		

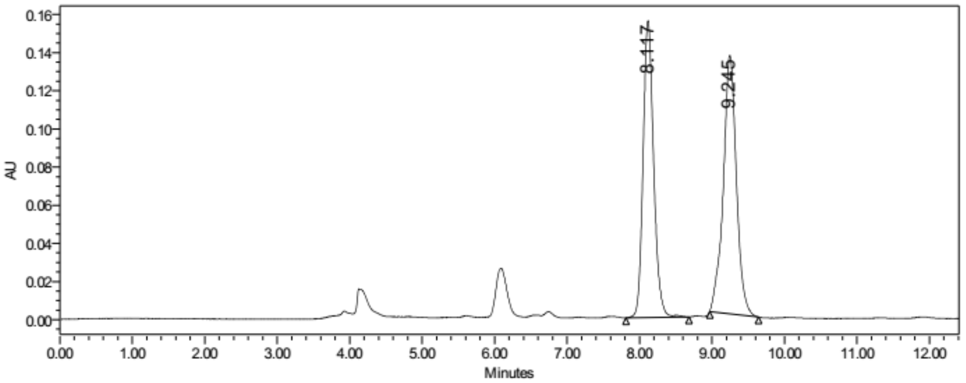

	RT	Area	% Area	Height
1	8.117	1583943	48.14	155599
2	9.245	1706621	51.86	135502

Asy-**3e**

SAMPLE INFORMATION

Sample Name:	CXH-7-73-1-AD-5%	Acquired By:	System
Sample Type:	Unknown	Sample Set Name:	
Vial:	91	Acq. Method Set:	5%
Injection #:	1	Processing Method	3e2
Injection Volume:	10.00 ul	Channel Name:	222.0nm
Run Time:	50.0 Minutes	Proc. Chnl. Descr.:	2998 PDA 222.0 nm (2998
Date Acquired:	6/22/2020 3:56:40 PM CST		
Date Processed:	8/21/2020 9:23:35 PM CST		

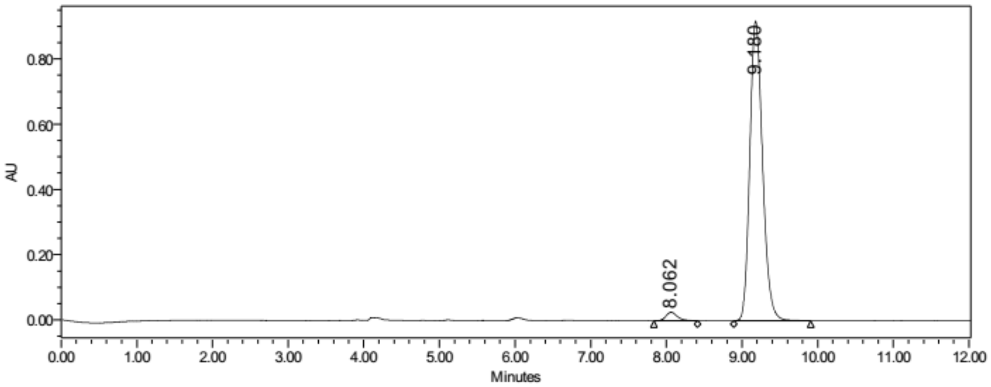

	RT	Area	% Area	Height
1	8.062	277515	2.50	26144
2	9.180	10813420	97.50	918699

Rac-**3f**

SAMPLE INFORMATION

Sample Name:	cxh-7-41-5-RAC-OD-3%	Acquired By:	System
Sample Type:	Unknown	Sample Set Name:	
Vial:	10	Acq. Method Set:	3%
Injection #:	1	Processing Method	3f
Injection Volume:	10.00 ul	Channel Name:	211.0nm
Run Time:	80.0 Minutes	Proc. Chnl. Descr.:	2998 PDA 211.0 nm (2998
Date Acquired:	5/29/2020 7:04:37 PM CST		
Date Processed:	8/21/2020 9:28:13 PM CST		

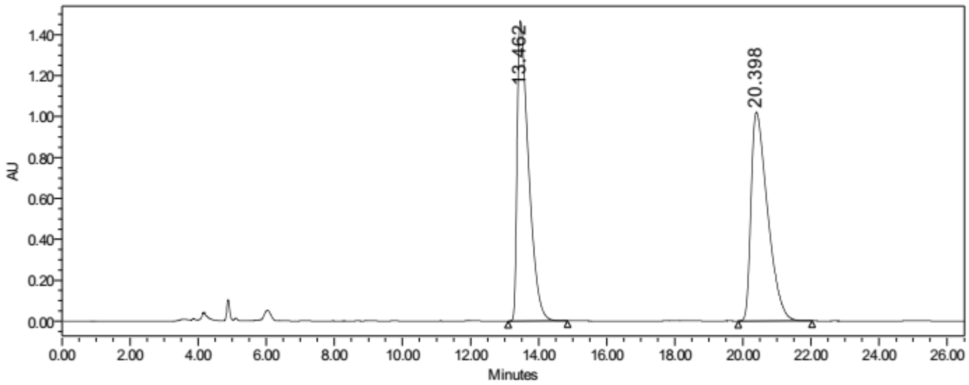

	RT	Area	% Area	Height
1	13.462	34410482	49.82	1466352
2	20.398	34655408	50.18	1021475

Asy-**3f**

SAMPLE INFORMATION

Sample Name:	cxh-7-66-5-OD-3%	Acquired By:	System
Sample Type:	Unknown	Sample Set Name:	
Vial:	34	Acq. Method Set:	3%
Injection #:	2	Processing Method	3f1
Injection Volume:	10.00 ul	Channel Name:	211.0nm
Run Time:	80.0 Minutes	Proc. Chnl. Descr.:	2998 PDA 211.0 nm (2998
Date Acquired:	5/29/2020 8:16:08 PM CST		
Date Processed:	8/21/2020 9:29:22 PM CST		

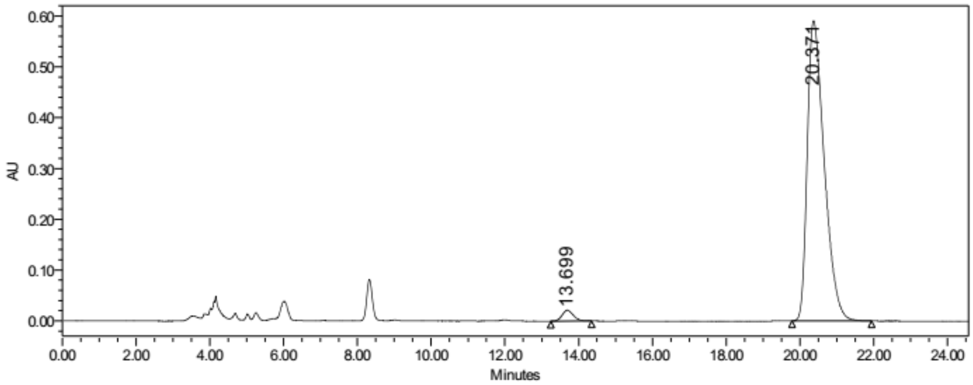

	RT	Area	% Area	Height
1	13.699	457493	2.35	21389
2	20.371	18972148	97.65	591114

Rac-**3g**

SAMPLE INFORMATION			
Sample Name:	cxh-7-41-3-RAC-OD-3%	Acquired By:	System
Sample Type:	Unknown	Sample Set Name:	
Vial:	73	Acq. Method Set:	3%
Injection #:	3	Processing Method	3g
Injection Volume:	10.00 ul	Channel Name:	211.0nm
Run Time:	80.0 Minutes	Proc. Chnl. Descr.:	2998 PDA 211.0 nm (2998
Date Acquired:	5/29/2020 5:34:08 PM CST		
Date Processed:	8/21/2020 9:31:16 PM CST		

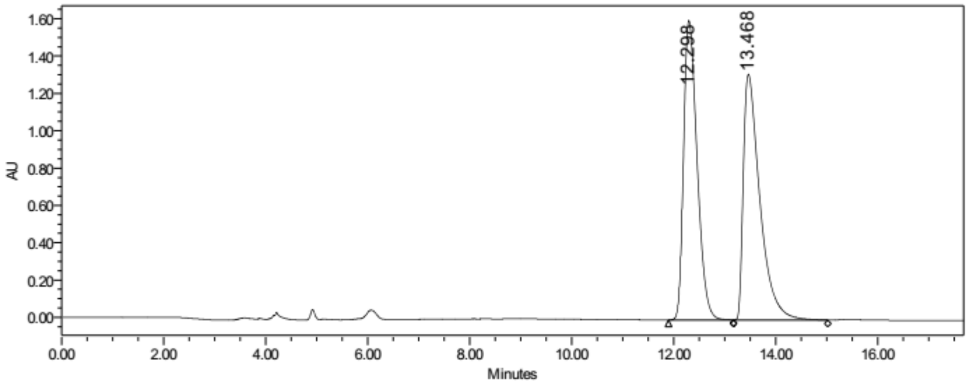

	RT	Area	% Area	Height
1	12.298	29585234	49.70	1602831
2	13.468	29937977	50.30	1316510

Asy-**3g**

SAMPLE INFORMATION			
Sample Name:	cxh-7-66-4-OD-3%	Acquired By:	System
Sample Type:	Unknown	Sample Set Name:	
Vial:	104	Acq. Method Set:	3%
Injection #:	2	Processing Method	3g2
Injection Volume:	10.00 ul	Channel Name:	211.0nm
Run Time:	80.0 Minutes	Proc. Chnl. Descr.:	2998 PDA 211.0 nm (2998
Date Acquired:	5/29/2020 6:42:45 PM CST		
Date Processed:	8/21/2020 9:33:12 PM CST		

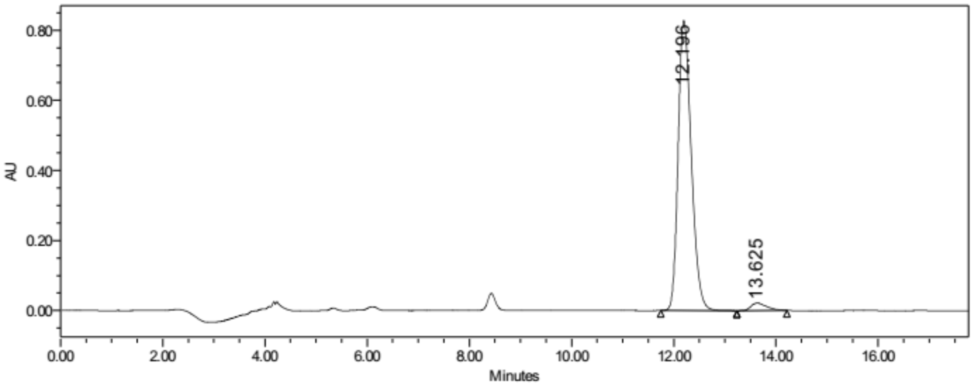

	RT	Area	% Area	Height
1	12.196	14727723	97.00	827816
2	13.625	455208	3.00	21181

Rac-**3h**

SAMPLE INFORMATION

Sample Name:	cxh-7-28-4-rac-IC-1%	Acquired By:	System
Sample Type:	Unknown	Sample Set Name:	
Vial:	37	Acq. Method Set:	1%
Injection #:	1	Processing Method	3k
Injection Volume:	10.00 ul	Channel Name:	211.0nm
Run Time:	60.0 Minutes	Proc. Chnl. Descr.:	2998 PDA 211.0 nm (2998
Date Acquired:	6/2/2020 8:51:21 PM CST		
Date Processed:	8/21/2020 9:46:23 PM CST		

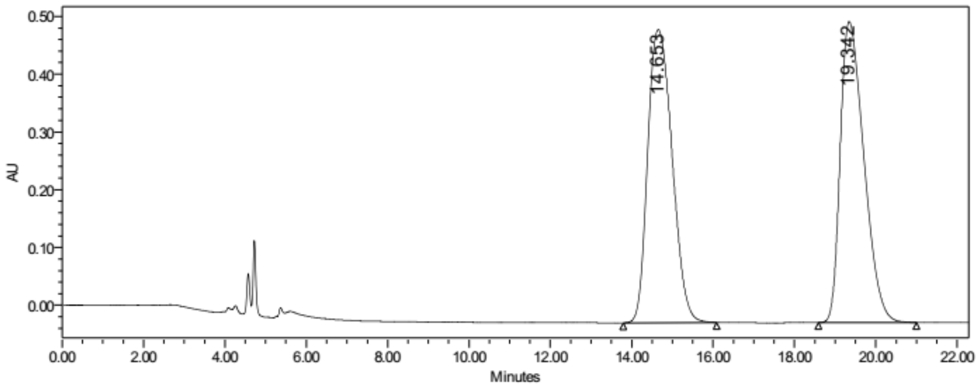

	RT	Area	% Area	Height
1	14.653	21125588	50.01	507704
2	19.342	21119470	49.99	520919

Asy-**3h**

SAMPLE INFORMATION

Sample Name:	cxh-7-69-6-rac-IC-1%	Acquired By:	System
Sample Type:	Unknown	Sample Set Name:	
Vial:	67	Acq. Method Set:	1%
Injection #:	1	Processing Method	3k2
Injection Volume:	10.00 ul	Channel Name:	211.0nm
Run Time:	30.0 Minutes	Proc. Chnl. Descr.:	2998 PDA 211.0 nm (2998
Date Acquired:	6/2/2020 9:14:40 PM CST		
Date Processed:	8/21/2020 9:47:09 PM CST		

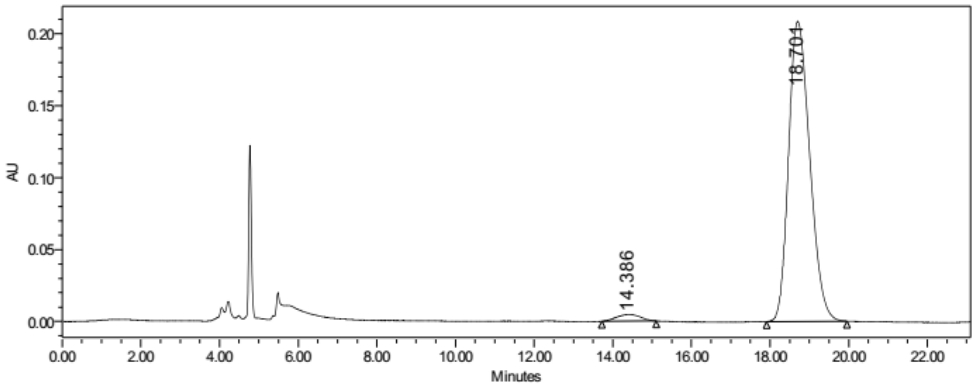

	RT	Area	% Area	Height
1	14.386	197275	2.46	4606
2	18.701	7825435	97.54	208658

Rac-**3i**

SAMPLE INFORMATION

Sample Name:	cxh-7-95-1-rac-AD-20%	Acquired By:	System
Sample Type:	Unknown	Sample Set Name:	
Vial:	57	Acq. Method Set:	20%
Injection #:	3	Processing Method	3I
Injection Volume:	10.00 ul	Channel Name:	211.0nm
Run Time:	40.0 Minutes	Proc. Chnl. Descr.:	2998 PDA 211.0 nm (2998
Date Acquired:	8/4/2020 2:25:20 PM CST		
Date Processed:	8/21/2020 9:48:24 PM CST		

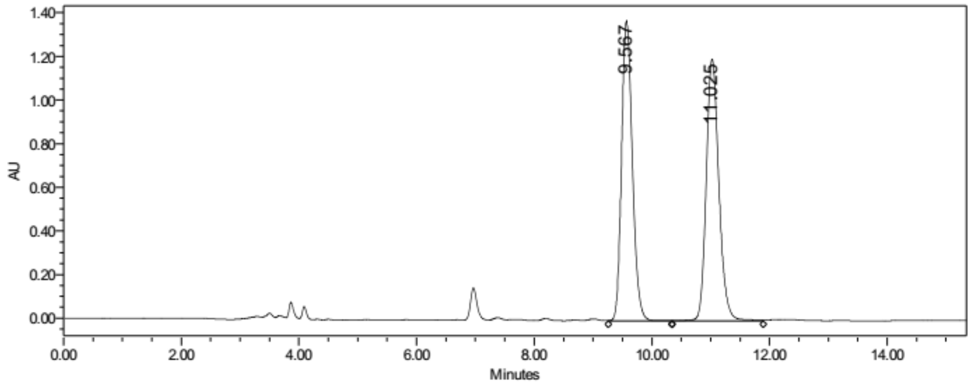

	RT	Area	% Area	Height
1	9.567	17403635	49.27	1375303
2	11.025	17921655	50.73	1200753

Asy-**3i**

SAMPLE INFORMATION

Sample Name:	cxh-7-95-2-AD-20%	Acquired By:	System
Sample Type:	Unknown	Sample Set Name:	
Vial:	85	Acq. Method Set:	20%
Injection #:	2	Processing Method	3I2
Injection Volume:	10.00 ul	Channel Name:	211.0nm
Run Time:	15.0 Minutes	Proc. Chnl. Descr.:	2998 PDA 211.0 nm (2998
Date Acquired:	8/5/2020 11:39:18 AM CST		
Date Processed:	8/21/2020 9:49:13 PM CST		

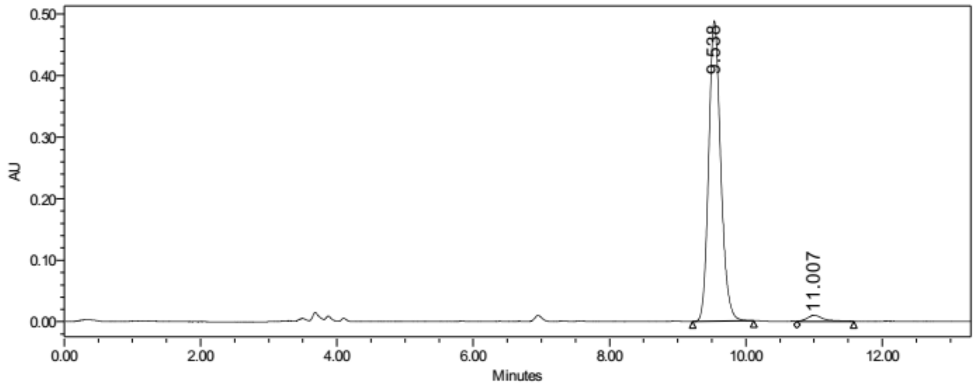

	RT	Area	% Area	Height
1	9.538	6021488	97.41	488394
2	11.007	160086	2.59	9780

Rac-**3j**

SAMPLE INFORMATION

Sample Name:	cxh-7-28-2-rac-IC-5%	Acquired By:	System
Sample Type:	Unknown	Sample Set Name:	
Vial:	10	Acq. Method Set:	5%
Injection #:	1	Processing Method	3i
Injection Volume:	10.00 ul	Channel Name:	211.0nm
Run Time:	30.0 Minutes	Proc. Chnl. Descr.:	2998 PDA 211.0 nm (2998
Date Acquired:	6/3/2020 8:53:49 AM CST		
Date Processed:	8/21/2020 9:41:01 PM CST		

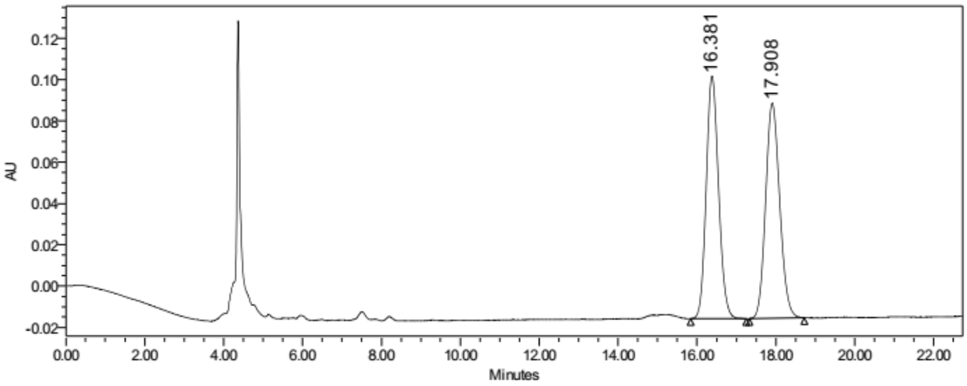

	RT	Area	% Area	Height
1	16.381	2560339	49.70	117689
2	17.908	2591020	50.30	104242

Asy-**3j**

SAMPLE INFORMATION

Sample Name:	cxh-7-70-1-IC-5%	Acquired By:	System
Sample Type:	Unknown	Sample Set Name:	
Vial:	27	Acq. Method Set:	5%
Injection #:	1	Processing Method	3i2
Injection Volume:	10.00 ul	Channel Name:	211.0nm
Run Time:	30.0 Minutes	Proc. Chnl. Descr.:	2998 PDA 211.0 nm (2998
Date Acquired:	6/3/2020 9:24:32 AM CST		
Date Processed:	8/21/2020 9:41:49 PM CST		

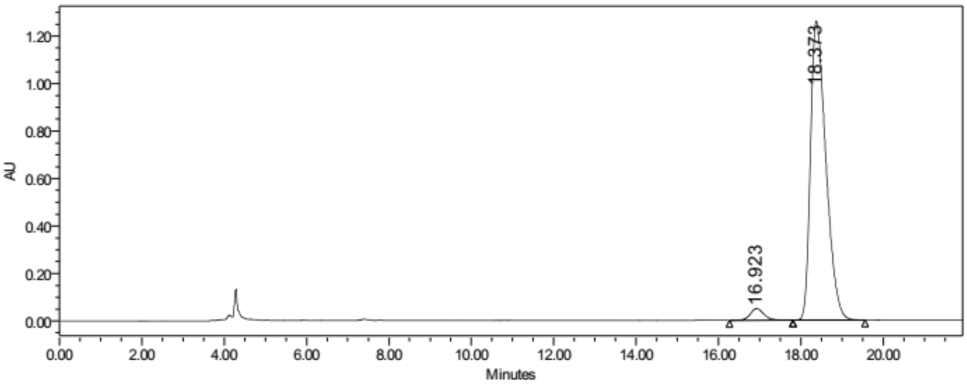

	RT	Area	% Area	Height
1	16.923	1133614	3.35	48765
2	18.373	32665753	96.65	1259739

Rac-**3k**

SAMPLE INFORMATION

Sample Name:	cxh-7-13-1-rac-IC-3%	Acquired By:	System
Sample Type:	Unknown	Sample Set Name:	
Vial:	6	Acq. Method Set:	3%
Injection #:	1	Processing Method	3h
Injection Volume:	10.00 ul	Channel Name:	211.0nm
Run Time:	80.0 Minutes	Proc. Chnl. Descr.:	2998 PDA 211.0 nm (2998
Date Acquired:	6/2/2020 6:25:32 PM CST		
Date Processed:	8/21/2020 9:38:48 PM CST		

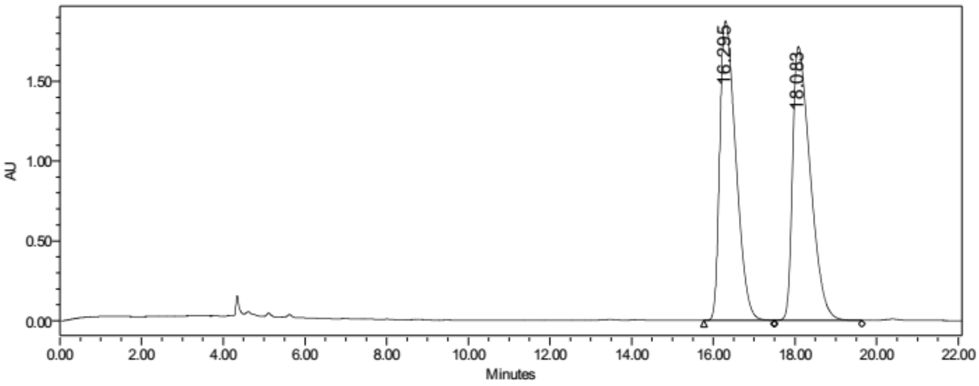

	RT	Area	% Area	Height
1	16.295	51120384	49.58	1874790
2	18.083	51994896	50.42	1715997

Asy-**3k**

SAMPLE INFORMATION

Sample Name:	cxh-7-69-1-IC-3%	Acquired By:	System
Sample Type:	Unknown	Sample Set Name:	
Vial:	26	Acq. Method Set:	3%
Injection #:	1	Processing Method	3h2
Injection Volume:	10.00 ul	Channel Name:	211.0nm
Run Time:	24.0 Minutes	Proc. Chnl. Descr.:	2998 PDA 211.0 nm (2998
Date Acquired:	6/2/2020 6:48:37 PM CST		
Date Processed:	8/21/2020 9:39:28 PM CST		

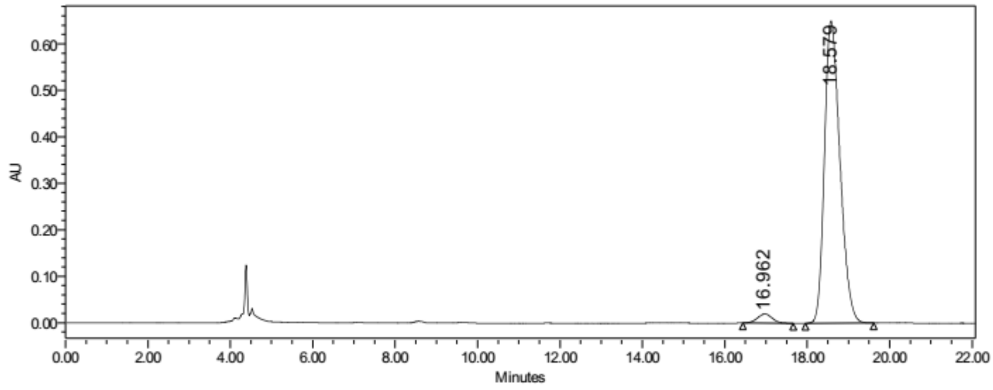

	RT	Area	% Area	Height
1	16.962	489688	2.81	19777
2	18.579	16932238	97.19	650066

Rac-**3l**

SAMPLE INFORMATION

Sample Name:	cxh-7-34-6-rac-AD-20%	Acquired By:	System
Sample Type:	Unknown	Sample Set Name:	0610
Vial:	20	Acq. Method Set:	20%
Injection #:	1	Processing Method	3j
Injection Volume:	10.00 ul	Channel Name:	211.0nm
Run Time:	20.0 Minutes	Proc. Chnl. Descr.:	2998 PDA 211.0 nm (2998
Date Acquired:	6/10/2020 11:57:41 AM CST		
Date Processed:	8/21/2020 9:43:04 PM CST		

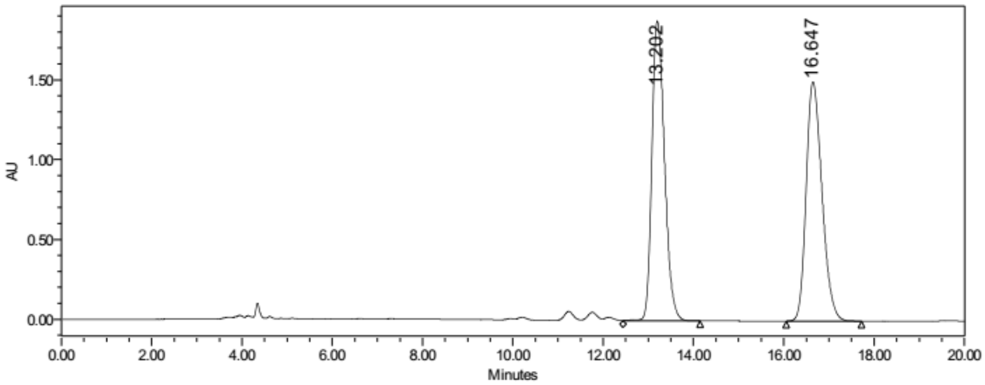

	RT	Area	% Area	Height
1	13.202	36353733	49.85	1878192
2	16.647	36567378	50.15	1498286

Asy-**3l**

SAMPLE INFORMATION

Sample Name:	cxh-7-72-4-AD-20%	Acquired By:	System
Sample Type:	Unknown	Sample Set Name:	0610
Vial:	33	Acq. Method Set:	20%
Injection #:	1	Processing Method	3j2
Injection Volume:	10.00 ul	Channel Name:	211.0nm
Run Time:	20.0 Minutes	Proc. Chnl. Descr.:	2998 PDA 211.0 nm (2998
Date Acquired:	6/10/2020 12:23:45 PM CST		
Date Processed:	8/21/2020 9:44:37 PM CST		

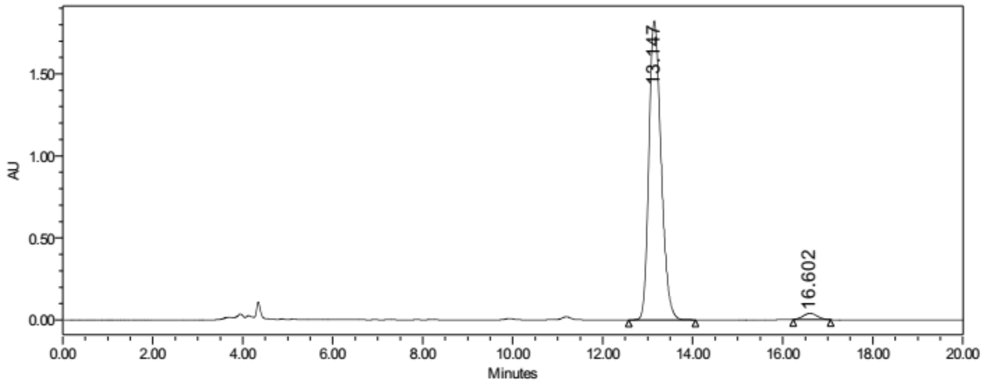

	RT	Area	% Area	Height
1	13.147	34256160	97.60	1822698
2	16.602	843099	2.40	38141

Rac-**3m**

SAMPLE INFORMATION

Sample Name:	cxh-7-34-3-rac-IC-3%	Acquired By:	System
Sample Type:	Unknown	Sample Set Name:	
Vial:	105	Acq. Method Set:	3%
Injection #:	1	Processing Method	3m
Injection Volume:	10.00 ul	Channel Name:	211.0nm
Run Time:	24.0 Minutes	Proc. Chnl. Descr.:	2998 PDA 211.0 nm (2998
Date Acquired:	6/2/2020 7:49:10 PM CST		
Date Processed:	8/21/2020 9:51:22 PM CST		

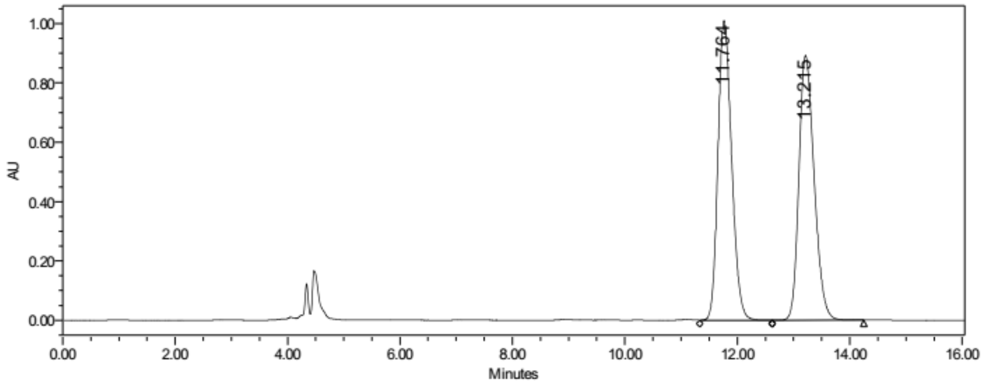

	RT	Area	% Area	Height
1	11.764	16905066	50.01	1009290
2	13.215	16896584	49.99	892210

Asy-**3m**

SAMPLE INFORMATION

Sample Name:	cxh-7-69-3-IC-3%	Acquired By:	System
Sample Type:	Unknown	Sample Set Name:	
Vial:	17	Acq. Method Set:	3%
Injection #:	1	Processing Method	3m2
Injection Volume:	10.00 ul	Channel Name:	211.0nm
Run Time:	35.0 Minutes	Proc. Chnl. Descr.:	2998 PDA 211.0 nm (2998
Date Acquired:	6/2/2020 8:06:16 PM CST		
Date Processed:	8/21/2020 9:52:10 PM CST		

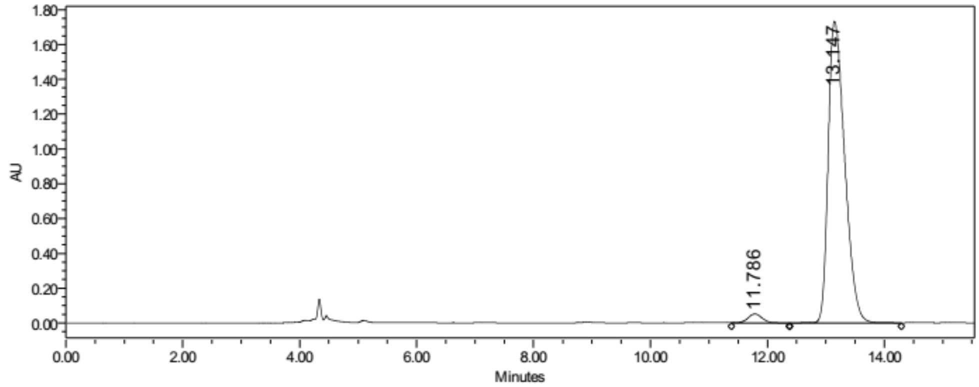

	RT	Area	% Area	Height
1	11.786	886869	2.55	52041
2	13.147	33883782	97.45	1732634

Rac-**3n**

SAMPLE INFORMATION

Sample Name:	cxh-7-34-2-rac-IC-3%	Acquired By:	System
Sample Type:	Unknown	Sample Set Name:	
Vial:	58	Acq. Method Set:	3%
Injection #:	1	Processing Method	3n
Injection Volume:	10.00 ul	Channel Name:	211.0nm
Run Time:	24.0 Minutes	Proc. Chnl. Descr.:	2998 PDA 211.0 nm (2998
Date Acquired:	6/2/2020 7:11:47 PM CST		
Date Processed:	8/21/2020 9:59:00 PM CST		

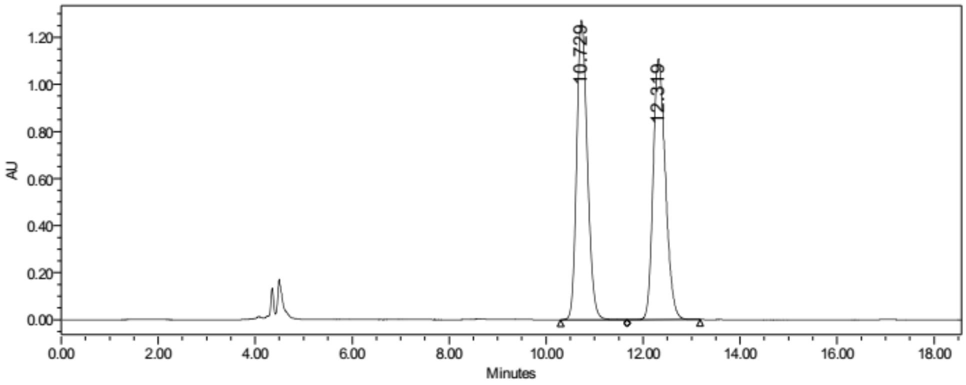

	RT	Area	% Area	Height
1	10.729	19562311	50.17	1270762
2	12.319	19426933	49.83	1108157

Asy-**3n**

SAMPLE INFORMATION

Sample Name:	cxh-7-69-2-IC-3%	Acquired By:	System
Sample Type:	Unknown	Sample Set Name:	
Vial:	79	Acq. Method Set:	3%
Injection #:	1	Processing Method	3n2
Injection Volume:	10.00 ul	Channel Name:	211.0nm
Run Time:	24.0 Minutes	Proc. Chnl. Descr.:	2998 PDA 211.0 nm (2998
Date Acquired:	6/2/2020 7:32:07 PM CST		
Date Processed:	8/21/2020 9:59:46 PM CST		

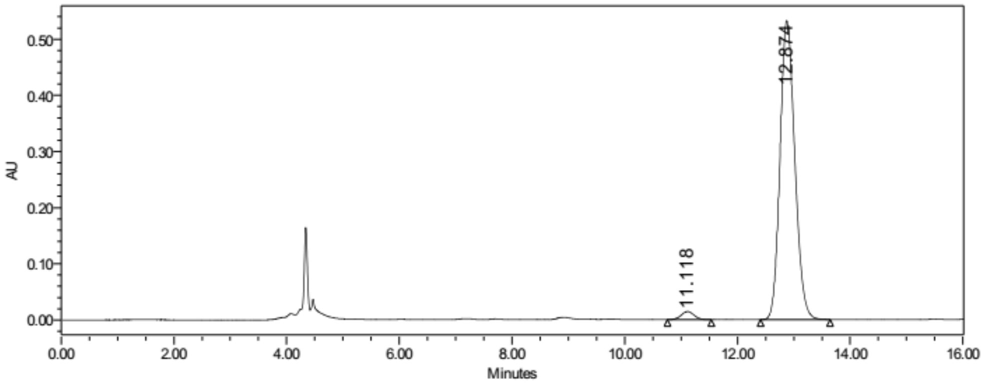

	RT	Area	% Area	Height
1	11.118	232308	2.34	14578
2	12.874	9690470	97.66	532175

Rac-**3o**

SAMPLE INFORMATION

Sample Name:	cxh-7-34-1-RAC-IC-3%	Acquired By:	System
Sample Type:	Unknown	Sample Set Name:	
Vial:	65	Acq. Method Set:	3%
Injection #:	1	Processing Method	3o0
Injection Volume:	10.00 ul	Channel Name:	220.0nm
Run Time:	80.0 Minutes	Proc. Chnl. Descr.:	2998 PDA 220.0 nm (2998
Date Acquired:	5/29/2020 9:09:48 PM CST		
Date Processed:	8/22/2020 3:04:00 PM CST		

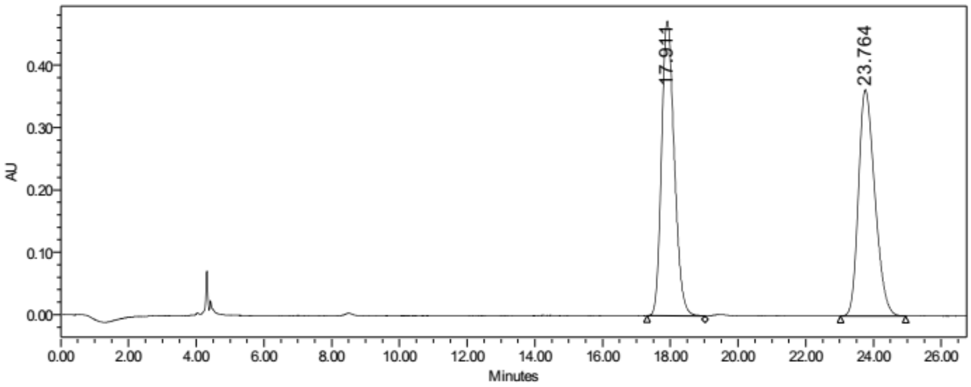

	RT	Area	% Area	Height
1	17.911	12232343	49.95	472316
2	23.764	12256885	50.05	362846

Asy-**3o**

SAMPLE INFORMATION

Sample Name:	cxh-7-67-3-IC-3%	Acquired By:	System
Sample Type:	Unknown	Sample Set Name:	05292
Vial:	75	Acq. Method Set:	3%
Injection #:	1	Processing Method	3o2
Injection Volume:	10.00 ul	Channel Name:	220.0nm
Run Time:	28.0 Minutes	Proc. Chnl. Descr.:	2998 PDA 220.0 nm (2998
Date Acquired:	5/29/2020 10:21:34 PM CST		
Date Processed:	8/22/2020 3:02:10 PM CST		

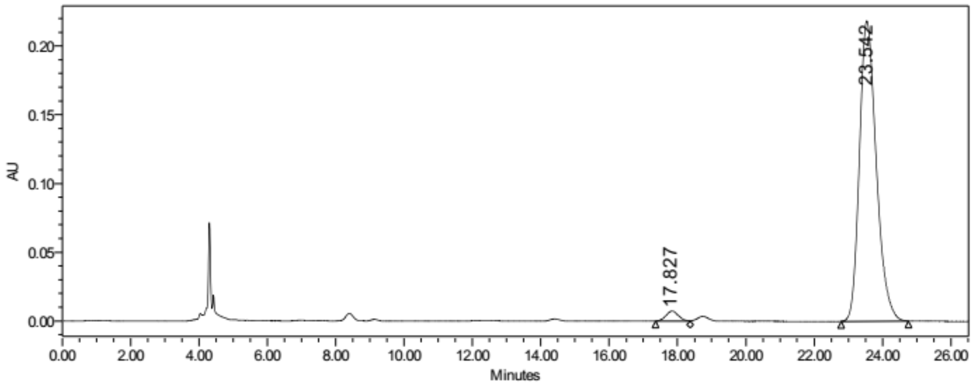

	RT	Area	% Area	Height
1	17.827	187374	2.43	7314
2	23.542	7534805	97.57	218454

Rac-**3p**

SAMPLE INFORMATION

Sample Name:	cxh-7-37-1-IC-10%-rac	Acquired By:	System
Sample Type:	Unknown	Sample Set Name:	
Vial:	85	Acq. Method Set:	10%
Injection #:	1	Processing Method	3p
Injection Volume:	10.00 ul	Channel Name:	230.0nm
Run Time:	50.0 Minutes	Proc. Chnl. Descr.:	2998 PDA 230.0 nm (2998
Date Acquired:	5/13/2020 5:21:26 PM CST		
Date Processed:	8/22/2020 3:10:19 PM CST		

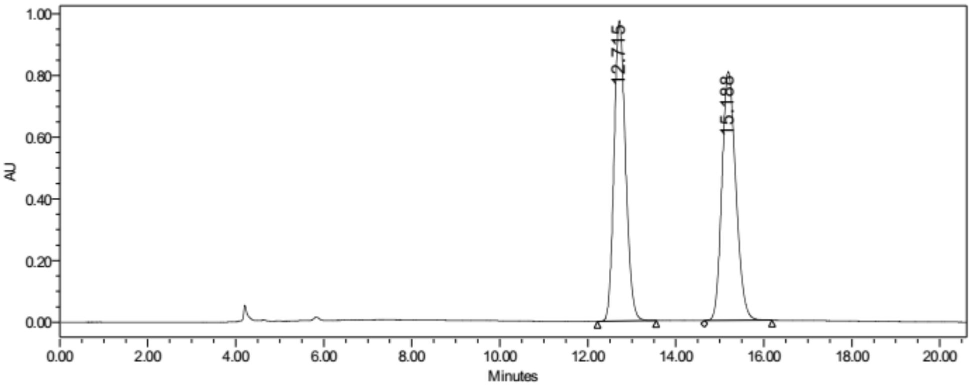

	RT	Area	% Area	Height
1	12.715	17508590	49.96	974085
2	15.188	17537437	50.04	806742

Asy-**3p**

SAMPLE INFORMATION

Sample Name:	cxh-7-60-4-IC-10%	Acquired By:	System
Sample Type:	Unknown	Sample Set Name:	
Vial:	40	Acq. Method Set:	10%
Injection #:	2	Processing Method	3p2
Injection Volume:	10.00 ul	Channel Name:	230.0nm
Run Time:	40.0 Minutes	Proc. Chnl. Descr.:	2998 PDA 230.0 nm (2998
Date Acquired:	5/22/2020 9:26:12 PM CST		
Date Processed:	8/22/2020 3:10:54 PM CST		

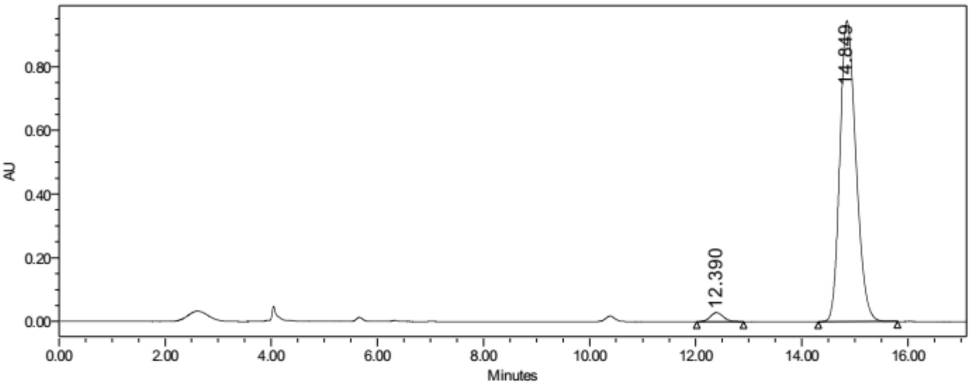

	RT	Area	% Area	Height
1	12.390	489768	2.40	28458
2	14.849	19896467	97.60	944797

Rac-**3q**

SAMPLE INFORMATION			
Sample Name:	hzy-1-26-2-ic-5%-re-rac	Acquired By:	System
Sample Type:	Unknown	Sample Set Name:	
Vial:	94	Acq. Method Set:	5%
Injection #:	1	Processing Method	3q
Injection Volume:	10.00 ul	Channel Name:	254.0nm
Run Time:	30.0 Minutes	Proc. Chnl. Descr.:	2998 PDA 254.0 nm (2998
Date Acquired:	5/18/2020 3:13:28 PM CST		
Date Processed:	8/22/2020 3:18:11 PM CST		

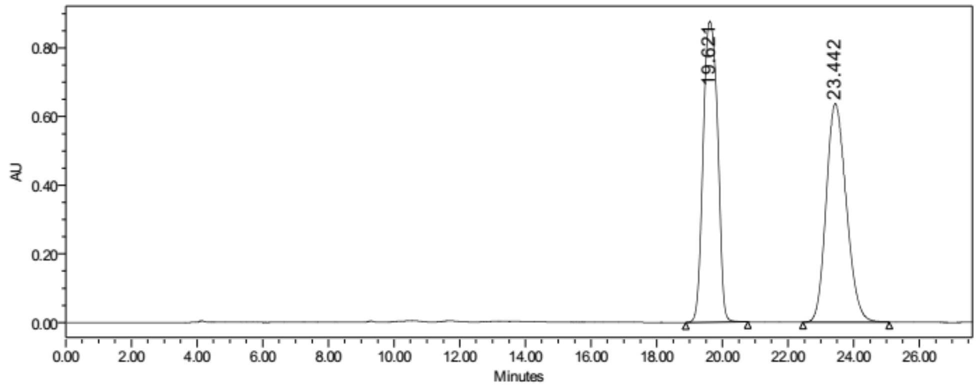

	RT	Area	% Area	Height
1	19.621	27042524	50.08	876797
2	23.442	26952796	49.92	636917

Asy-**3q**

SAMPLE INFORMATION			
Sample Name:	hzy-1-26-2-ic-5%-re	Acquired By:	System
Sample Type:	Unknown	Sample Set Name:	
Vial:	66	Acq. Method Set:	5%
Injection #:	2	Processing Method	3q2
Injection Volume:	10.00 ul	Channel Name:	254.0nm
Run Time:	30.0 Minutes	Proc. Chnl. Descr.:	2998 PDA 254.0 nm (2998
Date Acquired:	5/18/2020 2:44:27 PM CST		
Date Processed:	8/22/2020 3:17:37 PM CST		

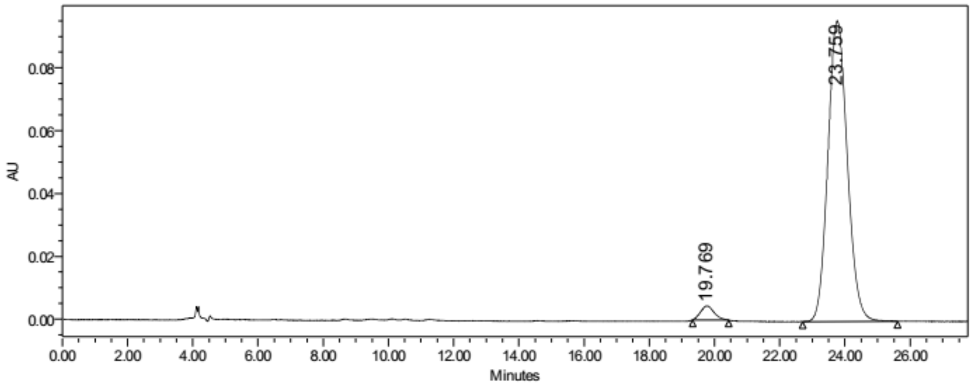

	RT	Area	% Area	Height
1	19.769	128693	3.18	4510
2	23.759	3923131	96.82	95746

Rac-**3r**

SAMPLE INFORMATION

Sample Name:	cxh-7-34-4-rac-IC-3%	Acquired By:	System
Sample Type:	Unknown	Sample Set Name:	
Vial:	107	Acq. Method Set:	3%
Injection #:	1	Processing Method	3r1
Injection Volume:	10.00 ul	Channel Name:	211.0nm
Run Time:	30.0 Minutes	Proc. Chnl. Descr.:	2998 PDA 211.0 nm (2998
Date Acquired:	6/6/2020 2:39:47 PM CST		
Date Processed:	8/22/2020 3:28:58 PM CST		

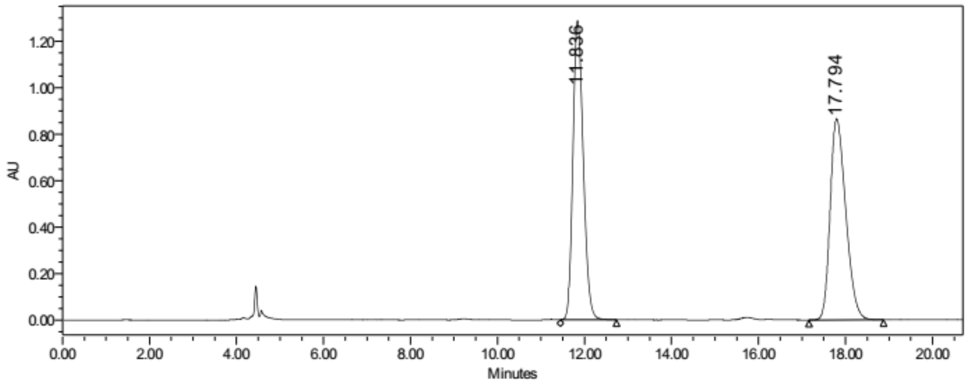

	RT	Area	% Area	Height
1	11.836	21375011	49.86	1287312
2	17.794	21494330	50.14	866163

Asy-**3r**

SAMPLE INFORMATION

Sample Name:	cxh-7-69-4-IC-3%	Acquired By:	System
Sample Type:	Unknown	Sample Set Name:	
Vial:	26	Acq. Method Set:	3%
Injection #:	1	Processing Method	3r2
Injection Volume:	10.00 ul	Channel Name:	211.0nm
Run Time:	30.0 Minutes	Proc. Chnl. Descr.:	2998 PDA 211.0 nm (2998
Date Acquired:	6/6/2020 3:32:08 PM CST		
Date Processed:	8/22/2020 5:00:02 PM CST		

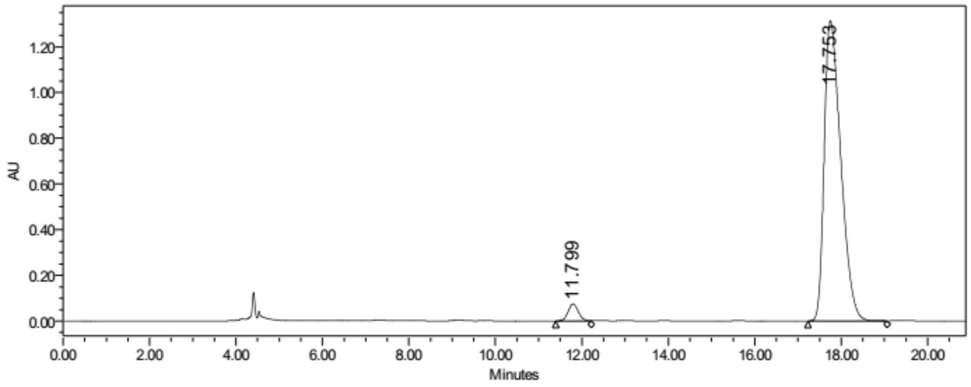

	RT	Area	% Area	Height
1	11.799	1241975	3.45	74698
2	17.753	34720671	96.55	1313893

Rac-**3s**

SAMPLE INFORMATION			
Sample Name:	cxh-7-34-5-rac-IC-3%	Acquired By:	System
Sample Type:	Unknown	Sample Set Name:	
Vial:	18	Acq. Method Set:	3%
Injection #:	1	Processing Method	3s
Injection Volume:	10.00 ul	Channel Name:	211.0nm
Run Time:	40.0 Minutes	Proc. Chnl. Descr.:	2998 PDA 211.0 nm (2998
Date Acquired:	6/6/2020 3:02:08 PM CST		
Date Processed:	8/22/2020 5:04:03 PM CST		

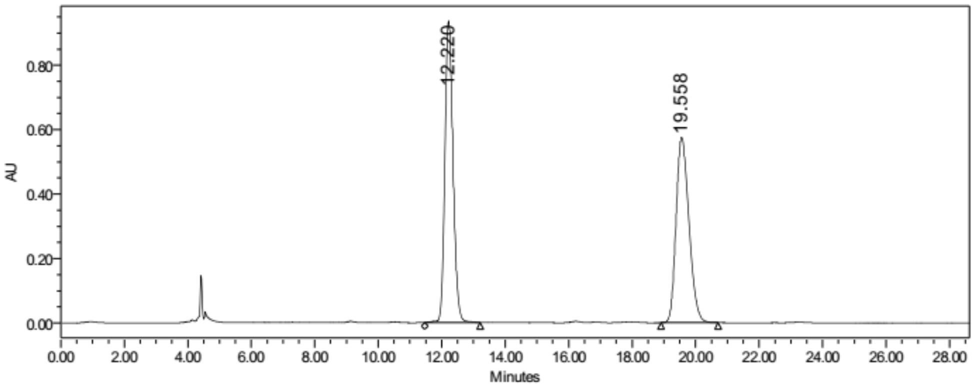

	RT	Area	% Area	Height
1	12.220	16391888	50.36	935760
2	19.558	16155033	49.64	575107

Asy-**3s**

SAMPLE INFORMATION			
Sample Name:	cxh-7-69-5-IC-3%	Acquired By:	System
Sample Type:	Unknown	Sample Set Name:	
Vial:	60	Acq. Method Set:	3%
Injection #:	1	Processing Method	3s2
Injection Volume:	10.00 ul	Channel Name:	211.0nm
Run Time:	30.0 Minutes	Proc. Chnl. Descr.:	2998 PDA 211.0 nm (2998
Date Acquired:	6/6/2020 3:54:00 PM CST		
Date Processed:	8/22/2020 5:03:31 PM CST		

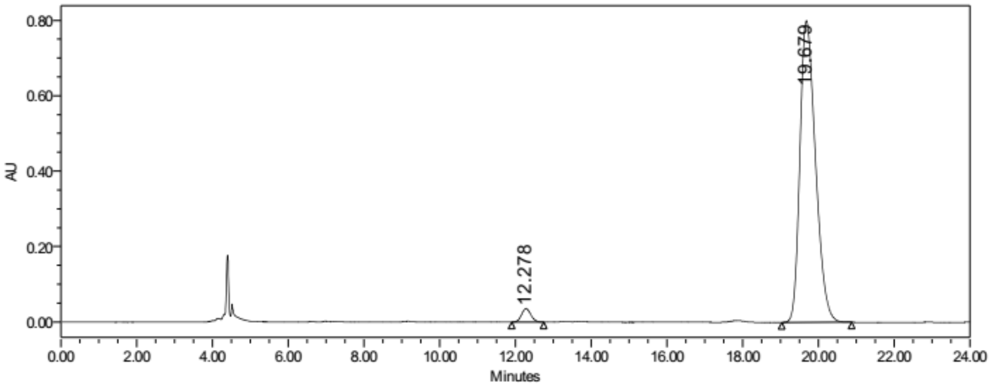

	RT	Area	% Area	Height
1	12.278	611405	2.59	35630
2	19.679	23015355	97.41	799833

Rac-**3t**

SAMPLE INFORMATION			
Sample Name:	cxh-7-35-1-RAC-OD-1%	Acquired By:	System
Sample Type:	Unknown	Sample Set Name:	
Vial:	18	Acq. Method Set:	1%
Injection #:	1	Processing Method	3t
Injection Volume:	10.00 ul	Channel Name:	254.0nm
Run Time:	40.0 Minutes	Proc. Chnl. Descr.:	2998 PDA 254.0 nm (2998
Date Acquired:	5/24/2020 3:23:14 PM CST		
Date Processed:	8/22/2020 5:13:24 PM CST		

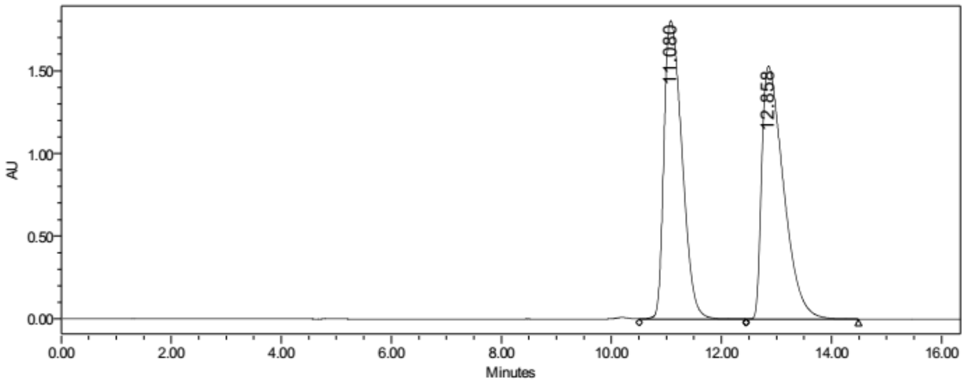

	RT	Area	% Area	Height
1	11.080	40950513	49.53	1803827
2	12.858	41727626	50.47	1529909

Asy-**3t**

SAMPLE INFORMATION			
Sample Name:	cxh-7-62-1-OD-1%	Acquired By:	System
Sample Type:	Unknown	Sample Set Name:	
Vial:	17	Acq. Method Set:	1%
Injection #:	1	Processing Method	3t2
Injection Volume:	10.00 ul	Channel Name:	254.0nm
Run Time:	40.0 Minutes	Proc. Chnl. Descr.:	2998 PDA 254.0 nm (2998
Date Acquired:	5/24/2020 3:40:55 PM CST		
Date Processed:	8/22/2020 5:12:58 PM CST		

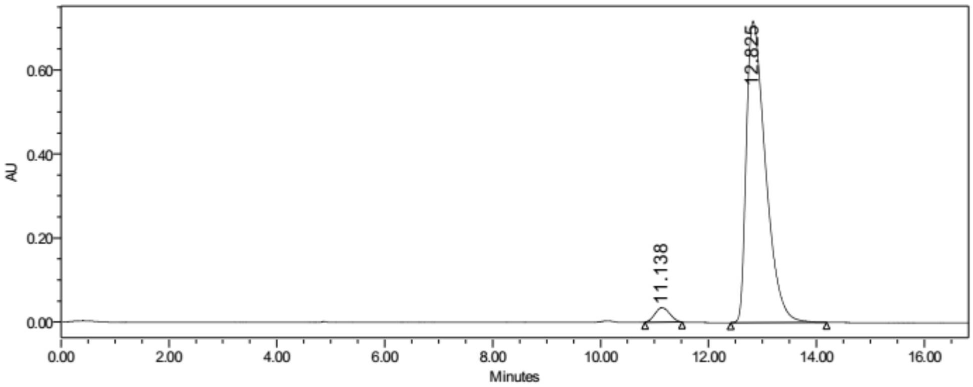

	RT	Area	% Area	Height
1	11.138	652320	3.54	34164
2	12.825	17774253	96.46	717258

Rac-**3u**

SAMPLE INFORMATION

Sample Name:	cxh-7-35-4-rac-oj-2%	Acquired By:	System
Sample Type:	Unknown	Sample Set Name:	
Vial:	24	Acq. Method Set:	2%
Injection #:	2	Processing Method	3u
Injection Volume:	10.00 ul	Channel Name:	254.0nm
Run Time:	30.0 Minutes	Proc. Chnl. Descr.:	2998 PDA 254.0 nm (2998
Date Acquired:	5/16/2020 7:29:51 PM CST		
Date Processed:	8/22/2020 5:18:05 PM CST		

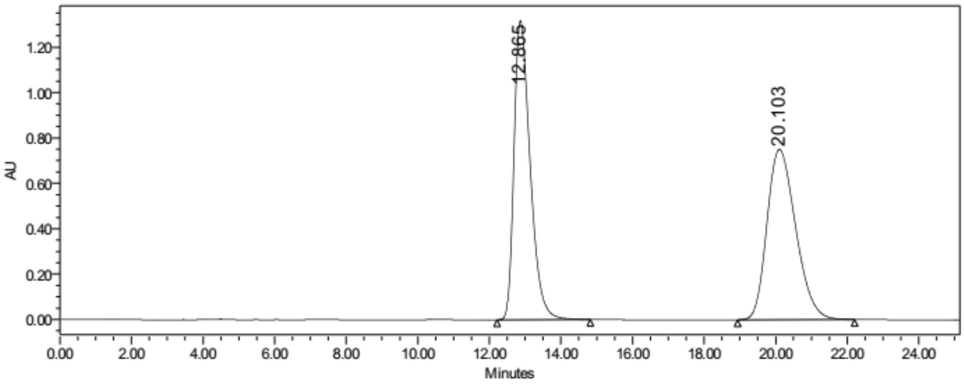

	RT	Area	% Area	Height
1	12.865	41491460	49.93	1317529
2	20.103	41611301	50.07	750556

Asy-**3u**

SAMPLE INFORMATION

Sample Name:	cxh-7-62-4-OJ-2%	Acquired By:	System
Sample Type:	Unknown	Sample Set Name:	
Vial:	100	Acq. Method Set:	2%
Injection #:	1	Processing Method	3u2
Injection Volume:	10.00 ul	Channel Name:	254.0nm
Run Time:	40.0 Minutes	Proc. Chnl. Descr.:	2998 PDA 254.0 nm (2998
Date Acquired:	5/24/2020 5:30:40 PM CST		
Date Processed:	8/22/2020 5:15:08 PM CST		

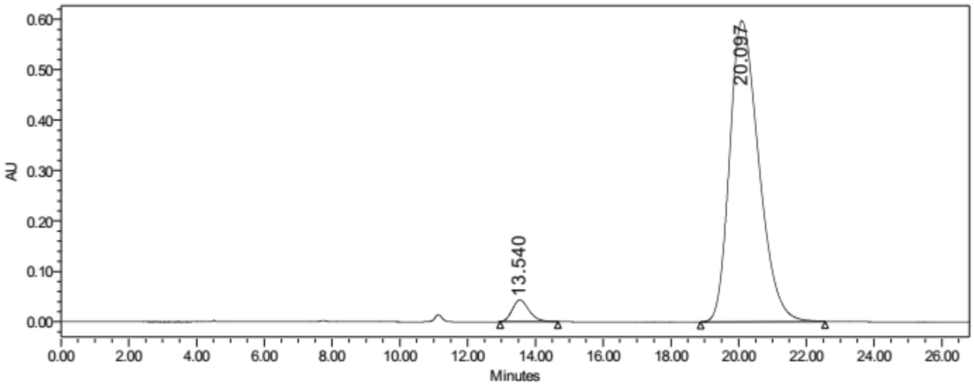

	RT	Area	% Area	Height
1	13.540	1481760	3.99	43147
2	20.097	35673767	96.01	596941

Rac-**3v**

SAMPLE INFORMATION

Sample Name:	cxh-7-35-5-rac-2%-OJ	Acquired By:	System
Sample Type:	Unknown	Sample Set Name:	
Vial:	83	Acq. Method Set:	2%
Injection #:	1	Processing Method	3v
Injection Volume:	10.00 ul	Channel Name:	254.0nm
Run Time:	60.0 Minutes	Proc. Chnl. Descr.:	2998 PDA 254.0 nm (2998
Date Acquired:	5/20/2020 3:14:59 PM CST		
Date Processed:	8/22/2020 5:21:44 PM CST		

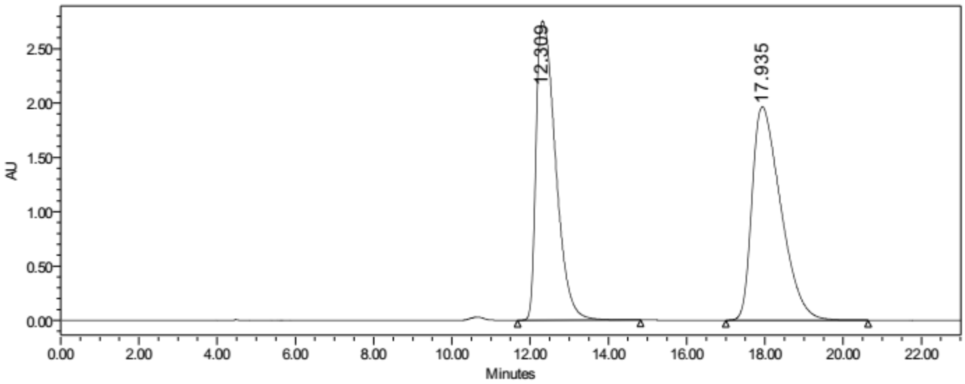

	RT	Area	% Area	Height
1	12.309	95105154	48.72	2755028
2	17.935	100116609	51.28	1965955

Asy-**3v**

SAMPLE INFORMATION

Sample Name:	cxh-7-60-1-2%-OJ	Acquired By:	System
Sample Type:	Unknown	Sample Set Name:	
Vial:	42	Acq. Method Set:	2%
Injection #:	1	Processing Method	3v2
Injection Volume:	10.00 ul	Channel Name:	254.0nm
Run Time:	60.0 Minutes	Proc. Chnl. Descr.:	2998 PDA 254.0 nm (2998
Date Acquired:	5/20/2020 4:58:59 PM CST		
Date Processed:	8/22/2020 5:22:16 PM CST		

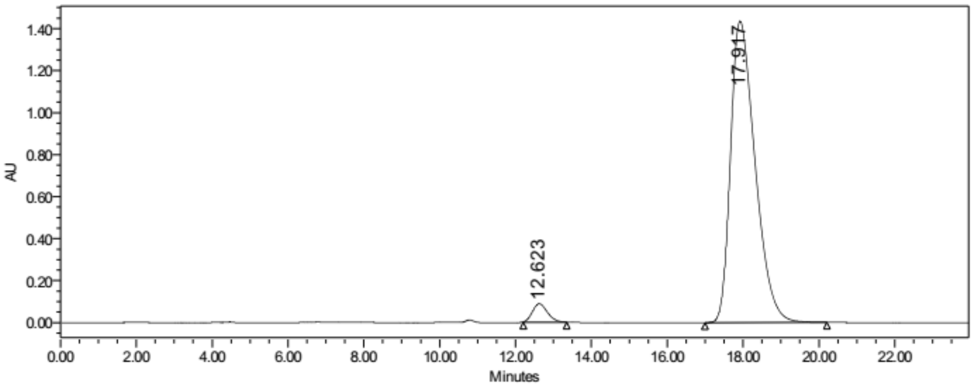

	RT	Area	% Area	Height
1	12.623	2372899	3.54	87306
2	17.917	64732669	96.46	1435510

Rac-**3w**

SAMPLE INFORMATION

Sample Name:	cxh-8-21-6-IC-rac -2%	Acquired By:	System
Sample Type:	Unknown	Sample Set Name	
Vial:	8	Acq. Method Set:	2%
Injection #:	2	Processing Method	CF3 2% ic rac
Injection Volume:	10.00 ul	Channel Name:	254.0nm
Run Time:	50.0 Minutes	Proc. Chnl. Descr.:	2998 PDA 254.0 nm (2998
Date Acquired:	10/15/2020 10:59:38 AM CST		
Date Processed:	10/28/2020 2:47:45 PM CST		

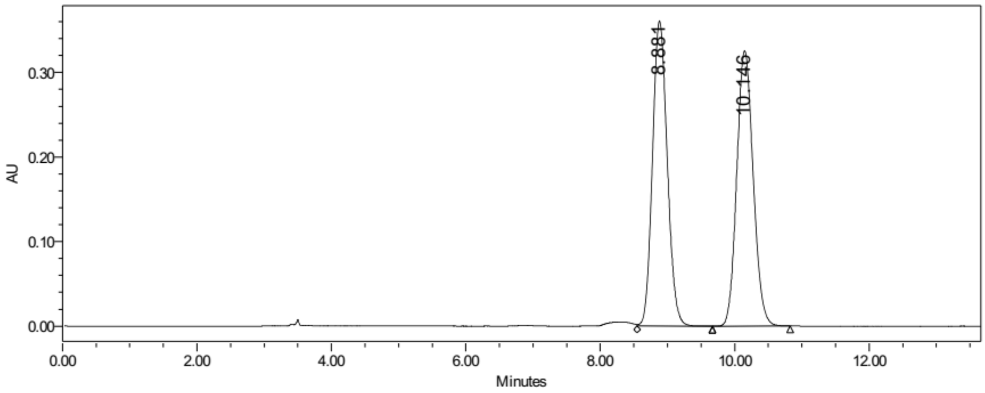

	RT	Area	% Area	Height
1	8.881	5524117	50.09	360823
2	10.146	5503802	49.91	325711

Asy-**3w**

SAMPLE INFORMATION

Sample Name:	cxh-8-29-2-IC-2%	Acquired By:	System
Sample Type:	Unknown	Sample Set Name	1024
Vial:	71	Acq. Method Set:	2%
Injection #:	1	Processing Method	CF3 ic 2% asy
Injection Volume:	10.00 ul	Channel Name:	254.0nm
Run Time:	15.0 Minutes	Proc. Chnl. Descr.:	2998 PDA 254.0 nm (2998
Date Acquired:	10/24/2020 4:45:09 PM CST		
Date Processed:	10/28/2020 2:44:10 PM CST		

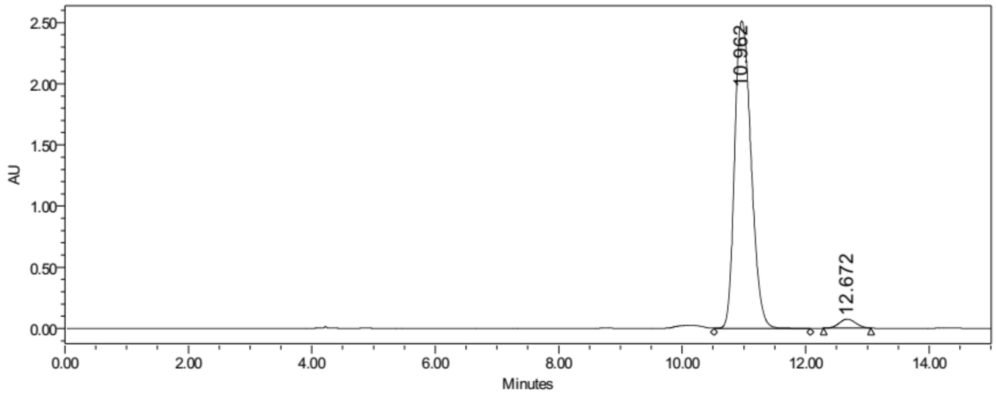

	RT	Area	% Area	Height
1	10.962	46149107	97.05	2511545
2	12.672	1400691	2.95	73661

Rac-**3x**

SAMPLE INFORMATION

Sample Name:	cxh-8-21-5-IC-rac -5%	Acquired By:	System
Sample Type:	Unknown	Sample Set Name	
Vial:	7	Acq. Method Set:	5%
Injection #:	1	Processing Method	CO2Me5%IC
Injection Volume:	10.00 ul	Channel Name:	254.0nm
Run Time:	50.0 Minutes	Proc. Chnl. Descr.:	2998 PDA 254.0 nm (2998
Date Acquired:	10/15/2020 10:21:42 AM CST		
Date Processed:	10/28/2020 2:38:53 PM CST		

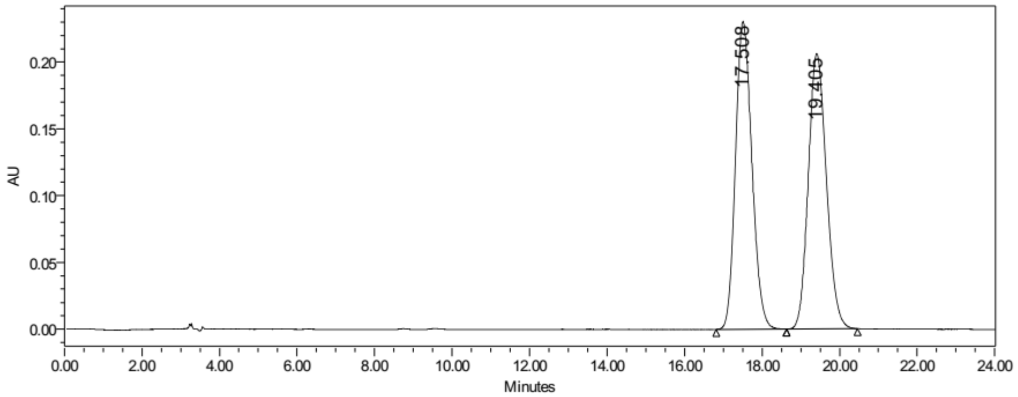

	RT	Area	% Area	Height
1	17.508	6714304	50.07	230541
2	19.405	6695952	49.93	206058

Asy-**3x**

SAMPLE INFORMATION

Sample Name:	cxh-8-25-6-ASY-IC-5%	Acquired By:	System
Sample Type:	Unknown	Sample Set Name	
Vial:	53	Acq. Method Set:	5%
Injection #:	1	Processing Method	CO2ME5%ICASY
Injection Volume:	10.00 ul	Channel Name:	254.0nm
Run Time:	60.0 Minutes	Proc. Chnl. Descr.:	2998 PDA 254.0 nm (2998
Date Acquired:	10/21/2020 7:38:04 PM CST		
Date Processed:	10/28/2020 2:41:16 PM CST		

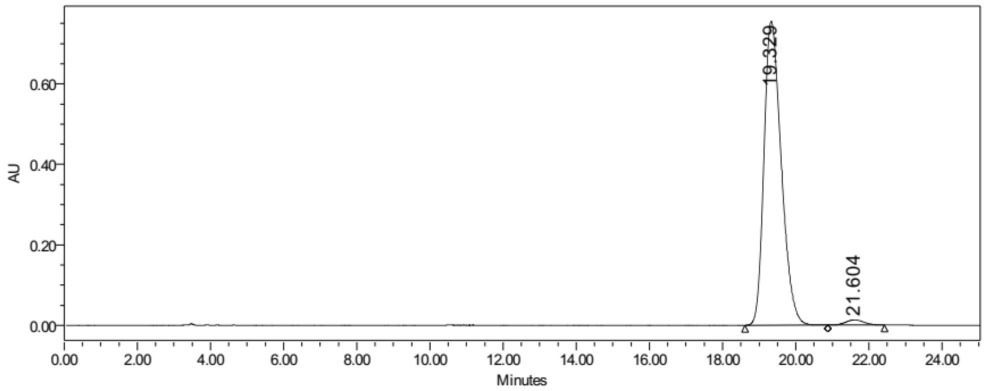

	RT	Area	% Area	Height
1	19.329	24890306	98.15	755321
2	21.604	468718	1.85	13257

Rac-**3y**

SAMPLE INFORMATION

Sample Name:	cxh-8-23-5-rac-OD-5%	Acquired By:	System
Sample Type:	Unknown	Sample Set Name	
Vial:	9	Acq. Method Set:	5%
Injection #:	1	Processing Method	OD5%CN
Injection Volume:	10.00 ul	Channel Name:	254.0nm
Run Time:	40.0 Minutes	Proc. Chnl. Descr.:	2998 PDA 254.0 nm (2998
Date Acquired:	10/18/2020 9:42:31 PM CST		
Date Processed:	10/28/2020 2:32:38 PM CST		

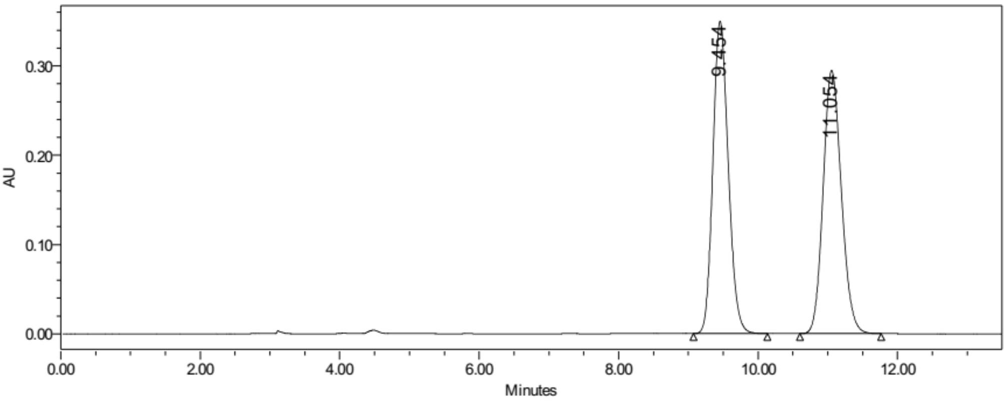

	RT	Area	% Area	Height
1	9.454	5374283	49.98	349605
2	11.054	5379257	50.02	294695

Asy-**3y**

SAMPLE INFORMATION

Sample Name:	CXH-8-25-5-5%-OD	Acquired By:	System
Sample Type:	Unknown	Sample Set Name	1018
Vial:	18	Acq. Method Set:	5%
Injection #:	1	Processing Method	CN5%OD
Injection Volume:	10.00 ul	Channel Name:	254.0nm
Run Time:	15.0 Minutes	Proc. Chnl. Descr.:	2998 PDA 254.0 nm (2998
Date Acquired:	10/18/2020 9:26:03 PM CST		
Date Processed:	10/28/2020 2:35:22 PM CST		

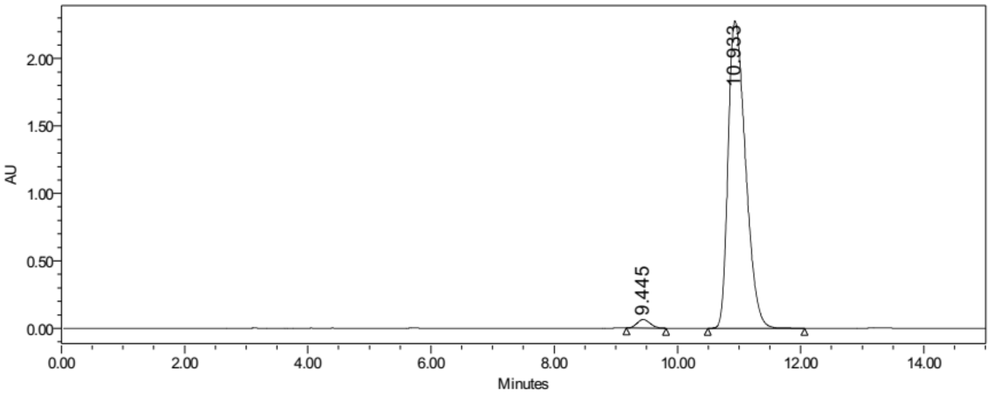

	RT	Area	% Area	Height
1	9.445	953091	2.10	63847
2	10.933	44376057	97.90	2278485

Rac-**3z**

SAMPLE INFORMATION

Sample Name:	cxh-7-35-2-rac-od-1%	Acquired By:	System
Sample Type:	Unknown	Sample Set Name:	
Vial:	20	Acq. Method Set:	1%
Injection #:	1	Processing Method	3w
Injection Volume:	10.00 ul	Channel Name:	254.0nm
Run Time:	50.0 Minutes	Proc. Chnl. Descr.:	2998 PDA 254.0 nm (2998
Date Acquired:	5/16/2020 5:02:28 PM CST		
Date Processed:	8/22/2020 5:25:10 PM CST		

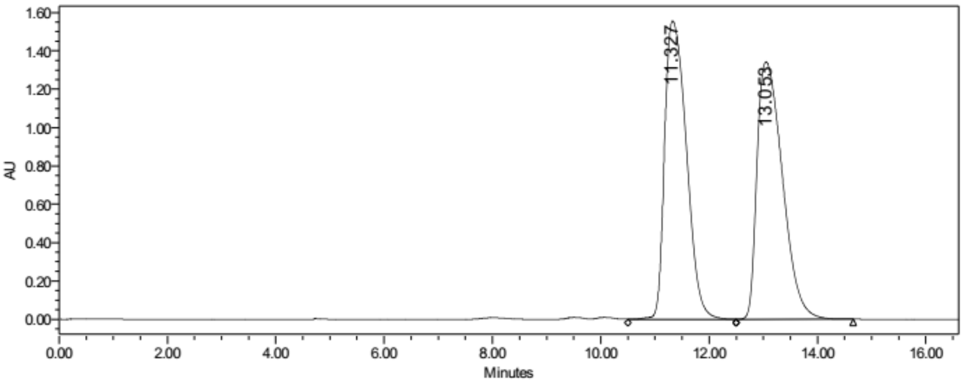

	RT	Area	% Area	Height
1	11.327	43451344	49.89	1555916
2	13.053	43640913	50.11	1342574

Asy-**3z**

SAMPLE INFORMATION

Sample Name:	cxh-7-62-2-OD-1%	Acquired By:	System
Sample Type:	Unknown	Sample Set Name:	
Vial:	36	Acq. Method Set:	1%
Injection #:	1	Processing Method	3w2
Injection Volume:	10.00 ul	Channel Name:	254.0nm
Run Time:	40.0 Minutes	Proc. Chnl. Descr.:	2998 PDA 254.0 nm (2998
Date Acquired:	5/24/2020 3:59:01 PM CST		
Date Processed:	8/22/2020 5:24:16 PM CST		

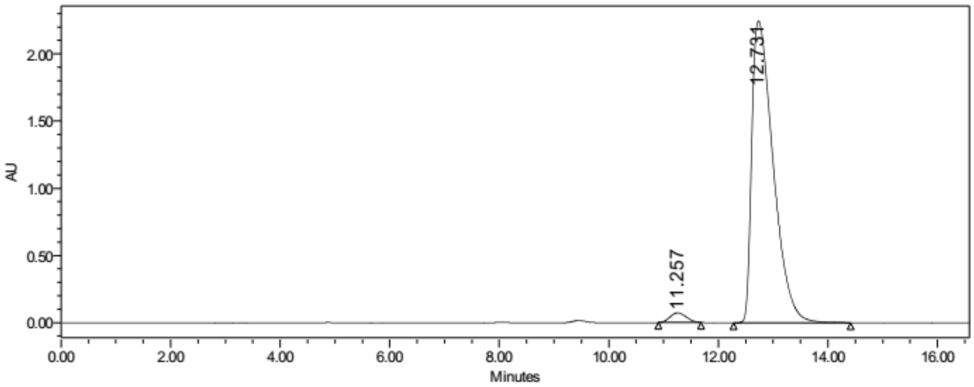

	RT	Area	% Area	Height
1	11.257	1501783	2.40	71908
2	12.731	61183817	97.60	2244915

Rac-**3aa**

SAMPLE INFORMATION

Sample Name:	cxh-7-35-3-rac-od-1%	Acquired By:	System
Sample Type:	Unknown	Sample Set Name:	
Vial:	22	Acq. Method Set:	1%
Injection #:	2	Processing Method	3x
Injection Volume:	10.00 ul	Channel Name:	254.0nm
Run Time:	30.0 Minutes	Proc. Chnl. Descr.:	2998 PDA 254.0 nm (2998
Date Acquired:	5/16/2020 6:16:58 PM CST		
Date Processed:	8/22/2020 5:26:47 PM CST		

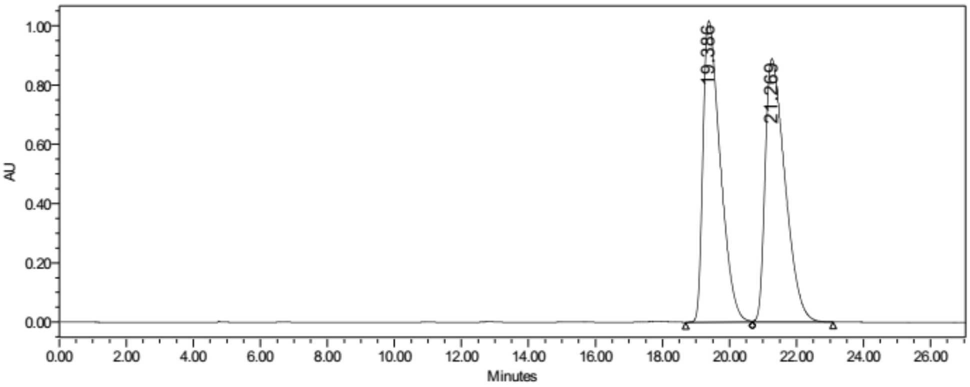

	RT	Area	% Area	Height
1	19.386	36345623	49.96	1016431
2	21.269	36402813	50.04	888633

Asy-**3aa**

SAMPLE INFORMATION

Sample Name:	cxh-7-62-3-OD-1%	Acquired By:	System
Sample Type:	Unknown	Sample Set Name:	
Vial:	65	Acq. Method Set:	1%
Injection #:	1	Processing Method	3x2
Injection Volume:	10.00 ul	Channel Name:	254.0nm
Run Time:	40.0 Minutes	Proc. Chnl. Descr.:	2998 PDA 254.0 nm (2998
Date Acquired:	5/24/2020 4:16:48 PM CST		
Date Processed:	8/22/2020 5:27:42 PM CST		

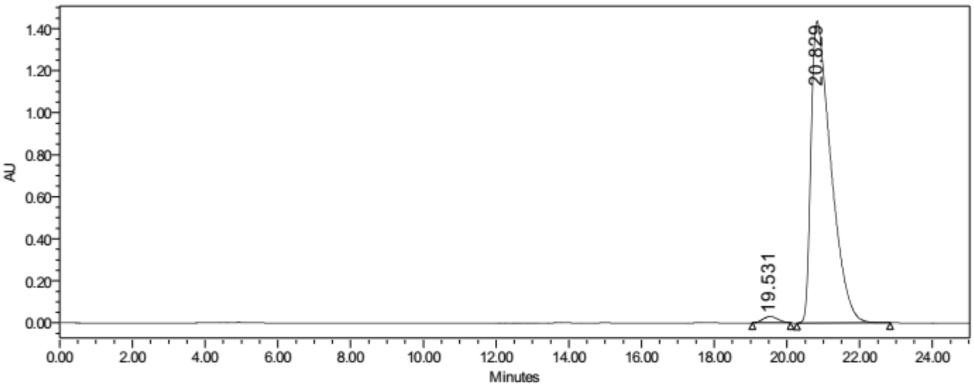

	RT	Area	% Area	Height
1	19.531	872710	1.57	30264
2	20.829	54669536	98.43	1435679

Rac-**3ab**

SAMPLE INFORMATION

Sample Name:	cxh-7-38-2-rac-IC-5%	Acquired By:	System
Sample Type:	Unknown	Sample Set Name:	
Vial:	74	Acq. Method Set:	5%
Injection #:	2	Processing Method	3y
Injection Volume:	10.00 ul	Channel Name:	254.0nm
Run Time:	40.0 Minutes	Proc. Chnl. Descr.:	2998 PDA 254.0 nm (2998
Date Acquired:	5/25/2020 4:14:02 PM CST		
Date Processed:	8/22/2020 5:31:11 PM CST		

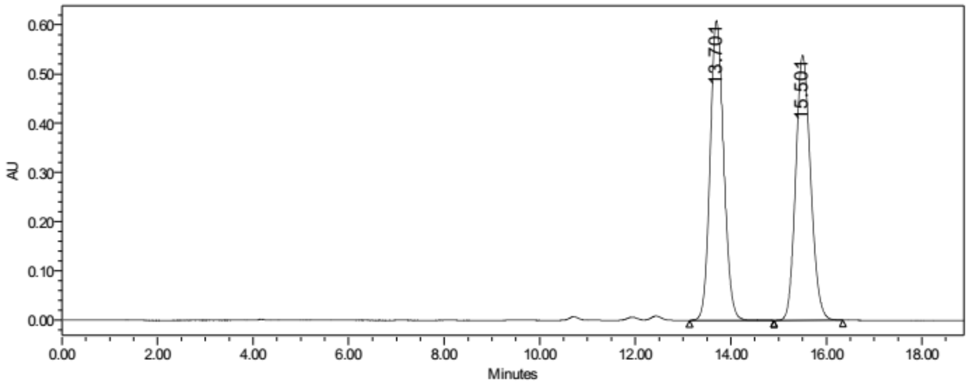

	RT	Area	% Area	Height
1	13.701	11908139	50.33	608860
2	15.501	11751667	49.67	538001

Asy-**3ab**

SAMPLE INFORMATION

Sample Name:	cxh-7-63-1-IC-5%	Acquired By:	System
Sample Type:	Unknown	Sample Set Name:	
Vial:	106	Acq. Method Set:	5%
Injection #:	1	Processing Method	3y2
Injection Volume:	10.00 ul	Channel Name:	254.0nm
Run Time:	40.0 Minutes	Proc. Chnl. Descr.:	2998 PDA 254.0 nm (2998
Date Acquired:	5/25/2020 4:34:18 PM CST		
Date Processed:	8/22/2020 5:30:39 PM CST		

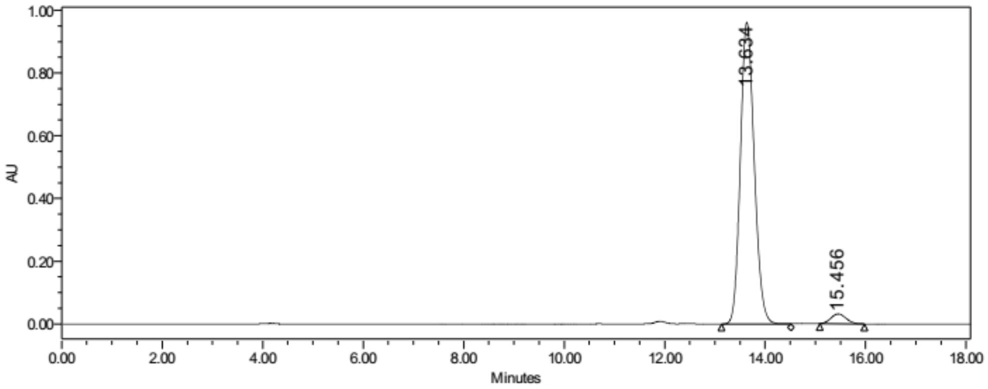

	RT	Area	% Area	Height
1	13.634	18551167	96.52	962085
2	15.456	669487	3.48	31561

Rac-**3ac**

SAMPLE INFORMATION

Sample Name:	cxh-7-38-4-RAC-IC-2%	Acquired By:	System
Sample Type:	Unknown	Sample Set Name:	
Vial:	88	Acq. Method Set:	2%
Injection #:	1	Processing Method	3z
Injection Volume:	10.00 ul	Channel Name:	254.0nm
Run Time:	40.0 Minutes	Proc. Chnl. Descr.:	2998 PDA 254.0 nm (2998
Date Acquired:	5/25/2020 7:23:01 PM CST		
Date Processed:	8/22/2020 5:46:43 PM CST		

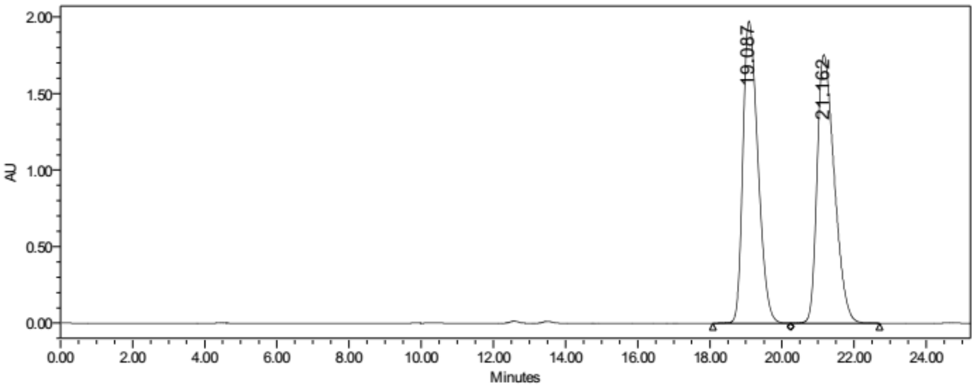

	RT	Area	% Area	Height
1	19.087	57398704	49.77	1974179
2	21.162	57940359	50.23	1755583

Asy-**3ac**

SAMPLE INFORMATION

Sample Name:	cxh-7-63-3-IC-2%	Acquired By:	System
Sample Type:	Unknown	Sample Set Name:	0525
Vial:	98	Acq. Method Set:	2%
Injection #:	1	Processing Method	3z2
Injection Volume:	10.00 ul	Channel Name:	254.0nm
Run Time:	26.0 Minutes	Proc. Chnl. Descr.:	2998 PDA 254.0 nm (2998
Date Acquired:	5/25/2020 8:14:19 PM CST		
Date Processed:	8/22/2020 5:45:47 PM CST		

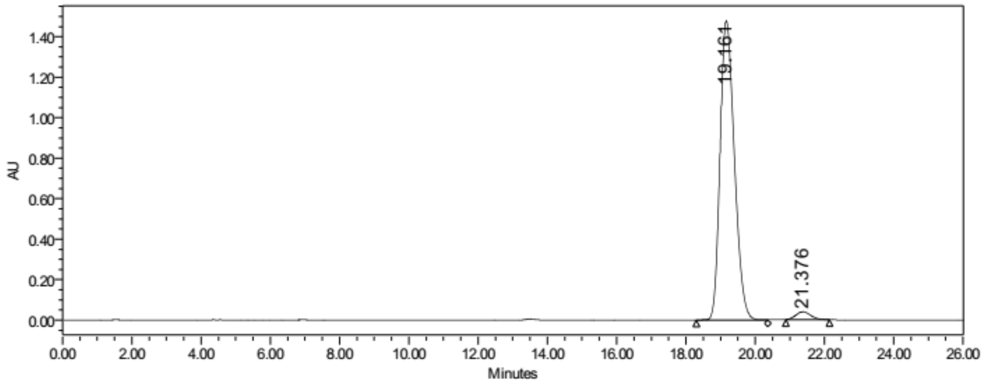

	RT	Area	% Area	Height
1	19.161	42012111	97.35	1478692
2	21.376	1143346	2.65	38547

Rac-**3ad**

SAMPLE INFORMATION

Sample Name:	cxh-7-38-3-RAC-IC-2%	Acquired By:	System
Sample Type:	Unknown	Sample Set Name:	
Vial:	67	Acq. Method Set:	2%
Injection #:	2	Processing Method	3aa
Injection Volume:	10.00 ul	Channel Name:	254.0nm
Run Time:	40.0 Minutes	Proc. Chnl. Descr.:	2998 PDA 254.0 nm (2998
Date Acquired:	5/25/2020 7:01:56 PM CST		
Date Processed:	8/22/2020 5:48:42 PM CST		

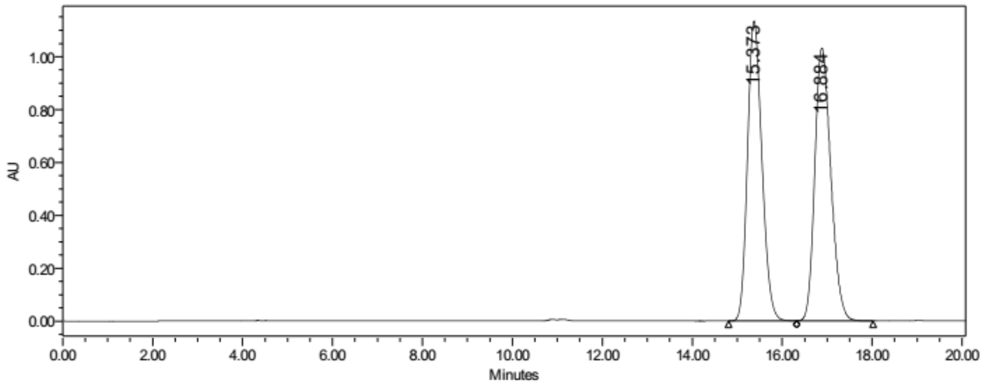

	RT	Area	% Area	Height
1	15.373	25518669	49.98	1134419
2	16.884	25541951	50.02	1032117

Asy-**3ad**

SAMPLE INFORMATION

Sample Name:	cxh-7-63-2-RE-IC-2%	Acquired By:	System
Sample Type:	Unknown	Sample Set Name:	
Vial:	66	Acq. Method Set:	2%
Injection #:	1	Processing Method	3aa2
Injection Volume:	10.00 ul	Channel Name:	254.0nm
Run Time:	80.0 Minutes	Proc. Chnl. Descr.:	2998 PDA 254.0 nm (2998
Date Acquired:	5/30/2020 7:25:03 PM CST		
Date Processed:	8/22/2020 5:51:32 PM CST		

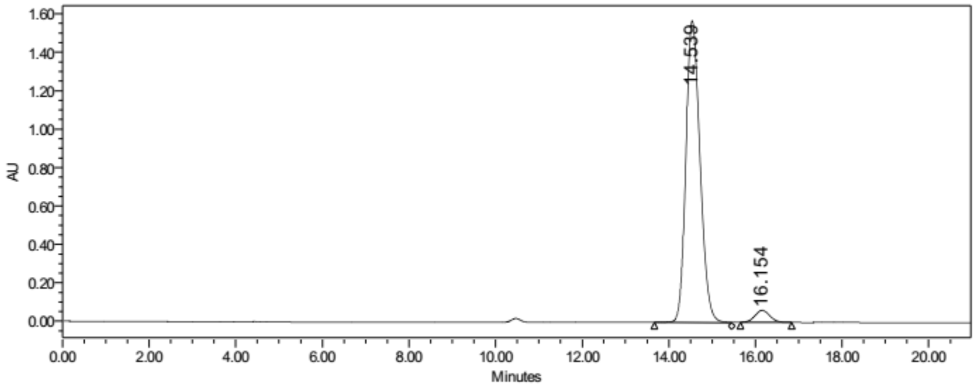

	RT	Area	% Area	Height
1	14.539	35779814	96.09	1571274
2	16.154	1456675	3.91	61868

Rac-**3ae**

SAMPLE INFORMATION

Sample Name:	cxh-8-32-2-RAC-OD-1%	Acquired By:	System
Sample Type:	Unknown	Sample Set Name	
Vial:	13	Acq. Method Set:	1%
Injection #:	1	Processing Method	32 2 rac
Injection Volume:	10.00 ul	Channel Name:	254.0nm
Run Time:	20.0 Minutes	Proc. Chnl. Descr.:	2998 PDA 254.0 nm (2998
Date Acquired:	10/31/2020 3:44:40 PM CST		
Date Processed:	10/31/2020 4:32:59 PM CST		

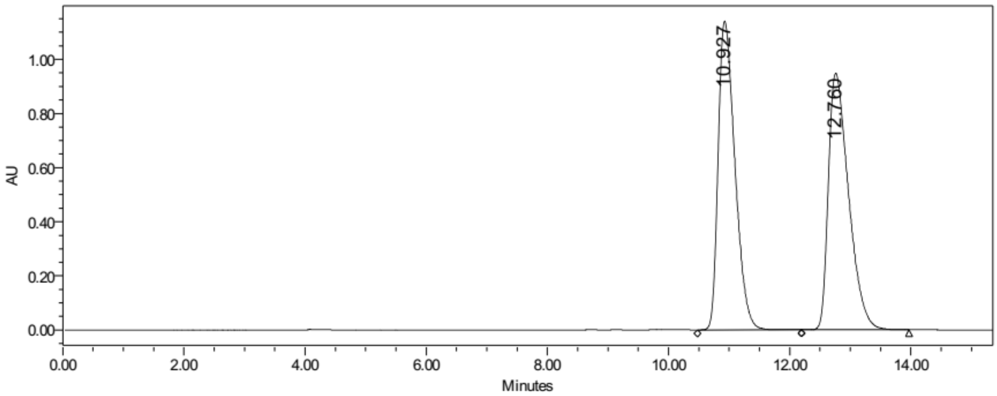

	RT	Area	% Area	Height
1	10.927	21986465	49.97	1140425
2	12.760	22017076	50.03	947988

Asy-**3ae**

SAMPLE INFORMATION

Sample Name:	cxh-8-32-1-asy-OD-1%	Acquired By:	System
Sample Type:	Unknown	Sample Set Name	
Vial:	117	Acq. Method Set:	1%
Injection #:	1	Processing Method	32 1 asy
Injection Volume:	10.00 ul	Channel Name:	254.0nm
Run Time:	17.0 Minutes	Proc. Chnl. Descr.:	2998 PDA 254.0 nm (2998
Date Acquired:	10/31/2020 4:01:32 PM CST		
Date Processed:	10/31/2020 4:31:27 PM CST		

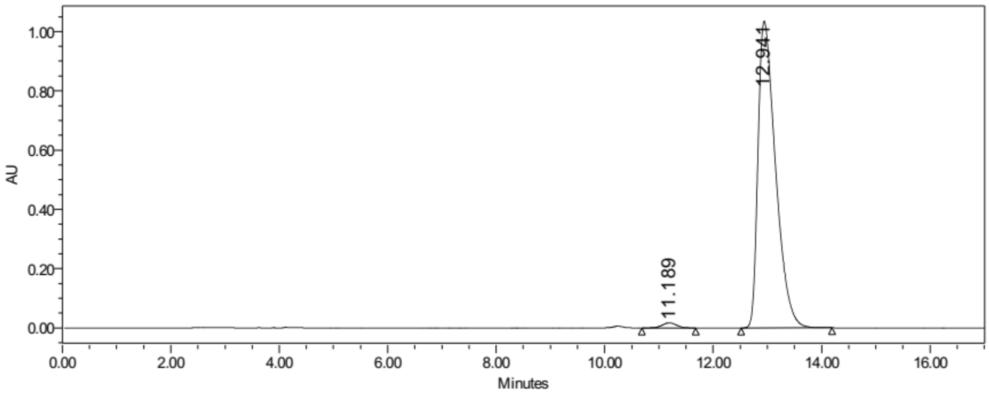

	RT	Area	% Area	Height
1	11.189	329621	1.39	17492
2	12.941	23376501	98.61	1034761

Rac-**3af**

SAMPLE INFORMATION

Sample Name:	cxh-7-38-1-RAC-IC-2%	Acquired By:	System
Sample Type:	Unknown	Sample Set Name:	
Vial:	93	Acq. Method Set:	2%
Injection #:	1	Processing Method	3ab
Injection Volume:	10.00 ul	Channel Name:	254.0nm
Run Time:	40.0 Minutes	Proc. Chnl. Descr.:	2998 PDA 254.0 nm (2998
Date Acquired:	5/24/2020 6:03:42 PM CST		
Date Processed:	8/22/2020 5:54:40 PM CST		

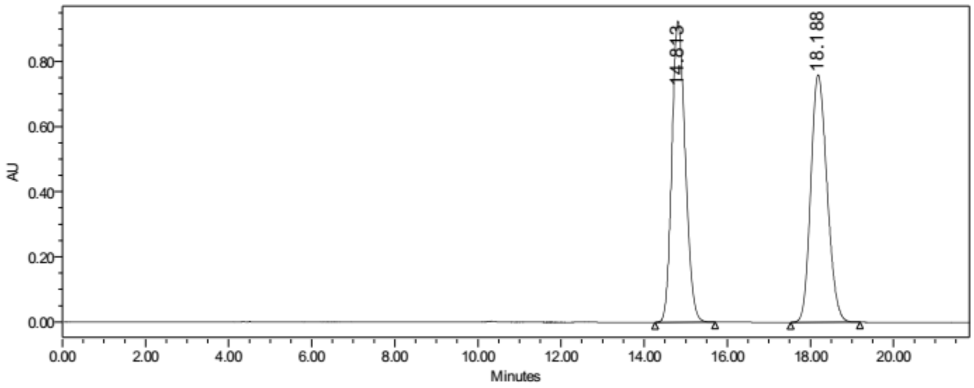

	RT	Area	% Area	Height
1	14.813	20639747	49.89	924875
2	18.188	20733079	50.11	760202

Asy-**3af**

SAMPLE INFORMATION

Sample Name:	cxh-7-62-5-IC-2%	Acquired By:	System
Sample Type:	Unknown	Sample Set Name:	
Vial:	114	Acq. Method Set:	2%
Injection #:	1	Processing Method	3ab2
Injection Volume:	10.00 ul	Channel Name:	254.0nm
Run Time:	40.0 Minutes	Proc. Chnl. Descr.:	2998 PDA 254.0 nm (2998
Date Acquired:	5/24/2020 6:27:16 PM CST		
Date Processed:	8/22/2020 5:53:57 PM CST		

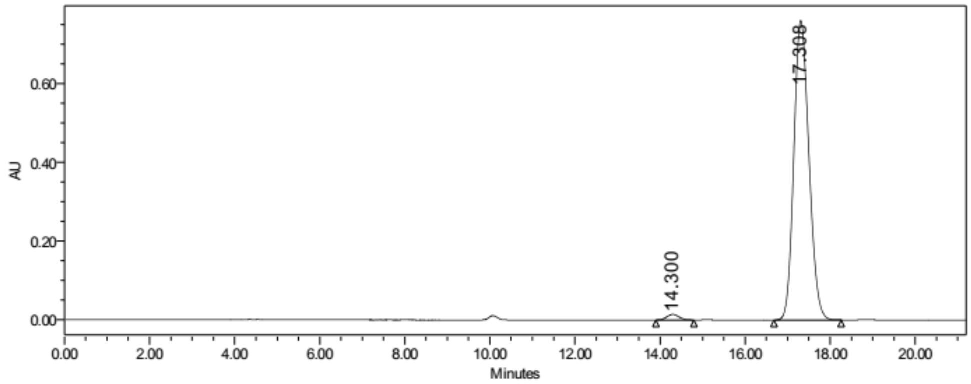

	RT	Area	% Area	Height
1	14.300	284256	1.48	14055
2	17.308	18951204	98.52	760144

Rac-**3ag**

SAMPLE INFORMATION

Sample Name:	cxh-7-43-1-rac-IC-2%	Acquired By:	System
Sample Type:	Unknown	Sample Set Name:	
Vial:	26	Acq. Method Set:	2%
Injection #:	1	Processing Method	3ac
Injection Volume:	10.00 ul	Channel Name:	254.0nm
Run Time:	40.0 Minutes	Proc. Chnl. Descr.:	2998 PDA 254.0 nm (2998
Date Acquired:	5/25/2020 2:34:52 PM CST		
Date Processed:	8/22/2020 5:57:27 PM CST		

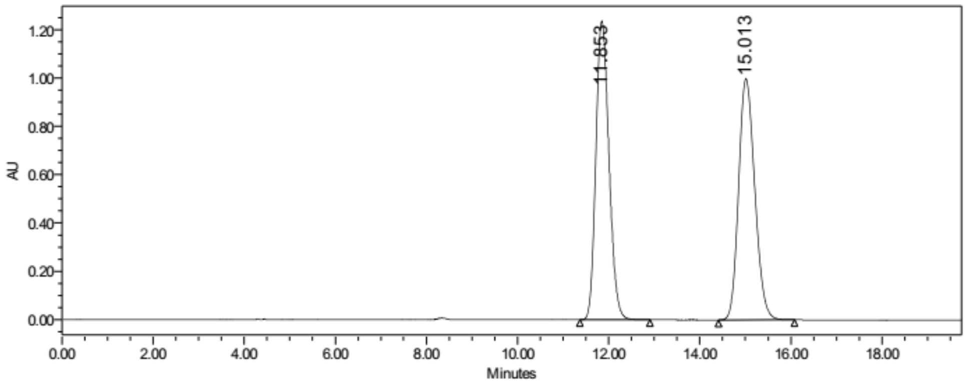

	RT	Area	% Area	Height
1	11.853	24635357	49.84	1237146
2	15.013	24792845	50.16	998789

Asy-**3ag**

SAMPLE INFORMATION

Sample Name:	cxh-7-63-4-IC-2%	Acquired By:	System
Sample Type:	Unknown	Sample Set Name:	
Vial:	55	Acq. Method Set:	2%
Injection #:	1	Processing Method	3ac2
Injection Volume:	10.00 ul	Channel Name:	254.0nm
Run Time:	40.0 Minutes	Proc. Chnl. Descr.:	2998 PDA 254.0 nm (2998
Date Acquired:	5/25/2020 2:59:29 PM CST		
Date Processed:	8/22/2020 5:56:39 PM CST		

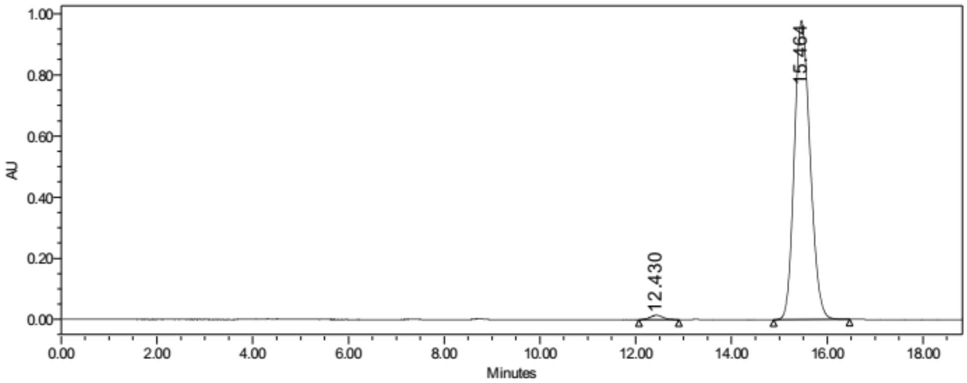

	RT	Area	% Area	Height
1	12.430	262793	1.15	14387
2	15.464	22616239	98.85	977564

Rac-**3ah**

SAMPLE INFORMATION

Sample Name:	cxh-7-37-4-rac-OJ-1%	Acquired By:	System
Sample Type:	Unknown	Sample Set Name:	
Vial:	4	Acq. Method Set:	1%
Injection #:	3	Processing Method	3ad
Injection Volume:	10.00 ul	Channel Name:	211.0nm
Run Time:	80.0 Minutes	Proc. Chnl. Descr.:	2998 PDA 211.0 nm (2998
Date Acquired:	5/27/2020 3:42:03 PM CST		
Date Processed:	8/22/2020 8:37:30 PM CST		

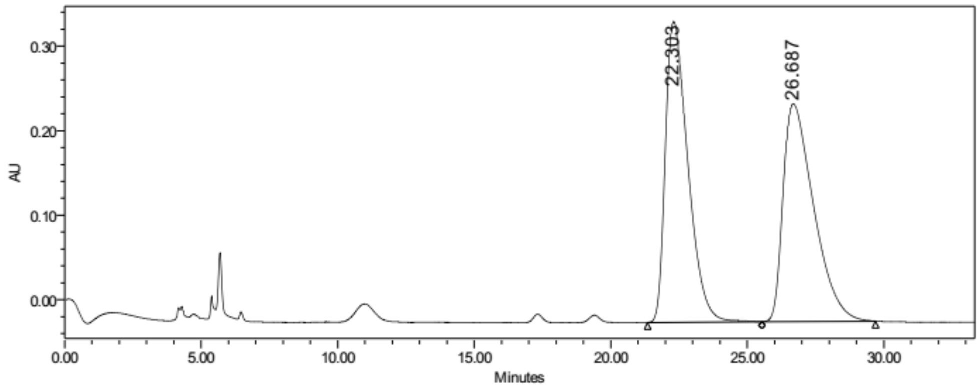

	RT	Area	% Area	Height
1	22.303	20295150	50.32	355875
2	26.687	20039357	49.68	257804

Asy-**3ah**

SAMPLE INFORMATION

Sample Name:	cxh-7-65-2-OJ-1%	Acquired By:	System
Sample Type:	Unknown	Sample Set Name:	
Vial:	29	Acq. Method Set:	1%
Injection #:	1	Processing Method	3ad2
Injection Volume:	10.00 ul	Channel Name:	211.0nm
Run Time:	80.0 Minutes	Proc. Chnl. Descr.:	2998 PDA 211.0 nm (2998
Date Acquired:	5/27/2020 4:20:15 PM CST		
Date Processed:	8/22/2020 8:37:17 PM CST		

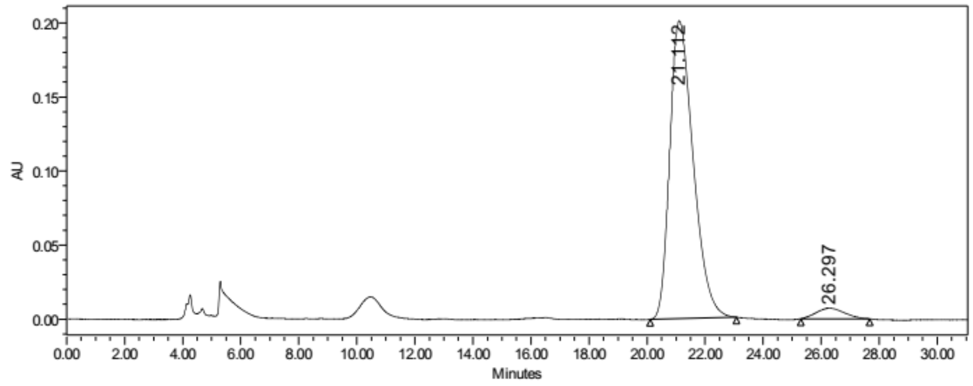

	RT	Area	% Area	Height
1	21.112	11266770	95.85	200950
2	26.297	487849	4.15	7198

Rac-**3ai**

SAMPLE INFORMATION

Sample Name:	cxh-7-42-5-RAC-IC-1%	Acquired By:	System
Sample Type:	Unknown	Sample Set Name:	
Vial:	6	Acq. Method Set:	1%
Injection #:	1	Processing Method	3ae
Injection Volume:	10.00 ul	Channel Name:	211.0nm
Run Time:	80.0 Minutes	Proc. Chnl. Descr.:	2998 PDA 211.0 nm (2998
Date Acquired:	5/25/2020 9:25:43 PM CST		
Date Processed:	8/22/2020 8:42:09 PM CST		

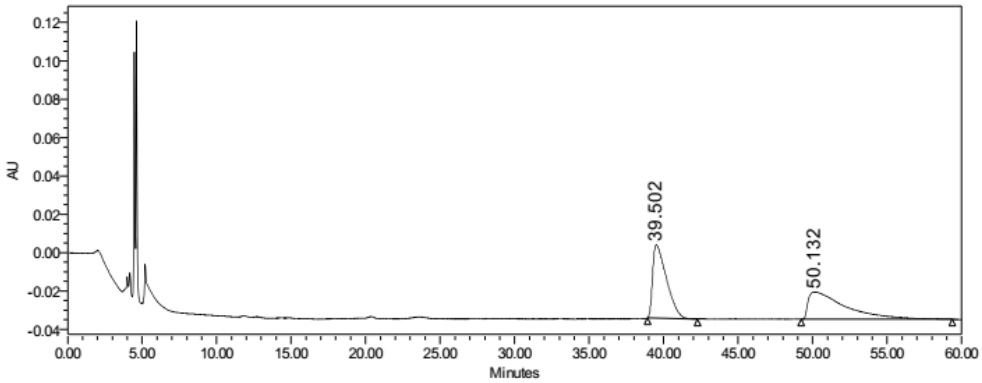

	RT	Area	% Area	Height
1	39.502	2446122	49.52	38200
2	50.132	2493845	50.48	14247

Asy-**3ai**

SAMPLE INFORMATION

Sample Name:	cxh-7-63-5-IC-1%	Acquired By:	System
Sample Type:	Unknown	Sample Set Name:	
Vial:	37	Acq. Method Set:	1%
Injection #:	1	Processing Method	3ae2
Injection Volume:	10.00 ul	Channel Name:	211.0nm
Run Time:	80.0 Minutes	Proc. Chnl. Descr.:	2998 PDA 211.0 nm (2998
Date Acquired:	5/25/2020 10:26:45 PM CST		
Date Processed:	8/22/2020 8:41:10 PM CST		

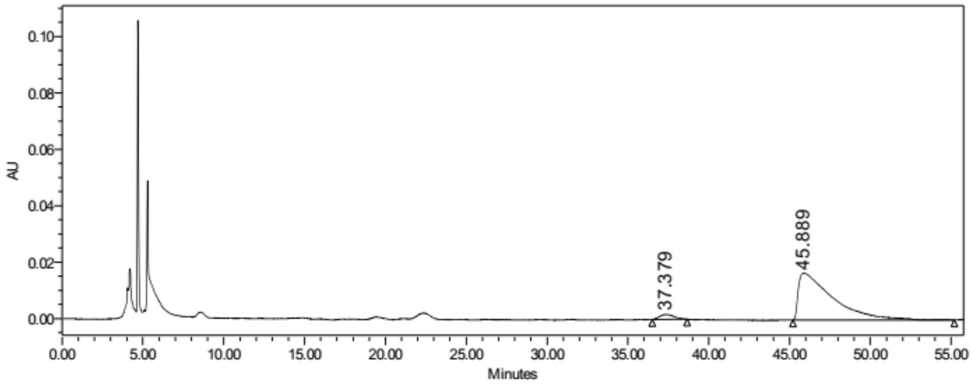

	RT	Area	% Area	Height
1	37.379	104366	4.09	1716
2	45.889	2444391	95.91	16654

Rac-**3aj**

SAMPLE INFORMATION

Sample Name:	cxh-7-37-3-RAC-IC-1%	Acquired By:	System
Sample Type:	Unknown	Sample Set Name:	
Vial:	62	Acq. Method Set:	1%
Injection #:	1	Processing Method	3af
Injection Volume:	10.00 ul	Channel Name:	211.0nm
Run Time:	80.0 Minutes	Proc. Chnl. Descr.:	2998 PDA 211.0 nm (2998
Date Acquired:	5/27/2020 9:10:52 PM CST		
Date Processed:	8/22/2020 8:45:08 PM CST		

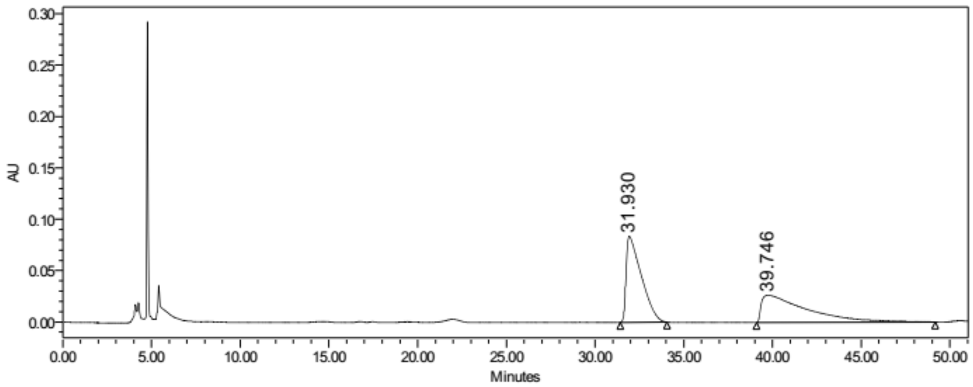

	RT	Area	% Area	Height
1	31.930	4997117	51.18	83905
2	39.746	4767568	48.82	26575

Asy-**3aj**

SAMPLE INFORMATION

Sample Name:	cxh-7-65-1-IC-1%	Acquired By:	System
Sample Type:	Unknown	Sample Set Name:	
Vial:	56	Acq. Method Set:	1%
Injection #:	1	Processing Method	3af2
Injection Volume:	10.00 ul	Channel Name:	211.0nm
Run Time:	80.0 Minutes	Proc. Chnl. Descr.:	2998 PDA 211.0 nm (2998
Date Acquired:	5/27/2020 10:03:07 PM CST		
Date Processed:	8/22/2020 8:46:40 PM CST		

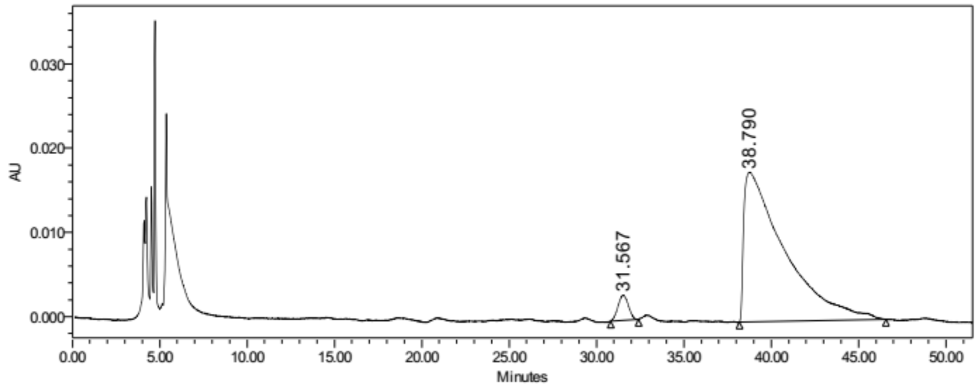

	RT	Area	% Area	Height
1	31.567	122591	4.08	2980
2	38.790	2884558	95.92	17774

Rac-**5**

SAMPLE INFORMATION

Sample Name:	cxh-5-rac-IG-0.3ML 1%	Acquired By:	System
Sample Type:	Unknown	Sample Set Name	
Vial:	71	Acq. Method Set:	03ml 1%
Injection #:	5	Processing Method	5 rac
Injection Volume:	10.00 ul	Channel Name:	220.0nm
Run Time:	30.0 Minutes	Proc. Chnl. Descr.:	2998 PDA 220.0 nm (2998
Date Acquired:	10/20/2020 10:52:37 AM CST		
Date Processed:	10/28/2020 3:03:41 PM CST		

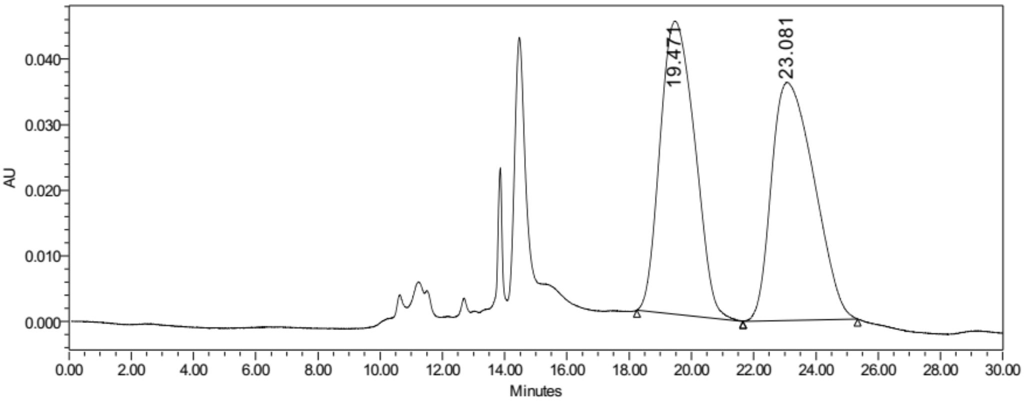

	RT	Area	% Area	Height
1	19.471	3455819	49.97	44676
2	23.081	3460036	50.03	36293

Asy-**5**

SAMPLE INFORMATION

Sample Name:	cxh-5-asy-IG-0.3ML 1%	Acquired By:	System
Sample Type:	Unknown	Sample Set Name	
Vial:	77	Acq. Method Set:	03 ml 1%
Injection #:	1	Processing Method	5 asy
Injection Volume:	10.00 ul	Channel Name:	220.0nm
Run Time:	30.0 Minutes	Proc. Chnl. Descr.:	2998 PDA 220.0 nm (2998
Date Acquired:	10/20/2020 8:31:27 PM CST		
Date Processed:	10/28/2020 3:00:49 PM CST		

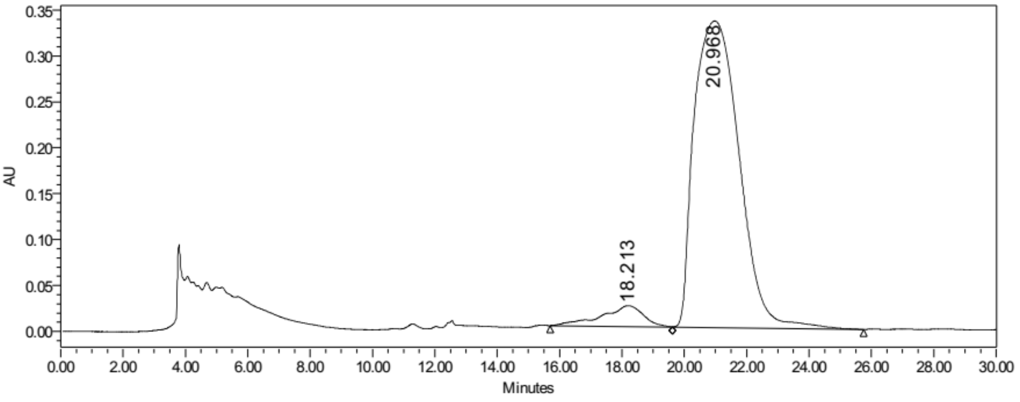

	RT	Area	% Area	Height
1	18.213	2107983	5.91	22779
2	20.968	33544425	94.09	333946

Rac-**6**

SAMPLE INFORMATION

Sample Name:	cxh-7-91-1-RAC-IF-10%	Acquired By:	System
Sample Type:	Unknown	Sample Set Name:	
Vial:	76	Acq. Method Set:	10%
Injection #:	1	Processing Method	6
Injection Volume:	10.00 ul	Channel Name:	300.0nm
Run Time:	40.0 Minutes	Proc. Chnl. Descr.:	2998 PDA 300.0 nm (2998
Date Acquired:	8/22/2020 2:50:30 PM CST		
Date Processed:	8/22/2020 4:56:22 PM CST		

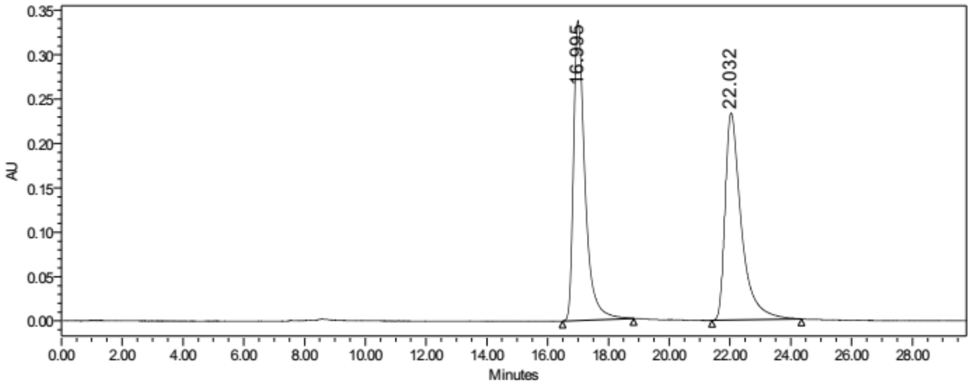

	RT	Area	% Area	Height
1	16.995	8844804	50.63	337760
2	22.032	8625382	49.37	233457

Asy-**6**

SAMPLE INFORMATION

Sample Name:	cxh-8-5-1-IF-10%	Acquired By:	System
Sample Type:	Unknown	Sample Set Name:	
Vial:	75	Acq. Method Set:	10%
Injection #:	1	Processing Method	62
Injection Volume:	10.00 ul	Channel Name:	300.0nm
Run Time:	40.0 Minutes	Proc. Chnl. Descr.:	2998 PDA 300.0 nm (2998
Date Acquired:	8/22/2020 3:55:19 PM CST		
Date Processed:	8/22/2020 4:56:05 PM CST		

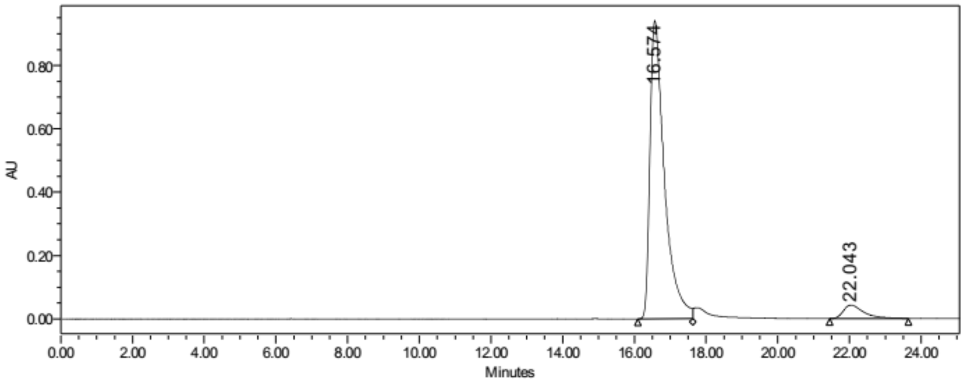

	RT	Area	% Area	Height
1	16.574	26238451	93.98	940869
2	22.043	1681600	6.02	42818

Rac-**7**

SAMPLE INFORMATION

Sample Name:	cxh-7-rac-IC-0.3ml 1%	Acquired By:	System
Sample Type:	Unknown	Sample Set Name	
Vial:	23	Acq. Method Set:	03 ml 1%
Injection #:	1	Processing Method	7rac
Injection Volume:	10.00 ul	Channel Name:	211.0nm
Run Time:	40.0 Minutes	Proc. Chnl. Descr.:	2998 PDA 211.0 nm (2998
Date Acquired:	10/22/2020 11:37:07 PM CST		
Date Processed:	10/23/2020 12:16:50 AM CST		

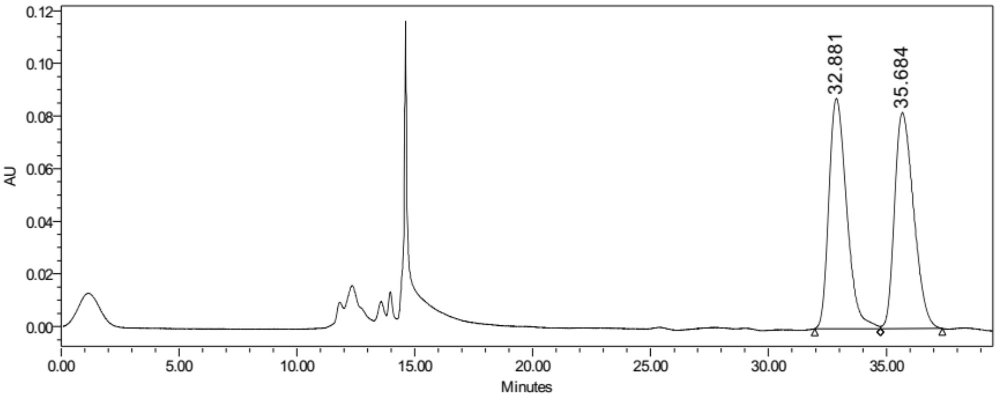

	RT	Area	% Area	Height
1	32.881	4627166	50.08	87537
2	35.684	4612829	49.92	82124

Asy-**7**

SAMPLE INFORMATION

Sample Name:	cxh-7-asy-IC-0.3ml 1%	Acquired By:	System
Sample Type:	Unknown	Sample Set Name	
Vial:	47	Acq. Method Set:	03 ml 1%
Injection #:	2	Processing Method	7asy
Injection Volume:	10.00 ul	Channel Name:	211.0nm
Run Time:	65.0 Minutes	Proc. Chnl. Descr.:	2998 PDA 211.0 nm (2998
Date Acquired:	10/22/2020 10:51:36 PM CST		
Date Processed:	10/22/2020 11:32:09 PM CST		

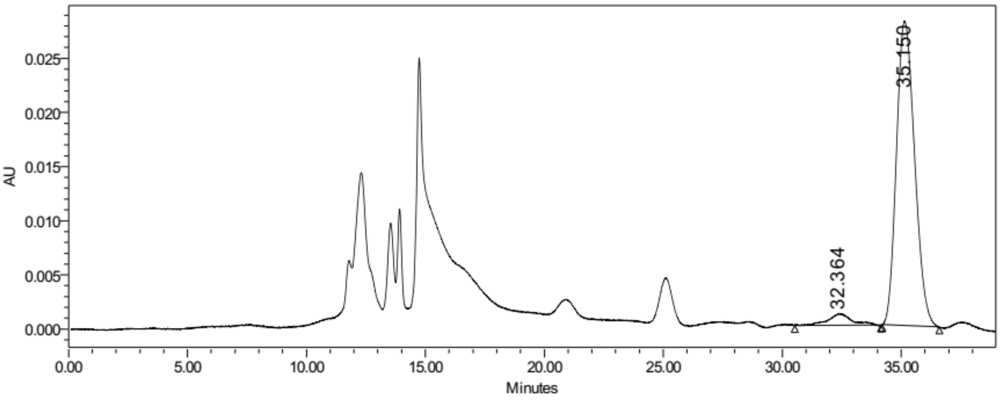

	RT	Area	% Area	Height
1	32.364	76705	4.88	1089
2	35.150	1493983	95.12	28131

Rac-**9**

SAMPLE INFORMATION			
Sample Name:	cxh-7-79-4-rac-IF-10%	Acquired By:	System
Sample Type:	Unknown	Sample Set Name:	
Vial:	30	Acq. Method Set:	10%
Injection #:	1	Processing Method	9
Injection Volume:	10.00 ul	Channel Name:	300.0nm
Run Time:	40.0 Minutes	Proc. Chnl. Descr.:	2998 PDA 300.0 nm (2998
Date Acquired:	8/12/2020 3:48:15 PM CST		
Date Processed:	8/22/2020 8:59:21 PM CST		

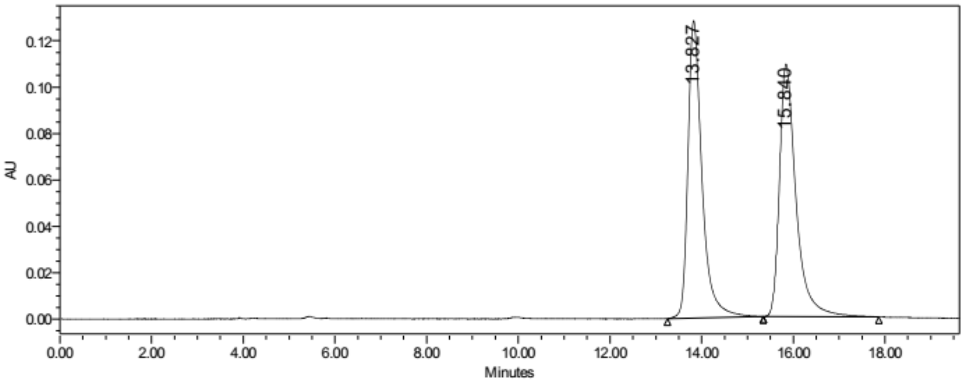

	RT	Area	% Area	Height
1	13.827	2753809	50.13	128285
2	15.840	2739601	49.87	108927

Asy-**9**

SAMPLE INFORMATION			
Sample Name:	cxh-7-97-4-IF-10%	Acquired By:	System
Sample Type:	Unknown	Sample Set Name:	
Vial:	117	Acq. Method Set:	10%
Injection #:	1	Processing Method	92
Injection Volume:	10.00 ul	Channel Name:	300.0nm
Run Time:	40.0 Minutes	Proc. Chnl. Descr.:	2998 PDA 300.0 nm (2998
Date Acquired:	8/12/2020 2:58:23 PM CST		
Date Processed:	8/22/2020 8:58:45 PM CST		

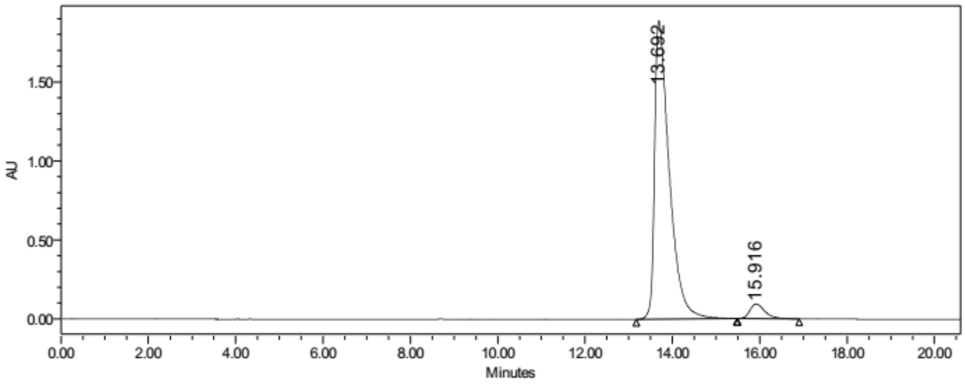

	RT	Area	% Area	Height
1	13.692	43764808	95.16	1889142
2	15.916	2227199	4.84	93425

Rac-**10**

SAMPLE INFORMATION

Sample Name:	cxh-7-97-2-IF-rac-5%	Acquired By:	System
Sample Type:	Unknown	Sample Set Name:	
Vial:	108	Acq. Method Set:	5%
Injection #:	3	Processing Method	10
Injection Volume:	10.00 ul	Channel Name:	211.0nm
Run Time:	60.0 Minutes	Proc. Chnl. Descr.:	2998 PDA 211.0 nm (2998
Date Acquired:	8/11/2020 10:57:34 AM CST		
Date Processed:	8/22/2020 9:01:23 PM CST		

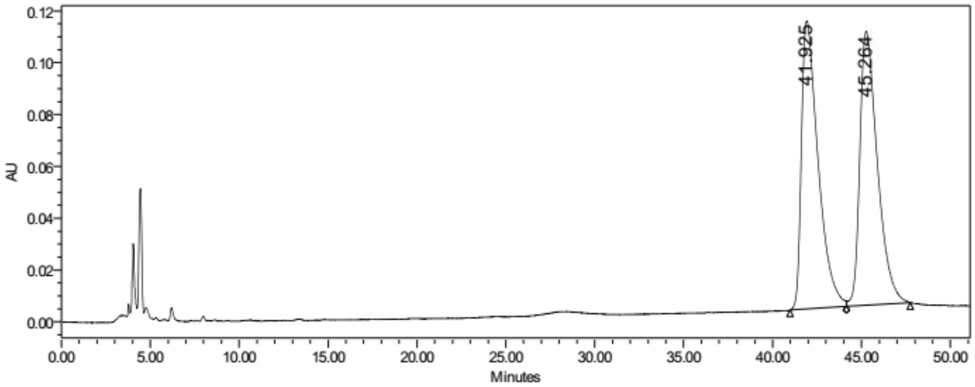

	RT	Area	% Area	Height
1	41.925	7296208	49.97	111270
2	45.264	7303579	50.03	105691

Asy-**10**

SAMPLE INFORMATION

Sample Name:	cxh-7-97-5-IF-5%	Acquired By:	System
Sample Type:	Unknown	Sample Set Name:	
Vial:	51	Acq. Method Set:	5%
Injection #:	1	Processing Method	102
Injection Volume:	10.00 ul	Channel Name:	211.0nm
Run Time:	70.0 Minutes	Proc. Chnl. Descr.:	2998 PDA 211.0 nm (2998
Date Acquired:	8/12/2020 4:19:27 PM CST		
Date Processed:	8/22/2020 9:02:32 PM CST		

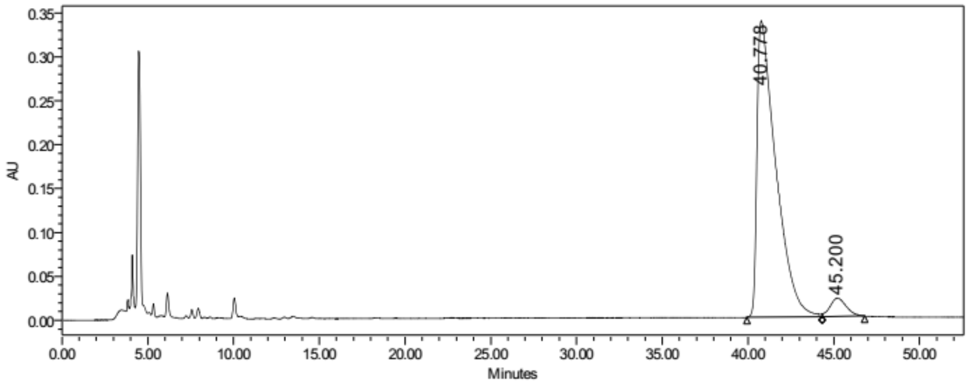

	RT	Area	% Area	Height
1	40.778	25795559	94.91	337630
2	45.200	1382517	5.09	20903

Rac-**11**

SAMPLE INFORMATION

Sample Name:	cxh-7-61-4-rac-AD-20%	Acquired By:	System
Sample Type:	Unknown	Sample Set Name:	
Vial:	5	Acq. Method Set:	20%
Injection #:	1	Processing Method	11
Injection Volume:	10.00 ul	Channel Name:	254.0nm
Run Time:	100.0 Minutes	Proc. Chnl. Descr.:	2998 PDA 254.0 nm (2998
Date Acquired:	5/22/2020 2:22:26 PM CST		
Date Processed:	8/22/2020 9:08:45 PM CST		

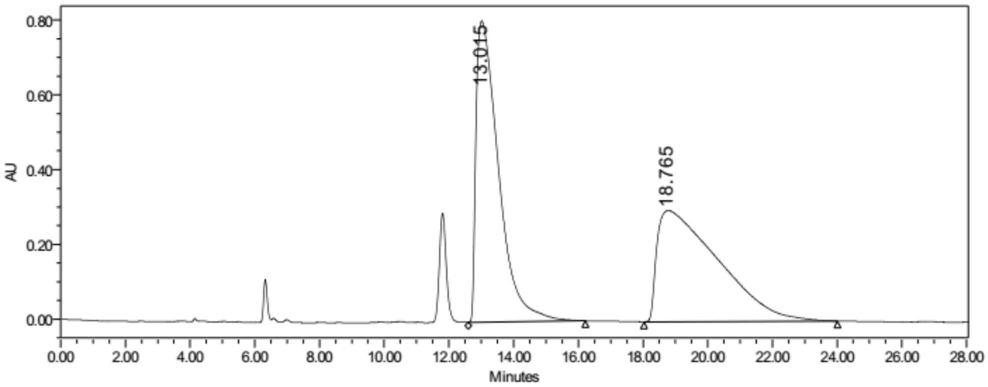

	RT	Area	% Area	Height
1	13.015	39170209	50.12	804821
2	18.765	38982911	49.88	297130

Asy-**11**

SAMPLE INFORMATION

Sample Name:	cxh-7-61-5-AD-20%	Acquired By:	System
Sample Type:	Unknown	Sample Set Name:	
Vial:	7	Acq. Method Set:	20%
Injection #:	1	Processing Method	112
Injection Volume:	10.00 ul	Channel Name:	254.0nm
Run Time:	100.0 Minutes	Proc. Chnl. Descr.:	2998 PDA 254.0 nm (2998
Date Acquired:	5/22/2020 3:27:40 PM CST		
Date Processed:	8/22/2020 9:10:26 PM CST		

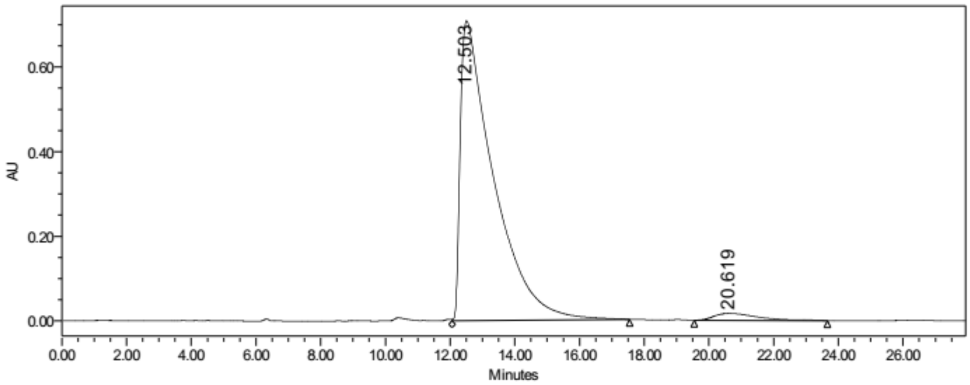

	RT	Area	% Area	Height
1	12.503	50703531	97.08	708651
2	20.619	1524809	2.92	16456

Rac-**13**

SAMPLE INFORMATION

Sample Name:	cxh-7-68-3-rac-OJ-2%	Acquired By:	System
Sample Type:	Unknown	Sample Set Name:	
Vial:	17	Acq. Method Set:	2%
Injection #:	1	Processing Method	12
Injection Volume:	10.00 ul	Channel Name:	254.0nm
Run Time:	50.0 Minutes	Proc. Chnl. Descr.:	2998 PDA 254.0 nm (2998
Date Acquired:	6/23/2020 9:43:59 AM CST		
Date Processed:	8/22/2020 9:22:23 PM CST		

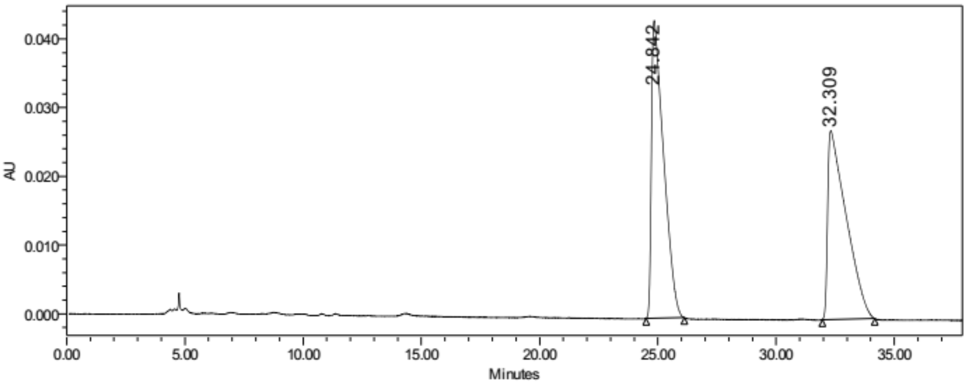

	RT	Area	% Area	Height
1	24.842	1552904	51.05	43295
2	32.309	1489043	48.95	27468

Asy-**13**

SAMPLE INFORMATION

Sample Name:	cxh-7-68-2-OJ-2%	Acquired By:	System
Sample Type:	Unknown	Sample Set Name:	0623
Vial:	32	Acq. Method Set:	2%
Injection #:	1	Processing Method	122
Injection Volume:	10.00 ul	Channel Name:	254.0nm
Run Time:	40.0 Minutes	Proc. Chnl. Descr.:	2998 PDA 254.0 nm (2998
Date Acquired:	6/23/2020 10:24:36 AM CST		
Date Processed:	8/22/2020 9:26:25 PM CST		

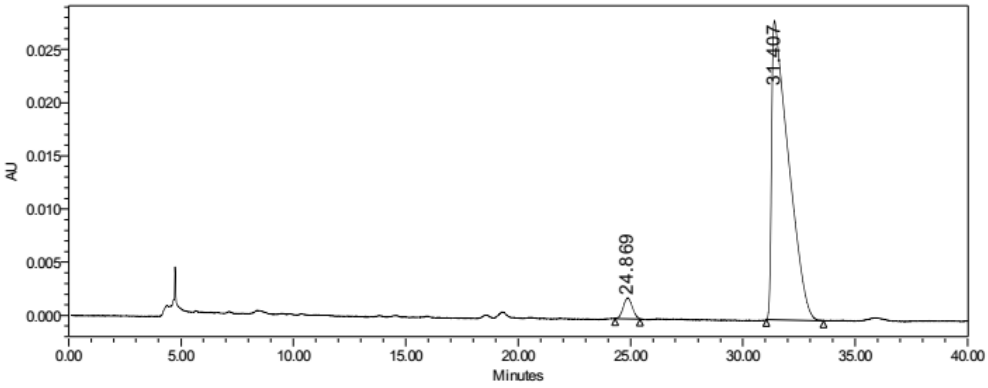

	RT	Area	% Area	Height
1	24.869	54437	3.59	1977
2	31.407	1459985	96.41	28114

Rac-**14**

SAMPLE INFORMATION

Sample Name:	cxh-7-76-1-re-rac-IB-2%	Acquired By:	System
Sample Type:	Unknown	Sample Set Name:	
Vial:	15	Acq. Method Set:	2%
Injection #:	3	Processing Method	14
Injection Volume:	10.00 ul	Channel Name:	310.0nm
Run Time:	30.0 Minutes	Proc. Chnl. Descr.:	2998 PDA 310.0 nm (2998
Date Acquired:	6/27/2020 5:24:32 PM CST		
Date Processed:	8/22/2020 9:30:42 PM CST		

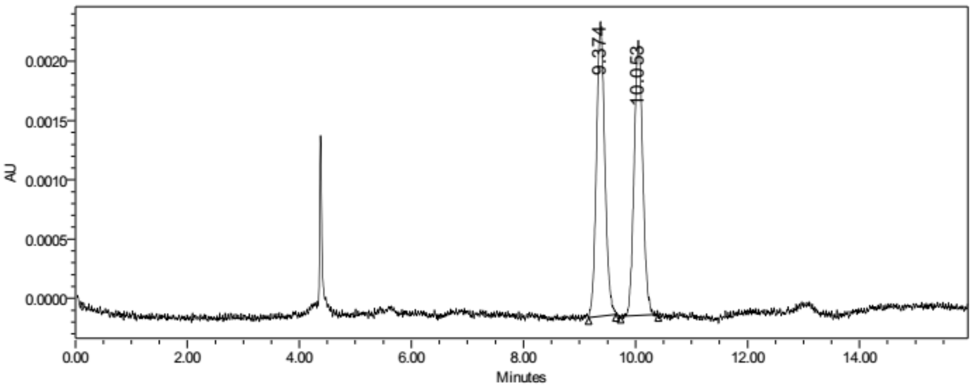

	RT	Area	% Area	Height
1	9.374	25581	50.84	2486
2	10.053	24740	49.16	2322

Asy-**14**

SAMPLE INFORMATION

Sample Name:	cxh-7-76-2-re-rac-IB-2%	Acquired By:	System
Sample Type:	Unknown	Sample Set Name:	
Vial:	16	Acq. Method Set:	2%
Injection #:	1	Processing Method	142
Injection Volume:	10.00 ul	Channel Name:	310.0nm
Run Time:	30.0 Minutes	Proc. Chnl. Descr.:	2998 PDA 310.0 nm (2998
Date Acquired:	6/27/2020 5:48:10 PM CST		
Date Processed:	8/22/2020 9:31:24 PM CST		

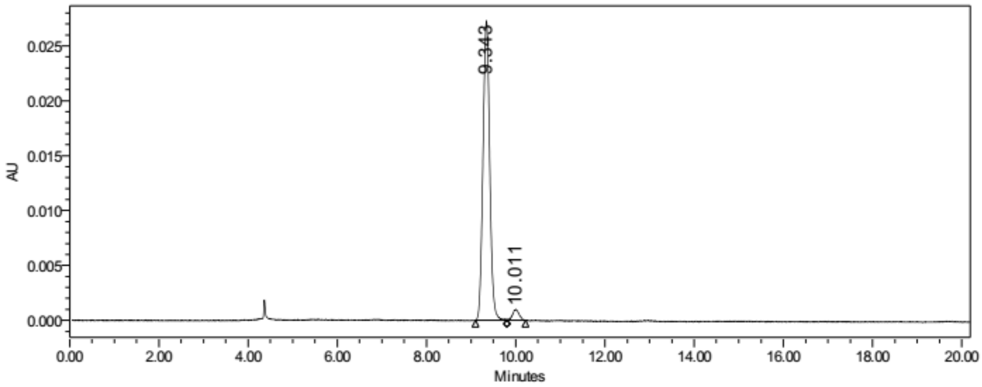

	RT	Area	% Area	Height
1	9.343	285798	96.55	27279
2	10.011	10215	3.45	997

Rac-**15**

SAMPLE INFORMATION

Sample Name:	cxh-7-76-3-rac-OD-5%	Acquired By:	System
Sample Type:	Unknown	Sample Set Name:	
Vial:	66	Acq. Method Set:	5%
Injection #:	2	Processing Method	15
Injection Volume:	10.00 ul	Channel Name:	211.0nm
Run Time:	50.0 Minutes	Proc. Chnl. Descr.:	2998 PDA 211.0 nm (2998
Date Acquired:	7/6/2020 9:32:59 PM CST		
Date Processed:	8/22/2020 9:33:03 PM CST		

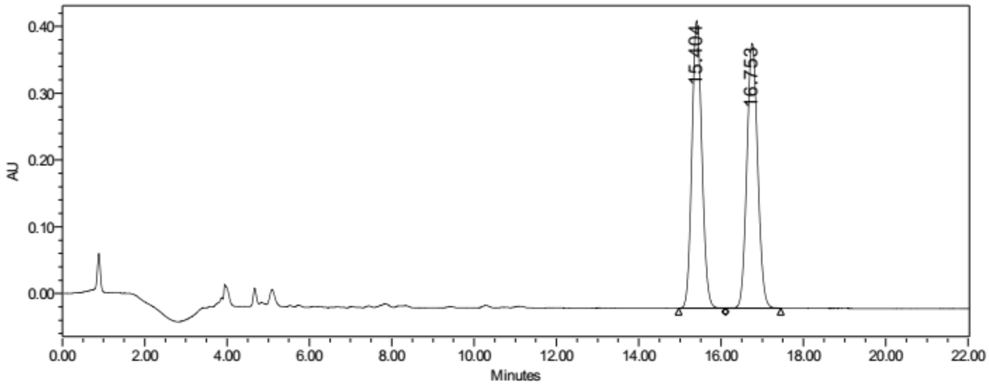

	RT	Area	% Area	Height
1	15.404	7300594	50.02	430801
2	16.753	7295148	49.98	396680

Asy-**15**

SAMPLE INFORMATION

Sample Name:	cxh-7-76-4-rac-OD-5%	Acquired By:	System
Sample Type:	Unknown	Sample Set Name:	
Vial:	67	Acq. Method Set:	5%
Injection #:	1	Processing Method	152
Injection Volume:	10.00 ul	Channel Name:	211.0nm
Run Time:	50.0 Minutes	Proc. Chnl. Descr.:	2998 PDA 211.0 nm (2998
Date Acquired:	7/6/2020 9:56:18 PM CST		
Date Processed:	8/22/2020 9:33:50 PM CST		

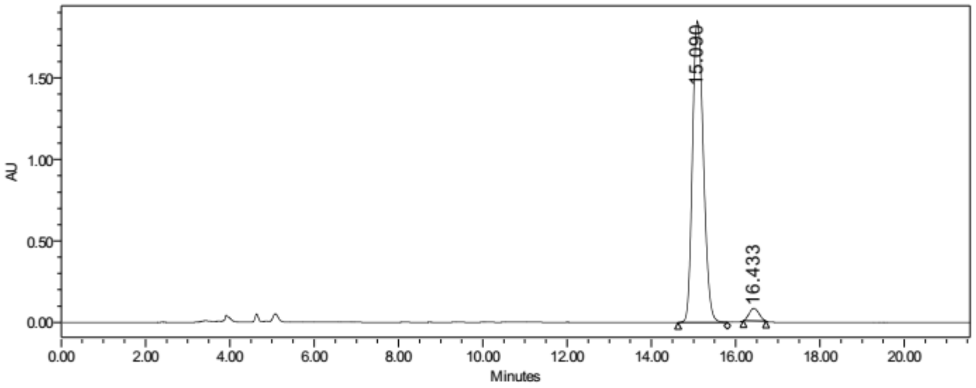

	RT	Area	% Area	Height
1	15.090	33464248	96.40	1850417
2	16.433	1249618	3.60	77626

13. Supplementary References

1. Watanabe, K., Miyazaki, Y., Okubo, M., Zhou, B., Tsuji, H. & Kawatsura, M. Nickel-catalyzed asymmetric propargylic amination of propargylic carbonates bearing an internal alkyne group. *Org. Lett*. **20**, 5448-5451 (2018).
2. Zhou, Z., Liu, G., Chen, Y. & Lu, X. Cascade synthesis of 3-alkylidene dihydrobenzofuran derivatives via rhodium(III)-catalyzed redox-neutral C−H functionalization/cyclization. *Org. Lett*. **17**, 5874-5877 (2015).
3. Tanaka, K. & Shoji, T. Cationic rhodium(I)/BINAP complex-catalyzed isomerization of secondary propargylic alcohols to α,β-enones. *Org. Lett*. **7**, 3561-3563 (2005).
4. Werner, E. W., Mei, T.-S., Burckle, A. J. & Sigman, M. S. Enantioselective heck arylations of acyclic alkenyl alcohols using a redox-relay strategy. *Science* **338**, 1455-1458 (2012).
5. Darwish, K. M., Salama, I., Mostafa, S., Gomaa, M. S. & Helal, M. A. Design, synthesis, and biological evaluation of novel thiazolidinediones as PPARγ/FFAR1 dual agonists. *Eur. J. Med. Chem*. **109**, 157-172 (2016).
6. Cook, C. E. & Tallent, C. R. Synthesis of (R)-5-(2-pentyl)barbituric acid derivatives of high optical purity. *J. Heterocycl. Chem*. **6**, 203-206 (1969).
7. Huang, G., Cheng, C., Ge, L., Guo, B., Zhao, L. & Wu, X. Trialkyl methanetricarboxylate as dialkyl malonate surrogate in copper-catalyzed enantioselective propargylic substitution. *Org. Lett*. **17**, 4894-4897 (2015).
8. Du, Y., Huang, H.-Y., Liu, H., Ruan, Y.-P. & Huang, P.-Q. Stereoselective total syntheses of (-)-desoxoprosopinine and (-)-desoxoprosophylline: palladium(0)-catalyzed intramolecular *N*-alkylation for the key piperidine ring formation. *Synlett* **4**, 565-567 (2011).
9. Superchi, S., Marchitiello, V., Pisani, L. & Scafato, P. Asymmetric addition of dimethylzinc to alkylidenmalonates mediated by phosphorous ligands: A new synthetic route to floral fragrances. *Chirality* **23**, 761-767 (2011).
